# WaterRAG: A Multiagent Retrieval-Augmented Generation Framework to Support Water Industry Transitions to Net-Zero

**DOI:** 10.1021/acs.est.5c15806

**Published:** 2026-04-07

**Authors:** Mudi Zhai, Qingyun Zeng, Ruihong Qiu, Jiaying Li, Qixiang Zhu, T. David Waite, Bing-Jie Ni, Haoran Duan

**Affiliations:** † UNSW Water Research Centre, School of Civil and Environmental Engineering, The University of New South Wales, Sydney, NSW 2052, Australia; ‡ 6834 Microsoft Copilot Studio AI, Redmond, Washington 98052, United States; § Departments of Mathematics & Department of Computer and Information Science, University of Pennsylvania, Philadelphia, Pennsylvania 19104, United States; ∥ School of Electrical Engineering and Computer Science, 1974 The University of Queensland, Brisbane, QLD 4072, Australia; ⊥ School of Civil Engineering, The University of Sydney, Sydney, NSW 2050, Australia; # Department of Civil Engineering, 25809 The University of Hong Kong, Pokfulam, Hong Kong SAR, China

**Keywords:** retrieval-augmented generation (RAG), agent, large language model (LLM), wastewater treatment, net-zero

## Abstract

Achieving net-zero carbon emissions in wastewater treatment requires a complex, interdisciplinary information integration. While large language models (LLMs) have the potential to break down information silos, their capabilities remain limited in specialized wastewater domains. Therefore, we introduce WaterRAG, a multiagent retrieval-augmented generation (RAG) framework that couples LLM reasoning with verifiable wastewater knowledge. WaterRAG integrates a selective database of 7637 peer-reviewed studies and 11 engineering references on wastewater treatment. Through iterative collaboration among retrieval, review, and evaluation agents, WaterRAG produces evidence-based output for three critical applications in wastewater management: (i) wastewater expert-level question answering, (ii) literature review on specific topics, and (iii) plant-specific engineering scientific support. In benchmarking of 370 technical questions, WaterRAG achieved an 80.5% answer correctness rate on professional wastewater treatment questions, outperforming standalone GPT-4.1 (64.9%). WaterRAG also generated a more comprehensive citation-supported review, with quality improving across refinement iterations. Ablation experiments confirm that the superior performance arises from the synergistic contributions of optimized retrieval and iterative multiagent framework. This work represents an early domain-specific application of multiagent RAG to wastewater treatment, highlighting the potential of retrieval-grounded LLM systems to complement professional expertise and support evidence-based decision-making toward sustainable wastewater management.

## Graphical abstract

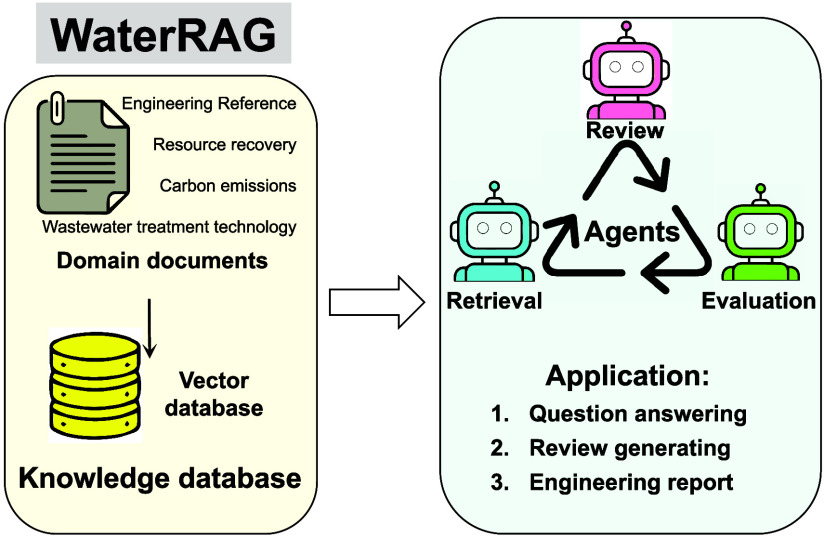

## Introduction

As the fifth-largest source of CH_4_ and the third-largest source of N_2_O globally, the wastewater sector is undergoing a paradigm shift toward integrating carbon emissions management as a core component of both operational practice and strategic planning, beyond its traditional focus on pollutant removal.^1^ Water utilities around the world, including the United Kingdom, Australia, Denmark, and New Zealand, have committed to achieving net-zero by 2050, aligning with the UN Race to Zero initiative.^2,3^ However, this transition remains challenging, with many barriers to achieving the goal. Wastewater treatment operations are inconsistent across plants and regions, while greenhouse gas (GHG) emissions from different treatment processes are highly variable.^4^ Technical solutions, ranging from advanced process configurations to carbon and energy recovery technologies, are developing faster than practitioners can readily adopt.^5–9^ This requires policymakers and utility engineers to cope with the increasing knowledge translation bottleneck. Currently, critical evidence is scattered across thousands of newly published scientific articles and engineering documents, making it challenging to obtain consistent and actionable insights.

Large language models (LLMs) have transformed how knowledge can be accessed and synthesized across domains, opening new opportunities for addressing complex wastewater engineering challenges.^10–13^ Some powerful general-purpose LLMs, such as GPT,^14^ Gemini,^15^ and Claude,^16^ provide satisfactory answers across general-purpose tasks. However, in highly specialized fields, general-purpose LLMs still exhibit limitations, mainly reflected by the lack of professional domain knowledge, leading to insufficient capability to solve complex engineering problems.^17,18^ To improve the question answering (QA) ability of LLMs in the wastewater treatment domain, recent studies have applied fine-tuning techniques.^17,19^ For example, Xu et al.^19^ employed prompt engineering methods and fine-tuned the open-source Llama3–8B model, developing a specialized model called WaterGPT. Compared to the baseline model (Llama3–8B), the performance of WaterGPT in addressing water engineering tasks and research tasks improved by 135.4 and 18.8%, respectively.

Although recent studies have embedded domain knowledge by fine-tuning general-purpose LLMs, the critical limitations of fine-tuning methods are obvious. Fine-tuning large-scale LLMs is often costly and challenging. Fine-tuning small-scale LLMs is comparatively straightforward, yet their performance may not surpass that of general-purpose large-scale LLMs. Zhu et al.^17^ fine-tuned several models based on GPT-3.5 for environmental engineering, but these did not outperform the baseline model. For example, regarding the topic of “denitrification”, a fine-tuned model (FTM3–3EP) produced a significant number of factually incorrect responses (Factuality ≈ 0.465). In the study by Xu et al.,^19^ WaterGPT surpassed a small-scale baseline model (Llama 3–8B) but did not match mainstream large-scale LLMs such as GPT-4. These issues stem from the fact that domain knowledge is stored implicitly in the model’s parameters, which are difficult to train effectively.^20^ Consequently, hallucinations can appear more likely when LLMs encounter untrained highly specialized domains as they may generate outputs that are statistically likely but factually incorrect.^21^

Beyond fine-tuning, retrieval-augmented generation (RAG) frameworks have emerged as a promising solution for empowering LLMs with domain-specific knowledge. Unlike fine-tuning, which primarily adapts model behavior by updating parameters and storing knowledge implicitly, RAG generates outputs supported by externally retrieved sources, thereby reducing hallucinations and improving reliability through evidence citations.^22^ Moreover, empowered by multiagent collaboration, agentic RAG exhibits robust capabilities in complex reasoning and problem-solving. Through distributed task decomposition and logical validation, agents collaboratively overcome single-model cognitive limits, enabling high-level autonomous decision-making and continuous execution.^22,23^ Multiagent collaborative RAG frameworks have demonstrated promising results on complex tasks across multiple domains, including biology, chemistry, and medicine.^12,24–26^ For example, Ferber et al.^24^ developed an autonomous clinical agent system that combines RAG with a multitool chain, integrating various external tools such as medical image analysis, genetic testing, and literature retrieval. When handling 20 real-world tumor diagnosis cases, this system improved decision-making completeness and accuracy from 30.3% with GPT-4 to 87.2%. However, no such framework has been designed for the wastewater sector, where processing advanced scientific and engineering information and providing transparent, auditable knowledge support are crucial for the emerging net-zero challenge.

Here, we introduce WaterRAG, a multiagent RAG framework specifically designed for wastewater treatment and carbon emissions management. WaterRAG integrates a domain-specific knowledge base of 7637 selected peer-reviewed articles and 11 engineering references. By iterative coordination of retrieval, review, and evaluation agents, WaterRAG supports wastewater by grounding outputs in retrieved evidence and refining them through agentic evaluation, enabling literature-supported QA, review generation, and engineering decision support. The performance of standalone LLMs, Naive RAG systems, and the proposed WaterRAG framework was systematically evaluated in domain-specific wastewater treatment tasks. By consolidating extensive domain knowledge into accessible, verifiable, and task-relevant outputs, WaterRAG provides efficient and intelligent support for utilities, researchers, and policymakers pursuing net-zero.

## Methods

### Data Set Preparation

As the essential factual foundation, the knowledge base dictates the reliability of the RAG system’s outputs. The knowledge base for this study comprises two types of knowledge sources: the scientific literature and engineering sources. For scientific literature, we focused on three themes closely related to achieving net-zero carbon emissions in wastewater treatment: “Wastewater treatment carbon emissions”, “Wastewater resource recovery”, and “Low-carbon wastewater treatment technologies”. Peer-reviewed articles on the above three topics published between 2000 and July 2025 were retrieved from major publishers, including Elsevier, Springer Nature, and ACS. We utilized a GPT-4.1 model to scan the titles and abstracts of the searched literature, leveraging the LLM comprehension capabilities to filter out articles that were irrelevant to the topic. The search terms are provided in Text S1. In parallel, we incorporated 11 engineering knowledge sources, including textbooks, engineering guidelines, standards, and technical handbooks, to better capture practical knowledge. All article PDFs and engineering documents were obtained and parsed by GROBID into JSON format containing only textual information.^27^ Based on these sources, a comprehensive domain-specific knowledge base comprising 7637 peer-reviewed articles and 11 engineering documents was constructed to provide a robust foundation for subsequent analyses. The detailed knowledge sources are provided in Tables S1–S3.

The long text of the original documents was segmented into smaller chunks to avoid diluting the meaning and weakening the retrieval quality. We used spaCy’s specialized scientific literature model (en_core_sci_md), due to its robust performance in processing scientific and technical text, to segment the original text into small chunks while ensuring sentence-level boundaries.^28^ Furthermore, to maintain semantic coherence between text chunks, we adopted a sentence-level overlapping chunking strategy.^29^ Specifically, when generating new text chunks, the system retained the last *N* complete sentences of the previous text chunk as the beginning part of the current chunk, where *N* was the preset number of overlapping sentences (in this study, *N* = 2). The maximum length of each chunk was limited to a fixed 256 tokens length. Chunks were formed by aggregating sentences until they approached a maximum token limit without truncating the final sentence. The citation information on each document, including authors, publication time, abstract, journal title, and DOI information, was used as metadata for the chunks.

### Overall Workflow of WaterRAG

The workflow of WaterRAG consisted of three principal stages: knowledge base construction, retrieval, and generation (Figure 1). In knowledge base construction, textual information was semantically encoded and indexed. Chunks were converted into standard Document objects through the LangChain framework while preserving the complete hierarchical metadata structure to ensure traceability of retrieval results and citation information.^30^ To enable retrieval by meaning, each chunk was embedded as a numeric vector in a semantic space where distance reflects the conceptual similarity. BAAI/bge-large-en-v1.5 pretrained embedding model (commit d4aa690) was used to generate 1024-dimensional dense vector representations.^31^ BAAI/bge-large-en-v1.5 has been trained on large-scale text corpora, which can effectively capture semantic information within the text. The generated vectors were L2-normalized and then used to construct efficient approximate nearest neighbor indices through the FAISS (Facebook AI Similarity Search) library, supporting rapid semantic similarity retrieval.^32^

**Figure 1:**
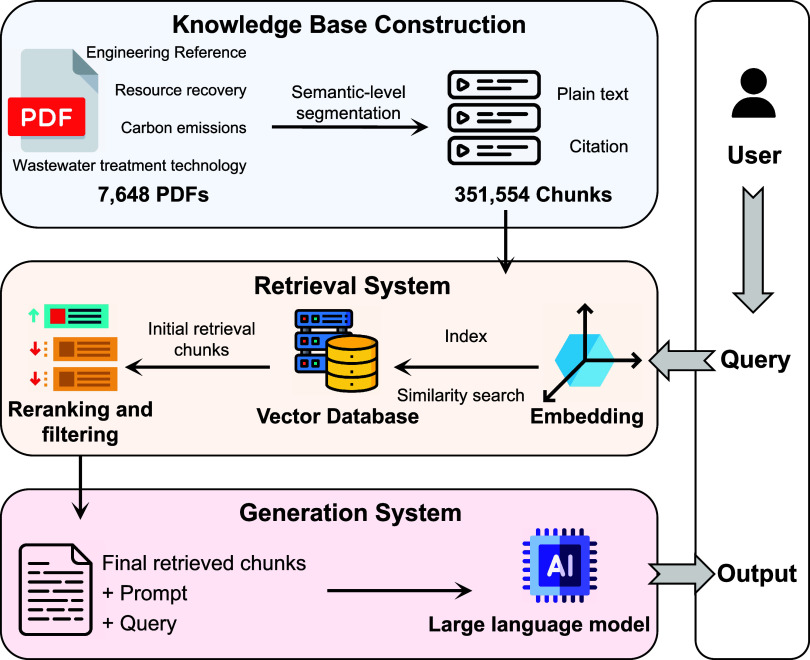
Overview of the WaterRAG knowledge base and RAG pipeline. Wastewater engineering documents are collected and semantically segmented into text chunks, embedded and indexed in a vector database, and retrieved through similarity search with reranking and filtering. The selected evidence is then provided to an LLM to generate grounded responses to user queries.

In the retrieval stage, when users posed questions, the system encoded the query into a vector using embedding models. It then searched the vector index based on similarity, returning the initial top k most relevant chunks. A reranker was applied to improve the retrieval quality. We compared the LLM-based reranker (GPT-4o) against three commercial rerankers (Voyage reranker-2.5, Jina reranker v3, and Qwen3 reranker). The LLM-based reranker achieved the best overall performance and was therefore adopted in this study, though Voyage reranker-2.5 offers competitive accuracy at lower latency and cost (Table S4). The impact of the number and length of retrieved chunks on QA answer correctness was evaluated (Figure S1). The optimal setting was determined to be 30 initial candidates with 10 retained after reranking. The complete reranking prompt is provided in Text S2.

Finally, in the generation stage, to ensure LLMs had complete contextual understanding, the final retrieved chunks from the same article were merged into a single document. Subsequently, the retrieved chunks, the user’s query, and the prompt instructing faithful responses based on the chunks were collectively submitted to an LLM serving as the answer generator. Finally, the LLM presented the generated answer and relevant cited references to the user. All models in this study were provided with a domain-relevant expert role prompt (see Text S3–S7 for the complete prompt).

For Q&A tasks requiring rapid response, we conducted a single retrieval and tested three standalone LLMs (without retrieval) as the answer generator: Llama-3.1–8B, GPT-4o, and GPT-4.1. For review and case study tasks involving large volumes of text processing, we employed a multiagent system to perform multiple retrievals, selecting GPT-4.1 for its superior contextual processing capabilities. In addition, we compared WaterRAG against Naive RAG and GPT 4.1 Search (GPT with Internet access) to assess potential improvements in retrieval quality and generation. The Naive RAG used a single-pass retrieval without WaterRAG’s text preprocessing or reranking, and directly fed the retrieved chunks into the generator. GPT 4.1 Search is a model with Internet retrieval capabilities that use the same prompts as Naive RAG.

### Multiagent Workflow

In complicated tasks of literature review and engineering case studies, advanced information processing and professional synthesis capabilities are needed. WaterRAG therefore integrates a multiagent system where role-specific LLM agents collaborate via iterative handoffs. This architecture provides more reliable support for wastewater-related tasks by enabling the systematic filling of evidence gaps and traceable synthesis. As shown in Figure 2, WaterRAG consists of three agent components: retrieval agent, review agent, and evaluation agent. These agents collaborate by performing their respective tasks and receiving information provided by other agents, and iterating continuously to update the review. Specifically, when WaterRAG initially receives a review task query, one GPT-4.1 within the retrieval agent extends it with domain-specific synonyms and related terms without altering the original intent, thereby improving the retrieval coverage. Similarity searches are then conducted using the expanded query to return relevant chunks. Subsequently, another GPT-4.1 reranks and filters relevant chunks, with scoring each chunk (relevance >6 out of 10) to determine usefulness before passing to the review agent. The review agent then generates an initial review based on relevant chunks and then passes the review on to the evaluation agent. The evaluation agent assesses the quality of the review by assigning a score, providing revision comments, and identifying missing information. Based on this identified missing information, the retrieval agent reexecutes the retrieval process and passes the newly retrieved chunks to the review agent. The review agent subsequently updates the current version of the review based on newly retrieved chunks and revised comments. This iterative process continues until the evaluation agent approves the review quality, with the stopping criterion defined as either achieving a relevance score greater than 9 or reaching a maximum of four iterations, whichever comes first, resulting in the final review output. All three agents use GPT-4.1 as the backbone model with different role configurations and task instructions for the retrieval, review, and evaluation stages. The complete prompt is presented in Text S5–S7.

**Figure 2:**
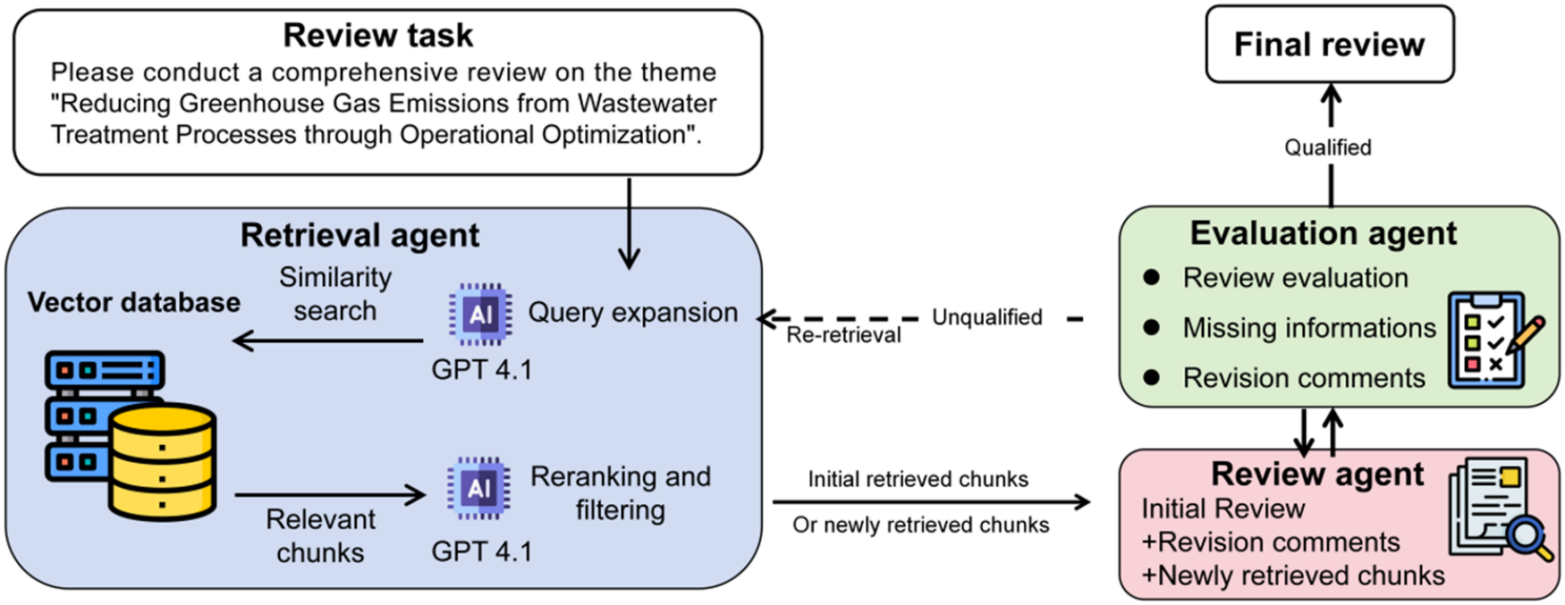
Illustration of the WaterRAG multiagent iterative review generation workflow. A retrieval agent performs query expansion, similarity search, and reranking to collect relevant evidence from the established vector database. A review agent synthesizes an initial draft based on retrieved chunks, while an evaluation agent assesses review quality and provides feedback to trigger further work cycles until a satisfied final review is generated.

To evaluate the contribution of the multiagent architecture of WaterRAG, we conducted ablation experiments using three progressively simplified variants: (1) a Two-Agent RAG (retrieval + review agents), (2) a Single-Agent RAG (one agent performing retrieval and review synthesis via a unified prompt), and (3) a 4-Step Naive RAG baseline, which generates responses sequentially by feeding retrieved documents in batches. Detailed settings are provided in Text S4.

### Evaluation of WaterRAG

This study constructed a total of 370 question–answer data sets (Q&As) with standard answers to evaluate the response quality of RAG and LLM systems on wastewater treatment problems. Among these, 138 literature-derived questions (90 multiple-choice and 48 open-ended) were generated by prompting GPT-4.1 (see Text S8 for the full prompt) on three topics of relevant literature (each containing 46 questions) and then manually screened by experts to remove items that were overly simplistic, ambiguous, or not representative of professional wastewater practice. Additionally, 232 engineering practice questions (114 multiple-choice and 118 open-ended) were compiled from existing exercise collections related to wastewater treatment operations. Detailed information about the exercise set sources can be found in Table S5. We employed LLM-as-a-Judge to evaluate both answer quality and retrieval quality.^33^ In this setup, an independent GPT-4.1 acts as an automatic reviewer, scoring each answer based on predefined criteria and the provided context. Specifically, two metrics were used to measure the answer quality: Answer Correctness and Answer Relevancy. For multiple-choice questions, correctness was determined by matching the chosen option with the ground truth, while for open-ended questions, it was judged by the LLM-as-a-Judge method with a 0.5 score threshold. We calculated the pass rate to evaluate the overall performance of the answers. The answer correctness pass rate was calculated as the ratio of the correct answers to the total number of questions. Answer relevancy pass rate was assessed by comparing answers (or explanations for multiple-choice) with the question using LLM-as-a-Judge, with a threshold of 0.9. Score of faithfulness, context precision, context recall, and context relevancy were used to evaluate the retrieval quality by incorporating retrieved chunks. Detailed information about the evaluation metrics is provided in Text S9. The LLM-as-a-Judge utilized the Deepeval framework to score these metrics.^34^ To validate the reliability of LLM-as-a-judge, a 10% random subset of the Q&As (GPT-4.1 as answer generator) was evaluated by both LLM and human experts. Cohen’s Kappa coefficient (calculated using sklearn in Python) of ≈ 0.8 indicates high consistency between assessments (Figure S2).

To evaluate the performance of LLM and RAG on review tasks, we generated 10 review tasks by referencing reviews previously published in renowned environmental journals. The specific inputs for each review task are detailed in Table S8. We excluded these referenced published reviews from the Knowledge Base to avoid artificially inflating performance through direct retrieval. Unlike QA, there is no single expected ground truth output for a review. Therefore, the quality of all generated reviews was cross-evaluated using both human expert assessment and LLM-as-a-Judge. Three evaluation metrics were established through custom prompt engineering in the Deepeval framework,^34^ each scored on a 0–10 scale, with a weighted composite score calculated for the total. The three evaluation metrics and their proportions were critical analysis (40%), professional depth (35%), and cutting edge (25%), which assessed the analytical depth and critical thinking capability of reviews, professional competence and academic rigor, and timeliness and forward-looking perspective. Expert evaluation was conducted by 4 professors in the wastewater treatment field through blind assessment, where experts were unaware of which system generated each answer. The complete evaluation criteria of review can be found in Text S11.

The energy consumption of model execution was estimated following the EPOCH AI methodology,^35^ with detailed procedures described in Text S12. Operational costs, token usage, energy consumption, and inference latencies for all models are summarized in Tables S9 and S10. In this work, statistical significance was assessed in Python using SciPy (scipy.stats), with McNemar’s tests for QA pass rates and paired *t* tests conducted for retrieval and review scores. All comparisons yielded *p* < 0.001, confirming significant differences (Tables S11–S13).

## Results and Discussion

### WaterRAG in Wastewater Treatment QA

We first evaluated the answer correctness and relevance of using three different LLMs as WaterRAG’s answer generator in wastewater treatment Q&As. As shown in Figure 3a, the answer relevance pass rate for all standalone LLMs and WaterRAG exceeds 90%. However, in the correct evaluation, WaterRAG significantly outperformed the standalone LLMs. Compared to the standalone baselines of Llama-3.1–8B (38.79%), GPT-4o (57.82%), and GPT-4.1 (64.91%), WaterRAG elevates the correctness pass rates to 64.12, 77.87, and 80.51%, respectively, when using these LLMs as answer generators. This demonstrates that while standalone LLMs possess excellent language comprehension and can produce semantically relevant responses, they still exhibit significant deficiencies in factual correctness. In contrast, by leveraging a tailored knowledge base, WaterRAG not only maintains comparable linguistic expression quality to standalone LLMs, but more importantly significantly improves the factual correctness of answers. Specifically, WaterRAG achieves more significant improvements for small-scale models compared to those for large-scale models. WaterRAG (64.12%) elevates the standalone Llama-3.1–8B (38.79%) correctness pass rate exceeding the standalone GPT-4o (57.82%) and approaching the standalone GPT-4.1 (64.91%). This indicates that WaterRAG can lift open-source, small-parameter models to a level of domain expertise comparable to that of commercial LLMs. For the wastewater industry, this offers a cost-effective and practical solution, while maintaining advanced wastewater knowledge. WaterRAG shows a smaller improvement over GPT-4o (20.05%) and GPT-4.1 (15.60%) compared with Llama-3.1–8B (25.33%). The relatively smaller improvement margin for GPT can be attributed to the larger scale. Also, large-scale models are exposed to more extensive pretraining data, which may already capture aspects of wastewater knowledge in open-source materials, thus leaving less room for enhancement.

**Figure 3:**
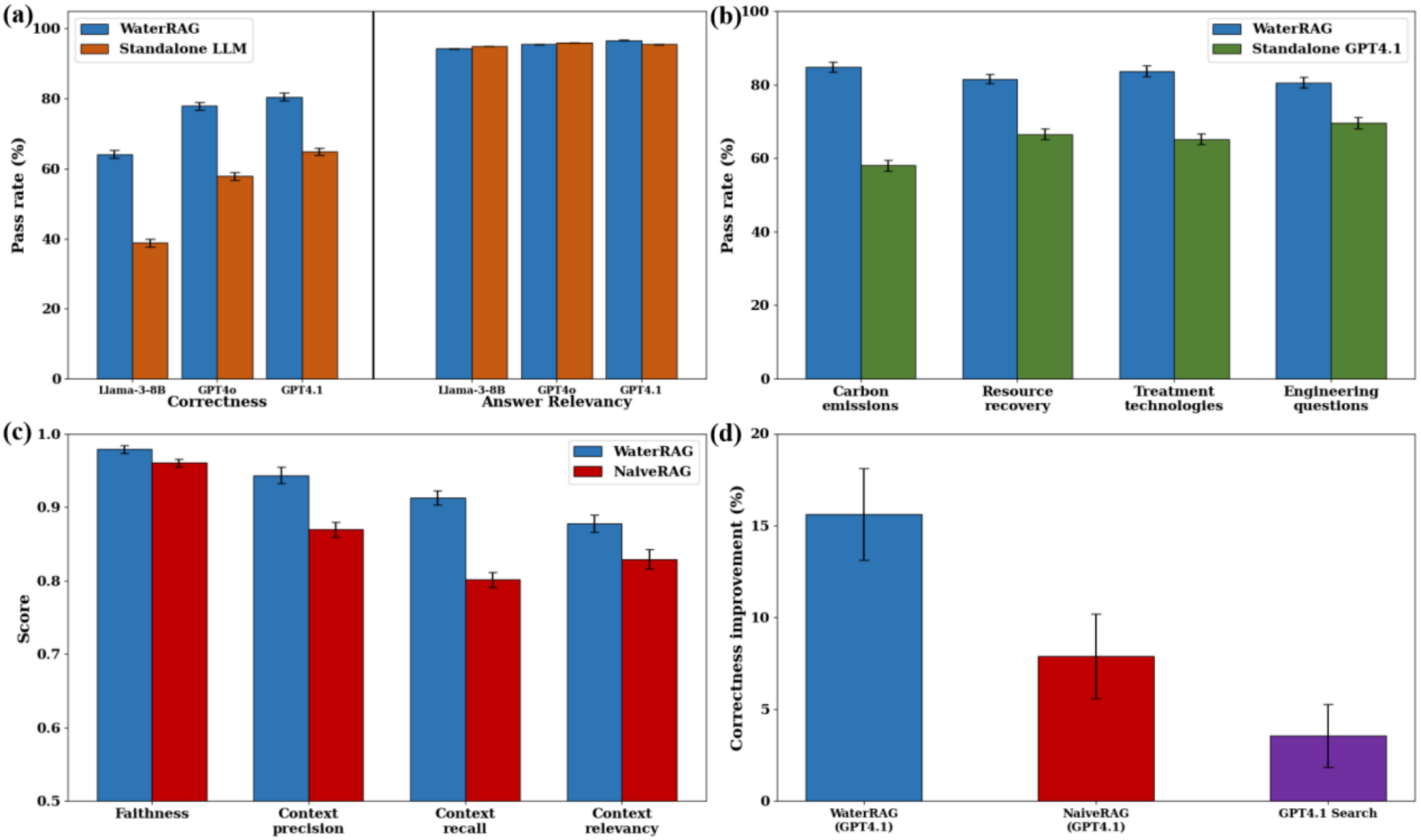
(a) Answer correctness and relevancy pass rate of WaterRAG and standalone LLMs on Q&A sets, (b) answer correctness pass rate of WaterRAG (GPT 4.1 as answer generator) and standalone GPT 4.1 in literature-derived questions and engineering practice questions, (c) score of retrieval metrics of WaterRAG and Naive RAG using GPT 4.1 as answer generator, (d) answer correctness pass rate improvement of WaterRAG, Naive RAG, and GPT-4.1 Search relative to standalone GPT-4.1.

The performance of standalone LLMs varies across different question types. As shown in Figure 3b, the standalone GPT-4.1 model achieved a lower correctness pass rate on literature-derived questions (63.25%) compared to engineering questions (69.61%), particularly on carbon emission questions where it only reached 58.01%. In contrast, WaterRAG achieved a correct pass rate of over 80% across all question types. This discrepancy may reflect fundamental differences in the model knowledge structures. The pretraining data of general-purpose LLMs likely has more extensive coverage of traditional wastewater engineering topics, whereas emerging areas such as carbon emissions accounting and net-zero wastewater treatment remain comparatively underrepresented. Tables S14 and S15 provide the complete Q&A pairs. For example, as shown in Question 1 in Figure 4, when asked about the relationship between N_2_O emissions from NH_2_OH oxidation pathways and the Ammonia Oxidation Rate (AOR), all standalone models incorrectly selected “Positively linearly” or “Negatively linearly”. However, a previous study has shown that linear relationships cannot fit this phenomenon.^36^ WaterRAG provided the correct answer (“Exponentially”) after synthesizing multiple literature sources.^5,36–38^ In Question 2, when asked whether anaerobic-anoxic-aerobic (AAO) or sequencing batch reactor (SBR) processes produce higher emissions, both Llama-3.1–8B and GPT-4o provided factually incorrect answers, stating AAO generates more emissions. GPT 4.1 indicates that SBR produces higher emissions but lacks detailed data support. WaterRAG not only explicitly points out that batch reactors have higher discharge volumes but also provides specific data to support this claim based on several recent papers:^4,39,40^ “*The total greenhouse gas (GHG) emissions from a SBR process can reach up to 1474 gCO*_2_ *-eq/m* ^3^, *whereas the highest emissions from an AAO process are about 437 gCO*_2_ *-eq/m* ^3^ *(Nguyen et al., 2019)*”.

**Figure 4:**
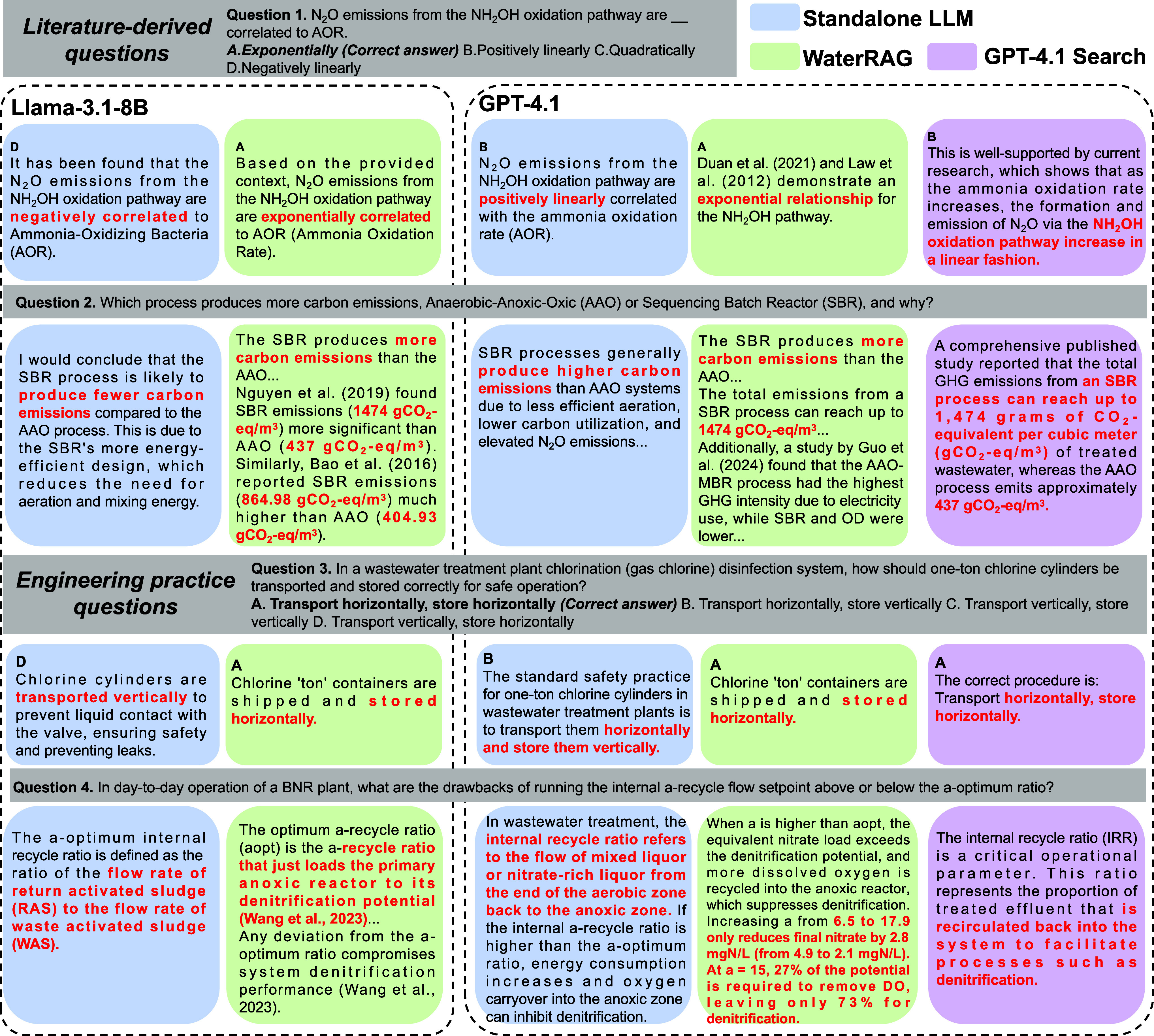
Four examples of Q&A tasks from WaterRAG, GPT 4.1 Search, and standalone LLMs. The red text highlights the key differences in their answers.

Errors in engineering operations are particularly critical because they can directly lead to safety risks and economic losses. WaterRAG can support operators’ decision-making by providing traceable, evidence-based knowledge. For example, in Question 3, when asked about safe operating procedures for chlorination in wastewater treatment, standalone LLMs consistently selected incorrect options contrary to safety regulations. In contrast, WaterRAG provided the correct answer based on operational manuals. In Question 4, when asked about the drawbacks of setting the internal aerobic-to-anoxic mixed liquor recycle (a-recycle) above or below its optimum ratio (aopt) in biological nutrient removal (BNR), Llama-3.1–8B incorrectly interpreted the optimum internal recycle ratio as the ratio of the return activated sludge (RAS) flow rate to the waste activated sludge (WAS) flow rate. GPT-4.1 generally recognized the context of a-recycle and a-opt within BNR systems, but its analysis remained largely qualitative. In contrast, WaterRAG not only provides mechanistic explanations but also offers verifiable quantitative evidence based on engineering documents, emphasizing diminishing marginal returns: “*Increasing a from 6.5 to 17.9 reduces final nitrate by only 2.8 mgN/L (from 4.9 mgN/L to 2.1 mgN/L)···When a = 15, 27% of the system’s potential capacity is consumed by dissolved oxygen removal, leaving only 73% available for denitrification*”.

We further analyzed the incorrect answers generated by WaterRAG (GPT-4.1 as generator) in the QA task and identified three principal causes of error (Table S6), with representative examples provided in Table S7 and a more detailed analysis in Text S10. Retrieval failure was the most common error type, accounting for 46% of all incorrect responses, followed by missing knowledge coverage (38%) and reasoning errors or hallucinations (16%). Together, retrieval failure and missing knowledge coverage comprised 84% of all errors, indicating that most failures arose from insufficient supporting evidence rather than from deficiencies in answer generation itself.

Retrieval failure primarily occurred when the initial retrieval stage did not return the most relevant answer-bearing documents. This was often associated with a semantic mismatch between the wording of the query and the language used in the source text. As shown in Table S7, for example, one query referred to “ancient practices used to recover resources from wastewater”, whereas the relevant answer-bearing chunk described the same concept indirectly using domain-specific expressions such as “human excreta as fertilizer” and “latrine-derived nutrients in 2000 BC”. In another case, the relevant information was embedded within a long paragraph, which further reduced its retrievability. These examples suggest that retrieval failures were mainly driven by short queries and the limited ability of general-purpose embeddings to capture specialized wastewater terminology and implicit semantic relationships.

In addition to retrieval failure, 38% of errors were attributed to missing knowledge coverage in the knowledge base. In these cases, no answer-bearing chunks were available in the knowledge base for retrieval, particularly for highly specific engineering practice questions, such as the threshold speed of a gravity belt press or the causes of chlorine vent line clogging (Table S7). By contrast, only a relatively small proportion of errors can be attributed to reasoning failures or hallucinations. These mainly occurred in calculation-based questions, such as unit conversion errors or a mismatch between the reasoning process and the final option selected (Table S7).

Cost and latency comparison indicates that, while WaterRAG introduces a slight additional latency in Q&A tasks (0.21–0.27 min beyond standalone LLMs), it maintains a minimal per-question cost of approximately $0.035–$0.044/task (Table S9).

### Retrieval in WaterRAG

To identify the factors contributing to WaterRAG’s superior performance, we benchmarked it against two baseline retrieval-enabled systems, both utilizing GPT-4.1 as the answer generator for the Q&A task: (1) a Naive RAG model without semantic segmentation and reranking, and (2) GPT-4.1 equipped with online search (without access to the curated domain knowledge base). As shown in Figure 3d, WaterRAG, Naive RAG, and GPT-4.1 Search improved over standalone GPT-4.1 by 15.6, 7.89, and 3.54%, respectively. WaterRAG achieved an answer correctness pass rate of 80.51%, compared with 72.80% for Naive RAG and 68.45% for the GPT-4.1 Search (Table S9). This indicates that, compared with standalone systems without retrieval (64.91%), both GPT-4.1 Search and the Naive RAG baseline benefit from the incorporation of retrieval capabilities. For example, as shown in Figure 4, GPT 4.1 Search can correctly answer water treatment safety operation questions (Q3) by retrieving documentation from open-source engineering materials. However, their performance remains limited in more advanced wastewater questions due to the use of nontailored knowledge base and weak retrieval quality. For example, regarding Q1, as shown in Table S14, GPT-4.1 Search selected the incorrect option, “Positively linearly”, based on two articles that did not directly address the question, and fabricated citation links. Although Naive RAG retrieved partially relevant documents, it incorrectly treated the N_2_O emission decrease caused by multipathway and DO-driven effects in the Partial Nitritation-SBR system observed by Lv et al.^41^ as an intrinsic relationship of the NH_2_OH oxidation pathway, and hence wrongly selected the option “Negatively linearly”. In contrast, WaterRAG provided the correct answer after synthesizing multiple article sources.^5,36–38^ These results indicate that WaterRAG’s effectiveness is attributed to the synergy between its optimized retrieval mechanism and its tailored knowledge base, which ensures reliability for wastewater treatment tasks.

Given that retrieval quality directly determines RAG performance, we further evaluated the retrieval metrics based on the chunks retrieved by WaterRAG and Naive RAG. As illustrated in Figure 3c, WaterRAG achieved significant improvements across all retrieval metrics except Faithfulness compared with Naive RAG. The consistency in Faithfulness scores demonstrated that the LLM-generated responses remained faithful to the retrieved chunks, while the improvements in all other metrics indicated that the enhancements were primarily attributed to retrieval quality rather than generation quality. In particular, compared with Naive RAG (0.80), WaterRAG (0.93) achieved the largest gain in context recall (16.25%), indicating that at equal retrieval counts, it captured more question critical information. These improvements can be attributed to the segmentation strategy and reranking mechanism used in WaterRAG. Table S16 presents the ablation results. Relative to fixed token-based segmentation, semantic segmentation increases the performance by 11.94%. Likewise, enabling reranking (vs no reranking) increases performance by 12.98%. These procedures ensure that WaterRAG can retrieve the most relevant chunks. Among the 138 literature-derived questions, WaterRAG achieved literature source consistency with standard answers for 120 questions, surpassing the 92 questions achieved by Naive RAG. Table S17 shows the retrieved chunks of the example question. The chunk containing the key answer in Question 1 ranks low (31st) in the Naive RAG due to incomplete semantic segmentation but ranks first in WaterRAG. In another question concerning N_2_O emissions outside biological reactors, Naive RAG predominantly retrieved chunks describing biological reactors. In contrast, WaterRAG filtered out such irrelevant content through reranking.

### Generation of Professional Wastewater Treatment Reviews

Reliable synthesis of rapidly expanding wastewater literature is as important as factual accuracy for utilities, policymakers, and researchers. For instance, utilities need structured evidence to evaluate emerging technologies such as mainstream Anammox and membrane-aerated biofilm reactors (MABR) when considering investments or process redesign. Policymakers require the transparent collection of real GHG emission data and operational data to support GHG inventories and net-zero roadmaps. WaterRAG addresses these needs by generating structured, citation-rich reviews that integrate relevant, up-to-date peer-reviewed articles, accelerating how knowledge is synthesized and applied in the water sector.

To evaluate the capability of generating literature reviews, WaterRAG was assigned 10 review tasks. LLM-as-a-Judge-based evaluation revealed performance rankings of WaterRAG (9.45 ± 0.13) > Naive RAG (8.51 ± 0.11) > GPT-4.1 (7.33 ± 0.20). Human expert evaluation demonstrated identical trends (Figure 5a) with mean scores of WaterRAG (7.77 ± 0.44) > Naive RAG (6.32 ± 0.31) > GPT-4.1 (5.17 ± 0.66). Among the three evaluation domains (Critical analysis, Professional depth, Cutting edge), WaterRAG demonstrated the most significant improvement in Professional depth, achieving mean score increases of 2.83 and 1.53 relative to GPT-4.1 and Naive RAG, respectively. For review generation, WaterRAG is more resource-intensive (13.65 min, USD 0.39) than simpler baselines (0.69 min-3.40 min, USD 0.008–0.016) (Table S10), but this is still reasonable considering substantially stronger literature grounding and higher expert-rated quality. Additionally, our fact-checking of WaterRAG revealed that all statements are substantiated by authentic literature with no fabricated information (details in Text S13 and Table S19).

**Figure 5:**
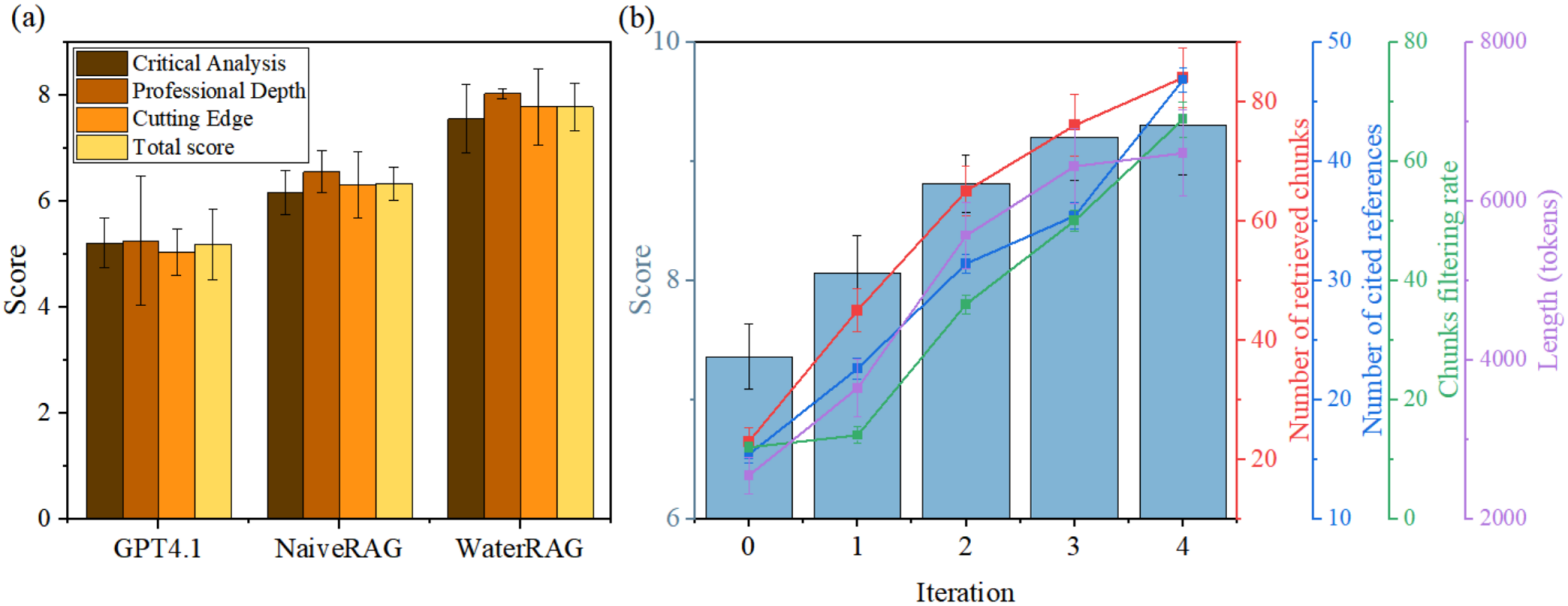
(a) Human expert evaluation of WaterRAG, Naive RAG, and GPT-4.1 on review tasks. (b) Evaluation of WaterRAG at each iteration.

Compared to GPT-4.1, WaterRAG was able to better integrate information from recent primary literature and propose more thoughtful arguments. Figure 6 shows the partial outputs of three different systems in an example review task (“Ammonia-oxidizing microorganisms (AOM) in wastewater and their N_2_O emissions”). In contrast to GPT-4.1’s conclusion that “*AOB are the dominant nitrifiers in most systems, especially under high-ammonia conditions, but AOA and comammox play important roles in specific niches”*, WaterRAG emphasized the rising trends of ecological niches and engineering roles of comammox and AOA in various wastewater treatment scenarios. WaterRAG summarized evidence from multiple studies reporting that the relative abundance of comammox could exceed that of AOB in some WWTPs, along with advantages over traditional AOB in achieving net-zero carbon emissions (see Table S18 for a complete review). In addition, when discussing the biological mechanisms of N_2_O production, WaterRAG provided specific emission factor comparisons along with biochemical pathway analyses, and explicitly stated in the conclusion that “*AOA and comammox typically lack typical NOR and emit far less than AOB*”. In contrast, GPT-4.1 did not provide such quantitative data support while incorrectly attributing the low N_2_O production rate of AOA to the lack of nirK/norB. This conflicted with recent studies revealing that AOA does not lack nirK but, rather, lacks NOR, which is the key factor for the low N_2_O production rates of AOA and comammox.^6,42,43^

**Figure 6:**
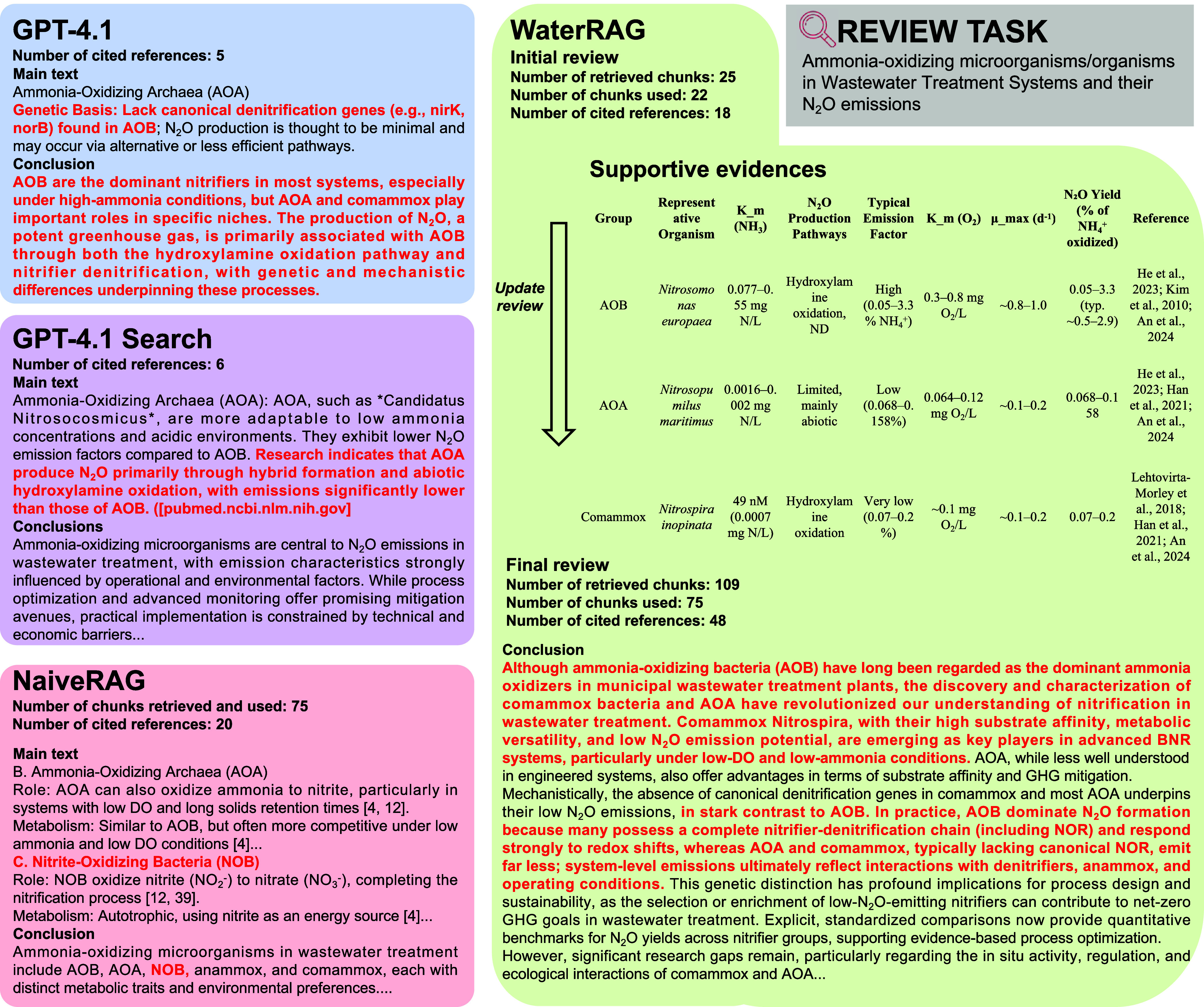
An example of professional review sections generated by WaterRAG, GPT, and Naive RAG in the review task of “Ammonia-oxidizing microorganisms and their N_2_O emission in wastewater”.

Compared to Q&A tasks, review generation required greater retrieval capabilities. Therefore, WaterRAG significantly outperformed Naive RAG and GPT-4.1 Search in retrieval performance. The average number of cited references in the 10 reviews generated by WaterRAG (46.8) was significantly higher than that of Naive RAG (23.6) and GPT 4.1 Search (7.1). This is due to the fact that WaterRAG’s retrieval agent maximized retrieval potential through extended querying, while also ensuring relevance through reranking and filtering. As shown in Figure 6, WaterRAG retrieved 109 chunks in total for this task, ultimately utilizing 75 highly relevant chunks for the review, generating and citing 48 references. Naive RAG, however, despite the query explicitly constraining the review to AOM in wastewater treatment, still retrieved 15 chunks describing non-AOM organisms (such as NOB, anammox, microalgae, etc.) alongside less relevant content. This resulted in only 20 cited references from Naive RAG in the example review task, with incorrect categorization of NOB and anammox under AOB in the conclusion. For GPT-4.1 Search, due to the lack of a specialized knowledge base, only 6 references were cited, with 3 of them retrieving only abstracts, thus lacking a comprehensive review. This demonstrates that WaterRAG’s knowledge base and retrieval mechanism can lead to more comprehensive and relevant retrieved information in the studied domain.

More importantly, WaterRAG can continuously improve review quality through its iterative three-agent refinement loop. To experimentally investigate the contribution of this architecture, we conducted systematic ablation experiments comparing WaterRAG against progressively simplified single-pass variants (Table S10). Single-Agent RAG (8.24), Two-Agent RAG (8.41), and four-step Naive RAG (8.10) achieved higher LLM-as-a-Judge review scores than WaterRAG’s initial draft (7.36), largely because they return more retrieved material in a single generation step. However, unlike WaterRAG, the simplified baselines lack an evaluation-guided agentic mechanism for iterative revision. Consequently, their performance remains below the WaterRAG final output, which reaches a substantially higher score of 9.34. As illustrated in Figure 5b, as the number of iterations increased, WaterRAG review score increased steadily, along with the number of cited references, response length, and the rate of filtering out irrelevant chunks. This indicates that WaterRAG’s advantages arise from the synergistic interplay of all components rather than any single module. In the WaterRAG architecture, three agents collaboratively enhance review quality. Specifically, the evaluation agent systematically identifies information gaps and provides targeted feedback to the retrieval module, enabling the review agent to incorporate updated evidence and thereby improve output quality. For example, as shown in Figure 6, compared to the initial review, the final review of WaterRAG updated key supportive evidence such as the N_2_O production pathways and emission levels of AOM during the iteration process, and formed a structured summary table. Additionally, the multicycle iterative mechanism enables WaterRAG to generate clearer structures. As shown in the review task example, the WaterRAG review structured the narrative as follows: “AOB ecological niche—Operations and Process Configurations Drive Nitrifier Selection—Quantitative Comparison of N_2_O—Mechanistic and Genetic Differences Leading to N_2_O Production”. By contrast, Naive RAG’s output appeared more fragmented, with sections typically comprising only one or two summary sentences and weaker connections between arguments and evidence.

In summary, WaterRAG combines robust retrieval with a self-refinement agentic loop to generate comprehensive, well-structured, and citation-rich reviews that outperform the baseline. By addressing missing evidence and avoiding information overload in a single pass, we provide clearer and more robust conclusions for wastewater management.

### Case Study of WaterRAG-Powered Wastewater Engineering Scientific Support

In the wastewater treatment industry, upgrades, retrofits, and troubleshooting of wastewater treatment plants often require engineering scientific support to provide recommendations and guidance, but this process is typically time-consuming and of high cost. Therefore, we aimed to combine the semantic understanding capabilities of LLMs with water treatment professional knowledge to provide faster, lower-cost, yet effective recommendations. Here we present a case study of using WaterRAG to provide comprehensive technical engineering solutions to a wastewater treatment plant (provided information on process configuration, influent and effluent quality data, sludge data, and energy and carbon emission data), toward achieving net-zero carbon emissions by 2030 (full prompt and responses are shown in Table S20).

Compared with a generic GPT, WaterRAG provided a source traceable report structured as “Potential Problem identification”, “Potential Root causes”, and “Solutions”. As shown in Figure 7, WaterRAG first reviewed the characteristics of the modified Ludzack-Ettinger (MLE) process together with the provided plant information and identified potential problems. It then reviewed each identified potential problem and, combined with available plant data, generated plausible contributing factors, supported by retrieved literature evidence. Finally, it proposed solution pathways for the identified potential problem. During this process, WaterRAG gained stronger support by blending advanced domain knowledge with site specifics.

**Figure 7:**
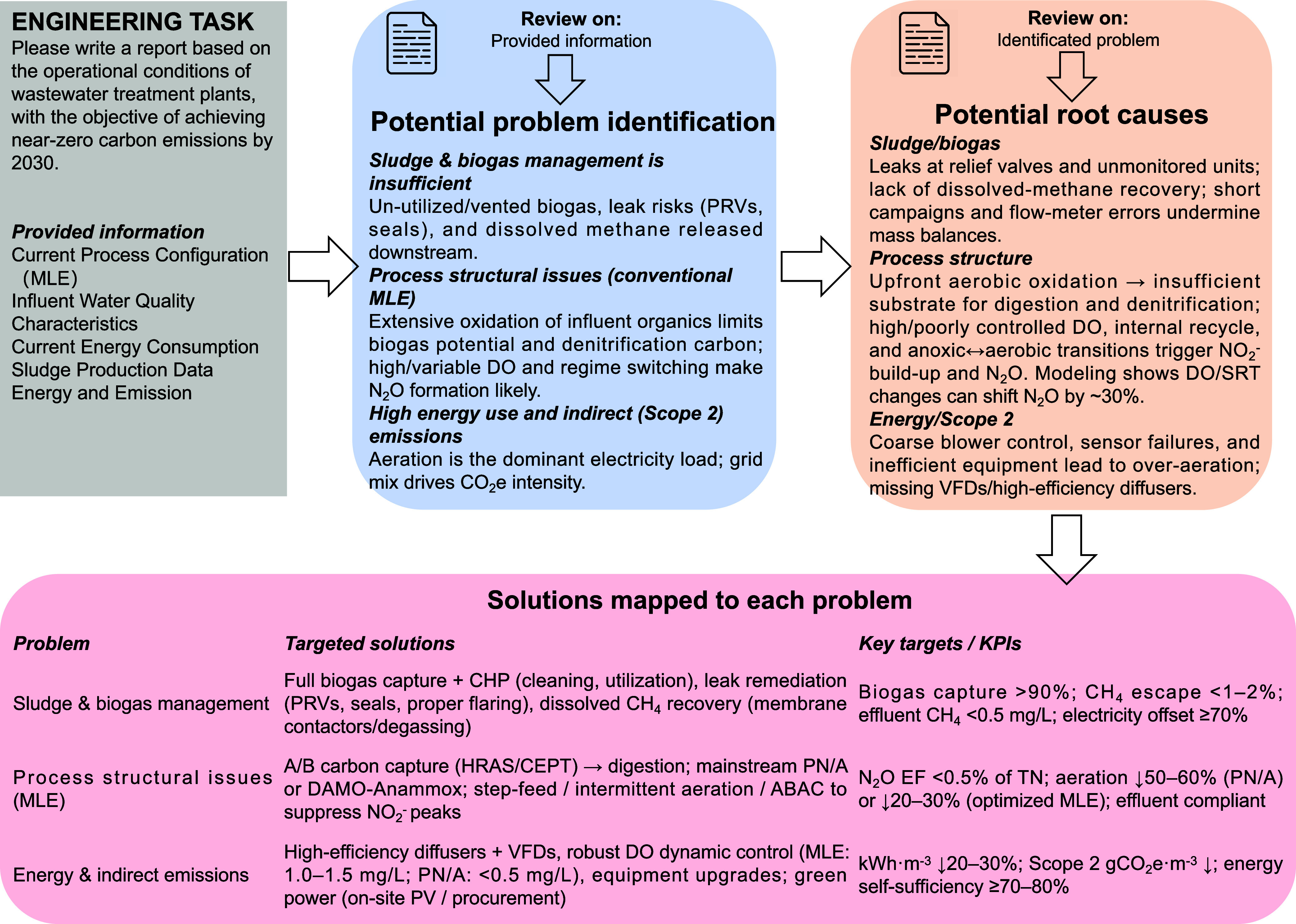
Summary of WaterRAG workflow and outputs in the engineering support case study.

The WaterRAG implementation roadmap follows a progressive five-stage path: baseline assessment, pilot-scale trials, modeling and decision support, phased implementation, and continuous monitoring and adaptive management. Specifically, WaterRAG recommends first compiling a current-state baseline focused on aeration electricity use and biogas handling, with targeted process-emissions monitoring for comparison. It then pilots high-impact actions (e.g., DO control optimization and biogas capture with combined heat and power (CHP)) at the unit or line scale before scaling them plant-wide in phases. Finally, it proposes continuous monitoring to verify outcomes and adjust operations. These align with the published net-zero roadmaps and core implementation logic. For instance, the IWA/WaCCliM Roadmap explicitly states that net-zero transition is a dynamic process requiring a closed-loop mechanism including assessing current status, identifying implementation actions, monitoring and verifying progress, and iterating cyclically.^44^ Additionally, WaterRAG’s proposed actions, such as switching to high-efficiency diffused aeration and deploying renewables via biogas capture with CHP units and on-site solar photovoltaic systems, align with the Water UK Net-zero routemap’s emphasis on improving energy efficiency by upgrading energy-intensive assets and deploying renewable energy generation.^45^

Drawing on the review literature, WaterRAG noted that in MLE systems, several factors, including low carbon-to-nitrogen ratios, high or fluctuating dissolved oxygen, anoxic to aerobic transitions, and adjustments in sludge age or external carbon, can evidently increase the N_2_O emission risk. Taking dissolved oxygen as an example, based on evidence from plant-level scenarios and multiobjective optimization studies regarding the relationship between aeration DO set points and GHG emissions, WaterRAG recommends setting dissolved oxygen within the range of 1.0–1.5 mg/L when optimizing MLE processes.^46–48^ Alternatively, conversion to partial nitrification/anammox processes should be pursued, maintaining dissolved oxygen below 0.5 mg/L. Notably, WaterRAG provides evidence-grounded decision support; however, this case study remains a proof-of-concept rather than a field deployment or operational validation. On-site verification, pilot testing, and operator assessment are still required before practical implementation. For example, based on prompt cues of biogas handling and literature linking pressure relief valves (PRV) to fugitive CH_4_, WaterRAG flagged potential PRV leakage as a plausible risk, which requires on-site inspection to confirm. In conclusion, WaterRAG showcased the capacity of integrating literature evidence with wastewater treatment plant data to form a traceable solution that supports zero-carbon pathways.

### Implications

Traditionally, practitioners in the wastewater treatment sector have acquired specialized expertise primarily through accumulated operational experience or by consulting external engineering experts, which can be time-consuming and costly. With the increasing availability of LLMs, consulting general LLM for rapid information gathering is becoming a common practice.^14,49^ However, these general-purpose LLMs still exhibit significant knowledge limitations in the highly specialized field of wastewater treatment, as demonstrated in recent studies^17,19^ and this work, e.g., in assessing process carbon emission variations and their driving factors (Figure 4).

As an early domain-tailored application of multiagent RAG to wastewater treatment, WaterRAG addresses this gap by integrating the linguistic and reasoning capabilities of LLMs and agent-based workflows with a curated, up-to-date wastewater knowledge base comprising 7637 peer-reviewed articles and 11 engineering references. WaterRAG provides a structured decision-support tool that improves evidence grounding beyond standalone general-purpose models. For example, WaterRAG achieves an 80.51% correctness pass rate in wastewater Q&A tasks, substantially outperforming standalone GPT-4.1 (64.91%). Notably, WaterRAG also delivers meaningful gains when paired with locally deployed open-source models (e.g., WaterRAG–Llama-3.1–8B: 64.12%), enabling more cost-effective and practical applications. Beyond Q&A tasks, WaterRAG can accelerate evidence synthesis for engineers and researchers by generating more comprehensive literature-grounded reviews. A comprehensive review of the latest developments in low-emission wastewater treatment can be developed within minutes rather than spending weeks on manual reviews, with expert evaluation scores of 7.77 and containing an average of 46.8 cited references. In addition, as shown in the case study, plant managers can obtain plant-specific engineering scientific support to inform decision-making. Importantly, WaterRAG is intended to complement rather than replace professional expertise. This tool substantially lowered the threshold for practitioners to access specialist knowledge, enabling even resource-constrained small wastewater treatment plants to benefit from technical support equivalent to that were only available to large-scale facilities.

Despite the promising capabilities, gaps still remain between WaterRAG and the reasoning capacity of experienced wastewater engineers. Expertise in wastewater treatment is not limited to reviewing the literature or documents but extends to conducting detailed analyses. For example, a competent wastewater engineer can quantitatively assess the impact of process upgrades on pollutant removal, energy demand, and operational costs by using various tools. At present, WaterRAG is only capable of evidence synthesis and lacks the quantitative, predictive, and evaluative capabilities that underpin true expert-level reasoning.

More broadly, both knowledge base quality and foundation model capability play critical roles in determining the accuracy and effectiveness of multiagent RAG deployment in wastewater applications. In particular, improvements in curated knowledge base and retrieval agentic workflows may represent a more accessible pathway for near-term progress than relying solely on advances in foundational models. To approach real expert-level capabilities, future systems should expand knowledge acquirement channels and integrate quantitative reasoning with real-world complex engineering problem evaluation. Strengthening domain knowledge structures and establishing closer interaction and iterations with practitioners will also be important to ensure that outputs remain both accurate and practical. In this way, AI frameworks such as WaterRAG can evolve from professional support tools into decision-support systems that complement expert judgment and help utilities progress toward carbon neutrality.

## Supplementary Material



## Data Availability

All code and evaluation processes involved in this study can be found on GitHub (https://github.com/Mudi12138/WaterRAG).

## Notes

Literature source search terms (Text S1), prompts of reranking and filtering (Text S2), prompts of question answering for WaterRAG, Naive RAG, GPT-4.1 with web search, and standalone LLM (Text S3), prompts of review and ablation setup of agent, including standalone LLM review prompts (Text S4), prompts of retrieval agent, including query expansion, reranking, and filtering (Text S5), prompts of review agent, including initial review generation and review refinement (Text S6), prompts of evaluation agent for review evaluation (Text S7), prompt for generating 138 literature-based questions (Text S8), LLM evaluator based on the DeepEval framework (Text S9), error analysis of incorrect answers from WaterRAG (GPT-4.1) in the QA task (Text S10), evaluation criteria of review (Text S11), energy consumption calculation (Text S12), and fact-checking (Text S13); the impact of chunk size and retrieval ratio on QA answer correctness pass rate (Figure S1) and consistency between LLM-as-a-judge and human expert evaluations in 10% of GPT-4.1’s QA tasks (Figure S2); volume of the literature data set (Table S1), research literature sources in the knowledge base (Table S2), sources of engineering knowledge (Table S3), performance of different rerankers in WaterRAG on Q&A tasks (Table S4), statistics on the number of Q&A (Table S5), breakdown of error types of WaterRAG in the QA task (Table S6), error examples of WaterRAG in the QA task (Table S7), 10 reference-based review tasks (Table S8), correctness and cost of QA tasks across different systems (Table S9), comparison of the evaluation of the review task by different systems (Table S10), QA correctness pass rate comparison using the McNemar test (Table S11), paired *t* test results of retrieval metrics (Table S12), paired *t* test comparison of expert review scores (Table S13), answer examples of multiple-choice questions from different systems (Table S14), answer examples of open-ended questions from different systems (Table S15), ablation evaluation of WaterRAG’s semantic segmentation and reranking (Table S16), example of retrieved document (Table S17), review examples of WaterRAG, naive RAG, and GPT-4.1 (Table S18), fact-checking of key arguments in the review task (Table S19), and case study of AI-powered wastewater engineering consultancy (Table S20); references (PDF)

## References

[1] Song C. H., Zhu J. J., Yuan Z. G., van Loosdrecht M. C. M., Ren Z. J. (2024). Defining and achieving net-zero emissions in the wastewater
sector. Nat. Water.

[2] Race to Zero, U. N. C. C. Net Zero Water Utilities: Global Industry Commitments 2022 https://www.water.org.uk/news-views-publications/news/global-water-industry-net-zero-commitments-top-72-million-peopleUnitedNations (accessed Jan 25, 2026).

[3] Xylem Inc . Net Zero Water Utility Roadmap: Practical Steps and Global Case Studies for Water Utilities to Achieve Net Zero Emissions; Xylem Inc., 2022 https://www.xylem.com/siteassets/campaigns/netzero/xylem-net-zero-paper-10.07.22-final.pdf. (accessed Jan 25, 2026).

[4] Song C., Zhu J.-J., Willis J. L., Moore D. P., Zondlo M. A., Ren Z. J. (2024). Oversimplification
and misestimation of nitrous oxide
emissions from wastewater treatment plants. Nat. Sustainability.

[5] Duan H., Zhao Y., Koch K., Wells G. F., Zheng M., Yuan Z., Ye L. (2021). Insights into Nitrous
Oxide Mitigation
Strategies in Wastewater Treatment and Challenges for Wider Implementation. Environ. Sci. Technol..

[6] Liu T., Duan H. R., Luecker S., Zheng M., Daims H., Yuan Z. G., Guo J. H. (2024). Sustainable
wastewater management
through nitrogen-cycling microorganisms. Nat.
Water.

[7] Lu L., Guest J. S., Peters C. A., Zhu X., Rau G. H., Ren Z. J. (2018). Wastewater treatment for carbon capture and utilization. Nat. Sustainability.

[8] Wu Z., Duan H., Li K., Ye L. (2022). A comprehensive carbon
footprint analysis of different wastewater treatment plant configurations. Environ. Res..

[9] Zheng M., Hu Z., Liu T., Sperandio M., Volcke E. I. P., Wang Z., Hao X., Duan H., Vlaeminck S. E., Xu K., Zuo Z., Guo J., Huang X., Daigger G. T., Verstraete W., van Loosdrecht M. C. M., Yuan Z. (2024). Pathways to advanced resource recovery
from sewage. Nat. Sustainability.

[10] Dessimoz C., Thomas P. D. (2024). AI and the democratization of knowledge. Sci. Data.

[11] Miret S., Krishnan N. M. A. (2025). Enabling large language models for real-world materials
discovery. Nat. Mach. Intell..

[12] Mon-Williams R., Li G., Long R., Du W., Lucas C. G. (2025). Embodied large language
models enable robots to complete complex tasks in unpredictable environments. Nat. Mach. Intell..

[13] Zhang Y., Han Y., Chen S., Yu R., Zhao X., Liu X., Zeng K., Yu M., Tian J., Zhu F., Yang X., Jin Y., Xu Y. (2025). Large language models
to accelerate organic chemistry synthesis. Nat.
Mach. Intell..

[14] Josh, A. ; Steven, A. ; Sandhini, A. ; Lama, A. ; Ilge, A. ; Florencia Leoni, A. ; Diogo, A. ; Janko, A. ; Sam, A. ; OpenAi . GPT-4 Technical Report arXiv 2023 10.48550/arXiv.2303.08774.

[15] Gemini, T. ; Rohan, A. ; Sebastian, B. ; Jean-Baptiste, A. ; Jiahui, Y. ; Radu, S. ; Johan, S. ; Andrew, M. D. ; Anja, H. ; Katie, M. Gemini: A Family of Highly Capable Multimodal Models arXiv 2023 arxiv.2312.11805.

[16] Yuntao, B. ; Saurav, K. ; Sandipan, K. ; Amanda, A. ; Jackson, K. ; Andy, J. ; Anna, C. ; Anna, G. ; Azalia, M. ; Cameron, M. ; Carol, C. ; Catherine, O. ; Christopher, O. ; Danny, H. ; Dawn, D. ; Deep, G. ; Dustin, L. ; Eli, T.-J. ; Ethan, P. ; Jamie, K. ; Jared, M. ; Jeffrey, L. ; Joshua, L. ; Kamal, N. ; Kamile, L. ; Liane, L. ; Michael, S. ; Nelson, E. ; Nicholas, S. ; Noemi, M. ; Nova, D. ; Robert, L. ; Robin, L. ; Sam, R. ; Scott, J. ; Shauna, K. ; Sheer El, S. ; Stanislav, F. ; Tamera, L. ; Timothy, T.-L. ; Tom, C. ; Tom, H. ; Tristan, H. ; Samuel, R. B. ; Zac, H.-D. ; Ben, M. ; Dario, A. ; Nicholas, J. ; Sam, M. ; Tom, B. ; Jared, K. Constitutional AI: Harmlessness from AI Feedback arXiv 10.48550/arXiv.2212.08073.

[17] Zhu J. J., Yang M. Q., Jiang J. Y., Bai Y. M., Chen D. Q., Ren Z. J. (2024). Enabling GPTs for Expert-Level Environmental
Engineering
Question Answering. Environ. Sci. Technol. Lett..

[18] Xu B., Wu G., Li Z., Xu G., Zeng H., Tong R., Ng H. Y. (2025). Towards
domain-adapted large language models for water and wastewater
management: methods, datasets and benchmarking. Npj Clean Water.

[19] Xu B., Li Z., Yang Y., Wu G., Wang C., Tang X., Li Y., Wu Z., Su Q., Shi X., Yang Y., Tong R., Wen L., Ng H. Y. (2025). Evaluating and Advancing
Large Language Models for Water Knowledge Tasks in Engineering and
Research. Environ. Sci. Technol. Lett..

[20] Vaswani, A. ; Shazeer, N. ; Parmar, N. ; Uszkoreit, J. ; Jones, L. ; Gomez, A. N. ; Kaiser, L. ; Polosukhin, I. Attention Is All You Need. In 31st Annual Conference on Neural Information Processing Systems (NIPS), Long Beach, CA, Dec 04–09, 2017; Neural Information Processing Systems (NIPS): LA Jolla, 2017; Vol. 30.

[21] Wu F., Shen T., Back T., Chen J., Huang G., Jin Y., Kuang K., Li M., Lu C., Miao J. (2025). Knowledge-Empowered,
Collaborative, and Co-Evolving AI Models: The
Post-LLM Roadmap. Engineering.

[22] Aditi, S. ; Abul, E. ; Saket, K. ; Tala Talaei, K. Agentic Retrieval-Augmented Generation: A Survey on Agentic RAG 2025 https://arxiv.org/abs/2501.09136 (accessed Jan 25, 2026).

[23] Şakar T., Emekci H. (2025). Maximizing
RAG efficiency: A comparative analysis of
RAG methods. Nat. Lang. Process..

[24] Ferber D., El Nahhas O. S. M., Woelflein G., Wiest I. C., Clusmann J., Lessmann M.-E., Foersch S., Lammert J., Tschochohei M., Jaeger D. (2025). Development and validation of an autonomous artificial
intelligence agent for clinical decision-making in oncology. Nat. Cancer.

[25] Qu Y., Huang K., Yin M., Zhan K., Liu D., Yin D., Cousins H. C., Johnson W. A., Wang X., Shah M. (2026). CRISPR-GPT for agentic automation of gene-editing experiments. Nat. Biomed. Eng..

[26] Wu K., Wu E., Wei K., Zhang A., Casasola A., Nguyen T., Riantawan S., Shi P., Ho D., Zou J. (2025). An automated
framework for assessing how well LLMs cite relevant medical references. Nat. Commun..

[27] GROBID . GitHub 2008 https://GitHub.com/kermitt2/grobid (accessed Jan 25, 2026).

[28] Honnibal, M. ; Montani, I. ; Van Landeghem, S. ; Boyd, A. spaCy: Industrial-Strength Natural Language Processing in Python Zenodo 2020 10.5281/zenodo.1212303.

[29] Eibich, M. ; Nagpal, S. ; Fred-Ojala, A. ARAGOG: Advanced RAG Output Grading. https://arxiv.org/abs/2404.01037submitted2024-04-01 (accessed Jan 25, 2026).

[30] LangChain . GitHub 2022 https://github.com/langchain-ai/langchain (accessed Jan 25, 2026).

[31] Shitao, X. ; Zheng, L. ; Peitian, Z. ; Niklas, M. ; Defu, L. ; Jian-Yun, N. C-Pack: Packed Resources For General Chinese Embeddings arXiv 10.48550/arXiv.2309.07597.

[32] Matthijs, D. ; Alexandr, G. ; Chengqi, D. ; Jeff, J. ; Gergely, S. ; Pierre-Emmanuel, M. ; Maria, L. ; Lucas, H. ; Hervé, J. Faiss library arXiv 10.48550/arXiv.2401.08281.

[33] Gu, J. ; Jiang, X. ; Shi, Z. ; Tan, H. ; Zhai, X. ; Xu, C. ; Li, W. ; Shen, Y. ; Ma, S. ; Liu, H. A Survey on LLM-as-a-Judge arXiv 2024 https://arxiv.org/abs/2411.15594v1 (accessed Jan 25, 2026). 10.1016/j.xinn.2025.101253 PMC13237853 42254963

[34] DeepEval . GitHub 2025 https://github.com/confident-ai/deepeval (accessed Jan 25, 2026).

[35] You, J. How much energy does ChatGPT use? Epoch AI Gradient Updates 2025 https://epoch.ai/gradient-updates/how-much-energy-does-chatgpt-use (accessed Jan 25, 2026).

[36] Ni B. J., Yuan Z. G., Chandran K., Vanrolleghem P. A., Murthy S. (2013). Evaluating four mathematical models
for nitrous oxide
production by autotrophic ammonia-oxidizing bacteria. Biotechnol. Bioeng..

[37] Law Y., Ni B.-J., Lant P., Yuan Z. (2012). N_2_O production
rate of an enriched ammonia-oxidising bacteria culture exponentially
correlates to its ammonia oxidation rate. Water
Res..

[38] Ribera-Guardia A., Pijuan M. (2017). Distinctive NO and N_2_O
emission patterns
in ammonia oxidizing bacteria: Effect of ammonia oxidation rate, DO
and pH. Chem. Eng. J..

[39] Nguyen T. K. L., Huu Hao N., Guo W., Chang S. W., Dinh
Duc N., Long Duc N., Liu Y., Ni B., Hai F. I. (2019). Insight
into greenhouse gases emissions from the two popular treatment technologies
in municipal wastewater treatment processes. Sci. Total Environ..

[40] Bao Z., Sun S., Sun D. (2016). Assessment of greenhouse gas emission from A/O and
SBR wastewater treatment plants in Beijing, China. Int. Biodeterior. Biodegrad..

[41] Lv Y. T., Ju K., Sun T., Wang L., Miao R., Liu T. T., Wang X. D. (2016). Effect
of the dissolved oxygen concentration on the
N2O emission from an autotrophic partial nitritation reactor treating
high-ammonium wastewater. Int. Biodeterior.
Biodegrad..

[42] Ran X., Wang T., Zhou M., Li Z., Wang H., Tsybekmitova G. T., Guo J., Wang Y. (2025). A Novel Perspective
on the Instability of Mainstream Partial Nitrification: The Niche
Differentiation of Nitrifying Guilds. Environ.
Sci. Technol..

[43] Kozlowski J. A., Kits K. D., Stein L. Y. (2016). Comparison of Nitrogen Oxide Metabolism
among Diverse Ammonia-Oxidizing Bacteria. Front.
Microbiol..

[44] Ballard, S. ; Porro, J. ; Trommsdorff, C. The Roadmap to a Low-Carbon Urban Water Utility: An International Guide to the WaCCliM Approach; IWA Publishing, 2018 10.2166/9781780409924.

[45] Water, U. K. Net Zero 2030 Routemap 2020 https://www.Water.org.uk/news-views-publications/publications/net-zero-2030-routemapWaterUK (accessed Jan 25, 2026).

[46] Lackner S., Gilbert E. M., Vlaeminck S. E., Joss A., Horn H., van Loosdrecht M. C. M. (2014). Full-scale
partial nitritation/anammox experiences
- An application survey. Water Res..

[47] Flores-Alsina X., Amell M., Arnerlinck Y., Corominas L., Gernaey K. V., Guo L., Lindblom E., Nopens I., Porro J., Shaw A. (2014). Balancing
effluent quality,
economic cost and greenhouse gas emissions during the evaluation of
(plant-wide) control/operational strategies in WWTPs. Sci. Total Environ..

[48] Du R., Li C., Liu Q., Fan J., Peng Y. (2022). A review of enhanced
municipal wastewater treatment through energy savings and carbon recovery
to reduce discharge and CO_2_ footprint. Bioresour. Technol..

[49] Lee B. C., Chung J. Y. (2024). An empirical investigation
of the impact of ChatGPT
on creativity. Nat. Hum. Behav..

