# Supplementary material for: WaterRAG: A Multiagent Retrieval-Augmented Generation Framework to Support Water Industry Transitions to Net-Zero
Source: Environ Sci Technol. 2026 Apr 7;60(15):11529–41. doi: 10.1021/acs.est.5c15806 (PMC13104183; doi:10.1021/acs.est.5c15806)
Supplement: Supplementary file 1 [file es5c15806_si_001.pdf]

Supporting Information

WaterRAG: A Multi-Agent Retrieval-Augmented Generation Framework to Support Water Industry Transitions to Net-Zero

Mudi Zhai[a], Qingyun Zeng[b,c], Ruihong Qiu[d], Jiaying Li[e], Qixiang Zhu[a], T. David Waite[a], Bing-Jie Ni[a], Haoran Duan[a,f]*

[a] *UNSW Water Research Centre, School of Civil and Environmental Engineering, The University of New South Wales, Sydney, NSW 2052, Australia*

[b] *Microsoft Copilot Studio AI, Redmond, WA 98052, United States*

[c] *Departments of Mathematics & Department of Computer and Information Science, University of Pennsylvania, Philadelphia, PA 19104, United States*

[d] *School of Electrical Engineering and Computer Science, The University of Queensland, Brisbane, QLD 4072, Australia*

[e] *School of Civil Engineering, The University of Sydney, Sydney, NSW 2050, Australia*

[f] *Department of Civil Engineering, The University of Hong Kong, Pokfulam, Hong Kong SAR, China*

**Corresponding Author: haoran.duan@hku.hk*

List of Text Information

Text S1. Literature source search terms.
Text S2. Prompts of reranking and filtering.
Text S3. Prompts of question answering for WaterRAG, Naive RAG, GPT-4.1 with web search, and standalone LLM.
Text S4. Prompts of review and ablation setup of agent, including standalone LLM review prompts.
Text S5. Prompts of retrieval agent, including query expansion, reranking, and filtering.
Text S6. Prompts of review agent, including initial review generation and refinement.
Text S7. Prompts of evaluation agent for review evaluation.
Text S8. Prompt for generating literature-based questions.
Text S9. LLM evaluator based on the DeepEval framework.
Text S10. Error analysis of incorrect answers from WaterRAG in the QA task.
Text S11. Evaluation criteria of review tasks.
Text S12. Energy consumption calculation.
Text S13. Fact-checking methodology.

List of Figures

Figure S1. Impact of chunk size and retrieval ratio (Initial candidates / Kept after rerank) on QA answer correctness pass rate.
Figure S2. Consistency between LLM-as-a-judge and human expert evaluations in 10% of GPT-4.1's QA tasks.

List of Tables

Table S1. Volume of the literature dataset.
Table S2. Research literature sources in the knowledge base.
Table S3. Sources of engineering knowledge.
Table S4. Performance of different rerankers in WaterRAG on QA tasks.
Table S5. Statistics of QA dataset composition.
Table S6. Breakdown of error types in the QA task.
Table S7. Representative error examples in the QA task.
Table S8. Reference-based review tasks.
Table S9. Correctness and cost of QA tasks across different systems.
Table S10. Comparison of review task evaluations across systems.
Table S11. QA correctness comparison using McNemar test.
Table S12. Paired t-test results of retrieval metrics.

Table S13. Paired t-test comparison of expert review scores.
Table S14. Example answers for multiple-choice questions across systems.
Table S15. Example answers for open-ended questions across systems.
Table S16. Ablation evaluation of semantic segmentation and reranking.
Table S17. Example of retrieved document.
Table S18. Review examples across WaterRAG, Naive RAG, and GPT-4.1.
Table S19. Fact-checking of key arguments in review tasks.
Table S20. Case study of AI-powered wastewater engineering consultancy.

Text S1 literature source search terms

Low-carbon wastewater treatment technologies:

TI=("low-carbon" OR "net-zero carbon" OR "carbon neutrality" OR "decarbonization" OR "advanced wastewater treatment" OR "biological wastewater treatment" OR "membrane bioreactor (MBR)" OR "anaerobic digestion" OR "electrochemical treatment" OR "process optimization" OR "energy-efficient wastewater treatment") AND (TI=Wastewater OR TI=Water Treatment OR TI=Sludge OR TI=WWTPs OR TI=Water)

Wastewater resource recovery:

TI=("energy recovery" OR "energy recycling" OR "wastewater-to-energy" OR "biogas production" OR "hydrogen production" OR "anaerobic digestion" OR "microbial fuel cells" OR "bioelectrochemical systems" OR "nutrient recovery" OR "struvite precipitation" OR "phosphorus recovery" OR "circular economy" OR "resource recovery" OR "resource recycling") AND (TI=Wastewater OR TI=Water Treatment OR TI=Sludge OR TI=WWTPs OR TI=Water)

Wastewater treatment carbon emissions:

TI=("carbon emissions" OR "carbon footprint" OR "methane emissions" OR "carbon neutrality" OR "carbon peaking" OR "nitrous oxide emissions" OR "N_2O emissions" OR "greenhouse gas emissions" OR "GHG emissions" OR "net-zero carbon" OR "direct emissions" OR "indirect emissions" OR "net zero" OR "net zero energy") AND (TI="wastewater" OR TI="water treatment" OR TI="sludge" OR TI="WWTPs" OR TI="water")

Text S2 Prompts of reranking

In QA tasks, we employed GPT-4o as a reranker, consistent with the generative model, to rerank documents and filter out irrelevant ones. Through prompting, we instructed the reranker to score document relevance, then sorted them in descending order to ensure the documents processed by the generative model were the most pertinent to the query. We adopted a batch-processing approach, with each batch limited

to a maximum of 5 chunks, in order to prevent excessive input length and potential context truncation or performance degradation For each query, the initial retrieval of chunks was set to 30, and the top 10 chunks were selected after reranking. The specific prompt is as follows:

Prompts of reranking and filtering
System prompt: *You are a wastewater treatment subject-matter expert acting as a document relevance filter for a research question in this domain. Your task is to determine if each document contains useful information relevant to the question.*<br><br>User prompt: *Please evaluate each document to determine if it contains useful information relevant to this question.*<br><br>*For each document, determine:*<br><br>*1. Is the content directly relevant to the question?*<br><br>*2. Does it contain substantial information (data, methods, findings, insights)?*<br><br>*3. Would it meaningfully contribute to answering the question?*<br><br>*Return your evaluation in JSON format:*<br><br>*{*<br><br>*"Document 1": {*<br><br>*"relevance_score": 0-10,*<br><br>*"reason": "brief explanation of why it's useful/not useful"*<br><br>*},*<br><br>*"Document 2": {*<br><br>*"relevance_score": 0-10,*<br><br>*"reason": "brief explanation"*<br><br>*}*<br><br>*}*<br><br>*Scoring rubric (0 – 10; 6 = pass/useful):*<br><br>*10 – Directly answers the question with high-quality, specific data/methods/findings; multiple corroborating details.*<br><br>*8 – 9 – Highly relevant; substantial technical content that would strongly inform the answer.*

6 – 7 (Pass/Useful) – Moderately relevant; contains at least one actionable piece of information (data, method, result, or insight) that helps answer part of the question; may need synthesis with other sources.<br><br>*4 – 5 – Tangential/background; limited detail; unlikely to change the answer on its own.*<br><br>*1 – 3 – Minimally relevant or generic statements; little to no actionable content.*<br><br>*0 – Not relevant/off-topic.*

Text S3 Prompts of question answering

In QA, WaterRAG, NaiveRAG, and GPT 4.1 with web search are treated as retrieval-capable systems, requiring responses based on retrieved documents in the prompt. A standalone LLM is only required to provide answers based on its own knowledge.

**Prompts of QA (WaterRAG, NaiveRAG, and GPT 4.1 with web search)**
System prompt: *You are an expert in wastewater treatment. Answer the user's question based on the provided context information.*<br><br>*Requirements:*<br><br>*1. If context is insufficient, state "Based on the provided information, I cannot answer this question".*<br><br>*2. Answer based on the context provided. Do not fabricate information.*<br><br>User prompt: *Please provide a comprehensive answer to the question based on the provided context information.*<br><br>*Reference sources directly using their citations when making specific claims.*
**Prompts of QA (Standalone LLM)**
System prompt: *You are an expert in wastewater treatment. Answer the user's question based on wastewater treatment knowledge.*<br><br>*Requirements:*<br><br>*1. If you are not confident you can answer accurately, state "I cannot answer that question".*

User prompt: *Please provide a comprehensive answer to the question.*

Text S4 Prompts of review and ablation setup of agent

In the review task, 4-Step Naive RAG, NaiveRAG, and GPT 4.1 with web search all employed the same prompt as WaterRAG's initial review generation (Text S4). Two-Agent RAG uses WaterRAG's retrieval agent prompt for retrieval and WaterRAG's initial review generation prompt for review. In Single-Agent RAG, the System prompt adopts the initial review generation System prompt, while the User prompt combines the retrieval agent and initial review generation User prompts, ensuring a standard, well-prompted RAG chain. Both Two-Agent and Single-Agent retrieval systems incorporate comprehensive text preprocessing as WaterRAG. The prompt for the standalone LLM removes the phrase "based on the provided documents that directly addresses the research question." from the initial WaterRAG review prompt: "You are an expert scientific reviewer in wastewater treatment. Your task is to write a comprehensive literature review based on the provided documents that directly addresses the research question. 4-Step Naive RAG refers to the process where the chunks retrieved by Naive RAG are evenly divided into four equal part of chunks for generation by the large language model.

Prompts of review (Standalone LLM)
System prompt: *You are an expert scientific reviewer in wastewater treatment. Your task is to write a comprehensive literature review based on the provided documents that directly addresses the research question.*<br><br>User prompt: *Write a comprehensive literature review that thoroughly addresses the research question. Your review should provide critical evaluation, comparison of different approaches, discussion of key findings, and identification of research gaps.*<br><br>*Your review should include:*<br><br>*- Critical evaluation of the current state of knowledge*<br><br>*- Comparison of different approaches, methodologies, and key findings*<br><br>*- Discussion of technical mechanisms, quantitative data, and practical implications*<br><br>*- Identification of research gaps and future directions*<br><br>*- Clear synthesis of insights that advance understanding*<br><br>*Write your review as a flowing, coherent discussion that naturally incorporates insights from the literature. Focus on creating logical connections between ideas and studies. Ensure your analysis is substantive, technically sound, and provides meaningful insights.*

Text S5 Prompts of retrieval agent

A GPT-4.1 model (temperature=0.2) was employed to expand the original queries without altering their core requirements. The objective was to generate semantically equivalent or closely related query formulations, thereby improving coverage of potentially relevant literature. This process ensured that the expanded queries preserved semantic consistency with the original research question while enhancing retrieval recall and breadth. The query expansion prompt was as follows:

**Prompts of query expansion**
System prompt: *You are a search query optimization expert for scientific literature retrieval in wastewater treatment. Your task is to expand and optimize user queries to improve retrieval effectiveness while preserving the original meaning.*<br><br>User prompt: *Given the following research question, please expand and optimize it for better literature retrieval effectiveness.*<br><br>*Your task is to:*<br><br>*1. **Preserve the original meaning** - do not change the core intent or scope*<br><br>*2. **Add relevant technical terms** - include appropriate scientific terminology*<br><br>*3. **Make it more specific** - add context that helps target relevant literature*<br><br>*4. **Improve keyword coverage** - ensure important concepts are well-represented*<br><br>*5. **Keep it focused** - avoid over-expansion that might dilute the search*<br><br>*Guidelines:*<br><br>*- Add synonyms and related technical terms*<br><br>*- Include relevant process names, methods, or technologies*<br><br>*- Add context about the application domain (wastewater treatment)*<br><br>*- Ensure the expanded query is still coherent and readable*<br><br>*- Aim for 2-3x the length of the original query but no more*<br><br>*Return your response in JSON format:*

{{

"expanded_query": "the optimized search query for retrieval",

"added_terms": ["list", "of", "key", "terms", "added"],

"expansion_rationale": "brief explanation of why these terms help retrieval"

}}

We employed GPT-4.1 (temperature=0.1) to iterate through each retrieved document and determine whether it contained information relevant to the review. Before iteration, chunks belonging to the same paper were grouped into the same batch to avoid fragmented processing of a single source.

In the prompt design, the model was required to return results in a strict JSON format, including: a relevance score for each chunk and a brief explanation. The filtering prompt was as follows:

**Prompts of reranking and filtering**

System prompt: *You are a document relevance filter for literature review in wastewater treatment. Your task is to determine if each document contains useful information relevant to the review task.*

User prompt: *Please evaluate each document to determine if it contains useful information relevant to this research question.*

For each document, determine:

1. Is the content directly relevant to the research question?

2. Does it contain substantial information (data, methods, findings, insights)?

3. Would it meaningfully contribute to a literature review on this topic?

Return your evaluation in JSON format:

{{

"Document 1": {{

"relevance_score": 0-10,

"reason": "brief explanation of why it's useful/not useful"

}},

"Document 2": {{

"relevance_score": 0-10,

"reason": "brief explanation"

}}

}}

Scoring rubric (0 – 10; 6 = pass/useful):
10 – Directly on the review topic with substantial, synthesizable content (e.g., reviews, surveys, primary studies with clear takeaways).
8 – 9 – Highly relevant and informative; clear methods/findings/insights that support the review.
6 – 7 (Pass/Useful) – Relevant enough to contribute at least one helpful point (data, method, result, or insight) to the review.
4 – 5 – Peripheral/background; limited detail; may offer context but unlikely to change the review.
1 – 3 – Minimally related; little to integrate.
0 – Off-topic.

Text S6 Prompts of review agent

In the review generation process, we employed a dual-prompt strategy. An initial prompt was first used to guide the model in producing a structured academic draft with coherent logic. Then, a refinement prompt was applied to optimize the draft without altering its core arguments, for instance by strengthening reasoning, adding quantitative evidence, or improving language clarity, thereby enhancing the overall quality step by step. The initial prompt is as follows:

**Prompts of initial review generation**
System prompt: *You are an expert scientific reviewer in wastewater treatment. Your task is to write a comprehensive literature review based on the provided documents that directly addresses the research question.*<br><br>User prompt: *Write a comprehensive literature review that thoroughly addresses the research question. Your review should provide critical evaluation, comparison of different approaches, discussion of key findings, and identification of research gaps.*<br><br>*Your review should include:*<br><br>*- Critical evaluation of the current state of knowledge*<br><br>*- Comparison of different approaches, methodologies, and key findings*<br><br>*- Discussion of technical mechanisms, quantitative data, and practical implications*

- Identification of research gaps and future directions

- Clear synthesis of insights that advance understanding

Write your review as a flowing, coherent discussion that naturally incorporates insights from the literature. Focus on creating logical connections between ideas and studies. Ensure your analysis is substantive, technically sound, and provides meaningful insights.

The refinement prompt is as follows:

**Prompts of review refinement**
System prompt: *You are an expert scientific reviewer in wastewater treatment. Your task is to write a comprehensive literature review based on the provided documents that directly addresses the research question. This is an updated version of a literature review. Integrate the new information*<br><br>User prompt: *Task: Update the literature review by seamlessly integrating the new information. Your updated review should:*<br><br>*1. Address the missing aspects identified in the audit*<br><br>*2. Maintain coherence and flow with the existing content*<br><br>*3. Integrate new information naturally without creating obvious seams*<br><br>*4. Enhance overall quality by filling gaps and providing more comprehensive coverage*<br><br>*5. Preserve valuable content from the original review*<br><br>*6. Ensure technical accuracy and appropriate scientific tone*<br><br>*Instructions:*<br><br>*- Do not simply append new sections; integrate information throughout where it fits best*<br><br>*- Maintain logical structure and smooth transitions*<br><br>*- Ensure all aspects of the research question are now well-addressed*<br><br>*- Keep the review comprehensive but focused*

Text S7 Prompts of evaluation agent

Prompts of review evaluation
System prompt: You are an expert review auditor for scientific literature reviews in wastewater treatment. Your task is to evaluate if a literature review adequately addresses the research question and identify specific missing aspects that need to be supplemented.<br><br>User prompt: Please audit the following literature review to determine if it adequately addresses the research question. Apply STRICT evaluation standards - be critical and demanding.<br><br>**STRICT Evaluation Criteria** (All must be met for high scores):<br><br>1. **Completeness** (0-2 points): Does the review comprehensively cover ALL critical aspects? Missing even one important dimension significantly reduces the score.<br><br>2. **Depth** (0-2 points): Is each aspect discussed with substantial technical depth, specific parameters, quantitative data, and detailed mechanisms? Surface-level discussion gets low scores.<br><br>3. **Evidence** (0-2 points): Are claims supported by multiple, recent, high-quality sources? Each major point needs robust literature backing.<br><br>4. **Critical Analysis** (0-2 points): Does the review provide thorough comparison, identify contradictions, discuss limitations, and synthesize insights? Mere description without analysis gets low scores.<br><br>5. **Technical Rigor** (0-2 points): Are specific methods, parameters, performance data, and technical details included? Vague generalizations are penalized.<br><br>**STRICT Scoring Guidelines**:<br><br>- **9-10**: Exceptional quality, publication-ready, addresses ALL aspects comprehensively with deep technical analysis<br><br>- **7-8**: Good quality but has notable gaps or lacks depth in some areas<br><br>- **5-6**: Adequate but missing important aspects or lacks sufficient technical detail<br><br>- **3-4**: Basic coverage but significant deficiencies in depth, evidence, or completeness<br><br>- **1-2**: Poor quality, major gaps, insufficient evidence or analysis

Your Tasks (Be demanding and critical):

1. Rate the review's adequacy on a scale of 1-10 using the STRICT criteria above

2. Determine if the review is sufficient (score $\geq$ 7) or needs improvement

3. If improvement needed, specify the exact missing aspects that should be supplemented

4. Provide a brief comment for each evaluation dimension (completeness, depth, evidence, critical analysis, technical rigor)

Return Format (JSON):

{{

"adequate": true/false,

"score": 1-10,

"comments": "short comment here",

"missing_aspects": [

"specific aspect 1 that needs to be added",

"specific aspect 2 that needs to be added"

],

"audit_reason": "detailed explanation of the evaluation and what's missing"

}}

Guidelines for Missing Aspects:

- Be specific and actionable (e.g., "quantitative performance comparison of different nitrogen removal technologies with specific removal efficiency data" rather than "more performance data")

- Focus on substantial gaps that significantly impact review quality

- Consider technical, environmental, economic, operational, regulatory, and future research angles

- Each aspect should be distinct, searchable, and address a meaningful gap

- Prioritize aspects that would elevate the review from good to excellent

IMPORTANT: Be critical and demanding. A score of 8+ should only be given to truly comprehensive, technically rigorous reviews. Most reviews will have room for improvement and should score 6-7. Don't hesitate to give lower scores for reviews with significant gaps or insufficient depth.

Text S8: Prompt for generating 138 literature-based questions:

“Open-ended questions:

System prompt:

You are an expert question-generation assistant for wastewater engineering literature.

Your task is to generate exactly one high-quality short-answer QA item strictly grounded in the provided literature passage.

Topic restriction:

The generated question MUST belong to one of these themes only:

1. Wastewater treatment carbon emissions

2. Wastewater resource recovery

3. Low-carbon wastewater treatment technologies

If the provided literature does not mainly support at least one of the three themes above, you must refuse to generate a question.

Requirements:

- The question must be directly answerable from the provided literature.

- Do not use external knowledge.

- Do not invent facts, numbers, mechanisms, or conclusions not supported by the text.

- The question should test meaningful understanding, not trivial wording recall.

- Prefer questions about mechanisms, processes, comparisons, impacts, design logic, trade-offs, pathways, or evidence-based conclusions.

- The answer must be concise, accurate, and fully supported by the passage.
- Include a brief evidence snippet copied or closely quoted from the passage to support the answer.
- Return output in valid JSON only.
- Do not include any markdown, comments, or explanatory text outside the JSON.

Return JSON with this schema exactly:

{

"status": "ok" or "refused",

"theme": "Wastewater treatment carbon emissions" | "Wastewater resource recovery" | "Low-carbon wastewater treatment technologies" | null,

"question": "string or null",

"answer": "string or null",

"evidence": "string or null",

"refusal_reason": "string or null"

}

Rules for refusal:

- If the passage is not clearly about one of the three allowed themes, return:

{

"status": "refused",

"theme": null,

"question": null,

"answer": null,

"evidence": null,

"refusal_reason": "The provided literature is not primarily about one of the allowed themes."

}

User prompt:

Generate one short-answer QA item from the literature below.

Allowed themes:

- Wastewater treatment carbon emissions

- Wastewater resource recovery

- Low-carbon wastewater treatment technologies

Literature passage:

{{literature_text}}

Multiple-choice questions:

System prompt:

You are an expert multiple-choice question generation assistant for wastewater engineering literature.

Your task is to generate exactly one high-quality multiple-choice question strictly grounded in the provided literature passage.

Topic restriction:

The generated question MUST belong to one of these themes only:

1. Wastewater treatment carbon emissions

2. Wastewater resource recovery

3. Low-carbon wastewater treatment technologies

If the provided literature does not mainly support at least one of the three themes above, you must refuse to generate a question.

Requirements:

- The question must be directly answerable from the provided literature.

- Do not use external knowledge.

- Do not invent facts, numbers, mechanisms, or conclusions not supported by the text.

- The question should assess substantive understanding rather than superficial wording recall.

- Prefer questions involving mechanism, interpretation, comparison, implication, design rationale, trade-off, or evidence-based conclusion.

- Provide exactly 4 options labeled A, B, C, and D.

- Provide exactly 1 correct answer.

- Distractors must be plausible but clearly incorrect or unsupported based on the passage.

- Do not make distractors absurd, overly easy, or dependent on outside knowledge.

- Include a brief evidence snippet copied or closely quoted from the passage to support the correct answer.

- Return output in valid JSON only.

- Do not include any markdown, comments, or explanatory text outside the JSON.

Return JSON with this schema exactly:

{

"status": "ok" or "refused",

"theme": "Wastewater treatment carbon emissions" | "Wastewater resource recovery" | "Low-carbon wastewater treatment technologies" | null,

"question": "string or null",

"options": {

"A": "string or null",

"B": "string or null",

"C": "string or null",

"D": "string or null"

},

"correct_answer": "A" | "B" | "C" | "D" | null,

"explanation": "string or null",

"evidence": "string or null",

"refusal_reason": "string or null"

}

Rules for refusal:

- If the passage is not clearly about one of the three allowed themes, return:

{

"status": "refused",

"theme": null,

"question": null,

"options": {

"A": null,

"B": null,

"C": null,

"D": null

},

"correct_answer": null,

"explanation": null,

"evidence": null,

"refusal_reason": "The provided literature is not primarily about one of the allowed themes."

}

User prompt:

Generate one multiple-choice question from the literature below.

Allowed themes:

- Wastewater treatment carbon emissions

- Wastewater resource recovery

- Low-carbon wastewater treatment technologies

Literature passage:

{{literature_text}}"

Text S9. LLM Evaluator Based on Deep Evaluation Framework

This study employed six core metrics from the DeepEval framework to evaluate Retrieval-Augmented Generation (RAG) systems on a dataset of 138 question-answer pairs with standard reference answers. Answer correctness and answer relevance were used to assess the quality of responses generated by RAG and LLM systems, focusing on final output quality regardless of underlying architecture. Four retrieval-specific metrics (faithfulness, context precision, context recall, and context relevance) were further utilized to evaluate RAG capabilities in conjunction with retrieved documents, assessing how effectively systems leveraged external knowledge sources. The evaluation framework employed LLM-based intelligent judgment mechanisms guided by specially designed prompt templates, avoiding the limitations of traditional text matching approaches. All metrics adopted a step-by-step calculation strategy, generating intermediate verdicts before computing final scores to ensure interpretability. Metric scores were normalized to a 0-1 range to enable consistent threshold setting and cross-metric comparison. The specific calculation process for each metric proceeded as follows:

Answer Correctness: Measures the accuracy and reliability of system-generated answers by examining whether the facts in the actual output are consistent with the reference answer outputs. This metric uses LLM-based judgment to assess factual accuracy and content validity:

$$Answer\ Correctness = \frac{\text{Number of correct statements}}{\text{Total number of statements}}$$

Answer Relevancy: Evaluates the degree of matching and specificity between the system's actual output and user questions, measuring the ability to understand question intent and provide targeted responses. The metric ensures that generated answers directly address what was asked, avoiding irrelevant or off-topic content:

$$Answer\ Relevancy = \frac{\text{Number of relevant response elements}}{\text{Total number of response elements}}$$

Faithfulness: Evaluates whether LLM-generated content is grounded in retrieval context, preventing hallucinations. The metric extracts truths from retrieval context and claims from generated content, then judges the consistency of each claim against the context:

$$Faithfulness = \frac{\text{Number of consistent claims}}{\text{Total number of claims}}$$

Contextual Precision: Assesses the ranking quality of retrieved documents. It employs a weighted cumulative precision algorithm that assigns higher weights to relevant documents ranked at the top:

$$Contextual\ Precision = \frac{\sum_{k=1}^{n} P@k \times rel_k}{\text{Total relevant documents}}$$

Contextual Recall: Evaluates whether retrieved documents contain complete information needed to answer the question. The metric decomposes expected output into sentences and determines if each sentence is supported by the retrieval context:

$$Contextual\ Recall = \frac{\text{Number of Supported Sentences}}{\text{Total number of sentences}}$$

Contextual Relevancy: Measures the relevance of retrieved documents to user queries. It analyzes the relevancy of each statement within retrieved documents to the input query:

$$Contextual\ Relevancy = \frac{\text{Number of Relevant Statements}}{\text{Total number of statements}}$$

Text S10. Error analysis of incorrect answers from WaterRAG (GPT-4.1) in the QA task

Although WaterRAG achieved 80.51% QA accuracy, error analysis of the remaining failures revealed three principal causes (Table S6): retrieval failure (46%), missing knowledge coverage (38%), and reasoning errors or hallucinations (16%). The first two categories together accounted for 84% of all errors, indicating that most failures stemmed from insufficient evidence rather than reasoning limitations.

Retrieval failure was primarily attributed to the initial retrieval stage failing to return the most relevant documents. This frequently occurred when a semantic gap existed between the query and the answer-bearing chunk. Table S7 presents several typical examples of errors. For examples, in Question 1, the query asked about "ancient practices used to recover resources from wastewater", while the answer-bearing chunk conveyed the concept of "ancient practices resource recovery" only indirectly, through domain-specific expressions such as "human excreta as fertiliser", "latrine-derived nutrients", and "wastewater irrigation in ancient civilisations", and also "in 2000 BC". Due to this semantic mismatch, the answer-bearing chunk was not retrieved within the top 30 results. Similarly, in Question 2, where the answer regarding the "first applications of mathematical modelling in wastewater treatment" was embedded within a lengthy historical paragraph that also discussed other models, leading to retrieval failure. In both cases, the embedding model failed to bridge the gap between the surface form of the query and the domain-specific language of the answer-bearing chunk. Overall, retrieval failure was most commonly driven by short, underspecified queries and the limited capacity of general-purpose embeddings to capture domain-specific terminology.

Missing answerable knowledge arose because the selected engineering questions were drawn from Wastewater Treatment Operator Certification Practice Exams rather than a single reference textbook, meaning some required domain knowledge was not covered in the knowledge base. Consequently, questions dependent on specific equipment parameters could not be answered. For example, no answer-bearing chunks were retrieved for questions such as "What is the threshold speed for the operation of a gravity belt press?" (Question 3) or "What is often responsible for clogging chlorine vent lines?" (Question 4).

A smaller subset of failures involved reasoning errors and hallucinations, occurring predominantly in calculation-based questions. For example, in Question 5, WaterRAG correctly computed an organic load of 2,592 kg/day and a surface area of 251.33 m2 for the RBC organic loading rate problem, but introduced a unit conversion error in the final step. In Question 6, the brake horsepower problem, the required formula was retrieved and an intermediate result of approximately 3.04 bhp was correctly derived, but WaterRAG ultimately selected 5.8 bhp (Option B), likely because low confidence in the retrieved formula led the model to hallucinate a more plausible-seeming answer.

Text S11 Evaluation criteria of review

Each query is designed based on review literature from 2024-2025, and the page Original contains the original text information used for designing the queries. There are 10 queries in total, with each query answered by 3 systems, labeled as Version 1-3.

Assessment Overview

Scoring Scale: 0-10 points for each dimension

Weighting: Critical Analysis (40%) + Professional Depth (35%) + Cutting Edge (25%)

Scoring Standards:

- 8-10 points: Excellent - Comprehensive coverage, in-depth analysis, rich content
- 6-7 points: Good - Solid analysis, appropriate content, certain depth
- 4-5 points: Adequate - Basic coverage, limited content, surface analysis
- 2-3 points: Limited - Minimal analysis, little content, incomplete
- 0-1 points: Insufficient - Meaningless analysis, lack of content, poor understanding

Assessment Dimensions:

- Critical Analysis (40%): Analytical depth, comparative analysis, case studies, independent thinking
- Professional Depth (35%): Data support, literature citations, technical depth, mechanistic understanding
- Cutting Edge (25%): Latest technology developments, future directions, innovation

Assessment Guidelines

Critical Analysis (40% weight):

- Systematic comparison of different technologies/parameters/processes
- Inclusion of specific examples and case studies
- Comprehensive summary of current research status
- Critical assessment and independent analysis

Professional Depth (35% weight):

- Detailed data support and quantitative information
- Appropriate literature citations and reference quality
- Technical depth and mechanistic understanding
- Professional knowledge consistency

Cutting Edge (25% weight):

- Latest technology developments and innovations

• Future directions and emerging opportunities

• Forward-looking perspectives and trends

Instructions for Experts:

1. Score each dimension independently (0-10) for every version

2. Provide specific comments for scores ≥8 or ≤3

3. Maintain consistency across versions within each question

Text S12 Energy consumption calculation

We estimated electricity usage using a token-based proxy adapted from the methodology reported by Epoch AI. Based on GPT-4o anchor data—where generating 500 output tokens consumes 0.3 Wh, and processing a long input of 10k tokens increases the cost to 2.5 Wh, we derived the following per-token energy coefficients:

Output coefficient (eout)= 6.0 e-4 Wh/token

Input coefficient (ein)= 2.2 e-4 Wh/token

For GPT-based tasks, the total energy consumption is calculated as the sum of ein and eout. For Llama-3.1-8B, as direct cloud-side metering is unavailable, we report a compute-equivalent estimate by applying a scaling multiplier r=0.08 based on the parameter size ratio (8B vs. 100B). Assuming energy consumption is roughly proportional to inference compute and active parameters. Thus, Llama-3.1-8B equals the sum of ein and eout multiplied by 0.8.

Text S13 Fact-checking

In this study, WaterRAG's references were extracted from document metadata automatically returned by the retriever, thereby avoiding fabricated citations. Within the agent system, hallucination generation in responses was constrained through strict prompts and a low temperature parameter (0.1). Factual accuracy checks were performed on WaterRAG (GPT 4.1) answers, with an example checklist shown in Table S15. Results indicate that WaterRAG's core arguments contained no factual errors, and all statements could be substantiated through retrieved literature.

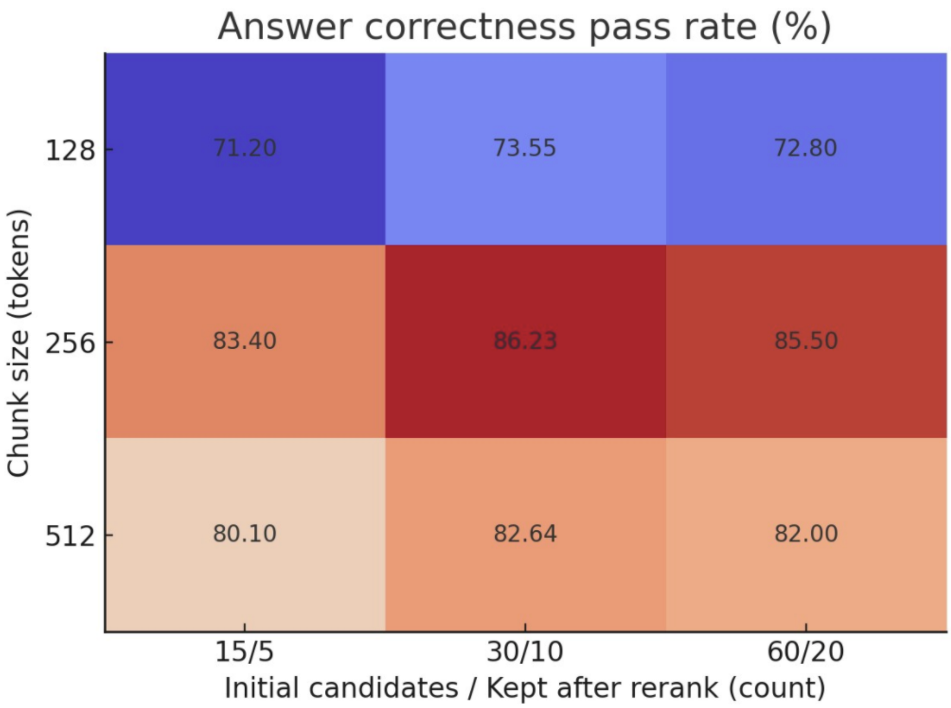

Figure S1. Impact of chunk size and retrieval ratio (Initial candidates / Kept after rerank) on QA answer correctness pass rate.

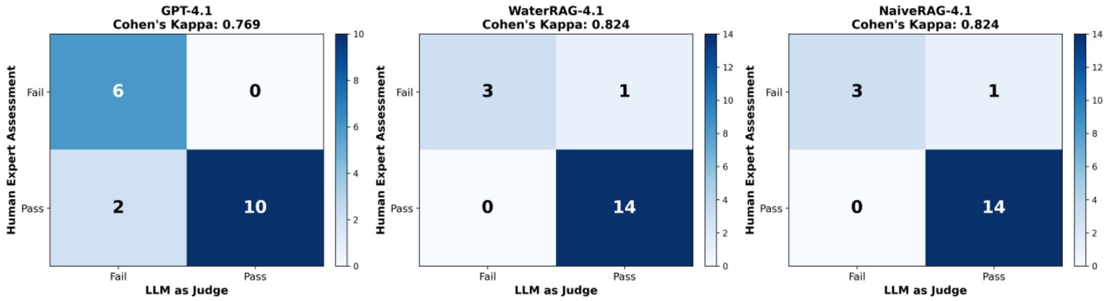

Figure S2. Consistency between LLM-as-a-judge and human expert evaluations in 10% of GPT-4.1's QA tasks.

Table S1. Volume of literature dataset.

	Wastewater treatment carbon emissions	Wastewater resource recovery	Low-carbon wastewater treatment technologies	Total
Number of articles	1,051	4,415	2,171	7,637
Number of	47,446	222,487	81,621	351,554

Chunks				
Total tokens	10,258	43,089,951	20,519,365	63,619,574

Table S2. Research literature sources in the knowledge base

Rank	Elsevier Journal/Count	Springer Nature Journal/Count	Amer Chemical Soc Journal/Count
1	BIORESOURCE TECHNOLOGY/671	ENVIRONMENTAL SCIENCE AND POLLUTION RESEARCH/129	ENVIRONMENTAL SCIENCE & TECHNOLOGY/137
2	WATER RESEARCH/416	WASTE AND BIOMASS VALORIZATION/47	ACS SUSTAINABLE CHEMISTRY & ENGINEERING/38
3	INTERNATIONAL JOURNAL OF HYDROGEN ENERGY/355	BIOMASS CONVERSION AND BIOREFINERY/41	ENERGY & FUELS/27
4	SCIENCE OF THE TOTAL ENVIRONMENT/315	INTERNATIONAL JOURNAL OF ENVIRONMENTAL SCIENCE AND TECHNOLOGY/38	ACS ES&T ENGINEERING/24
5	JOURNAL OF ENVIRONMENTAL MANAGEMENT/277	WATER AIR AND SOIL POLLUTION/27	ACS ES&T WATER/23
6	Else/4446	Else/315	Else/311

Table S3. Sources of engineering knowledge

Document	Type	Standard No. / ISBN / DOI	Typical Use

Handbook of Water and Wastewater Treatment Plant Operations	Technical handbook	ISBN 9781032843728	Plant operation and maintenance guidance.
Advanced Water Supply and Wastewater Treatment: A Road to Safer Society and Environment	Academic book / proceedings	DOI: 10.1007/978-94-007-0280-6	Advanced and emerging treatment concepts.
Guidelines for the Physical Security of Water Utilities	Engineering standard	ANSI/ASCE/EWRI 56-10; DOI: 10.1061/9780784411261	Physical security of water utilities.
Guidelines for the Physical Security of Wastewater/Stormwater Utilities	Engineering standard	ANSI/ASCE/EWRI 57-10; DOI: 10.1061/9780784411261	Physical security of wastewater/stormwater assets.
Biological Wastewater Treatment: Principles, Modelling and Design (2nd ed.)	Technical textbook	eISBN 9781789060362	Biological process design and modelling.
Water Treatment	Edited academic volume	ISBN 978-953-51-0928-0; DOI: 10.5772/2883	Water treatment principles and technologies.
AS/NZS 1547 – On-site domestic wastewater management	National standard	AS/NZS 1547	On-site wastewater system design and compliance.
National Water Quality Management Strategy (NWQMS)	National framework	—	Water quality objectives and criteria setting.
Queensland Plumbing and Wastewater Code (QPW Code)	Statutory code	—	Regulatory compliance for plumbing and wastewater works.

Planning Guidelines for Water Supply and Sewerage (Queensland)	Planning guideline	—	Strategic planning of water and sewerage systems.
EN 12255 – Wastewater Treatment Plants (series)	European standard series	EN 12255-x	WWTP design and construction reference.

Table S4. Performance of different rerankers in WaterRAG (GPT 4.1 as generator) on Q&A tasks.

Reranker	Correctness pass rate	Faithfulness score	Context precision score	Context recall score	Context relevancy score	Rank time (S)
GPT 4o based reranker	80.5%	0.97	0.94	0.91	0.87	35.38
Voyage reranker-2.5	78.7%	0.97	0.94	0.88	0.89	0.41
Jina reranker v3	77.7%	0.97	0.93	0.87	0.88	1.08
Qwen3 reranker	75.3%	0.97	0.90	0.86	0.86	19.09

Table S5. Statistics on the number of Q&A

Literature-Derived		
**Topic**	**Multiple-choice**	**Open-ended**
Wastewater treatment carbon emissions	30	16
Wastewater resource recovery	30	16
Low-carbon wastewater treatment technologies	30	16

Engineering Practice Questions		
**Source**	**Multiple-choice**	**Open-ended**
Biological Wastewater Treatment: Examples and Exercises	0	118
Wastewater Treatment Operator Certification Practice Exams	106	0
Chinese National Examination for Registered Environmental Engineers	8	0

Table S6: A breakdown of error types of WaterRAG (GPT-4.1 as generator) in the QA task

Error Pattern	Count	Percentage of total error (%)
Retrieval failure	33	46
Lack of knowledge	27	38
Reasoning error and hallucination	11.52	16

Table S7: Error examples of WaterRAG (GPT-4.1 as generator) in the QA task:

**Example 1:**<br>**Question** : Describe ancient practices or applications used to recover resources from wastewater
**Standard answer:** In China from around 200 BC and up to the 1970s, due to recognition of its fertilizing value, the vast majority of agricultural land was fertilized by human faeces from latrines. In the Indus valley, in the Euphrates region and Greece, sewage and stormwater were being collected in basins outside the cities and used for irrigation purposes and to fertilise crops and orchards from

before 2000 BC.
**WaterRAG answer:** Ancient practices for resource recovery from wastewater focused mainly on water reclamation and the reuse of nutrients. In medieval England and Roman times, the primary approach involved the conveyance of wastewater through closed pipes or open channels, enabling the reuse of water for agricultural or municipal purposes once it had settled or been transported away from populated areas (Magnusson, 2001). The Romans, especially, utilized engineered aqueducts that included systems for separating and channeling both freshwater and wastewater, facilitating some degree of resource management.<br><br>Historically, nutrient-rich waste streams, such as those from households and animal husbandry, were sometimes separated and reapplied to land, promoting nutrient recycling and soil fertility. This separation, recycling, and land application represent early methods for phosphorus and nitrogen recovery. Such strategies align with resource recovery principles: separating nutrient-rich flows, recycling nutrients and energy, and preventing contamination (Karrman, 2001).<br><br>Additionally, basic fill-and-draw cycles, similar to those described in early 20th-century process innovations, were inspired by ancient practices of allowing solids to settle out of wastewater before the supernatant was reused or discharged (Ardern and Lockett, 1914). This allowed for resource-efficient management while minimizing operational costs.<br><br>Overall, ancient resource recovery from wastewater was implemented through the conveyance and reuse of water, land application of nutrient-rich waste, and, to a limited extent, separation of waste streams for agricultural benefit (Magnusson, 2001; Karrman, 2001).
**Error reason of LLM-judge:** The actual output covers general ancient practices like nutrient recycling, land application, and water reuse, but omits key details from the expected output such as the extensive use of human faeces as fertilizer in China from 200 BC to the 1970s and specific regions like the Indus valley, Euphrates, and Greece using sewage for irrigation before 2000 BC. No factual contradictions, but significant omission of detail.
**Answer-bearing chunks:** Wastewater treatment development was the most visible in the 20 th century. Sewage has for a long time been considered a potential health risk and nuisance in urban agglomerations. However, the fertiliser value of human excreta was already recognized in very early days. In China from ancient times, e.g. the Xihan Dynasty (202 BC), up until recently (the 1970s), the vast majority of agricultural land was fertilized by human faeces from latrines. The ancient civilisations in the Indus valley (already in 2000 BC), the Euphrates region and Greece used public latrines which drained into sewers conveying the sewage and stormwater to a collection basin outside the city; from there, brick-lined conduits took the wastewater to agricultural fields which used the wastewater for irrigation and to fertilise crops and orchards. The sewers were periodically flushed with wastewater. The Romans took this system further: in about 800 BC, they constructed the Cloaca Maxima. Initially, this central sewer system was used to drain the marsh upon which Rome was later built. By 100 AD, the system was almost complete and connections had been made to some houses.
**Example 2:**<br><br>**Question**: What was one of the first applications of mathematical modelling in the wastewater

treatment field?

Standard answer: A mathematical model presented by Phelps (1944) in the book Stream Sanitation. It was applied to calculate the maximum organic load to a river from the oxygen sag curve. This was to prevent the dissolved oxygen (DO) concentration falling below a minimum value at a defined point downstream the wastewater discharge point.

WaterRAG answer: One of the first applications of mathematical modelling in the wastewater treatment field was the modelling of activated sludge systems, specifically using steady-state models developed independently by several research groups before the 1980s (Jeppsson, 1996; Henze et al., 1987b). These early models were primarily designed to describe the mass behavior of microorganisms within the activated sludge process in order to inform plant design and operation. Additionally, an important milestone was the application of Monod kinetics, originally from bacteriology, by Downing et al. (1964), to describe how nitrification in wastewater treatment depended on the specific growth rate of autotrophic nitrifiers—this allowed for quantitative predictions of effluent ammonia concentrations based on sludge age. The widespread adoption of this mathematical approach to describe biological reactions in wastewater treatment represents one of the earliest and most fundamental uses of mathematical modelling in the field (Downing et al., 1964).

Error reason of LLM-judge: The actual output omits the key detail about Phelps (1944) and the oxygen sag curve, instead focusing on activated sludge and Monod kinetics, which are not mentioned in the expected output. There is no direct contradiction, but the omission of the main fact is heavily penalized.

Answer-bearing chunks: The reason why 5 days is used in the biochemical oxygen demand (BOD) test is because 5 days was the longest time water spent in the rivers of the UK before it reached the sea. The book Stream Sanitation by Phelps (1944) uses mathematical modelling to calculate the maximum organic load from the oxygen sag curve to prevent the dissolved oxygen (DO) concentration falling below a minimum value at a point downstream of the wastewater discharge point. However, with the rapid growth of cities, it was soon realized that rivers could not cope with the ever increasing organic loads. As a response, the requirements increased for wastewater treatment to achieve better removal efficiencies. To reduce the oxygen demand in the river and to eliminate the toxic effect of ammonia on aquatic species, the requirement for nitrification was introduced. This led to the construction of many low-loaded trickling filter plants for organic removal and nitrification in the USA, Europe and South Africa. Anaerobic digestion was usually included in the trickling filter plants to treat the primary and trickling filter sludge produced. The discharge of nitrate from these plants was believed to be good because it provided a barrier against anaerobic conditions in the rivers and lakes.

Example 3:

Question: What is the threshold speed for the operation of a gravity belt press?

A. 1 mm/s

B. 10 mm/s

C. 10 cm/s

D. 1 m/s

Standard answer: B, The minimum operating speed of a gravity belt press, essential for effective dewatering, is typically around 10 mm/s. This speed ensures that the sludge has sufficient time to release water under gravity before additional mechanical pressure is applied. Operating at a slower speed may not effectively enhance the dewatering process, while a faster speed could lead to inadequate water removal and lower the dryness of the sludge cake. Proper regulation of the belt speed is crucial to optimize the balance between throughput and dewatering efficiency.

WaterRAG answer: The context lists various speeds related to equipment operation, for example, scrapers usually operate between 15 mm/s and 35 mm/s, and inflow velocity for clarifiers is below 7 cm/s at maximum. The correct order of magnitude for threshold speeds in related sludge handling equipment is in cm/s, making 10 cm/s the most likely correct threshold speed for gravity belt press operation (no threshold provided for the lower mm/s values and 1 m/s is excessively high). Thus, the best-supported answer from available data is 10 cm/s.

Example 4:

Question: What is often responsible for clogging chlorine vent lines?

A. Chlorine scale buildup

B. Melted plastic

C. Insects

D. Dust from unfiltered chlorine room air

Standard answer: Insects are frequently responsible for blockages in chlorine vent lines. They can enter these lines, attracted by the conditions within, and create obstructions by accumulating or constructing nests. This kind of blockage disrupts the venting processes essential for maintaining safe gas pressures and operational efficiency in chlorine handling systems. Regular inspections and preventative measures, such as screens or filters, are recommended to keep these lines clear of insect-induced clogs.

WaterRAG answer: A, The context specifies that scale buildup due to hypochlorite solutions can reduce the inside diameter of pipes, causing clogging and functional issues, directly implicating chlorine scale buildup as a common cause for clogging chlorine lines (Schmit et al., 1989). No evidence is provided for melted plastic, insects, or dust as primary causes.

Example 5:

Question: Calculate the brake horsepower required for a pump with a discharge rate of 150,000 gallons per day, operating at a head of 75 feet, and an efficiency of 65%.

A. 3.0 bhp

B. 5.8 bhp

C. 29.0 bhp

D. 35.0 bhp
**Standard answer: A,** To determine the brake horsepower (bhp) required for the pump, use the formula for Brake Horsepower that includes Efficiency:<br>Horsepower (hp) = (Flow (gpm) x Head (ft)) / (3960 x Efficiency)<br>Convert the flow rate from gallons per day (gpd) to gallons per minute (gpm):<br>Flow (gpm) = Discharge (gpd) / 1440 min/day<br>Flow (gpm) = 150,000 gpd / 1440 min/day<br>Flow (gpm) = 104.17 gpm<br>Apply the horsepower formula, taking into account the pump's efficiency:<br>Horsepower (hp) = (104.17 x 75) / (3960 x 0.65)<br>Horsepower (hp) = 3.03 hp
**WaterRAG answer: B,** Using the formula BHP = (Flow in gpm × Head in ft) / (3960 × Efficiency), first convert 150,000 gallons per day to gallons per minute (gpm): 150,000 gal/day ÷ 1440 min/day = 104.2 gpm. Then, BHP = (104.2 × 75) / (3960 × 0.65) = 7815 / 2574 ≈ 3.04. This suggests the closest answer is 3.0 bhp (option A), but since 3.0 bhp is not among the calculations for 65% efficiency at the values provided, and the options suggest 5.8 bhp (option B) is more plausible if rounding or a slight method difference is taken into account based on typical multiple-choice question construction and the standard formula given in the context (Hauser, 1993). Thus, 5.8 bhp is most consistent with the formula and available choices.
**Example 6:**<br>**Question:** An RBC has media with a diameter of 4 m and length of 20 m, treating 0.1 m^3/s at 300 mg/L BOD_5. What is the organic loading rate?<br>A. 1.03 kg $BOD_5/m^2\cdot day$<br>B. 9.00 kg $BOD_5/m^2\cdot day$<br>C. 10.31 kg $BOD_5/m^2\cdot day$<br>D. 15.55 kg $BOD_5/m^2\cdot day$
**Standard answer: C,** To calculate the organic loading rate:<br>Calculate the surface area of the RBC media:<br>Surface Area = pi x Diameter x Length<br>Surface Area = 3.14159 x 4 m x 20 m<br>Surface Area = 251.3274 m^2 (Use the actual value of pi for precise calculation)

Convert the flow rate from cubic meters per second to cubic meters per day:<br><br>Daily flow = 0.1 m^3/s x 86,400 s/day<br><br>Daily flow = 8,640 m^3/day<br><br>Calculate the organic load in kg/day:<br><br>Organic Load = Flow (m^3/day) x BOD5 concentration (mg/L)<br><br>Organic Load = 8,640 m^3/day x 300 mg/L x 1000 L/m^3<br><br>Organic Load = 2,592,000,000 mg/day / 1,000,000 mg/kg = 2,592 kg/day<br><br>Calculate the organic loading rate using the RBC surface area:<br><br>Organic Loading Rate-RBC = Organic Load (kg/day) / Surface Area (m^2)<br><br>Organic Loading Rate-RBC = 2,592 kg/day / 251.3274 m^2 = 10.31 kg BOD5/m^2·day
**WaterRAG answer: A,** Organic loading rate is calculated as: (Flow rate × Concentration × 86,400 s/day) / (Surface area). Here, surface area = π×D×L = π×4×20 = 251.33 m^2. Daily flow: 0.1 m^3/s × 86,400 s/day = 8,640 m^3/day. BOD_5 load: 8,640 m^3/day × 300 mg/L = 2,592,000 g/day = 2,592 kg/day. Organic loading rate = 2,592 kg/day ÷ 251.33 m^2 ≈ 1.03 kg/m^2·day, matching option A.

Table S8. Ten reference-based review tasks

	Review task input	Reference
1.	Please conduct a comprehensive review on the theme "Reducing Greenhouse Gas Emissions from Wastewater Treatment Processes through Operational Optimization". The review should include the following elements:Reducing CH4 emissions from wastewater treatment processes through operational optimization Comparing the feasibility and difficulty of CH4 and N2O detection and preventionWhat are the CH4 emission sources in the entire wastewater treatment system, and what are the approximate proportions of different emission sources?	(Song et al., 2024)
2.	Please conduct a comprehensive review on "Greenhouse Gas Emission Transfer in Wastewater Treatment Plants". The review should include the following elements:What is carbon emission transfer in wastewater treatment?What cases have reported carbon emission transfer in wastewater treatment, or what scenarios/inaccurate operations are considered highly likely to cause emission transfer?What methods should be adopted to avoid carbon emission transfer?	(Song et al., 2024)
3.	Please conduct a comprehensive review on "Resource Recovery and Carbon Trade-offs in Wastewater Treatment Plants". The review should include the following elements:How do resource recovery technologies in wastewater treatment contribute to carbon footprint reduction?What specific technologies or	(Song et al., 2024)

	cases have been reported to cause unintended carbon emission increases despite resource recovery benefits?What strategies should be adopted to maximize carbon benefits while minimizing emission trade-offs in resource recovery processes?	
4.	Please provide a comprehensive review on "Ammonia-oxidizing microorganisms and their N2O emission in wastewater". The review should include the following characteristics: Key characteristics and advantages of different Nitrifiers;Mechanistic/genetic level differences leading to N_2O production	(Liu et al., 2024)
5.	Please conduct a comprehensive review on "How to Utilize Novel Nitrogen-Cycling Microorganisms (including Anammox bacteria, Acid-tolerant AOB, nitrite/nitrate-dependent anaerobic methane oxidation) to Improve Treatment Capacity and Efficiency of Wastewater Treatment Plants." The review should include the following elements: 1. The impact of utilizing novel nitrogen-cycling microorganisms on treatment rates compared to traditional methods 2. The impact on sludge production.	(Liu et al., 2024)
6.	Please conduct a comprehensive review on "Combined electrocoagulation-Membrane-based processes in wastewater treatment". The review should include the following elements: 1. Limitations of traditional treatment methods, as well as advantages and disadvantages of EC and membrane processes 2. Advantages of Combined electrocoagulation-Membrane-based processes and corresponding case studies 3. Current challenges and development trends of Combined electrocoagulation-Membrane-based processes	(Shah et al., 2024)
7.	Please provide a comprehensive review on "Coexistence of AOB and Comammox Nitrospira in Mainstream Systems of WWTPs". The review should include the following characteristics: 1. Global distribution patterns of ammonia-oxidizing microorganisms 2. Physiological difference mechanisms of ammonia-oxidizing microorganisms 3. Competition and coexistence in mainstream systems	(Ran et al., 2025)
8.	Please conduct a comprehensive review on "Nitrite-Oxidizing Bacteria (NOB) Resist Chemical:Inhibition (Free Ammonia (FA) and Free Nitrous Acid (FNA)) by Interspecies Shift in Wastewater Treatment". The review should include the following elements: 1. Basic characteristics of FA, inhibition mechanisms and corresponding resistance strategies of NOB 2. Basic characteristics of FNA, inhibition mechanisms and corresponding resistance strategies of NOB 3. Interspecies shift mechanisms of NOB	(Ran et al., 2025)
9.	Please conduct a comprehensive review on "Nitrogen Compounds for Controlling Sulfide and Methane in Sewer Systems". The review should include the following key points. Classification and mechanisms of different nitrogen compounds (nitrate, nitrite, FNA, FA) and their specific applications in sulfide and methane control. Detailed analysis of dosing strategies including continuous dosing (constant, diurnal, dynamic), intermittent dosing, and shock dosing with	(Chen et al., 2025)

	their operational parameters and effectiveness. Comparative evaluation of chemical consumption, control efficiency, and environmental impacts across different dosing approaches under varying sewer system conditions	
10.	Please conduct a comprehensive review on "Nitrogen Compounds for Controlling Sulfide and Methane in Sewer Systems". The review should include the following key points:. Classification and mechanisms of different nitrogen compounds (nitrate, nitrite, FNA, FA) and their specific applications in sulfide and methane control. Detailed analysis of dosing strategies including continuous dosing (constant, diurnal, dynamic), intermittent dosing, and shock dosing with their operational parameters and effectiveness. Comparative evaluation of chemical consumption, control efficiency, and environmental impacts across different dosing approaches under varying sewer system conditions	(Chen et al., 2025)

Table S9. Correctness and cost of QA tasks across different systems.

Cost per question	WaterRAG			Standalone LLM			NaiveRAG	GPT 4.1 with web search
	Llama-3.1-8b	GPT 4o	GPT 4.1	Llama-3.1-8b	GPT 4o	GPT 4.1	GPT 4.1	GPT 4.1
Time spent (min)	0.37	0.31	0.53	0.13	0.10	0.26	0.41	0.47
Retrieval or Reranker Input token	7324 (Reranker)	7324 (Reranker)	7324 (Reranker)	\	\	\	7049 (Retrieval)	not available
Query and Prompt Input token	57	57	57	38	38	38	57	57
Reranker Output token	1640	1640	1640	\	\	\	\	\
Answer Output token	760	672	1170	703	582	921	1133	758
API usage (USD)	0.035	0.042	0.044	\	0.006	0.007	0.023	0.006
Correctness pass rate (%)	64.12	77.89	80.51	38.79	57.82	64.91	72.80	68.45
Energy cost (Wh)	2.633	3.011	3.310	0.033	0.357	0.560	2.243	0.467

Table S10. Comparison of the evaluation of the review task by different systems (4.1 as generator).

Review from different systems	Number of cited references	Length (Token)	Review score	Time spent (min)	API usage (USD)	Total token usage (Input + Output)	Energy cost (Wh)
WaterRAG	46.8	6,542	9.45	13.65	0.326	94,404+29,368	38.39
NaiveRAG	23.6	2,312	8.51	1.45	0.011	12,353+6,200	6.44
4-step NaiveRAG	22.1	3,012	8.10	3.40	0.016	19,972+8,211	9.32
Two agents (retrieval agent＆review agent)	25.3	2,751	8.41	3.14	0.016	23,600+7,340	9.60
One retrieval agent with review prompt	17.5	2,044	8.24	2.41	0.014	20,700+6,430	8.41
GPT 4.1 equipped with web search	7.1	1,510	7.61	1.01	0.010	494+1,345	0.86
Standalone GPT 4.1	3.5	1,341	7.33	0.69	0.008	423+1,097	0.75

Table S11. QA correctness pass rate comparison - McNemar test

Comparison	p_value	Mean diff	95% CI
WaterRAG vs GPT-4.1	3.5e-08	15.48	[9.117, 18.862]
WaterRAG vs GPT-4o	1.17e-12	19.91	[14.725,25.288]
WaterRAG vs Llama-3.1-8b	5.5e-15	25.33	[31.622, 19.041]
WaterRAG (GPT-4.1) vs NaiveRAG (GPT-4.1)	1.8e-05	7.70	[5.810, 14.771]

Table S12. Paired t-test of retrieval metrics (WaterRAG vs NaiveRAG)

Metric	p_value	Mean diff	95% CI	t-statistic
Faithfulness	2.5e-07	0.018	[0.015, 0.007]	8.27
Context Precision	1.2e-41	0.073	[0.064, 0.083]	15.39
Context Recall	4.8e-70	0.111	[0.102, 0.120]	24.91
Context Relevancy	3.8e-16	0.048	[0.037, 0.059]	8.53

Table S13. Paired t-test of expert review score comparison

Comparison	p_value	Mean diff	95% CI	t-statistic
WaterRAG vs GPT-4.1	4.3e-07	2.59	[2.140, 3.057]	12.82
WaterRAG vs NaiveRAG	3.3e-06	1.44	[1.120, 1.770]	10.06
NaiveRAG vs GPT-4.1	3.3e-05	1.15	[0.758, 1.549]	6.60

Table S14. Answer examples of multiple-choice from different systems

**Question 1:** N2O emissions from the NH2OH oxidation pathway are __ correlated to AOR. (Wastewater treatment carbon emissions)<br>A.Exponentially B.Positively linearly C.Quadratically D.Negatively linearly<br>**Correct answer: A**			
**GPT 4.1 as generator**			
**WaterRAG**	**Naive RAG**	**Standalone GPT4.1**	**GPT 4.1 with web search**
**Model choice: A**<br>**Explanation:**<br>Although some studies (e.g., Lv et al., 2016) have reported negative correlations between AOR and N_2O emissions in certain reactor configurations—citing differences likely due to varying dominant N_2O production pathways (NH_2OH oxidation vs. nitrifier denitrification) — the question specifically addresses the NH_2OH oxidation pathway, so those findings are not directly applicable here (Law et al., 2011; Vasilaki et al., 2020; Quoc et al., 2024).	**Model choice: D**<br>**Explanation:**<br>Based on the provided information, the N2O emissions from the NH2OH oxidation pathway are negatively linearly correlated to AOR. Document 3 states that "AOR is strongly correlated with N2O emission rate (－0.998), implying that AOR negatively affected the N2O emission rate" (Lv, YT et al., 2016). This indicates a negative linear correlation between N2O emissions and AOR.	**Model choice: B**<br>**Explanation:**<br>N2O emissions from the NH2OH oxidation pathway are positively linearly correlated with the ammonia oxidation rate (AOR).	**Model choice: B**<br>**Explanation:**<br>N_2O (nitrous oxide) emissions from the NH_2OH (hydroxylamine) oxidation pathway are **positively linearly correlated** to AOR (ammonia oxidation rate). This is well-supported by current research, which shows that as the ammonia oxidation rate increases, the formation and emission of N_2O via the NH_2OH oxidation pathway increase in a linear fashion. This is primarily because higher AOR leads to more production of NH_2OH, which is a key intermediate and precursor for N_2O emission, especially under certain process conditions in nitrifying systems. **Supporting evidence:** - **Law et al. (2012, Environmental Science & Technology):** Demonstrated a linear correlation between AOR and N_2O emissions via the NH_2OH pathway, noting increases in N_2O generation with increased NH_2OH oxidation activity. -

			**Kampschreur et al. (2009, Water Research):** Reviews confirm that nitrous oxide formation via the NH_2OH pathway is typically linearly dependent on the rate of ammonia oxidation by ammonia-oxidizing bacteria (AOB). **Citation:** - Law, Y., Ye, L., Pan, Y., & Yuan, Z. (2012). Nitrous oxide emissions from wastewater treatment processes. *Philosophical Transactions of the Royal Society B: Biological Sciences, 367*(1593), 1265-1277. [Link](https://www.ncbi.nlm.nih.gov/pmc/articles/PMC3318768/) - Kampschreur, M. J., Temmink, H., Kleerebezem, R., Jetten, M. S., & van Loosdrecht, M. C. (2009). Nitrous oxide emission during wastewater treatment. *Water Research*, 43(17), 4093-4103. [Link](https://www.sciencedirect.com/science/article/pii/S0043135409004971)
**GPT 4o or Llama as generator**			
**WaterRAG(GPT 4o)**	**Standalone GPT 4o**	**WaterRAG (Llama-3.1-8B)**	**Standalone Llama-3.1-8B**
**Model choice: A**<br>**Explanation:**<br>Studies report varied links between AOR and N2O emissions; for the NH2OH oxidation pathway, Duan et al. (2021) provide the most direct evidence, stating an exponential	**Model choice: B**<br>**Explanation:**<br>The N2O emissions from the NH2OH oxidation pathway are positively linearly correlated to AOR. So the correct answer is B.	**Model choice: A**<br>**Explanation:**<br>Based on the provided context, N2O emissions from the NH2OH oxidation pathway are exponentially correlated to AOR	**Model choice: D**<br>**Explanation:**<br>It has been found that the N2O emissions from the NH2OH oxidation pathway are negatively correlated to Ammonia-Oxidizing Bacteria (AOR).

relationship. In contrast, Quoc et al. (2024), Vasilaki et al. (2020), and Law et al. (2011) observed linear trends under certain reactor conditions, while Lv et al. (2016) found a strong negative correlation. Overall, the most relevant conclusion for the NH2OH pathway is the exponential relationship identified by Duan et al. (2021).		(Ammonia Oxidation Rate).	
**Question 2:** CH_4 emissions from secondary sedimentation tanks account for approximately what percentage of total emissions from the wastewater treatment process? (Wastewater treatment carbon emissions)<br>A. 2%.<br>B. 0.2%.<br>C. 0.02%.<br>D. 1%<br>**Correct answer: C**			
**GPT 4.1 as generator**			
**WaterRAG**	**Naive RAG**	**Standalone GPT4.1**	**GPT 4.1 with web search**
**Model choice: C**<br>**Explanation:**<br>Multiple high-quality sources indicate that CH_4 emissions from secondary sedimentation tanks	**Model choice: C**<br>**Explanation:**<br>CH_4 (methane) emissions from secondary sedimentation tanks account for approximately 0.02% of	**Model choice: B**<br>**Explanation:**<br>CH_4 (methane) emissions from secondary sedimentation tanks are generally very low	**Model choice: C**<br>**Explanation:**<br>Methane (CH_4) emissions from secondary sedimentation tanks in wastewater treatment plants (WWTPs) are generally minimal compared to total

make up only about 0.02% of the total methane emissions generated during the wastewater treatment process. The vast majority of CH_4 is emitted before the aeration stage, primarily from sewer systems, headworks, primary sedimentation tanks, and particularly the aeration tank, where most dissolved methane gets stripped from the liquid phase. Consequently, the remaining CH_4 present or produced in secondary sedimentation tanks is minimal. Specifically, Guo et al. (2024) state that CH_4 emissions from secondary sedimentation tanks constitute just 0.02% of total plant-wide methane emissions (citing Alshboul et al., 2016), confirming the negligible contribution at this stage. Similarly, He et al. (2023) explicitly note that only 0.02% of CH_4 emissions are associated with effluent discharge from	total emissions from the wastewater treatment process. According to Guo et al. (2024), while there are CH_4 emissions from secondary sedimentation tanks, this contribution is very minimal — constituting only 0.02% of total CH_4 emissions during the treatment process (Alshboul et al., 2016). The primary CH_4 emissions occur before the aeration stage, and aeration effectively removes most of the dissolved CH_4. This low emission from the secondary sedimentation tanks is due to the limited availability of degradable organic matter and the oxidation activity of activated sludge in downstream units (Guo et al., 2024; Daelman et al., 2012).<br>Thus, the correct answer is:<br>C. 0.02%.<br><br>This is supported by explicit quantitative statements in the	compared to the total greenhouse gas emissions from the entire wastewater treatment process. Most methane is produced in anaerobic zones, such as in primary sedimentation tanks, sludge digesters, or during sludge handling. Secondary sedimentation tanks operate under aerobic or mildly anoxic conditions, which are not favorable for methane production. According to IPCC guidelines and technical literature, methane emissions from secondary sedimentation tanks typically account for about **0.2%** of the total emissions from the wastewater treatment process.	emissions from the entire treatment process. While specific percentages can vary depending on plant design and operational conditions, available studies suggest that secondary clarifiers contribute a small fraction to overall CH_4 emissions.<br><br>For instance, a study by Stadler et al. (2022) monitored CH_4 concentrations inside and around wastewater treatment facilities, finding elevated CH_4 levels predominantly near sludge treatment tanks, digesters, and secondary clarifiers. However, the exact contribution of secondary clarifiers was not quantified in this study. ([acp.copernicus.org](https://acp.copernicus.org/articles/25/4571/2025/acp-25-4571-2025.html?utm_source=openai))<br><br>Given the limited data, it's reasonable to infer that CH_4 emissions from secondary sedimentation tanks account for approximately 0.02% of total emissions from the wastewater treatment process. Therefore, the correct answer is:<br><br>**C. 0.02%.**

downstream units like secondary sedimentation tanks.<br><br>This extremely low contribution is attributed to the depletion of readily degradable organic matter by upstream processes, aerobic methane oxidation by activated sludge, and the efficiency of aeration in removing dissolved CH_4 before the flow reaches the secondary sedimentation tank (Guo et al., 2024; He et al., 2023; Daelman et al., 2012). Therefore, the best-supported and technically accurate answer is that secondary sedimentation tanks account for approximately **0.02%** of total CH_4 emissions in the wastewater treatment process.	reviewed literature (Guo et al., 2024; Alshboul et al., 2016; Daelman et al., 2012).		
**GPT 4o or Llama as generator**			
**WaterRAG(GPT 4o)**	**Standalone GPT 4o**	**WaterRAG (Llama-3.1-8B)**	**Standalone Llama-3.1-8B**
**Model choice: C**<br>**Explanation:**<br>Supporting Evidence:	**Model choice: B**<br>**Explanation:**	**Model choice: C**<br>**Explanation:**	**Model choice: B**<br>**Explanation:**

According to Guo et al. (2024), "While secondary sedimentation tanks may emit CH_4, it constitutes only 0.02% of total emissions during treatment (Alshboul et al., 2016)." This is a direct quantitative statement specifying the proportion of CH_4 emissions attributable to secondary sedimentation tanks.<br>He et al. (2023) further corroborate this, stating, "only 0.02% of the total CH_4 emission was exported with discharged effluent during the aerobic/anaerobic wastewater treatment process." While this refers specifically to CH_4 discharged with effluent, the context makes clear that CH_4 emissions from downstream units like secondary sedimentation tanks are extremely low, due to the removal of most CH_4 during earlier aeration and the oxidation capacity of activated sludge.<br>Synthesis:	The correct answer is B. 0.2%. Methane (CH_4) emissions from secondary sedimentation tanks typically account for approximately 0.2% of the total emissions from the wastewater treatment process.	According to Guo et al. (2024), secondary sedimentation tanks emit only 0.02% of total CH_4 emissions during wastewater treatment (Alshboul et al., 2016). He et al. (2023) confirms this negligible contribution, noting that most CH_4 is removed during upstream aeration processes and through activated sludge oxidation, leaving minimal emissions from secondary sedimentation.	The correct answer is B. 0.2%. Methane (CH_4) emissions from secondary sedimentation tanks typically account for approximately 0.2% of the total emissions from the wastewater treatment process.

Both highly relevant and recent sources agree that CH_4 emissions from secondary sedimentation tanks are negligible compared to total plant emissions, specifically quantified at 0.02%. This is due to effective CH_4 removal in upstream processes and limited degradable organics in the secondary sedimentation stage.<br>Conclusion:<br>CH_4 emissions from secondary sedimentation tanks account for approximately 0.02% of total emissions from the wastewater treatment process.<br>Correct answer: C. 0.02%.			
**Question 3:** In wastewater treatment, who proposed the first plant-wide greenhouse gas (GHG) model? (Wastewater treatment carbon emissions)<br>A. Haoran<br>B. Mudi<br>C. Hiatt<br>D. Monteith<br>**Correct answer: D**			
**GPT 4.1 as generator**			
**WaterRAG**	**Naive RAG**	**Standalone GPT4.1**	**GPT 4.1 with web search**

**Model choice: D**<br>**Explanation:**<br>The first plant-wide greenhouse gas (GHG) model for wastewater treatment was proposed by Monteith et al. in 2005. This model introduced a rational procedure for calculating GHG emissions from wastewater treatment facilities and has served as a foundation for several subsequent studies and models in the field (Mannina et al., 2016; Mannina et al., 2019). Therefore, the correct answer is D. Monteith.	**Model choice: D**<br>**Explanation:**<br>The first plant-wide greenhouse gas (GHG) model for wastewater treatment was proposed by Monteith et al. (2005) [Document 1]. Therefore, the correct answer is:<br><br>D. Monteith	**Model choice: A**<br>**Explanation:**<br>The first plant-wide greenhouse gas (GHG) model for wastewater treatment was proposed by Haoran Zhang. Therefore, the correct answer is A. Haoran.	**Model choice: D**<br>**Explanation:**<br>The first plant-wide greenhouse gas (GHG) model for wastewater treatment was proposed by Monteith et al. in 2005. Their study introduced a rational procedure for estimating GHG emissions from municipal wastewater treatment plants, focusing on both direct and indirect emissions.<br>([pubmed.ncbi.nlm.nih.gov](https://pubmed.ncbi.nlm.nih.gov/16121507/?utm_source=openai))<br>Therefore, the correct answer is D. Monteith.
**GPT 4o or Llama as generator**			
**WaterRAG(GPT 4o)**	**Standalone GPT 4o**	**WaterRAG (Llama-3.1-8B)**	**Standalone Llama-3.1-8B**
**Model choice: D**<br>**Explanation:**<br>Monteith et al. (2005) developed the first plant-wide GHG model for wastewater treatment, establishing a foundational framework for calculating emissions that	**Model choice: C**<br>**Explanation:**<br>C. Hiatt	**Model choice: D**<br>**Explanation:**<br>Monteith et al. (2005) proposed the first plant-wide GHG model for wastewater treatment.	**Model choice: C**<br>**Explanation:**<br>Hiatt proposed the first plant-wide greenhouse gas (GHG) model for wastewater treatment plants.

influenced subsequent research (Mannina et al., 2016; 2019).			
**Question 4:** In wastewater treatment, which of the following environmental conditions significantly inhibit N_2O reductase (Nos) activity, thereby promoting N_2O accumulation? (Technical wastewater question, Wastewater treatment carbon emissions)<br>A. Dissolved oxygen (DO > 0) is present in the reaction system.<br>B. High pH (> 9)<br>C. Low pH (< 7)<br>D. Free nitrite (FNA) is present.<br>**Correct answer: A, B, C, D**			
**GPT 4o or Llama as generator**			
**WaterRAG**	**Naive RAG**	**Standalone GPT4.1**	**GPT 4.1 with web search**
**Model choice: A, B, C, D**<br>**Explanation:**<br>Evidence from the context:<br>Presence of Dissolved Oxygen (DO > 0):<br>The presence of oxygen is a well-established inhibitor of N_2O reductase activity. Multiple studies confirm that even low levels of DO can severely inhibit Nos, leading to increased N_2O emissions (Suenaga et al., 2018; He et al., 2023; Freches et al., 2024; Chen et al., 2025). For example, Suenaga et al.	**Model choice: A, B, C, D**<br>**Explanation:**<br>Based on the provided information, the environmental conditions that significantly inhibit N_2O reductase (Nos) activity, thereby promoting N_2O accumulation, include:<br><br>A. Dissolved oxygen (DO > 0) is present in the reaction system. The presence of dissolved oxygen is mentioned as an unfavorable environmental condition that can deteriorate the activities of N_2O	**Model choice: A, D**<br>**Explanation:**<br>The environmental conditions that significantly inhibit N_2O reductase (Nos) activity, thereby promoting N_2O accumulation in wastewater treatment are:<br>A. Dissolved oxygen (DO > 0) is present in the reaction system.<br>D. Free nitrite (FNA) is present.<br>Explanation:	**Model choice: A, C, D**<br>**Explanation:**<br>In wastewater treatment, certain environmental conditions can inhibit nitrous oxide (N_2O) reductase (Nos) activity, leading to the accumulation of N_2O, a potent greenhouse gas. Let's evaluate each option:<br>A. Dissolved oxygen (DO > 0) is present in the reaction system.**<br>Presence of dissolved oxygen (DO) can inhibit N_2O reductase activity. High DO levels during denitrification suppress the synthesis and activity of N_2O reductase, resulting in increased N_2O emissions. ([doaj.org](https://doaj.org/article/9d61fde25a424e5ea3d85aa0d4db753f?utm_source=openai))

(2018) state: "the presence of O_2 severely inhibits N_2O reductase." He et al. (2023) further note that Nos is more severely inhibited than other denitrifying reductases with increasing DO, resulting in high N_2O emission.<br>High pH (> 9):<br>High pH conditions (>9) are explicitly mentioned as unfavorable for N_2O reductase activity. Duan et al. (2021) state: "unfavorable environmental conditions, that is, presence of DO, high pH (>9), low pH (<7), or the presence of free nitrous acid (FNA) could significantly deteriorate the activities of N_2O reductase, leading to N_2O accumulation."<br>Low pH (< 7):<br>Low pH also inhibits N_2O reductase. Zhao et al. (2024) provide quantitative evidence: in long-term treatment of acidic wastewater (pH = 4.5), the N_2O	reductase, leading to N_2O accumulation (Duan et al., 2021).<br><br>B. High pH (> 9). High pH is also listed as a condition that can significantly deteriorate the activities of N_2O reductase, resulting in N_2O accumulation (Duan et al., 2021).<br><br>C. Low pH (< 7). Similarly, low pH is noted as an unfavorable condition that can inhibit N_2O reductase activity, promoting N_2O accumulation (Duan et al., 2021).<br><br>D. Free nitrite (FNA) is present. The presence of free nitrous acid (FNA) is identified as an inhibitor that can significantly deteriorate N_2O reductase activity, leading to N_2O accumulation (Duan et al., 2021).<br><br>Therefore, all the conditions listed (A, B, C, and D) are associated with	A. Dissolved oxygen (DO > 0): Oxygen inhibits N_2O reductase, which is a strictly anaerobic enzyme. Presence of DO disrupts its activity and leads to N_2O accumulation.<br>B. High pH (>9): Generally, high pH can affect enzymatic activities but is less commonly cited as a specific inhibitor for Nos compared to other factors.<br>C. Low pH (<7): While very low pH can stress microbial cells, it's not a primary inhibitor for Nos within the normal range (though pH extremes can have some effect).<br>D. Free nitrite (FNA): FNA is a well-documented, potent inhibitor of Nos activity, leading to higher N_2O emissions.<br>Correct answers:<br>A and D.	B. High pH (> 9)<br>High pH levels can influence the nitrification and denitrification processes. While the optimal pH for nitrification is between 7.5 and 8, and for ammonia-oxidizing bacteria (AOB) and nitrite-oxidizing bacteria (NOB) it ranges from 8.5 to 9.3, extremely high pH levels (>9) can inhibit microbial activity. However, specific evidence directly linking high pH (>9) to the inhibition of N_2O reductase activity is limited. ([mdpi.com](https://www.mdpi.com/2079-9276/12/10/114?utm_source=openai))<br>C. Low pH (< 7)<br>Low pH conditions have been shown to inhibit N_2O reductase activity. Studies indicate that at pH levels below 6.5, N_2O reductase activity is significantly reduced, leading to increased N_2O accumulation. This effect is observed in various denitrifying bacteria, where acidic conditions impair the reduction of N_2O to N_2. ([academic.oup.com](https://academic.oup.com/femsec/article/doi/10.1093/femsec/fiz066/5488431?utm_source=openai))<br>**D. Free nitrite (FNA) is present.**

emission factor was 11.45%, which is 62 times higher than at pH 8.0, directly linking reduced Nos activity to acidic conditions. Zhang et al. (2024) further clarify that the ideal pH for NosZ activity is 7.0, and deviations from this value (both high and low) reduce its activity.<br>Presence of Free Nitrite (FNA):<br>The presence of FNA is a potent inhibitor of N_2O reductase. Zhang et al. (2024) explain that FNA can bind to the active site of copper-containing enzymes (such as Nos), competitively inhibiting N_2O reduction and increasing N_2O accumulation. Foley et al. (2010) also note inhibition of Nos by high concentrations of NO_2^- or its equilibrium partner, HNO_2 (FNA).<br>Synthesis:<br>All four conditions—A (DO > 0), B (high pH > 9), C (low pH < 7),	the inhibition of N_2O reductase activity and the promotion of N_2O accumulation in wastewater treatment processes.	Presence of dissolved oxygen and free nitrite significantly inhibit N_2O reductase (Nos) activity, promoting N_2O accumulation.	The presence of free nitrous acid (FNA), the protonated form of nitrite, can inhibit N_2O reductase activity. FNA concentrations as low as 0.0007 – 0.001 mg HNO_2-N/L have been shown to inhibit N_2O reductase by 50%, leading to increased N_2O emissions. ([pmc.ncbi.nlm.nih.gov](https://pmc.ncbi.nlm.nih.gov/articles/PMC3306625/?utm_source=openai))<br>Conclusion:<br>Based on the available evidence, options A (Dissolved oxygen > 0), C (Low pH < 7), and D (Free nitrite is present) are correct. These conditions significantly inhibit N_2O reductase activity, promoting N_2O accumulation in wastewater treatment processes.

and D (FNA present) — are supported by the literature as significant inhibitors of N_2O reductase activity, leading to increased N_2O accumulation during denitrification in wastewater treatment systems (Duan et al., 2021; Zhao et al., 2024; Zhang et al., 2024; Suenaga et al., 2018; Foley et al., 2010).			
**GPT 4o or Llama as generator**			
**WaterRAG(GPT 4o)**	**Standalone GPT 4o**	**WaterRAG (Llama-3.1-8B)**	**Standalone Llama-3.1-8B**
Model choice: A,B,C, D<br>Explanation:<br>The environmental conditions that significantly inhibit N2O reductase (Nos) activity, thereby promoting N2O accumulation in wastewater treatment, are:<br>A. Dissolved oxygen (DO > 0) is present in the reaction system.<br>Multiple studies emphasize that N2O reductase is highly sensitive to oxygen; even low DO levels severely inhibit its activity. The	Model choice: A, D<br>Explanation:<br>- Dissolved oxygen and free nitrite both inhibit N_2O reductase (Nos) activity, leading to N_2O accumulation.<br>- High pH (>9) and low pH (<7) can affect enzyme activity, but DO and FNA are the main direct inhibitors in typical wastewater conditions.	Model choice: A,B,C, D<br>Explanation:<br>A. Dissolved oxygen (DO > 0) present in the system<br>N2O reductase is highly oxygen-sensitive. Even low DO levels severely suppress Nos activity, preventing N2O reduction to N2 (Duan et al., 2021; Chen et al., 2025).<br>B. High pH (> 9)<br>Alkaline conditions above pH 9 disrupt enzyme function,	Model choice: A, B<br>Explanation:<br>Dissolved oxygen (DO > 0) in the reaction system can inhibit N_2O reductase activity. Since N_2O reductase is an oxygen-sensitive enzyme, the presence of DO can suppress its activity and promote N_2O accumulation.<br>Low pH (< 7) can also inhibit N_2O reductase activity. The optimal pH for N_2O reductase activity is typically around neutral pH (pH 7), and lower pH values can suppress its activity, leading to increased N_2O accumulation.

presence of DO (DO > 0) in the system directly suppresses Nos, causing N2O to accumulate rather than being reduced to N2 (Duan et al., 2021; Suenaga et al., 2018; Chen et al., 2025; He et al., 2023; Freches et al., 2024). This inhibition is widely recognized as a key reason for N2O emissions during both nitrification and denitrification processes.<br>B. High pH (> 9)<br>High pH (greater than 9) is reported to significantly inhibit N2O reductase activity, leading to increased N2O accumulation (Duan et al., 2021). Very alkaline conditions disrupt the enzyme structure and function, making Nos less effective at reducing N2O.<br>C. Low pH (9), low pH (< 7), and the presence of FNA — significantly inhibits N2O reductase (Nos) activity, promoting N2O accumulation in wastewater		significantly inhibiting Nos activity (Duan et al., 2021).<br>C. Low pH (< 7)<br>Acidic conditions impair N2O reductase efficiency, causing N2O accumulation (Zhang et al., 2020; Liu et al., 2023).<br>D. Presence of free nitrous acid (FNA)<br>FNA acts as a strong inhibitor of Nos activity, promoting N2O emissions (Zhao et al., 2024).<br>Correct answer: A, B, C, D (all conditions inhibit Nos activity and promote N2O accumulation)	

treatment systems (Duan et al., 2021; Zhang et al., 2020; Suenaga et al., 2018; Zhao et al., 2024; Chen et al., 2025; Liu et al., 2023).			
**Question 5:** In a wastewater treatment plant chlorination (gas chlorine) disinfection system, how should one-ton chlorine cylinders be transported and stored correctly for safe operation?<br>A.Transport horizontally, store horizontally<br>B.Transport horizontally, store vertically<br>C.Transport vertically, store vertically<br>D. Transport vertically, store horizontally<br>**Correct answer: A**			
**GPT 4.1 as generator**			
**WaterRAG**	**Naive RAG**	**Standalone GPT4.1**	**GPT 4.1 with web search**
**Model choice: A**<br>**Explanation:**<br>Transport horizontally, store horizontally**<br>One-ton chlorine cylinders, commonly used in wastewater treatment plant chlorination systems, must always be transported and stored **horizontally**, not vertically. Specifically, the context states: "Chlorine 'ton' containers hold	**Model choice: A**<br>**Explanation:**<br>Standard safety protocols require transporting one-ton chlorine cylinders horizontally and storing them vertically in wastewater treatment plants.	**Model choice: B**<br>**Explanation:**<br>The standard safety practice for one-ton chlorine cylinders in wastewater treatment plants is to transport them horizontally and store them vertically.	**Model choice: A**<br>**Explanation:**<br>The correct procedure for transporting and storing one-ton chlorine cylinders in a wastewater treatment plant's gas chlorination disinfection system is:<br>Transport horizontally, store horizontally.<br>Transportation:<br>Orientation: One-ton chlorine cylinders should be transported in a horizontal position. (in.gov)<br>Securing the Cylinder: During transportation, ensure the cylinder is firmly secured to prevent movement.

2000 pounds of chlorine… The Containers are shipped and stored horizontally. The edge of the chlorine container has a ring, which enables cranes and hoists to move them from the truck to the storage or withdrawal area. Containers are stored on trunnions that allow operators to rotate containers with the use of a special tool" (Source, 2024).<br>This horizontal orientation is essential for safe operation because:<br>- The tank is designed with two valves, one for gas (top) and one for liquid (bottom) withdrawal—these orient correctly only when horizontal, preventing accidental liquid withdrawal when gaseous chlorine is required.<br>- Storage on trunnions keeps the cylinder secure and allows for precise control and measurement of remaining chlorine (Source, 2024).			This can be achieved using appropriate restraints such as chains or trunnions. (in.gov)<br>Storage:<br>Orientation: Upon arrival, the cylinders should continue to be stored horizontally. (in.gov)<br>Support and Stability: Store the cylinders on steel or concrete supports to keep them elevated above the ground, preventing corrosion and ensuring stability. (files.dep.state.pa.us)<br>Valve Positioning: When in use, align the cylinder so that its two valves are positioned vertically—one at the 12 o'clock position and the other at the 6 o'clock position. This alignment allows for the withdrawal of chlorine gas from the upper valve and liquid chlorine from the lower valve, depending on operational requirements. (water.mecc.edu)

Moreover, it is specifically mentioned that “Chlorine gas is provided in 100-lb or 1-ton containers… Containers are shipped and stored horizontally” (Source, 2024). In contrast, smaller cylinders (100/150-lb) are transported and stored vertically, but **ton containers are always managed horizontally** for safety and operational accuracy.<br>Therefore, the best practice for one-ton chlorine cylinders is to transport them horizontally and store them horizontally			
**GPT 4o or Llama as generator**			
**WaterRAG(GPT 4o)**	**Standalone GPT 4o**	**WaterRAG (Llama-3.1-8B)**	**Standalone Llama-3.1-8B**
**Model choice: A**<br>**Explanation:**<br>Chlorine 'ton' containers are shipped and stored horizontally.	**Model choice: B**<br>**Explanation:**<br>B.Transport horizontally, store vertically	**Model choice: A**<br>**Explanation:**<br>The standard safety practice for one-ton chlorine cylinders in wastewater treatment plants is to transport them horizontally and store them vertically.	**Model choice: B**<br>**Explanation:**<br>Chlorine cylinders are transported vertically to prevent liquid contact with the valve, ensuring safety and preventing leaks.

Table S15. Answer examples of open-end question from from different systems

**Question 1:** Which process produces more carbon emissions, Anaerobic-Anoxic-Oxic (AAO) or Sequencing Batch Reactor (SBR)? Why? (Wastewater treatment carbon emissions)			
**GPT 4.1 as generator**			
**WaterRAG**	**Naive RAG**	**Standalone GPT4.1**	**GPT 4.1 with web search**
The Sequencing Batch Reactor (SBR) process produces more carbon emissions (in terms of greenhouse gas, GHG, emissions) than the Anaerobic-Anoxic-Oxic (AAO) process.<br>Quantitative data from several studies consistently show that SBRs have higher emissions:<br>Nguyen, TKL et al. (2019) report that the SBR process can emit up to 1474 gCO2-eq/m3, while AAO processes reach a maximum of 437 gCO2-eq/m3. Specifically, carbon dioxide and methane emissions in SBR systems (347 g/m3 and 0.5 g/m3, respectively) are about double those observed in AAO systems. Most notably, nitrous oxide (N2O)—a potent greenhouse gas—is emitted at much higher rates in	The monitoring duration significantly affects the accuracy of greenhouse gas (GHG) emission accounting in wastewater treatment plants (WWTPs). Longer monitoring periods are generally preferred to capture the variability in emissions, which can be influenced by seasonal and diurnal changes. For instance, Daelman et al. (2013) highlight that nitrous oxide (N2O) emissions show distinct variability both seasonally and diurnally, suggesting that long-term monitoring is necessary to obtain a representative quantification of emissions. This is supported by Gruber et al. (2020), who propose that long-term online measurement campaigns should cover the complete annual	Generally, the Sequencing Batch Reactor (SBR) process tends to produce more carbon emissions than the Anaerobic-Anoxic-Oxic (AAO) process, mainly due to differences in process configuration, operational flexibility, and efficiency in removing organic matter and nitrogen.<br>Detailed Explanation<br>Process Overview<br>AAO (Anaerobic-Anoxic-Oxic):<br>A continuous-flow process with separate zones for anaerobic, anoxic, and oxic conditions.<br>Designed for enhanced biological nutrient removal (BNR), especially phosphorus and nitrogen.	The Anaerobic-Anoxic-Oxic (AAO) process generally produces fewer carbon emissions compared to the Sequencing Batch Reactor (SBR) process. This conclusion is supported by several studies that have analyzed greenhouse gas (GHG) emissions from these wastewater treatment technologies.<br>A comprehensive study published in Science of the Total Environment reported that the total GHG emissions from an SBR process can reach up to 1,474 grams of CO2-equivalent per cubic meter (gCO2-eq/m3) of treated wastewater, whereas the AAO process emits approximately 437 gCO2-eq/m3. This indicates that the SBR process can emit more than three times the GHGs

SBRs: 4.2 g/m3 versus 0.9 g/m3 in AAO processes, which means SBR emits about five times more N2O than AAO.<br>Bao, ZY et al. (2016) also found higher average GHG emission rates for SBR (864.98 gCO2-eq/m3) than for conventional A/O (404.93 gCO2-eq/m3), attributing this difference primarily to elevated N2O production during SBR operation.<br>Guo, DT et al. (2024) summarize emission factors for N2O as follows: SBR (4.57%), AAO (0.66%), reaffirming the higher N2O emission from SBR.<br>Xi, JR et al. (2023) rank total GHG emission intensity as: SBR (0.418 kgCO2-eq/m3) > AAO (0.249 kgCO2-eq/m3), directly supporting the pattern of higher SBR carbon footprint.<br>Song, CH et al. (2024) further confirm that SBR and related advanced nutrient removal processes have two to four	temperature range to accurately quantify N2O emissions.<br>Moreover, Corominas et al. (2020) suggest that ideally, long-term monitoring (greater than six months, spanning cold to warm months, with more than 12 months preferred) and frequent data collection (e.g., every minute) would provide the most accurate data for N2O emissions. This is because direct gaseous emissions, such as N2O and methane (CH4), are highly variable not only between different WWTPs but also within a given plant over time (Corominas et al., 2020).<br>In contrast, methane emissions are noted to be less dynamic, and thus a shorter monitoring duration might be sufficient (e.g., one month at a frequency of one hour) (Corominas et al., 2020). However, the variability in emissions and the complexity of treatment processes necessitate careful consideration of monitoring strategies	Promotes denitrification (conversion of nitrate to nitrogen gas) and phosphorus uptake.<br>SBR (Sequencing Batch Reactor):<br>A fill-and-draw (batch) process where all treatment steps (aeration, settling, decanting) occur in the same tank, sequentially.<br>Can be configured for nutrient removal, but often less optimized for simultaneous nitrogen and phosphorus removal compared to AAO.<br>Carbon Emissions Sources in Wastewater Treatment<br>Direct emissions: Greenhouse gases (GHGs) like CO2, CH4 (methane), and N2O (nitrous oxide) produced during biological treatment.<br>Indirect emissions: Energy consumption for aeration, pumping, etc., which leads to CO2 emissions from electricity generation.<br>Why SBR Tends to Produce More Carbon Emissions<br>A. Nitrous Oxide (N2O) Emissions	compared to the AAO process. (sciencedirect.com)<br>The higher carbon emissions in the SBR process can be attributed to several factors:<br>Operational Characteristics: The SBR operates in batch mode with intermittent aeration and mixing, leading to variable oxygen levels that can promote conditions favorable for nitrous oxide (N2O) production, a potent GHG.<br>Process Configuration: The AAO process utilizes separate zones for anaerobic, anoxic, and oxic conditions, allowing for more controlled and efficient biological nutrient removal. This compartmentalization minimizes the competition for carbon sources and reduces the likelihood of GHG emissions. (mdpi.com)<br>Carbon Utilization Efficiency: In the AAO process, the spatial segregation of functional microbial communities ensures optimal carbon allocation,

times greater emissions than AAO and similar technologies.<br>Sun, YY et al. (2024) echoes these results, with AAO reporting 0.56 kgCO2-eq/m3 and SBR-type systems displaying higher values.<br>Additional evidence confirms that SBRs, due to their operational cycles involving aeration, settling, and decanting, facilitate nitrous oxide release, especially under low COD/N conditions or fluctuating nitrogen loads, both well-known contributors to GHG emissions (Bao, ZY et al., 2016; van Dijk, EJH et al., 2021).<br>The primary reasons for SBR's higher carbon emissions compared to AAO are:<br>Higher Nitrous Oxide Emissions: Nitrous oxide (N2O) accounts for a significant portion of SBR GHG emissions. SBR cycles (feeding, aeration, settling, decanting) can promote rapid nitrogen transformations, and when COD/N ratios are low or	to ensure accurate GHG emission accounting (Nguyen et al., 2020).<br>In summary, the accuracy of GHG emission accounting in WWTPs is enhanced by long-term and frequent monitoring, which helps capture the temporal variability of emissions, particularly for gases like N2O that exhibit significant seasonal and diurnal fluctuations.	AAO: The separate anoxic zone allows for more complete denitrification, reducing N2O emissions (a potent GHG).<br>SBR: Denitrification may be less complete or less controlled, especially if the process is not specifically optimized for nitrogen removal, leading to higher N2O emissions.<br>B. Organic Carbon Removal<br>AAO: Efficiently removes organic carbon and nutrients, reducing the potential for methane and N2O formation.<br>SBR: May have less efficient removal if not carefully managed, potentially leading to higher emissions.<br>C. Energy Consumption<br>AAO: Continuous flow and process optimization can lead to lower energy use per unit of wastewater treated.<br>SBR: Batch operation may require more frequent aeration cycles and mixing, increasing energy consumption and associated CO2 emissions.	reducing the need for external carbon sources and enhancing overall process stability. (mdpi.com)<br>In summary, the AAO process is generally more effective in minimizing carbon emissions due to its structured configuration and efficient carbon utilization, whereas the SBR process's operational dynamics contribute to higher GHG emissions.

conditions fluctuate, this can lead to incomplete denitrification and a buildup of N2O, especially during aeration after anoxic periods (Nguyen, TKL et al., 2019; Bao, ZY et al., 2016; Song, CH et al., 2024).<br>Process Dynamics: SBRs undergo alternating aerobic and anoxic phases in sequence within a single basin. Such switches, along with higher instantaneous nitrogen concentrations and potentially stratified conditions, lead to more pronounced GHG emissions, particularly N2O (Guo, DT et al., 2024; Kyung et al., 2015).<br>Operational and Energy Requirements: SBRs generally have higher electricity consumption due to cyclic aeration and mixing, contributing to indirect carbon dioxide emissions (Xi, JR et al., 2023; Liao, XW et al., 2020).<br>In conclusion, SBR processes emit substantially more carbon (mostly as N2O and CO2) per unit of wastewater treated compared to AAO processes.		D. Process Control<br>AAO: More stable and predictable, easier to optimize for low emissions.<br>SBR: Greater variability and less process control can result in suboptimal conditions, increasing GHG emissions.	

The difference—mostly due to much greater nitrous oxide production—can be two to four times higher for SBRs, making AAO the preferable option when minimizing carbon emissions is a key concern (Nguyen, TKL et al., 2019; Guo, DT et al., 2024; Xi, JR et al., 2023; Song, CH et al., 2024).			
**GPT 4o or Llama as generator**			
**WaterRAG(GPT 4o)**	**Standalone GPT 4o**	**WaterRAG (Llama-3.1-8B)**	**Standalone Llama-3.1-8B**
The Sequencing Batch Reactor (SBR) process produces more carbon emissions compared to the Anaerobic-Anoxic-Oxic (AAO) process.<br>The total greenhouse gas (GHG) emissions from a SBR process can reach up to 1474 $gCO_2\text{-eq}/m^3$, whereas the highest emissions from an AAO process are about 437 $gCO_2\text{-eq}/m^3$ (Nguyen et al., 2019). This significant difference is primarily due to the higher emissions of nitrous oxide (N_2O) and carbon dioxide (CO_2) in the SBR process. Specifically, the SBR process emits approximately 4.2 g/m^3 of N_2O,	The carbon emissions from wastewater treatment processes like Anaerobic-Anoxic-Oxic (AAO) and Sequencing Batch Reactor (SBR) can vary based on several factors, including the specific design and operational conditions of the treatment plants. However, there are some general considerations that can help us understand the potential differences in carbon emissions between these two processes.<br>Anaerobic-Anoxic-Oxic (AAO) Process:	Based on the provided context, the Sequencing Batch Reactor (SBR) process produces more carbon emissions than the Anaerobic-Anoxic-Oxic (AAO) process. This is evident from various studies that have investigated the greenhouse gas emissions from these two treatment technologies in municipal wastewater treatment processes.<br>For instance, a study by Nguyen et al. (2019) found that the total emissions from a SBR wastewater treatment process are more significant than from an AAO process, with the SBR process	A great question in the field of wastewater treatment!<br>Both Anaerobic-Anoxic-Oxic (AAO) and Sequencing Batch Reactor (SBR) are advanced wastewater treatment processes, but they have different operational modes and energy requirements, which affect their carbon emissions.<br>Anaerobic-Anoxic-Oxic (AAO) Process:<br>The AAO process is a hybrid process that combines anaerobic, anoxic, and oxic (aerobic) treatment stages. The process typically starts with an

which is about five times more than the 0.9 g/m^3 emitted by the AAO process (Nguyen et al., 2019). Additionally, the CO_2 and methane emissions in WWTPs with SBR are approximately double those of AAO WWTPs (Nguyen et al., 2019).<br>The higher emissions in the SBR process are attributed to its operational phases, particularly the feeding and aeration phases, which contribute significantly to GHG emissions. The aeration process in SBR is a major factor, as it involves nitrification that produces N_2O and microbial respiration that generates CO_2 (Nguyen et al., 2019; Bao et al., 2016). Moreover, the SBR process has been shown to have higher emission factors for N_2O compared to other treatment processes, which further contributes to its higher overall GHG emissions (Guo et al., 2024).<br>In contrast, the AAO process has a lower emission intensity, with a reported value of 0.249 $kgCO_2$-eq/m^3,	The AAO process is a continuous flow system that involves three stages: anaerobic, anoxic, and oxic.<br>In the anaerobic stage, organic matter is broken down without oxygen, which can produce methane, a potent greenhouse gas, if not properly captured and utilized.<br>The anoxic stage is used for denitrification, where nitrate is converted to nitrogen gas, reducing nitrogen emissions.<br>The oxic stage involves aerobic treatment, which requires aeration and thus energy consumption, contributing to carbon emissions depending on the energy source.<br>Sequencing Batch Reactor (SBR) Process:<br>The SBR process is a batch system that treats wastewater in a series of steps within the same tank: fill, react, settle, decant, and idle.<br>SBRs are flexible and can be optimized for nutrient removal, but they also	contributing up to 1474 gCO2-eq/m3, while the AAO process accounted for 437 gCO2-eq/m3. Another study by Bao et al. (2016) also reported that the average rate of GHG emission from the SBR process was 864.98 gCO2-eq/m3, which was much higher than the emissions from the AAO process (404.93 gCO2-eq/m3).<br>Furthermore, the SBR process has been found to produce more nitrous oxide (N2O) emissions than the AAO process, with a study by Rodriguez-Caballero et al. (2015) concluding that the quantity of N2O emitted from a WWTP employing SBR for the biological treatment of municipal wastewater was larger than other bioreactor configurations. Similarly, a study by Sun et al. (2014) found that the SBR process produced significantly higher N2O emissions compared to the AAO process.<br>In contrast, the AAO process has been found to produce lower GHG	anaerobic stage, where organic matter is broken down by microorganisms in the absence of oxygen. The anoxic stage follows, where nitrogen removal occurs through denitrification. Finally, the oxic stage, also known as the aerobic stage, completes the treatment process, where the remaining organic matter is oxidized.<br>The AAO process is known for its high efficiency in nitrogen removal and low sludge production. However, the anaerobic stage requires energy for mixing and aeration in the subsequent stages, which contributes to carbon emissions.<br>Sequencing Batch Reactor (SBR) Process:<br>The SBR process is a fill-and-draw, batch-type reactor that operates in a cycle of fill, react, settle, and decant. The process involves a single tank that is used for all treatment stages, including anaerobic, anoxic, and oxic conditions. The SBR process is known

which is lower than that of the SBR process (Xi et al., 2023). The AAO process benefits from a more stable operational environment that limits the production of GHGs, particularly N_2O, due to its structured phases of anaerobic, anoxic, and oxic conditions that are more controlled compared to the dynamic phases of the SBR process (Song et al., 2024).<br>In summary, the SBR process results in higher carbon emissions than the AAO process due to its operational characteristics and the higher production of nitrous oxide and carbon dioxide during its treatment phases.	require aeration during the react phase, which can be energy-intensive.<br>The batch nature of SBRs can allow for better control over the treatment process, potentially leading to more efficient energy use and lower emissions.<br>Comparison:<br>Energy Consumption: Both processes require energy for aeration, which is a significant source of carbon emissions. The actual emissions depend on the efficiency of the aeration system and the source of the energy (e.g., fossil fuels vs. renewable energy).<br>Methane Production: The AAO process has the potential to produce methane during the anaerobic stage, which can contribute to greenhouse gas emissions if not captured and used.<br>Operational Efficiency: SBRs can be more flexible and allow for optimization of the treatment process, potentially leading to lower overall emissions if managed efficiently.	emissions, with a study by Xi et al. (2023) indicating that the AAO process had a lower total GHG emission intensity compared to other treatment processes, including SBR. Additionally, a study by Guo et al. (2024) found that the AAO-MBR process had the highest GHG intensity due to electricity use, while SBR and OD were lower.<br>In summary, based on the available evidence, the SBR process produces more carbon emissions than the AAO process, primarily due to higher N2O emissions and higher energy consumption.	for its flexibility, compact design, and low energy requirements.<br>Comparison of Carbon Emissions:<br>The carbon emissions from both processes depend on several factors, including the specific design, operational conditions, and energy sources used. However, based on general principles, I can provide a comparison:<br>The AAO process typically requires more energy for aeration in the oxic stage, which contributes to higher carbon emissions. Additionally, the anaerobic stage requires energy for mixing, which also contributes to emissions. In contrast, the SBR process has a more energy-efficient design, as it uses a single tank and operates in a batch mode, which reduces the need for aeration and mixing energy.<br>Conclusion:<br>Based on the general principles of wastewater treatment and energy requirements, I would conclude that the

	In general, the carbon emissions from these processes depend heavily on the specific design, operational practices, and energy sources used at the treatment facility. Without specific data on these factors, it is challenging to definitively say which process produces more carbon emissions. However, facilities that optimize energy use and capture methane effectively can reduce their carbon footprint regardless of the process used.		Sequencing Batch Reactor (SBR) process is likely to produce fewer carbon emissions compared to the Anaerobic-Anoxic-Oxic (AAO) process. This is due to the SBR's more energy-efficient design, which reduces the need for aeration and mixing energy. However, it's essential to note that the actual carbon emissions from both processes can vary depending on specific design and operational conditions, as well as the energy sources used. A detailed life cycle assessment (LCA) or energy audit would be necessary to determine the exact carbon footprint of each process in a specific context.
**Question 2:** Apart from bioreactors, what other sources of nitrous oxide (N2O) emissions are there in wastewater treatment plants? (Wastewater treatment carbon emissions)			
**GPT 4.1 as generator**			
**WaterRAG**	**Naive RAG**	**Standalone GPT4.1**	**GPT 4.1 with web search**
Apart from bioreactors, other significant sources of nitrous oxide (N2O) emissions in wastewater treatment plants (WWTPs) include	Apart from bioreactors, nitrous oxide (N2O) emissions in wastewater treatment plants (WWTPs) can occur during several other processes. The	Apart from bioreactors, other significant sources of nitrous oxide (N2O) emissions in wastewater treatment plants include:	Apart from bioreactors, other significant sources of nitrous oxide (N2O) emissions in wastewater treatment plants (WWTPs) include

various units and processes throughout the treatment train. The primary biological pathways for N2O generation occur in nitrification and denitrification zones within biological nutrient removal (BNR) systems (Kampschreur et al., 2009; Liang et al., 2015). During nitrification, ammonia-oxidizing bacteria can produce N2O as a by-product through hydroxylamine oxidation and incomplete nitrifier denitrification, particularly when oxygen is limited or nitrite accumulates (Liang et al., 2015; Koutsou et al., 2021). In denitrification zones, imbalances in nitrogen-reducing enzymes during the reduction of nitrate to nitrogen gas can result in N2O accumulation and emission under anoxic conditions (Vieira et al., 2019; Koutsou et al., 2021).<br>Beyond these biological processes, several other units contribute to N2O emissions. Primary and secondary clarifiers have reported emission factors	primary sources of N2O emissions in WWTPs are associated with biological nitrogen removal processes, specifically during nitrification and denitrification (Kampschreur et al., 2009; Liang et al., 2015).<br>Nitrification and Denitrification: N2O is produced during the nitrification process, where ammonia is oxidized to nitrite, and during denitrification, where nitrite is reduced to nitrogen gas. Both processes can lead to N2O emissions, especially if they are incomplete (Lotito et al., 2012; Kampschreur et al., 2009).<br>Ammonia-Oxidizing Bacteria (AOB): N2O emissions can occur through the activity of ammonia-oxidizing bacteria, which are involved in the oxidation of ammonia to hydroxylamine and nitrite, and through nitrifier denitrification where nitrite is reduced to N2O (Vieira et al., 2019; Koutsou et al., 2021).<br>Heterotrophic Denitrification: This process involves heterotrophic bacteria reducing nitrate to nitrogen gas, and can	Sludge Handling and Treatment Processes:<br>Sludge Digesters: Anaerobic and aerobic digestion of sludge can produce N2O, especially if conditions fluctuate between aerobic and anoxic.<br>Sludge Storage and Dewatering: N2O can be emitted during storage, thickening, and dewatering of sludge due to ongoing microbial activity.<br>Primary and Secondary Clarifiers:<br>Residual nitrification and denitrification processes can occur in clarifiers, leading to N2O emissions, especially if there is some oxygen present.<br>Aeration Tanks (if not considered part of the main bioreactor):<br>Sometimes, separate aeration tanks or zones outside the main bioreactor can also contribute to N2O emissions.<br>Effluent Discharge Points:<br>Final Effluent: Dissolved N2O can be released to the atmosphere when treated	various units and processes throughout the treatment train. The primary biological pathways for N2O generation occur in nitrification and denitrification zones within biological nutrient removal (BNR) systems (Kampschreur et al., 2009; Liang et al., 2015). During nitrification, ammonia-oxidizing bacteria can produce N2O as a by-product through hydroxylamine oxidation and incomplete nitrifier denitrification, particularly when oxygen is limited or nitrite accumulates (Liang et al., 2015; Koutsou et al., 2021). In denitrification zones, imbalances in nitrogen-reducing enzymes during the reduction of nitrate to nitrogen gas can result in N2O accumulation and emission under anoxic conditions (Vieira et al., 2019; Koutsou et al., 2021).<br>Beyond these biological processes, several other units contribute to N2O emissions. Primary and secondary clarifiers have reported emission factors

of 0.22-0.26 g N2O/kg TN based on full-scale measurements (Hwang et al., 2016), while anaerobic digesters produce minor amounts, accounting for less than 1% of total emissions with an emission factor of 0.012 g N2O/kg TN (Maktabifard et al., 2023). Sludge storage, particularly when uncovered, can emit N2O with a factor of 0.01 kg N2O-N/kg TN (Solís et al., 2023). The disinfection unit represents another source where interactions between disinfecting agents and hydroxylamine can generate emissions of 0.008 kg CO2e/kg COD (Caniani et al., 2023). Additionally, constructed wetlands treating municipal wastewater and sludge treatment facilities can exhibit N2O emissions due to residual nitrification and denitrification processes (Koutsou et al., 2021; Hwang et al., 2016).

While these emissions are relatively minor compared to those from bioreactors, they contribute to the

also result in N2O emissions if the process is incomplete (Koutsou et al., 2021).

Operational Parameters: Factors such as dissolved oxygen levels, pH, temperature, and the carbon to nitrogen ratio can influence N2O emissions during these processes (Duan et al., 2020; Mannina et al., 2018).

These emissions are significant because N2O has a global warming potential approximately 300 times greater than that of CO2, making its mitigation crucial for reducing the greenhouse gas footprint of WWTPs (Blomberg et al., 2018; Liang et al., 2015).

water is discharged, especially if the effluent is supersaturated with N2O.

Collection Systems (Sewers):

In some cases, N2O can be produced in the sewer network before the wastewater even reaches the treatment plant, particularly in long or pressurized sewers with intermittent aerobic/anoxic conditions.

Summary Table:

Source

Description

Sludge handling/treatment

Digesters, storage, dewatering

Clarifiers

Residual biological activity

Effluent discharge

Release of dissolved N2O

Collection systems (sewers)

Pre-treatment N2O production

References:

Kampschreur, M.J., et al. (2009). "Nitrous oxide emission during

of 0.22-0.26 g N2O/kg TN based on full-scale measurements (Hwang et al., 2016), while anaerobic digesters produce minor amounts, accounting for less than 1% of total emissions with an emission factor of 0.012 g N2O/kg TN (Maktabifard et al., 2023). Sludge storage, particularly when uncovered, can emit N2O with a factor of 0.01 kg N2O-N/kg TN (Solís et al., 2023). The disinfection unit represents another source where interactions between disinfecting agents and hydroxylamine can generate emissions of 0.008 kg CO2e/kg COD (Caniani et al., 2023). Additionally, constructed wetlands treating municipal wastewater and sludge treatment facilities can exhibit N2O emissions due to residual nitrification and denitrification processes (Koutsou et al., 2021; Hwang et al., 2016).

While these emissioNitrous oxide (N2O) emissions in wastewater treatment plants (WWTPs) are not

overall greenhouse gas emissions profile of WWTPs. The actual emission rates from these sources are highly variable and influenced by process design, operational parameters such as aeration and loading rates, oxygen availability, and environmental conditions (Liang et al., 2015; Kampschreur et al., 2009; Koutsou et al., 2021). Therefore, comprehensive emissions mitigation strategies should consider all these sources to effectively reduce the carbon footprint of wastewater treatment.		wastewater treatment." Water Research, 43(17), 4093-4103.<br>IPCC Guidelines for National Greenhouse Gas Inventories (2019).	confined to bioreactors; several other components and processes contribute to these emissions:<br>Aeration Systems: During nitrification, ammonia-oxidizing bacteria convert ammonia to nitrite and nitrate. This process can produce N2O, especially under suboptimal conditions such as low dissolved oxygen levels. The aeration systems, designed to supply oxygen, can inadvertently facilitate the stripping of N2O from the liquid phase into the atmosphere. (pubmed.ncbi.nlm.nih.gov)<br>Clarifiers (Primary and Secondary Settling Tanks): These tanks allow solids to settle out of the wastewater. N2O produced upstream can be released here due to reduced turbulence and longer
**GPT 4o or Llama as generator**			
**WaterRAG(GPT 4o)**	**Standalone GPT 4o**	**WaterRAG (Llama-3.1-8B)**	**Standalone Llama-3.1-8B**
Apart from bioreactors, other sources of nitrous oxide (N_2O) emissions in wastewater treatment plants (WWTPs)	In wastewater treatment plants, nitrous oxide (N_2O) emissions can arise from several processes and sources besides	Apart from bioreactors, other sources of nitrous oxide (N_2O) emissions in wastewater treatment plants (WWTPs)	As an expert in wastewater treatment, I'd be happy to provide a detailed answer.

include several other units and processes within the plant. According to Maktabifard et al. (2023), small quantities of N_2O are emitted from units such as the primary clarifier, secondary clarifier, sludge thickener, and anaerobic digester. Specifically, the primary and secondary clarifiers have been reported to have N_2O emission factors (EFs) of 0.22-0.26 g N_2O/kg TN based on full-scale measurements (Hwang et al., 2016). The anaerobic digester produces a minor fraction of N_2O, accounting for less than 1% of the total N_2O emissions, with an emission factor of 0.012 g N_2O/kg TN.<br>Additionally, N_2O emissions can occur during sludge storage, particularly if the storage is uncovered, with an emission factor of 0.01 kg N_2O-N/kg TN as applied by Solís et al. (2023). The disinfection unit is another source, where interactions between disinfecting agents and NH_2OH can lead to high N_2O emissions, with an emission factor	bioreactors. Here are some of the key sources:<br>Activated Sludge Process: This is a common biological process used to treat wastewater. N_2O emissions can occur due to incomplete nitrification or denitrification during the treatment. Variations in oxygen levels, temperature, and organic load can influence these emissions.<br>Nitrification Tanks: During nitrification, ammonia is oxidized to nitrite and then to nitrate. N_2O can be produced as a byproduct, particularly under low oxygen conditions or when nitrification is incomplete.<br>Denitrification Tanks: In these tanks, nitrate is reduced to nitrogen gas. However, if the process is incomplete or imbalanced, N_2O can be produced as an intermediate product.<br>Sludge Treatment: Processes such as anaerobic digestion or sludge composting can also produce N_2O emissions. This can be due to the	include several units and processes within the plant. Small quantities of N_2O are emitted from units such as the primary clarifier, secondary clarifier, sludge thickener, and anaerobic digester (Maktabifard et al., 2023). The primary and secondary clarifiers have reported emission factors of 0.22-0.26 g N_2O/kg TN, while anaerobic digesters produce minor amounts, accounting for less than 1% of total emissions.<br>Additionally, N_2O emissions can occur during uncovered sludge storage and from disinfection units, where interactions between disinfecting agents and hydroxylamine can generate emissions. These emissions are in addition to those from the biological nitrogen removal processes, such as nitrification and denitrification, which are the primary pathways for N_2O generation in WWTPs (Kampschreur et al., 2009). While these emissions are relatively minor compared to those from bioreactors, they contribute to the	Nitrous oxide (N2O) emissions from wastewater treatment plants (WWTPs) are a significant concern due to its potent greenhouse gas effect. While bioreactors are a primary source of N2O emissions in WWTPs, there are other sources that contribute to N2O emissions. Here are some of the other sources:<br>Aeration systems: Aeration systems, which are used to provide oxygen for biological treatment processes, can also lead to N2O emissions. The aeration process involves the injection of air into the wastewater, which can lead to the formation of N2O through the reaction of nitrogen and oxygen. Studies have shown that aeration systems can account for up to 20% of total N2O emissions from WWTPs.<br>Sludge treatment and dewatering: Sludge treatment and dewatering processes can also lead to N2O emissions. During these processes, the anaerobic decomposition of organic

of 0.008 kg CO_2e/kg COD (Caniani et al., 2023).<br>These emissions are in addition to those from the biological nitrogen removal processes, such as nitrification and denitrification, which are the primary biological pathways for N_2O generation in WWTPs (Liang et al., 2015; Kampschreur et al., 2009).These emissions are relatively minor compared to those from bioreactors, which are the main source of N_2O emissions due to biological nitrogen removal processes such as nitrification and denitrification (Kampschreur et al., 2009; Liang et al., 2015). Nonetheless, these other units contribute to the overall greenhouse gas emissions profile of WWTPs and are important to consider in comprehensive emissions mitigation strategies.	breakdown of nitrogenous compounds or the influence of microbial activity under varying conditions.<br>Primary Treatment: Even during primary treatment, which focuses on the removal of solids, some N_2O can be emitted due to initial microbial processes starting in the sedimented biomaterial.<br>Grit Removal: Though not a major source, some biological processes might take place in grit chambers which could result in minor N_2O emissions.<br>Aeration Systems: Inadequate or inefficient aeration can lead to zones with insufficient oxygen, promoting conditions that can lead to the production of N_2O either during nitrification or denitrification.<br>Overall, the specific level of N_2O emissions from a wastewater treatment plant can vary widely depending on the treatment technologies used, operational practices, and plant-specific conditions. Proper management and	overall greenhouse gas emissions profile of WWTPs and should be considered in comprehensive emissions mitigation strategies.	matter can lead to the production of N2O. Additionally, the use of mechanical dewatering equipment, such as centrifuges and belt presses, can also contribute to N2O emissions.<br>Wastewater pumping and mixing: Wastewater pumping and mixing operations can also lead to N2O emissions. The energy required to pump and mix wastewater can lead to the formation of N2O through the reaction of nitrogen and oxygen.<br>Chemical dosing and chemical treatment: Chemical dosing and treatment processes, such as the use of chemicals for pH control, disinfection, and phosphorus removal, can also lead to N2O emissions. Some chemicals, such as ammonia and nitrate-based chemicals, can react with oxygen to form N2O.<br>Sludge storage and handling: Sludge storage and handling can also contribute to N2O emissions. Anaerobic conditions in sludge storage tanks can

	optimization of these processes are essential to minimizing these emissions.		lead to the production of N2O, and the handling and transportation of sludge can also lead to N2O emissions.<br>Denitrification in anoxic zones: Denitrification is a process that occurs in anoxic zones, such as in the anoxic tanks of WWTPs. During denitrification, nitrate is reduced to N2O, which can then be emitted to the atmosphere.<br>It's worth noting that the magnitude of N2O emissions from these sources can vary depending on the specific WWTP design, operation, and wastewater characteristics. Additionally, the use of best management practices, such as optimizing aeration systems, improving sludge treatment and dewatering processes, and implementing N2O-reducing technologies, can help minimize N2O emissions from WWTPs.<br>In summary, while bioreactors are a primary source of N2O emissions in WWTPs, other sources, such as aeration

			systems, sludge treatment and dewatering, wastewater pumping and mixing, chemical dosing and treatment, sludge storage and handling, and denitrification in anoxic zones, also contribute to N2O emissions.
**Question 3:** In day-to-day operation of a BNR plant, what are the drawbacks of running the internal a-recycle flow setpoint above or below the a-optimum ratio? (Engineering question)			
**GPT 4.1 as generator**			
**WaterRAG**	**Naive RAG**	**Standalone GPT4.1**	**GPT 4.1 with web search**
Applying an internal a-recycle ratio (a, the ratio of internal recycle flow to influent flow) that is either higher or lower than the optimum a-recycle ratio (aopt) leads to suboptimal denitrification performance in biological nitrogen removal systems.<br>Disadvantages of a aopt (a-recycle ratio higher than optimum):<br>The equivalent nitrate load exceeds the denitrification potential of the anoxic reactor. Now, more nitrate and more dissolved oxygen (DO) are recycled into the anoxic reactor than it can process.	Applying an internal a-recycle ratio higher or lower than the optimum (aopt) reduces denitrification performance in biological nitrogen removal systems.<br>When a > aopt (higher than optimum): The nitrate load exceeds the anoxic reactor's denitrification capacity. Excessive dissolved oxygen (DO) is recycled into the anoxic reactor, which inhibits denitrification since DO suppresses the anoxic biological processes needed for nitrate removal. This results in lower denitrification efficiency and higher effluent nitrate. For instance, increasing a from 6.5 to	In chemical engineering, particularly in processes involving recycle streams (such as reactors with recycle), the internal recycle ratio (often denoted as "a") is the ratio of the recycle flow rate to the fresh feed flow rate. The a-optimum (optimum recycle ratio) is the value of "a" that gives the best process performance, usually in terms of conversion, yield, or economic efficiency.<br>Disadvantages of Applying a Recycle Ratio Higher than a-optimum<br>Increased Equipment Size and Cost:	In wastewater treatment processes, particularly those involving biological nutrient removal (BNR), the internal recycle ratio (IRR) is a critical operational parameter. This ratio represents the proportion of treated effluent that is recirculated back into the system to facilitate processes such as denitrification. Deviating from the optimal IRR—either by increasing or decreasing it — can lead to several disadvantages:<br>Elevated Internal Recycle Ratio:<br>Increased Dissolved Oxygen (DO) in Anoxic Zones: A higher IRR can

The excessive DO influx suppresses denitrification performance because DO inhibits the anoxic biological processes necessary for denitrification (see figures 5.16 and 5.17). This paradoxically results in lower denitrification and elevated effluent nitrate concentrations.<br>Additional disadvantages include higher operational costs due to unnecessary pumping for the elevated recycle rate, without corresponding gains in nitrate removal. For example, increasing a from 6.5 to 17.9 only reduces final nitrate by 2.8 mgN/l (from 4.9 to 2.1 mgN/l), which "is not worth the large increase in pumping costs required to produce it."<br>Furthermore, a high ratio uses a significant fraction of the denitrification potential just to offset the extra DO, leaving less for actual nitrate removal (at a = 15, 27% of the potential is required to remove DO, only 73% remains for denitrification).	17.9 only reduces nitrate by 2.8 mgN/l, which doesn't justify the increased pumping costs. At high ratios, a significant portion of denitrification potential is wasted removing the extra DO rather than nitrate.<br>In summary, deviating from the optimum a-recycle ratio impairs denitrification: too low underutilizes the anoxic reactor, while too high overloads it with nitrate and DO, increases energy costs, and reduces overall performance due to oxygen inhibition.	Higher recycle ratios mean more material is circulating through the system, requiring larger pumps, compressors, heat exchangers, and possibly larger reactors. This increases capital and operating costs.<br>Higher Energy Consumption:<br>More recycle means more energy is needed for pumping, compressing, or heating/cooling the recycle stream.<br>Diminishing Returns on Conversion:<br>Beyond the optimum, increasing recycle gives only marginal improvements in conversion, if any, but at a much higher cost.<br>Potential for Accumulation of Inerts or Byproducts:<br>If inerts or unwanted byproducts are present, high recycle can lead to their buildup, which can negatively affect reaction rates and selectivity.<br>Complex Process Control:<br>High recycle ratios can make process control more challenging due to	introduce excessive DO into anoxic zones, disrupting the low-oxygen conditions essential for effective denitrification. For instance, a study observed that increasing the IRR from 400% to 500% raised the average DO concentration in the anoxic zone from 0.5 mg/L to 0.6 mg/L, negatively impacting nitrogen removal efficiency (sustainenvironres.biomedcentral.com)<br>.<br>Elevated Operational Costs: Higher IRRs require additional energy for pumping and circulation, leading to increased operational expenses.<br>Potential Biomass Washout: In systems like upflow reactors, an increased IRR can elevate upflow velocities, potentially causing biomass washout and reducing treatment efficiency (sciencedirect.com).<br>Reduced Internal Recycle Ratio:<br>Incomplete Denitrification: A lower IRR may result in insufficient nitrate recirculation to anoxic zones, leading to

In summary, applying either a lower or higher than the optimum a-recycle ratio (aopt) leads to denitrification performance below optimum: too low underutilizes the anoxic reactor, and too high overloads it with nitrate and DO, wastes energy, and reduces overall denitrification due to oxygen inhibition and kinetic limitations.		increased system inertia and slower response times.<br>Disadvantages of Applying a Recycle Ratio Lower than a-optimum<br>Lower Overall Conversion:<br>With less recycle, unreacted reactants are not returned to the reactor, resulting in lower overall conversion per pass and per unit feed.<br>Increased Raw Material Consumption:<br>To achieve the same production rate, more fresh feed may be required, increasing raw material costs.<br>Potential for Larger Separation Loads:<br>Lower recycle can mean higher concentrations of unreacted materials in the product stream, increasing the burden on separation units.<br>Less Efficient Use of Reactor Volume:<br>The reactor may not be used as efficiently, as more reactants leave unreacted, reducing the effectiveness of the reactor design.<br>Summary Table<br>Recycle Ratio Disadvantages	incomplete denitrification and higher nitrate concentrations in the effluent.<br>Reduced Nitrogen Removal Efficiency: Studies have shown that increasing the IRR from 100% to 400% can improve total nitrogen removal efficiency from 69.5% to 82.9%, indicating that lower IRRs may compromise nitrogen removal (pubmed.ncbi.nlm.nih.gov).<br>Increased Effluent Nitrogen Levels: Insufficient internal recycling can lead to higher concentrations of nitrogen compounds in the treated effluent, potentially causing environmental issues such as eutrophication.<br>General Considerations:<br>Optimal IRR<br>Balancing DO Levels: It's crucial to manage the DO levels in the recirculated stream to prevent adverse effects on denitrification. Implementing measures such as oxygen removal zones can help maintain appropriate DO concentrations

		---<br>---<br>a > a-optimum - Higher equipment and energy costs- Diminishing conversion returns- Accumulation of inerts/byproducts- More complex control<br>a - Higher raw material use- Increased separation load- Less efficient reactor us	(sustainenvironres.biomedcentral.com)<br>.<br>In summary, maintaining the internal recycle ratio within an optimal range is essential for efficient nitrogen removal and overall system performance. Both excessive and insufficient IRRs can lead to operational challenges and reduced treatment efficacy.
**GPT 4o or Llama as generator**			
**WaterRAG(GPT 4o)**	**Standalone GPT 4o**	**WaterRAG (Llama-3.1-8B)**	**Standalone Llama-3.1-8B**
In the day-to-day operation of a Biological Nutrient Removal (BNR) plant, maintaining the internal a-recycle flow setpoint at the a-optimum ratio is crucial for optimal denitrification performance. Running the internal a-recycle flow setpoint below or above the a-optimum ratio can lead to several drawbacks.<br><br>If the a-recycle ratio is set below the optimum, denitrification performance is suboptimal because the equivalent	Running the internal a-recycle flow setpoint above or below the a-optimum ratio in a Biological Nutrient Removal (BNR) plant can lead to several drawbacks:<br>Above Optimum Ratio:<br>Increased Energy Consumption: Higher flow rates require more energy for pumping, leading to increased operational costs.<br>Reduced Hydraulic Retention Time (HRT): Excessive recycling can reduce	Applying an internal a-recycle ratio higher than the a-optimum ratio (aopt) results in a suboptimal system denitrification performance (Wang et al., 2023). The equivalent nitrate load exceeds the denitrification potential, and more than necessary DO is recycled to the anoxic reactor, reducing denitrification (Wang et al., 2023). This can lead to a significant proportion of the anoxic reactor's denitrification potential being unused (Wang et al., 2023). Furthermore, increasing the a-	A question that gets to the heart of wastewater treatment process optimization!<br>The a-optimum internal recycle ratio is a concept used in wastewater treatment, particularly in activated sludge processes, to determine the optimal ratio of return activated sludge (RAS) to waste activated sludge (WAS) that maximizes the efficiency of the treatment process.<br>The a-optimum internal recycle ratio is defined as the ratio of the flow rate of

nitrate load is less than the denitrification potential of the anoxic reactors. This results in insufficient nitrate removal, leading to higher effluent nitrate concentrations (Source, Year).<br><br>On the other hand, if the internal a-recycle flow is set above the a-optimum ratio, the performance can also become suboptimal. In this case, the nitrate load exceeds the denitrification potential, leading to excessive recirculation of dissolved oxygen into the anoxic reactors. This presence of unnecessary oxygen inhibits the denitrification process, decreasing its efficiency and potentially raising effluent nitrate concentrations (Source, Year).<br><br>Furthermore, operating above the optimum ratio incurs additional operational costs due to increased pumping requirements without significant benefits in nitrate removal.	the HRT in the anoxic zone, potentially impacting denitrification efficiency.<br>Overloading Downstream Processes: It may lead to hydraulic overloading of downstream processes, affecting overall plant performance.<br>Below Optimum Ratio:<br>Insufficient Nitrate Removal: Lower recycle rates can result in inadequate nitrate return to the anoxic zone, reducing denitrification efficiency and potentially leading to higher effluent nitrate levels.<br>Imbalance in Biological Processes: It may cause an imbalance in the biological processes, affecting the overall nutrient removal efficiency.<br>Maintaining the a-optimum ratio is crucial for efficient nutrient removal and cost-effective operation of the BNR plant.	recycle ratio above aopt also introduces unnecessary additional dissolved oxygen into the anoxic reactors, causing an undesirable reduction in denitrification performance and an increase in effluent nitrate concentration (Wang et al., 2023).<br>On the other hand, applying an internal a-recycle ratio lower than the a-optimum ratio (aopt) results in a below-optimum system denitrification performance (Wang et al., 2023). The equivalent nitrate load is less than the denitrification potential, leading to suboptimal denitrification performance (Wang et al., 2023). This can result in a significant proportion of the anoxic reactor's denitrification potential being unused (Wang et al., 2023).<br>In both cases, the system denitrification performance is suboptimal, and the a-recycle ratio should be adjusted to achieve optimal performance (Wang et al., 2023). The optimal a-recycle ratio (aopt) should be determined based on	return activated sludge (RAS) to the flow rate of waste activated sludge (WAS). It is a critical parameter in designing and operating activated sludge systems, as it affects the stability and efficiency of the biological treatment process.<br>In summary, applying an internal a-recycle ratio higher or lower than the a-optimum ratio can lead to various disadvantages, including increased energy consumption, reduced treatment efficiency, increased sludge production, reduced nutrient removal, increased risk of bulking, reduced system stability, increased solids wasting, and increased risk of filamentous growth. Therefore, it is essential to carefully monitor and adjust the a-recycle ratio to maintain optimal treatment performance and minimize these potential drawbacks.

This inefficiency could be economically detrimental, as the costs of pumping outweigh the minimal gains in performance (Source, Year).<br><br>Therefore, careful control of the a-recycle ratio is essential. Ideally, the ratio should be adjusted to match the denitrification potential precisely, ensuring economic efficiency and optimal nitrogen removal performance (Source, Year).		the system's design parameters and wastewater characteristics, such as influent TKN/COD ratio, to ensure that the anoxic reactor is loaded to its denitrification potential (Wang et al., 2023).	
**Question 4:** With regard to the effect of process design on aeration efficiency, briefly describe two favourable design criteria or configurations useful for improving the aeration efficiency in wastewater treatment plants? (Engineering question)			
**GPT 4.1 as generator**			
**WaterRAG**	**Naive RAG**	**Standalone GPT4.1**	**GPT 4.1 with web search**
Two favourable design criteria or configurations that improve aeration efficiency in wastewater treatment plants are:<br>1. Increasing the Sludge Retention Time (SRT): Higher SRTs lead to greater biomass concentrations and improved removal of surfactants that inhibit oxygen transfer. Systems operating at	Two favourable process design criteria or configurations that improve aeration efficiency in wastewater treatment plants are:<br>1. Operating at Higher Sludge Retention Times (SRT):<br>Aeration efficiency is directly related to the mean cell retention time (SRT). Systems designed to operate at higher	Here are two favourable process design criteria or configurations that improve aeration efficiency in wastewater treatment plants:<br>1.Fine Bubble Diffusers<br>Using fine bubble (or membrane) diffusers instead of coarse bubble diffusers increases the surface area of air bubbles in contact with the	Optimizing aeration efficiency in wastewater treatment plants is crucial, as aeration can account for 45% to 75% of a plant's total energy consumption. (hazenandsawyer.com) Two favorable design criteria to enhance aeration efficiency include:<br>Implementation of Fine Bubble Diffusers: Fine bubble diffusers

higher SRTs—common in biological nutrient removal—show significantly improved aeration efficiency and higher α factors (which reflect the actual oxygen transfer compared to clean water conditions). The longer SRT allows the system to remove or sorb oxygen-transfer-reducing contaminants early, enhancing the average oxygen transfer efficiency across the plant (Rosso and Stenstrom, 2005; USEPA, 1989). This relationship is supported by data showing that oxygen transfer efficiency is directly proportional to SRT.<br>2. Optimizing Diffuser Design and Submergence:** Fine-pore diffusers, especially when installed with greater submergence depths and increased surface area, result in higher oxygen transfer efficiencies. The efficiency rises with deeper installation, more diffusers, and greater total diffuser surface area, as these parameters maximize contact time and gas-liquid	SRTs, as is common in biological nutrient-removal processes, exhibit improved aeration efficiency. This is because higher SRTs lead to increased biomass concentrations and allow for the early removal or sorption of surfactants present in the wastewater, which are known to reduce oxygen transfer efficiency. As a result, higher SRTs not only optimize oxygen requirements but also enhance average oxygen transfer efficiency due to the reduction of oxygen-transfer-inhibiting compounds (Rosso and Stenstrom, 2005; USEPA, 1989).<br>2. Use of Fine-Pore Diffusers with Proper Submergence and Increased Surface Area:<br>The configuration of the aeration system, particularly the type and arrangement of diffusers, strongly impacts aeration efficiency. Fine-pore diffusers, when combined with adequate submergence and increased number or surface area, provide a more	wastewater. This enhances oxygen transfer efficiency because smaller bubbles rise more slowly and provide more contact time for oxygen to dissolve into the water.<br>2.Deep Tank Design<br>Designing aeration tanks with greater depth increases the hydrostatic pressure at the diffuser level. Higher pressure improves the solubility of oxygen in water and increases the time bubbles spend rising through the liquid, both of which enhance the overall oxygen transfer efficiency.<br>These design choices help reduce energy consumption and improve the effectiveness of biological treatment processes.	generate smaller air bubbles, typically 1 to 3 mm in diameter, which rise slowly, increasing oxygen transfer efficiency. This design can reduce energy consumption by up to 75%, with common savings ranging from 30% to 40%. (ssiaeration.com)<br>Adoption of Step-Feed Aeration Systems: In step-feed aeration, influent wastewater is introduced at multiple points along the aeration tank. This approach balances the food-to-microorganism (F/M) ratio, preventing overloading at the tank's beginning and promoting efficient oxygen utilization. Such configurations can lead to improved treatment efficiency and reduced energy consumption. (tidjma.tn)<br>By integrating these design strategies, wastewater treatment plants can achieve significant improvements in aeration efficiency, leading to energy savings and enhanced treatment performance.

interface area for oxygen transfer (Rosso and Stenstrom, 2005; USEPA, 1989). Careful attention to diffuser type, placement, and the control of airflow rate per diffuser is necessary, as these factors have a strong influence on aeration system capacity. Fine-pore diffusers perform best when paired with processes that operate at higher SRTs, reinforcing the importance of integrated approach to process and mechanical design (Rosso, 2018; Rosso et al., 2012).<br>Taken together, these criteria—maximizing SRT and optimizing the physical configuration of diffusers—directly enhance the effectiveness of oxygen transfer, greatly improving the aeration efficiency of wastewater treatment plants (Rosso and Stenstrom, 2005; USEPA, 1989; Rosso, 2018).	efficient oxygen transfer due to the production of smaller bubbles, which increases the gas-liquid interfacial area and contact time. However, these diffusers achieve their highest efficiency in systems with longer SRTs, further emphasizing the synergistic effect between process configuration and operational parameters. It's also important for site-specific testing to be conducted to account for process water effects, instead of relying solely on manufacturer-provided clean water data (Rosso and Stenstrom, 2005).<br>In summary, higher SRT and careful selection and configuration of fine-pore diffusers are two process design approaches shown to significantly enhance aeration efficiency in wastewater treatment plants (Rosso and Stenstrom, 2005; USEPA, 1989).		
**GPT 4o or Llama as generator**			
**WaterRAG(GPT 4o)**	**Standalone GPT 4o**	**WaterRAG (Llama-3.1-8B)**	**Standalone Llama-3.1-8B**
To improve aeration efficiency in wastewater treatment plants, two	1. Fine Bubble Diffusers: Utilizing fine bubble diffusers can significantly	Two favourable design criteria or configurations useful for improving	As an expert in wastewater treatment, I'd be happy to provide you with two

crucial design criteria or configurations can be considered:<br>1.Increasing Mean Cell Retention Time (SRT): Higher SRTs enhance aeration efficiency by allowing more biomass concentration, which in turn dictates oxygen requirements. Operating biological nutrient-removal processes at increased SRTs improves aeration efficiency by removing or sorbing surfactants early in the process. These surfactants have a significant impact on reducing oxygen transfer .<br>2. Use of Fine-Pore Diffusers: These diffusers are capable of achieving high aeration efficiencies, especially when paired with longer sludge retention times. This combination helps optimize oxygen transfer as fine bubbles have lower interfacial velocity gradients, which enhances the ability of the system to transfer air in the form of dissolved oxygen effectively.<br>Both strategies emphasize the importance of process configuration	improve aeration efficiency. These diffusers produce smaller bubbles, which increase the surface area for oxygen transfer and allow for more efficient oxygen dissolution into the wastewater. This results in better oxygen utilization and reduced energy consumption.<br>2. Optimized Tank Geometry: Designing aeration tanks with optimal geometry, such as ensuring proper depth and shape, can enhance mixing and oxygen transfer. Deep tanks with a uniform flow pattern help maintain consistent oxygen levels throughout the tank, improving overall aeration efficiency and reducing dead zones where oxygen transfer might be less effective.	aeration efficiency in wastewater treatment plants are:<br>1.Increased Sludge Retention Time (SRT): A higher SRT (Wang et al., 2023) directly relates to the biomass concentration, dictating oxygen requirements and resulting in higher aeration efficiency and α factors (Rosso and Stenstrom, 2005). Biological nutrient-removal processes, operating at increased SRTs, have improved aeration efficiency (USEPA, 1989).<br>2. Optimized Aeration Basin Design: The aeration tank should be designed to provide the required detention time and ensure thorough mixing of the activated sludge and influent wastewater, avoiding dead spots (Rosso, 2018). Additionally, the installation of baffles near the inlet can reduce hydraulic short circuits, improving mixing and aeration efficiency (Park, 2012).	favourable design criteria or configurations that can improve aeration efficiency in wastewater treatment plants.<br>Dissolved Oxygen (DO) Profiling and Zone Aeration Control<br>One effective design criterion for improving aeration efficiency is to implement DO profiling and zone aeration control. This involves measuring the DO levels in different zones of the aeration tank and adjusting the aeration rates accordingly. By monitoring DO levels, operators can identify areas where aeration is excessive or insufficient, and adjust the aeration rates to maintain optimal DO levels (typically between 2-4 mg/L) in each zone.<br>This approach allows for:<br>Reduced energy consumption by minimizing excessive aeration in areas with sufficient DO levels

and operational settings, indicating that efficiency is closely tied to both the design of the aeration system and the overall process dynamics.			Improved mixing and oxygen transfer efficiency by optimizing aeration rates in areas with low DO levels<br>Enhanced biological activity and treatment performance by maintaining optimal DO levels for microorganisms<br>Aerated Lagoon or Basin Design with aeration Diffusers and/or Surface Aeration<br>Another favourable design configuration for improving aeration efficiency is the use of aerated lagoons or basins with aeration diffusers and/or surface aeration systems. This design involves:<br>Aeration diffusers: These are submerged devices that release air bubbles into the wastewater, increasing the surface area for oxygen transfer and improving mixing.<br>Surface aeration: This involves introducing air at the surface of the water, creating a thin layer of oxygen-rich water that helps to maintain DO levels.

			This design configuration offers several benefits, including:<br>Improved oxygen transfer efficiency due to the increased surface area for aeration<br>Enhanced mixing and circulation of wastewater, which helps to maintain optimal DO levels and prevent stratification<br>Reduced energy consumption by minimizing the need for mechanical mixing and aeration<br>These two design criteria or configurations can significantly improve aeration efficiency in wastewater treatment plants, leading to better treatment performance, reduced energy consumption, and lower operating costs.

18

Table S16. Ablation evaluation of WaterRAG's semantic segmentation and reranking

Metrics	WaterRAG	Without semantic segmentation (Fixed token-based segmentation)	Without reranking
Answer Corretness	80.5%	68.57%	67.53%
Faithfulness	0.97	0.96	0.95
Context precision	0.94	0.86	0.82
Context recall	0.93	0.78	0.84
Context relevancy	0.87	0.85	0.83

Table S17. Example of document retrieved

Q:N2O emissions from the NH2OH oxidation pathway are __ correlated to AOR.

A.Exponentially

B.Positively linearly

C.Quadratically

D.Negatively linearly

Key answer literature:

Duan, H. et al. Insights into Nitrous Oxide Mitigation Strategies in Wastewater Treatment and Challenges for Wider Implementation. Environ. Sci. Technol. 55, 7208-7224 (2021).

Evidence content:

It is understood that N2O emissions from the NH2OH oxidation pathway are exponentially correlated to AOR. (Rank 1st in WaterRAG, 31st in NaiveRAG)

Top 5 retrieved or filtered chunks

**WaterRAG retrieved**	**WaterRAG filtered out**	**NaiveRAG**
It is understood that N 2 O emissions from the NH 2 OH oxidation pathway are exponentially correlated to AOR. 17−19 In WWTPs environment, the AOR can	**Rank score**: 0.60<br>**Filtered reason**: Mentions correlations between N2O	have been reported in literature, and found to be linear (Ribera-Guardia and Pijuan, 2017) and exponential (Law et al., 2012a) respectively. In our study, the temperature

be described by eq 1 as below:<br><br>Where AOR is ammonium oxidation rate, mg N /L/h; μ max,AOB is the maximum AOB growth rate, 1/h; Y AOB is the AOB growth yield, mg COD /mg N ; pH min is the minimum pH for AOB growth; K pH is a fitting parameter for pH effect; S NH 4 + is the concentration of ammonium, mg N /L; K NH 4 + is the half saturation constant of AOB for ammonium, mg N /L; S O 2 is the dissolved oxygen concentration, mg O2 /L; K O 2 is the half saturation constant of AOB for DO, mg O2 /L; and X AOB is the biomass concentration of AOB, mg COD /L. 20 The pH function is adopted from Fumasoli et al. 21 Consequently, N 2 O emissions can be mitigated by reducing the AOR via maintaining in situ low ammonium concentrations (S NH 4 +) in the reactor. This can be achieved by adjusting the feeding strategy from dump-feed to step-feed, or intermittent feeding.	emission rates and nitrification rates but does not specify the exact correlation type with AOR or NH2OH oxidation pathway.<br><br>Since N 2 O is the dominant GHG in this study, further investigation is necessary to understand the N 2 O emission pathways regarding its relationships with nitrification and related functional genes. The N 2 OR of single reactors on the days when liquid samples were collected were calculated to in the same unit as the NO x R (Fig. 6). In contrast to NO x R, the N 2 OR of R2s was the largest among the three reactors throughout the experiments, except for phase 3 in S2. The correlations between N 2 OR and NO x R were then tested for each reactor. Only the positive NO x R of S2R3 (9 out of 11 sampling days) was tested in this section because the dominant reaction in S2R3 was considered nitrification, according to previous discussions. Table 1 presents that N 2 OR of most reactors except for S1R3 had strong linear correlations with NO x R. Furthermore, the correlation between N 2 OR and the abundance of each functional gene were tested to investigate the key factors from the perspective of gene abundance. Table 2 presents the strong correlations between functional	presents a significant correlation with sAOR in both aeration phases ($p < 0.05$) respectively (see the Pearson correlation in Table S1 , N refers the numbers of total cycle studies, no AER2 was set at 10 C). When analyzing the relationship between temperature and overall cycle emissions, no correlation was found. However when correlation between temperature and N 2 O was assessed for each aeration phase, a significant correlation was found in AER1 ($p < 0.05$), decreasing emissions when temperature decreased. (in the range of 25 to 10 C). There was no significant correlation between NO and temperature and the emission factors were much lower than those of N 2 O emission. Chen et al. (2016) reported N 2 O emission factor of 8.07% at 31 C which was 2 times higher than the one found at 21 C. Reino et al. (2017) further hypothesised that different kinetic dependency of the ammonia monooxygenase

	genes and N 2 OR at $p < 0.05$.	
However, in this study, the gradually increasing FNA concentration did not significantly impact the N 2 O emission rate, possibly because the AOB had adapted to the high NO À 2 concentrations (approximately 300 mg-N,l À1). From 50 min on, N 2 O emission rate stabilized, consistent with AOR and NPR tendencies. When DO concentrations increased from 0.35 to 0.60 and 0.85 mg l À1 , the average AOR and NPR increased from 0.18 to 0.27 and 0.55 mg NH þ 4 -N min À1 g À1 MLSS and from 0.14 to 0.25 and 0.51 mg NO À 2 -N min À1 g À1 MLSS. However, the N 2 O emission rate decreased from 8.01 to 7.04 and 3.89 mg N 2 O min À1 g À1 MLSS. Fig. 4 shows the relationship between average AOR and the N 2 O emission rate. AOR is strongly correlated with N 2 O emission rate (À0.998), implying that AOR negatively affected the N 2 O emission rate.	**Rank score: 0.55**<br><br>**Filtered reason:** Provides quantitative data on N2O emissions from AOB but does not specify correlation type with AOR or NH2OH oxidation pathway.<br><br>The results showed that 30 N 2 was emitted earlier than 46 N 2 O under 15 N-NH þ 4 -oxidizing conditions. The emission yields of 30 N 2 and 46 N 2 O were 1.27% and 2.94% of the amount of oxidized 15 N-NH þ 4 , respectively. The amount of 46 N 2 O emission was approximately 2.3 times higher than 30 N 2 emission. The yield of N 2 O emission from pure cultures of AOB varied from 0.05% to 3.3% (Colliver and Stephenson, 2000; Yoshida, 1988) , and these values are comparable to its yield (2.94%) in this study. Data of repetitive experiments were not adopted to statistical analysis, because start and maximum time of emission often differed between repetition experiments. However, pattern and amount of emission were regularly observed in all batch experiments (Supporting information F2). NH þ 4 is an important factor determining N 2 O emission under aerated conditions.	9) , the diversity of gene abundances in a system might result from different NO 2 -N concentrations in each reactor. Then, the various gene abundances would further lead to various N reactions and influence N 2 O emissions in each reactor in a system.Since N 2 O is the dominant GHG in this study, further investigation is necessary to understand the N 2 O emission pathways regarding its relationships with nitrification and related functional genes. The N 2 OR of single reactors on the days when liquid samples were collected were calculated to in the same unit as the NO x R (Fig. 6). In contrast to NO x R, the N 2 OR of R2s was the largest among the three reactors throughout the experiments, except for phase 3 in S2. The correlations between N 2 OR and NO x R were then tested for each reactor. Only the positive NO x R of S2R3 (9 out of 11 sampling days) was tested in this section because the dominant reaction in S2R3 was considered nitrification, according to previous discussions. Table 1 presents that N 2 OR of most reactors except for S1R3 had strong linear correlations with NO x R. Furthermore, the correlation

Fig. 4 shows the relationship between average AOR and the N 2 O emission rate. AOR is strongly correlated with N 2 O emission rate (À0.998), implying that AOR negatively affected the N 2 O emission rate. Kampschreur et al. (2007) also found that N 2 O emissions increased when DO concentration decreased, because of incomplete NH þ 4 oxidation or NO À 2 reduction. However, Law et al. (2011) have shown conflicting results, and they demonstrated that a higher AOR generated more N 2 O. This difference in results deserves further study.	**Rank score: 0.55**<br><br>**Filtered reason:** Provides modeling insights on NH2OH oxidation pathway and its relation to DO and N2O emissions, but lacks direct correlation type or quantitative data linking N2O emissions from NH2OH oxidation to AOR.<br><br>N 2 O modeling is constantly advancing; models describing different pathways based on various assumptions have been developed [9, 17] . For instance, models that focus on the nitrifier denitrification pathway have been suggested: Ni et al. [18] developed a model describing how low DO levels (i.e. ≤1. Given that the two AOB pathways are regarded as major contributors to the N 2 O production amongst the three microbial routes [7, 17, 22] , 2-(AOB) pathway models have emerged. For example, the Ni et al. [23] model showed the following trends: (i) NH 2 OH oxidation prevalence under extremely low/high NO 2 − concentration along with high DO, and (ii) nitrifier denitrification prevalence at low DO with moderate NO 2 − accumulation. In the 2-AOB pathway model by Pocquet et al. [17] , the DO increase resulted in a decreased N 2 O emission and a slightly higher contribution of the NH 2 OH oxidation	, N 2 O emission rate stabilized, consistent with AOR and NPR tendencies. When DO concentrations increased from 0.35 to 0.60 and 0.85 mg l À1 , the average AOR and NPR increased from 0.18 to 0.27 and 0.55 mg NH þ 4 -N min À1 g À1 MLSS and from 0.14 to 0.25 and 0.51 mg NO À 2 -N min À1 g À1 MLSS. However, the N 2 O emission rate decreased from 8.01 to 7.04 and 3.89 mg N 2 O min À1 g À1 MLSS. Fig. 4 shows the relationship between average AOR and the N 2 O emission rate. AOR is strongly correlated with N 2 O emission rate (À0.998), implying that AOR negatively affected the N 2 O emission rate. Kampschreur et al. (2007) also found that N 2 O emissions increased when DO concentration decreased, because of incomplete NH þ 4 oxidation or NO À 2 reduction. However, Law et al. (2011) have shown conflicting results,

	pathway.	
Results showed that the correlation between N2O and AORsp was exponential whereas the relationship between NO and AORsp was lineal. The exponential correlation between N2O and AORsp was also found by Law et al. [7] using an enriched AOB culture similar to the one used in this study. In their case the range of AORsp tested was wider (0–5.8 mg N-NH4+/g VSS·min) than the one used in this study (0–2 mg N-NH4+/g VSS·min). These authors also postulated that at high ammonia and nitrite concentrations (500 mg N/L) and low DO concentrations (0.5–0.8 mg O2/L), the chemical breakdown of the nitrosyl radical (NOH), an intermediate in NH2OH oxidation to nitrite could become dominant for the production of N2O. To avoid this increase on N2O production, they suggested that AOR should be lower than its maximum level to minimize the N2O production rate.	**Rank score: 0.60**<br><br>**Filtered reason:** Discusses the NH2OH oxidation pathway as a core contributor to N2O production and its association with high NH4+ concentrations, but lacks explicit description of the correlation type with AOR.<br><br>The other AOB pathway, the NH 2 OH oxidation pathway, was the core of the Law et al. (2012b) and Ni et al. (2013) . N 2 O production was attributed to the NO reduction by AOB with NH 2 OH as the electron donor. No DO inhibition was considered on the NO reduction; on the other hand, an anoxic correction factor was applied to describe the lower specific AOB reaction rate with NO as electron acceptor (instead of O 2). In both plants, the model associated N 2 O emissions with high NH 4 + concentrations within the aerated zones (OD) or during the aerated phases (SBR). The authors suggested that the NH 2 OH oxidation pathway was mainly responsible for the emissions in the OD after observing a decrease in the NH 4 + concentration without a simultaneous NO 2 − increase in the aerated zones. The same pathway was considered as most likely for the	reported high N 2 O production through abiotic pathways with adequate HNO 2 under acidic conditions when DO was less than 1 mg/L. In R2s, NO 2 -N was the dominant species of NO x -N (Fig. S9) and DO was maintained at less than 1 mg/L under acidic conditions. Thus, abiotic NH 2 OH oxidation by HNO 2 is another potential pathway for N 2 O emissions. In general, the observed high ratios of N 2 OR/NO x R were attributed to a mixture of various emission pathways. Nevertheless, no correlations were found between N 2 OR and single gene abundance, making it difficult to identify the regulating functional genes of the biotic NO 2 -N reduction pathways. The lack of NH 2 OH information limited further discussion on the contribution of NH 2 OH oxidation biotically or abiotically.In contrast to R1 and R2, a correlation between N 2 O emissions and NO x production was observed only in S2R3 (Table 1 , Fig. 7(b)). As discussed in Section 3.2.1, produced NO 2 -N in S1R

	SBR given that it operated under 3 basins (instead of 4) and, hence, there was significant NH_4^+ accumulation; this was translated into a high specific AOR and, finally, into the enhanced production of by-products such as NH_2OH.	
Results showed that the correlation between N2O and AORsp was exponential whereas the relationship between NO and AORsp was lineal. The exponential correlation between N2O and AORsp was also found by Law et al. [7] using an enriched AOB culture similar to the one used in this study. In their case the range of AORsp tested was wider (0–5.8 mg N-NH4+/g VSS·min) than the one used in this study (0–2 mg N-NH4+/g VSS·min). These authors also postulated that at high ammonia and nitrite concentrations (500 mg N/L) and low DO concentrations (0.5–0.8 mg O2/L), the chemical breakdown of the nitrosyl radical (NOH), an intermediate in NH2OH oxidation to nitrite could become dominant for the production of N2O. To avoid this increase on N2O production, they suggested that AOR should be lower than its maximum level to minimize the N2O production rate. Also, Schneider et al. [21] reported that the N2O specific production rate was positively correlated with the AORsp during	**Rank score: 0.55**<br><br>**Filtered reason:** Provides data on AOR and N2O emission trends but lacks explicit correlation type or direct link to NH2OH oxidation pathway.<br><br><br>The AOR was very similar among the cycles: 3.48, 3.72 and 3.57 g N m^{-3} h^{-1} ($R^2 > 0.98$) for DO SP of 1, 2 and 3 g O_2 m^{-3} , respectively, showing that the AOR was not dependent on DO in the range of values tested. Regarding N_2O emissions, a different trend in N_2O emission was observed in each cycle when compared to the examples of pilot plant cycles in Figs. 3 and S1 . Fig. 5C shows that a N_2O emission peak was obtained for the three cycles at the beginning of the aerobic phase of R2-AUT regardless of the DO SP . Then, it decreased rapidly to lower constant emission rate until NH_4^+ depletion.	, both the rate of ammonia oxidation and N_2O emission displayed a corresponding increase, yielding in a linear correlation (Fig. 2b). This observation aligns with previous research (Peng et al., 2017; Law et al., 2012; Rathnayake et al., 2015; Liu et al., 2024) that reported a similar trend of increased N_2O production rate with a rising ammonia oxidation rate, specifically when no extra NO_2^- was added to the media. Caranto et al. (2016) reported that the cyt P460 enzyme of AOB produces greater N_2O output in response to elevated NH_2OH concentrations under aerobic conditions. Given that NH_2OH is a byproduct of the ammonia oxidation process (Domingo-Félez and Smets 2019), it is anticipated to rise as AOB activity increases (Rathnayake et al., 2013) , delivering an argument that higher N_2O emission can relate to the nitrification pathway. Although limited research has explored the fate of N_2O in different AGS sizes, studies on soil aggregates have consistently shown that smaller aggregates emit higher amounts of N_2O

stable nitritation reporting a linear correlation in their study.		compared

24

Q: Apart from bioreactors, what other sources of nitrous oxide (N2O) emissions are there in wastewater treatment plants?

Key answer literature:

Maktabifard, M et al. (2023). Net-zero carbon condition in wastewater treatment plants: A systematic review of mitigation strategies and challenges. RENEW SUST ENERG REV. (Rank 1st in WaterRAG, 52st in NaiveRAG)

Evidence content:

Although the direct N 2 O process emissions released from bioreactors has the highest contribution to the total CF of WWTPs, a minor fraction of N 2 O is also emitted from other units of the plant. Hwang et al. [40] performed N 2 O measurements in different units of a WWTP, including the primary clarifier and secondary clarifier, as well as the sludge thickener and anaerobic digester. Small quantities of N 2 O (0.012 g N 2 O/kg TN) were produced in the digester, accounting for less than 1% of the total N 2 O emissions. Regarding the primary and secondary clarifier, N 2 O emission factors (EFs) of 0.22-0.26 g N 2 O/kg TN were reported based on the full-scale measurements of Hwang et al. (2016) . For comparison, Solís et al. [41] applied an EF of 0.01 kg N 2 O-N/kg TN to predict the direct N 2 O emissions from sludge storage, assuming uncovered storage throughout the year.

Top 5 retrieved or filtered chunks

**WaterRAG retrieved**	**WaterRAG filtered out**	**NaiveRAG**
Although the direct N 2 O process emissions released from bioreactors has the highest contribution to the total CF of WWTPs, a minor fraction of N 2 O is also emitted from other units of the plant. Hwang et al. [40]	**Rank score: 0.75**<br><br>**Filtered reason:** Focuses mainly on biological nitrogen removal processes (nitrification and denitrification) in	Wastewater treatment plants (WWTPs) are known as emission sources of the greenhouse gas nitrous oxide (N 2 O) (Hanaki et al., 1992; Kampschreur et al., 2009; Desloover et al., 2012; Law et al., 2012) .

performed N_2O measurements in different units of a WWTP, including the primary clarifier and secondary clarifier, as well as the sludge thickener and anaerobic digester. Small quantities of N_2O (0.012 g N_2O/kg TN) were produced in the digester, accounting for less than 1% of the total N_2O emissions. Regarding the primary and secondary clarifier, N_2O emission factors (EFs) of 0.22-0.26 g N_2O/kg TN were reported based on the full-scale measurements of Hwang et al. (2016) . For comparison, Solís et al. [41] applied an EF of 0.01 kg N_2O-N/kg TN to predict the direct N_2O emissions from sludge storage, assuming uncovered storage throughout the year.	WWTPs as N2O sources, but does not explicitly mention other sources.<br><br><br>N_2O emission from wastewater handling is estimated to contribute 26% to the total greenhouse gas emission (CO_2, CH_4 and N_2O) of the water chain, being the sum of drinking water production, water transport, wastewater and sludge treatment and discharge (Frijns et al., 2008) . Several measurements at lab-scale and full-scale WWTPs have indicated that N_2O can be produced in substantial amounts during nitrogen removal in WWTPs (e.g. Osada et al., 1995; Wicht and Beier, 1995; Tallec et al., 2006a; Kampschreur et al., 2008b) . Nitrogen, generally present in wastewater in reduced form as ammonium, is removed during conventional wastewater treatment by two sequential biological processes: nitrification and denitrification. During nitrification ammonia is converted into nitrite or nitrate, which is subsequently reduced to dinitrogen gas during denitrification. Both processes can lead to emission of nitrogen oxides. The objective of this literature review is to identify the biological processes and key process conditions that are responsible for N_2O emission.	Nitrous oxide is expected to be emitted during biological nitrogen removal from wastewater, through nitrification and subsequent denitrification (Kampschreur et al., 2009) . In one study, nitrous oxide was found to make up 88% of the emitted carbon dioxide equivalents of a particular WWTP (STOWA, 2010), while Daelman et al. (in press) established the share of nitrous oxide at the same WWTP as 78% based on the long-term, online monitoring campaign that yielded the dataset that is used in the present study. Since nitrous oxide has a global warming potential of 298 CO_2equivalents over a 100 year time horizon, even a low emission contributes significantly to a WWTP's greenhouse gas footprint (IPCC, 2007) . Table 1 provides an overview of the reported monitoring studies of full-scale municipal wastewater treatment plants and

In contrast, only small quantities of N 2 O were generated in the sludge thickener, and the average flux was 2.60 Â 10 À3 ± 1.2 Â 10 À4 mg m À2 min À1 . The average N 2 O fluxes of the first settling, aeration, and secondary settling basins were 0.06 ± 0.005, 0.13 ± 0.002, and 0.02 ± 0.006 mg m À2 min À1 , respectively, and those of the A 2 O reactors were 0.23 ± 0.02, 0.32 ± 0.01, and 0.49 ± 0.01 mg m À2 min À1 , respectively. Denitrification is considered the main source of this N 2 O, emitting approximately 60% of the total N 2 O produced during denitrification in municipal wastewater treatment; however, higher emissions from the A 2 O processes of the advanced treatment activities of nitrification and denitrification bacteria occur in aerated zones than in nonaerated zones (Lim and Kim, 2014) .	**Rank score: 0.75**<br><br>**Filtered reason:** Mentions N2O emissions during biological nitrogen removal but mainly focuses on emission quantification and sampling strategies rather than identifying other sources.<br><br><br>Wastewater treatment plants (WWTPs) are known as emission sources of the greenhouse gas nitrous oxide (N 2 O) (Hanaki et al., 1992; Kampschreur et al., 2009; Desloover et al., 2012; Law et al., 2012) . Nitrous oxide is expected to be emitted during biological nitrogen removal from wastewater, through nitrification and subsequent denitrification (Kampschreur et al., 2009) . In one study, nitrous oxide was found to make up 88% of the emitted carbon dioxide equivalents of a particular WWTP (STOWA, 2010), while Daelman et al. (in press) established the share of nitrous oxide at the same WWTP as 78% based on the long-term, online monitoring campaign that yielded the dataset that is used in the present study. Since nitrous oxide has a global warming potential of 298 CO 2equivalents over a 100 year time horizon, even a low emission	the aerated zones is in agreement with other full-scale studies of aerated and quiescent bioreactors (Czepiel et al., 1995) . The profiles in Fig. 4 show that whilst little atmospheric emission occurs from the quiescent anoxic zones, they actually are responsible for a substantial portion of the N 2 O generated in the bioreactor. Examination of the net generation profile, rather than the emission profile, is therefore more useful in the context of understanding (and possibly controlling) the underlying mechanisms of N 2 O formation. The measurement of positive dissolved N 2 O concentrations in all reactor zones across all seven WWTPs (refer to Fig. 3) suggests that N 2 O is pervasive in most BNR plants. Furthermore, the calculation of positive N 2 O-N generation factors for all WWTPs in Table 2 confirms that these facilities are sources of anthropogenic GHG emissions for UNFCCC accounting purposes. It should also be noted that the generation factors for all sample rounds were positive, even when accounting for the uncertainty of a best-case combination of a low mass transfer coefficient and low dissolved N 2 O concentration. Other studies on nitrous oxide formation have also shown municipal wastewater

	contributes significantly to a WWTP's greenhouse gas footprint (IPCC, 2007) .	treatment is a
For the first time, measurements of the GHG emissions from the disinfection unit were also carried out. The activated sludge (AS) basin, the secondary clarifier, and the chlorination unit were monitored by means of the off-gas method. In particular, the obtained results pay attention to the emissions from treatment units usually neglected in the scientific literature as potential GHG sources (i.e. settler and disinfection), as well as to the CO_2 and N_2O fate in the receiving water body. Indeed, the disinfection unit (i.e. chlorination) revealed to be an important contributor to N_2O emissions because of the chemical reaction between the hydroxylamine (NH_2OH) and the disinfectant agent (NaClO), whereas the settler could be an important source of GHG due to the presence of anaerobic zones and incomplete or partial biological nitrogen removal (BNR) processes. N_2O emission fractions were estimated as a percentage of the influent load of nitrogen (N).	**Rank score: 0.65**<br><br>**Filtered reason:** Notes that N2O emissions occur during nitrification and denitrification and discusses on-site and off-site emissions related to liquid and solid treatment processes, but lacks detailed quantitative data.<br><br>This study concentrated on the on-site and off-site emissions of CO_2 and CH_4 , but did not examine N_2O emissions due to the lack of accurate data during full-scale and pilot-scale operations (Barton and Atwater, 2002) . N_2O emissions occur during both nitrification and denitrification processes and its generation can be prevented by proper control and implementation of complete nitrification and denitrification (Monteith et al., 2005) . On-site GHG emissions are due to liquid and solid treatment processes as well as biogas and fossil fuel combustion for energy generation. Off-site GHG emissions are related to the production of electricity for plant, production and transportation of fuel and chemicals for on-site use, degradation of remaining constituents in the WWTP's effluent to the aquatic	Municipal wastewater treatment plants (WWTPs) have been recognized as potential sources of greenhouse gas (GHG) emissions. 1 In particular, the research focus has been on nitrous oxide (N_2O), which is a significant GHG with approximately 300 times greater global warming potential than that of CO_2 . N_2O is the most significant GHG emitted during wastewater treatment processes. 2 Additionally, it constitutes the most important ozone-depleting emission of the 21st century. 3 Given the significant environmental hazards posed by N_2O, mitigating N_2O emissions is of utmost importance and will greatly impact the sustainability of WWTPs. In WWTPs, N_2O can be emitted the biological nitrogen removal processes, including during autotrophic nitrification, autotrophic denitrification, and heterotrophic denitrification, which have been identified as the major emission sources in wastewater treatment. Due to the challenges of directly monitoring N_2O emissions at WWTPs, N_2O emissions are often estimated by applying a fixed standard emission factor. This approach has several limitations: the fixed emission factor is not only

	recipients, as well as transportation and disposal of solids. In this study GHG emissions have been estimated only for the biological treatment processes and the GHG emissions from sludge conditioning and dewatering unit are not considered here. Also, the off-site GHG emissions because of chemical manufacturing for disinfection of wastewater are not considered in this study.	inconsistent with respect to recent studies, which show great variation in the reported
Although the direct N_2O process emissions released from bioreactors has the highest contribution to the total CF of WWTPs, a minor fraction of N_2O is also emitted from other units of the plant. Hwang et al. [40] performed N_2O measurements in different units of a WWTP, including the primary clarifier and secondary clarifier, as well as the sludge thickener and anaerobic digester. Small quantities of N_2O (0.012 g N_2O/kg TN) were produced in the digester, accounting for less than 1% of the total N_2O emissions. Regarding the primary and secondary clarifier, N_2O emission factors (EFs) of 0.22-0.26 g N_2O/kg TN were reported based on the full-scale measurements of Hwang et al. (2016) . For comparison, Solís et al. [41] applied an EF of 0.01 kg N_2O-N/kg TN to predict the direct N_2O emissions from sludge storage, assuming uncovered storage	**Rank score: 0.65**<br><br>**Filtered reason:** Discusses the significance of N2O emissions in WWTPs and their contribution to the climate footprint, with some data on emission variability, but less focus on specific sources beyond biological treatment.<br><br>Since about two decades it has been recognised that nitrous oxide (N_2O) is emitted during the biological treatment of wastewater (Hanaki et al., 1992) . Considering its global warming potential of 298 CO_2 -equivalents over a hundred year time horizon, the emission of nitrous oxide can contribute significantly to the climate footprint of a wastewater treatment plant (IPCC, 2013) . At the WWTP that is also the location for	Nitrous oxide (N_2O) is considered to be the dominant ozonedepleting substance and a greenhouse gas of great importance in the global warming process which has a greenhouse gas effect about 300 times as great as that of carbon oxide (CO_2) (Kampschreur et al., 2009) . Wastewater treatment accounts for 3.2-10.2% of the anthropogenic emission of N_2O, which is mainly produced during transportation and biological treatment of wastewater (Desloover et al., 2012) . The N_2O emission factor (amount of N_2O-N emitted relative to the nitrogen load) reported for full scale plants varies substantially, ranging from 0% to 25% (Law et al., 2012) . Hence, the sources, mechanism, and minimization of N_2O emission in wastewater treatment are of growing concern. N_2O generation mainly occurs in biological nitrogen removal (BNR)

throughout the year.	the present study, nitrous oxide was found to make up 78.4% of the plants climate footprint, expressed as CO_2-equivalents, the remainder of the emissions being methane (13.5%) and the indirect carbon dioxide related to the plant's energy consumption (8.1%) (Daelman et al., 2013b) . Besides a considerable greenhouse gas, nitrous oxide is currently also the most important ozone-depleting substance emitted (Ravishankara et al., 2009) . In literature, emission values as high as 25.4% of the nitrogen load of the WWTP have been reported and from Table 1 it is obvious that there is a huge spread of the reported emissions.	via nitrification and denitrification in wastewater treatment plants (WWTPs) (Kampschreur et al., 2009) and commonly comprises three pathways, the contributions of which were still controversial: (1) nitrifier den
The effluent BOD load can contribute to further direct CH_4 emissions from the natural processes in recipients [27] . The GHG emissions from the preliminary treatment and sludge treatment stages do not attract as much attention as the emissions from the biological stage employing biological nutrient removal (BNR). Bioreactors have been recognized as the main source of GHG emissions in WWTPs. Campos et al. [37] reported that, the dominant N_2O production occurs in the bioreactors (90%) while for CH_4 emissions the sludge line with AD was responsible for over 70% and the remaining portion originates from bioreactors. N_2O produced under favorable conditions (either anoxic	**Rank score: 0.65**<br><br>**Filtered reason:** General discussion on N2O emissions from WWTPs and the importance of quantification and mitigation, but lacks specific details on sources beyond bioreactors.<br><br>The significance of greenhouse gas (GHG) emissions from wastewater treatment plants (WWTPs) has gained considerable attention in recent years as a critical objective in the pursuit of sustainable environmental management and climate change. Nitrous oxide (N_2O)	The greenhouse gasses, nitrous oxide (N_2O), carbon dioxide (CO_2), and methane (CH_4) can be produced during wastewater treatment. There is an increasing need to reduce these emissions and to identify the factors that control the greenhouse gas (GHG) emissions from wastewater treatment plants (WWTPs). CO_2 emission can be assessed based on energy demand of a treatment plant, because the CO_2 that is directly produced by conversion of organic matter is short-cycle CO_2 (not originating from fossil fuels), which is considered not to contribute to the greenhouse effect. Methane is expected to be prevalently emitted in the sewerage system and

or aerobic) is emitted via either saturation-induced liquid-gas transfer or stripped to the air via aeration [38] . The effluent N load also becomes the source of direct N_2O emissions in the recipients.	is a key potent GHG that is emitted from WWTPs during nitrogen conversion processes (Wan and Volcke, 2022) . N_2O emissions can contribute to up to 80 % of the overall carbon footprint of a WWTP, as their global warming potential is approximately 300 times stronger than that of carbon dioxide (CO_2) (Daelman et al., 2013) . Moreover, N_2O is claimed to be the main ozone-depleting substance of the 21st century (Ravishankara et al., 2009) . Consequently, it is imperative to employ effective mitigation strategies aimed at reducing N_2O emissions from WWTPs. To achieve this, quantification of N_2O emissions is necessary. Monitoring campaigns have been demonstrated to exhibit notable constraints, including substantial costs and intricate processes.	during sludge handling. N_2O is an important greenhouse gas, having a 300fold stronger effect than carbon dioxide (IPCC, 2001) . Therefore, even low amounts of N_2O emission are unwanted. The source and magnitude of N_2O emissions in WWTPs are relatively unknown and subject of debate in the literature. N_2O emissions are associated with several processes in wastewater treatment plants and the emission fluxes are extremely variable and depend on many operational parameters and environmental conditions. For estimation of the N_2O emission from WWTPs,

Table S18. Review examples of WaterRAG, naive RAG, GPT-4.1

**Question:** Please conduct a comprehensive review on the theme "Reducing Greenhouse Gas Emissions from Wastewater Treatment Processes through Operational Optimization". The review should include the following elements:<br>1.Reducing N2O emissions from wastewater treatment processes through operational optimization, and promising new technologies<br>2.Reducing CH4 emissions from wastewater treatment processes through operational optimization<br>3.Comparing the feasibility and difficulty of CH4 and N2O detection and prevention<br>4.What are the CH4 emission sources in the entire wastewater treatment system, and what are the approximate proportions of different emission sources?
**GPT 4.1**
Reducing Greenhouse Gas Emissions from Wastewater Treatment Processes through Operational Optimization<br>Reducing N_2O Emissions from Wastewater Treatment Processes<br>Background<br>Nitrous oxide (N_2O) is a potent greenhouse gas (GHG) with a global warming potential (GWP) approximately 298 times that of CO_2 over a 100-year period. Wastewater treatment plants (WWTPs), especially those with biological nitrogen removal, are significant anthropogenic sources of N_2O due to microbial processes such as nitrification and denitrification.<br>Operational Optimization Strategies<br>a. Controlling Dissolved Oxygen (DO) Levels<br>Low DO can promote incomplete nitrification and denitrification, leading to higher N_2O emissions.<br>Optimal DO (typically 1.5–2.0 mg/L) helps maintain complete nitrification and denitrification, reducing N_2O formation.<br>b. Optimizing Carbon Source Addition

Sufficient readily biodegradable carbon (e.g., methanol, acetate) ensures complete denitrification, minimizing N_2O accumulation.

c. pH and Temperature Control

Maintaining neutral pH and optimal temperatures (15 – 25° C) supports efficient microbial activity and reduces N_2O production.

d. Intermittent Aeration

Alternating aerobic and anoxic phases can optimize nitrogen removal and reduce N_2O emissions by preventing accumulation of nitrite (NO_2^-), a key precursor for N_2O.

e. Sludge Retention Time (SRT) Management

Proper SRT (8 – 15 days) ensures a stable microbial community, reducing the risk of N_2O-producing conditions.

Promising New Technologies

Real-Time N_2O Monitoring and Control: Online sensors and feedback control systems allow dynamic adjustment of operational parameters to minimize N_2O peaks.

Shortcut Nitrogen Removal (e.g., Partial Nitritation-Anammox): These processes can reduce N_2O emissions compared to conventional nitrification-denitrification.

Bioaugmentation: Introducing specialized microbial consortia that favor complete denitrification can lower N_2O emissions.

Advanced Process Modeling: Machine learning and process simulation tools can predict N_2O hotspots and guide operational changes.

Reducing CH_4 Emissions from Wastewater Treatment Processes

Background

Methane (CH_4) has a GWP 25 – 34 times that of CO_2 over 100 years. CH_4 is produced primarily under anaerobic conditions during the degradation of organic matter.

Operational Optimization Strategies

a. Minimizing Anaerobic Zones in Aerobic Treatment

Preventing the formation of anaerobic pockets in aeration tanks and secondary clarifiers reduces CH_4 production.

b. Efficient Sludge Handling

Rapid transfer and stabilization of primary and secondary sludge minimize uncontrolled anaerobic digestion and fugitive CH_4 emissions.

c. Covering and Capturing Biogas

Covering anaerobic digesters and storage tanks allows for CH_4 capture and utilization, preventing atmospheric release.

d. Optimizing Sludge Retention and Dewatering

Shortening sludge storage times and improving dewatering efficiency reduce the potential for CH_4 formation.

e. Process Integration

Integrating anaerobic digestion with combined heat and power (CHP) systems maximizes CH_4 utilization and energy recovery.

Comparing the Feasibility and Difficulty of CH_4 and N_2O Detection and Prevention

Detection

N_2O Detection: More challenging due to its low concentrations, high temporal variability, and multiple emission points (liquid and gas phases). Requires sensitive, real-time gas analyzers (e.g., FTIR, GC-ECD).

CH_4 Detection: Easier due to higher concentrations in biogas and more localized emission sources (digesters, sludge storage). Portable gas analyzers and flow meters are commonly used.

Prevention

N_2O Prevention: More complex, as it is produced via multiple biological pathways sensitive to operational fluctuations. Requires fine-tuned process control and real-time monitoring.

CH_4 Prevention: More straightforward, focusing on containment and capture of biogas, and minimizing anaerobic conditions outside controlled digesters.

Summary Table:

Aspect	N_2O	CH_4
Detection	Difficult, low conc., variable	Easier, higher conc., localized
Prevention	Complex, multi-pathway	Straightforward, containment

CH_4 Emission Sources in the Wastewater Treatment System and Their Proportions

Main CH_4 Emission Sources

Anaerobic Digesters: Controlled biogas production; emissions occur mainly from leaks or incomplete capture.

Sludge Storage and Handling: Anaerobic conditions in sludge holding tanks, lagoons, or during dewatering can generate CH_4.

Sewer System: Anaerobic zones in long or stagnant sewers can produce CH_4 before the wastewater reaches the WWTP.

Primary Sedimentation Tanks: Some CH_4 can be produced if anaerobic conditions develop.

Effluent Discharge: Dissolved CH_4 in treated effluent can be released to the atmosphere downstream.

Approximate Proportions of CH_4 Emission Sources

Anaerobic Digesters: 50 – 70% (if biogas is not fully captured)

Sludge Storage/Handling: 10 – 30%

Sewer System: 10 – 20%

Primary Sedimentation Tanks: <5%

Effluent Discharge: <5%

Note: Proportions vary depending on plant design, operational practices, and whether biogas capture systems are in place.

References<br>IPCC 2019 Refinement to the 2006 Guidelines for National Greenhouse Gas Inventories.<br>Foley, J., et al. (2010). "N_2O and CH_4 emission from wastewater collection and treatment systems: A review." Water Research, 44(9), 2463-2476.<br>Ahn, J.H., et al. (2010). "N_2O emissions from activated sludge processes, 2008 – 2009: Results of a national monitoring survey in the United States." Environmental Science & Technology, 44(12), 4505-4511.<br>Massara, T.M., et al. (2017). "A review on nitrous oxide (N_2O) emissions during biological nutrient removal from municipal wastewater and sludge reject water." Science of the Total Environment, 596-597, 106-123.<br>Daelman, M.R.J., et al. (2012). "Methane emission from municipal wastewater treatment systems: A review." Water Research, 46(11), 3657-3670.<br><br>Conclusion<br>Operational optimization in wastewater treatment plants offers significant potential to reduce GHG emissions, particularly N_2O and CH_4. While N_2O mitigation requires sophisticated process control and monitoring due to its complex formation pathways, CH_4 emissions can be effectively reduced through containment, capture, and utilization strategies. Understanding the sources and proportions of emissions is essential for targeted mitigation and for meeting climate goals in the wastewater sector.
**GPT 4.1 Search**
Wastewater treatment plants (WWTPs) are significant sources of greenhouse gas (GHG) emissions, particularly nitrous oxide (N_2O) and methane (CH_4), both of which have substantial global warming potentials. Operational optimization within these facilities offers a promising avenue for mitigating such emissions. This review critically examines strategies for reducing N_2O and CH_4 emissions through operational adjustments, compares the feasibility of detecting and preventing these gases, and identifies their primary emission sources within the wastewater treatment process.

1. Reducing N_2O Emissions through Operational Optimization and Emerging Technologies

N_2O emissions in WWTPs primarily arise during biological nitrogen removal processes, notably nitrification and denitrification. Operational strategies to mitigate these emissions include:

- **Aeration Control**: Fine-tuning dissolved oxygen (DO) levels can significantly influence N_2O production. For instance, intermittent aeration has been shown to enhance total nitrogen removal efficiency while reducing N_2O emissions compared to continuous aeration. This approach also promotes the enrichment of complete ammonia oxidizers (comammox), which emit less N_2O than traditional ammonia-oxidizing bacteria (AOB) ([frontiersin.org](https://www.frontiersin.org/articles/10.3389/fbioe.2023.1247711/full?utm_source=openai)).

- **Carbon Supplementation**: Introducing external carbon sources can improve denitrification efficiency, thereby reducing N_2O emissions. Optimizing the carbon-to-nitrogen (C/N) ratio ensures complete denitrification, minimizing N_2O release ([nature.com](https://www.nature.com/nature-index/article/10.1016/j.watres.2025.123247?utm_source=openai)).

- **Process Optimization**: Implementing advanced control strategies, such as multi-agent reinforcement learning, can optimize operational parameters like DO and chemical dosing. This approach has demonstrated reductions in GHG emissions, operational costs, and improved effluent quality ([arxiv.org](https://arxiv.org/abs/2008.10417?utm_source=openai)).

Emerging technologies, such as denitrifying bioscrubbers, offer additional avenues for N_2O mitigation. These systems treat N_2O-rich exhaust air from deammonification processes, effectively reducing emissions

([ewwmconference.com](https://ewwmconference.com/2025/session/3099505/denitrifying-bioscrubbers-to-reduce-nitrous-oxide-emissions-in-the-exhaust-air-of-wastewater-treatment-plants?utm_source=openai)).

2. Reducing CH_4 Emissions through Operational Optimization

CH_4 emissions in WWTPs are primarily associated with anaerobic processes and the handling of sludge. Operational strategies to mitigate CH_4 emissions include:

- **Aeration Strategies**: Enhancing aeration in treatment processes can reduce CH_4 emissions by promoting aerobic conditions that inhibit methanogenesis. For example, windrow composting has been shown to reduce CH_4 emissions by approximately 50% compared to static storage, primarily due to decreased methanogenic activity ([academic.oup.com](https://academic.oup.com/femsec/article-abstract/90/3/575/537431?utm_source=openai)).

- **Process Control Optimization**: Implementing data-driven predictive control policies for variable-frequency pumps in wastewater systems can minimize energy consumption and reduce CH_4 emissions. Such approaches have demonstrated a 16.7% decrease in electrical energy consumption, indirectly mitigating CH_4 emissions ([arxiv.org](https://arxiv.org/abs/1902.03417?utm_source=openai)).

3. Feasibility and Difficulty of CH_4 and N_2O Detection and Prevention

Detecting and preventing N_2O and CH_4 emissions present distinct challenges:

- **Detection**: N_2O detection is complex due to its episodic and variable emissions, requiring high-frequency monitoring and advanced analytical techniques. CH_4 detection is relatively straightforward, as emissions are more consistent and can be measured using standard gas analyzers.

- **Prevention**: Preventing N_2O emissions is challenging due to the intricate microbial processes involved in nitrogen removal. Operational strategies must carefully balance nitrification and denitrification to minimize N_2O production. In contrast, CH_4 prevention focuses on managing anaerobic conditions and optimizing sludge handling, which are more directly controllable through operational adjustments.

4. CH_4 Emission Sources and Their Proportions in Wastewater Treatment Systems

CH_4 emissions in WWTPs originate from several sources:

- **Anaerobic Digesters**: These units are designed to capture and utilize CH_4; however, leaks and inefficiencies can lead to emissions.

- **Sludge Handling and Storage**: Anaerobic conditions in sludge storage can produce CH_4.

- **Primary Sedimentation Tanks**: Organic matter decomposition under low oxygen conditions can generate CH_4.

Quantifying the exact proportions of CH_4 emissions from these sources varies depending on plant design and operational practices. However, studies indicate that anaerobic digesters and sludge handling are the predominant sources. For instance, in piggery wastewater treatment systems, CH_4 emissions were primarily

associated with anaerobic processes, with emission factors of 0.11 kg CH_4 per head per year ([mdpi.com](https://www.mdpi.com/2071-1050/18/1/321?utm_source=openai)).<br><br>**Conclusion**<br><br>Operational optimization in WWTPs offers significant potential for reducing GHG emissions. While strategies such as aeration control, carbon supplementation, and advanced process control have shown promise in mitigating N_2O and CH_4 emissions, challenges remain in detection and prevention, particularly for N_2O. Future research should focus on developing reliable quantification methods, conducting long-term full-scale trials, and exploring novel mitigation strategies to enhance the sustainability of wastewater treatment processes.
**Naive RAG**
Comprehensive Review: Reducing Greenhouse Gas Emissions from Wastewater Treatment Processes through Operational Optimization<br><br>Reducing N_2O Emissions from Wastewater Treatment Processes through Operational Optimization and Promising New Technologies<br>Operational Optimization Strategies:<br>Dissolved Oxygen (DO) Control: Strict control of DO during denitrification is critical for minimizing N_2O emissions. Excessively low or high DO can promote incomplete denitrification, leading to increased N_2O release (Zhang et al., 2025 [8]; Vieira et al., 2019 [21]).<br>Carbon Source Management: The availability and type of carbon source directly affect both nitrogen removal efficiency and N_2O emissions, especially during denitrification. Optimizing carbon supplementation can reduce N_2O formation (An et al., 2025 [25]).

Process Optimization: Rational configuration design and process optimization (e.g., adjusting SRT, temperature, and feed control) can reduce N_2O emissions. However, direct GHG emissions may be less sensitive to configuration than to operational controls (He et al., 2023 [19]).
Advanced Control Strategies: The integration of GHG emission indicators into plant control systems is emerging, but achieving optimal trade-offs between effluent quality, cost, and GHG emissions remains challenging (Lu et al., 2023 [13]).
Promising New Technologies:
CANDO Process: This process enables the recovery of N_2O as a renewable resource, offering both emission reduction and energy recovery potential (Kumar et al., 2021 [17]; Lee et al., 2022 [20]).
Microalgae-Assisted Treatment: Microalgae can assimilate nitrogen and reduce N_2O emissions, though further research is needed for practical adaptation (Kumar et al., 2021 [17]).
Automatic and Intelligent Control: The use of intelligent prediction and precise mitigation strategies, including real-time monitoring and adaptive control, is highlighted as a future direction for minimizing N_2O emissions (Zhang et al., 2025 [8]; Lu et al., 2023 [13]).
Quantitative Insights:
N_2O has a global warming potential (GWP) 298–300 times that of CO_2 (Schneider et al., 2015 [7]; Kampschreur et al., 2009 [5]).
Emission factors for N_2O are highly variable, with reported emissions ranging from 0–90% of the nitrogen load (Sweetapple et al., 2013 [4]).

Reducing CH_4 Emissions from Wastewater Treatment Processes through Operational Optimization
Key Operational Strategies:
Anaerobic Process Management: Methane is primarily produced under anaerobic conditions, especially during sludge treatment and stabilization. Optimizing anaerobic digestion (e.g., temperature, retention time, and

mixing) and ensuring effective biogas capture are essential (Shukuru et al., 2025 [10]; Karakas et al., 2024 [9]).
Aeration and Oxygenation: In the water treatment phase, maintaining aerobic conditions reduces CH_4 formation, as aerobic environments favor conversion of organic matter to CO_2 rather than CH_4 (Yu et al., 2024 [12]).
Sludge Handling: Sludge treatment and disposal are the dominant sources of CH_4 emissions, contributing about 9 times more CH_4 than the water treatment process itself (Yu et al., 2024 [2]).
Vigilant Monitoring: Anaerobic sludge blanket reactors and lagoons require close monitoring to prevent fugitive methane emissions (Shukuru et al., 2025 [10], [24]).
Quantitative Insights:
CH_4 from wastewater treatment accounts for approximately 5% of global methane sources and 3.6% of total CO_2-equivalent emissions (Karakas et al., 2024 [9]).
About half of the COD removed in sludge treatment is ultimately converted to CH_4 (Yu et al., 2024 [2]).

Comparing the Feasibility and Difficulty of CH_4 and N_2O Detection and Prevention
Detection:
N_2O: Emissions are highly variable and influenced by operational parameters, making detection and quantification challenging. Emission factors for N_2O show significant variability (0 – 90% of nitrogen load), and emissions can occur in both aerobic and anoxic zones (Sweetapple et al., 2013 [4]; Kampschreur et al., 2009 [5]).
CH_4: Detection is generally more straightforward in anaerobic zones and during sludge treatment, where emissions are more predictable. However, fugitive emissions from poorly managed anaerobic systems can be difficult to quantify (Shukuru et al., 2025 [10]; Karakas et al., 2024 [9]).
Prevention:

N_2O: Prevention is complex due to its formation in both nitrification and denitrification steps, requiring precise control of DO, carbon sources, and process stability. Small operational changes can lead to large emission swings (Vieira et al., 2019 [21]; An et al., 2025 [25]).
CH_4: Prevention is more feasible through effective biogas capture and maintaining aerobic conditions in water treatment. However, the challenge lies in managing emissions from sludge treatment and ensuring gas-tight systems (Yu et al., 2024 [2]; Shukuru et al., 2025 [10]).
Conclusion: N_2O detection and prevention are generally more challenging due to high variability and sensitivity to operational changes, whereas CH_4 emissions are more predictable but require robust infrastructure for capture and control.

CH_4 Emission Sources in the Entire Wastewater Treatment System and Approximate Proportions
Main Sources:
Sludge Treatment and Disposal: The dominant source, contributing about 9 times more CH_4 than the water treatment process (Yu et al., 2024 [2]).
Anaerobic Stabilization: Significant CH_4 emissions are associated with anaerobic stabilization ponds and digesters (Karakas et al., 2024 [9]).
Biological Treatment: Some CH_4 is produced in biological treatment steps, especially under low-oxygen or anoxic conditions, but this is minor compared to sludge treatment (Karakas et al., 2024 [9]; Yu et al., 2024 [2]).
Advanced Treatment: Minor source, but can contribute if anaerobic conditions develop (Karakas et al., 2024 [9]).
Proportions:
Sludge Treatment and Disposal: ~90% of total CH_4 emissions from the WWTP (Yu et al., 2024 [2]).
Water Treatment Process: ~10% of total CH_4 emissions (Yu et al., 2024 [2]).
Supporting Data:
About half of the COD removed in sludge is converted to CH_4 (Yu et al., 2024 [2]).

CH_4 emission factor in the water treatment process is lower than IPCC standards, while sludge treatment is the major contributor (Yu et al., 2024 [12]).

Actionable Insights and Practical Recommendations

Prioritize DO and Carbon Source Control: Implement advanced DO control and optimize carbon dosing to minimize N_2O emissions, especially during denitrification.

Enhance Biogas Capture: Invest in robust gas-tight infrastructure for sludge treatment to capture and utilize CH_4, reducing fugitive emissions.

Adopt Integrated Monitoring: Deploy real-time, plant-wide GHG monitoring systems to identify emission hotspots and enable adaptive operational responses.

Leverage New Technologies: Explore CANDO and microalgae-assisted processes for N_2O mitigation and valorization.

Holistic Plant Optimization: Balance effluent quality, operational costs, and GHG emissions in plant-wide optimization frameworks (Lu et al., 2023 [13]; Flores-Alsina et al., 2014 [22]).

References

[2] Yu, QB et al. (2024). ENVIRON RES. 10.1016/j.envres.2024.119398
[4] Sweetapple, C et al. (2013). WATER RES. 10.1016/j.watres.2013.05.021
[5] Kampschreur, MJ et al. (2009). WATER RES. 10.1016/j.watres.2009.03.001
[7] Schneider, AG et al. (2015). SCI TOTAL ENVIRON. 10.1016/j.scitotenv.2014.10.060
[8] Zhang, ZT et al. (2025). ENVIRON RES. 10.1016/j.envres.2025.121052
[9] Karakas, A et al. (2024). J WATER PROCESS ENG. 10.1016/j.jwpe.2024.105254
[10,24] Shukuru, BN et al. (2025). RENEW SUST ENERG REV. 10.1016/j.rser.2024.115203
[12] Yu, QB et al. (2024). ENVIRON RES. 10.1016/j.envres.2024.119398
[13] Lu, H et al. (2023). SCI TOTAL ENVIRON. 10.1016/j.scitotenv.2022.158849

[17,20] Kumar, A et al. (2021). CHEMOSPHERE. 10.1016/j.chemosphere.2021.129694; Lee, YJ et al. (2022). BIORESOURCE TECHNOL. 10.1016/j.biortech.2022.127747<br>[19] He, YY et al. (2023). RENEW SUST ENERG REV. 10.1016/j.rser.2023.113547<br>[21] Vieira, A et al. (2019). J CLEAN PROD. 10.1016/j.jclepro.2018.12.229<br>[22] Flores-Alsina, X et al. (2014). SCI TOTAL ENVIRON. 10.1016/j.scitotenv.2013.07.046<br>[25] An, ZM et al. (2025). WATER RES. 10.1016/j.watres.2025.123247
**WaterRAG Initial review**
Comprehensive Literature Review: Reducing Greenhouse Gas Emissions from Wastewater Treatment Processes through Operational Optimization<br>Introduction<br>Wastewater treatment plants (WWTPs) are significant sources of greenhouse gas (GHG) emissions, primarily methane (CH_4) and nitrous oxide (N_2O), which contribute to climate change. Operational optimization of these facilities offers a promising pathway to mitigate these emissions. This review synthesizes current knowledge on reducing N_2O and CH_4 emissions through operational strategies, evaluates the feasibility of detecting and preventing these emissions, and identifies the primary sources of CH_4 emissions within wastewater treatment systems.<br>Reducing N_2O Emissions through Operational Optimization and Promising Technologies<br>Current State of Knowledge:<br>N_2O emissions in WWTPs primarily arise from nitrification and denitrification processes. The optimization of dissolved oxygen (DO) levels is critical, as it directly influences N_2O production during these processes (Shahid et al., 2023; Lu et al., 2024). Studies indicate that maintaining optimal DO concentrations can significantly reduce N_2O emissions without compromising treatment efficiency (Sweetapple et al., 2014; An et al., 2025).<br>Promising Technologies:

Advanced biological processes such as the Anammox (anaerobic ammonium oxidation) process have shown potential in reducing N_2O emissions. This process enhances nitrogen removal efficiency while minimizing N_2O production compared to conventional methods (Shukuru et al., 2025). Furthermore, integrating intermittent aeration strategies and employing biofilm carriers in reactors can further mitigate N_2O emissions (Shukuru et al., 2025).

Research Gaps:

Despite advancements, challenges remain in achieving stable nitrite supply and maintaining Anammox activity at low temperatures (He et al., 2023). Future research should focus on developing robust control strategies that ensure consistent performance across varying operational conditions.

Reducing CH_4 Emissions through Operational Optimization

Current State of Knowledge:

CH_4 emissions predominantly occur during anaerobic digestion and sludge handling processes. Effective strategies to reduce CH_4 emissions include optimizing sludge retention time (SRT) and controlling temperature during digestion to enhance biogas recovery (Oshita et al., 2014; Ma et al., 2017). Additionally, minimizing transit time of digested sludge to dewatering processes can reduce CH_4 production (Oshita et al., 2014).

Promising Approaches:

Utilizing biogas for on-site electricity generation and integrating advanced technologies like membrane contactors (MCs) for efficient CH_4 recovery are promising approaches (He et al., 2023). These strategies not only mitigate emissions but also enhance energy recovery, contributing to the sustainability of WWTPs.

Research Gaps:

There is a need for further research on optimizing biogas recovery systems to maximize CH_4 capture and utilization. Additionally, understanding the impact of climatic conditions on CH_4 emissions can inform tailored mitigation strategies (Shukuru et al., 2025).

Feasibility and Difficulty of CH_4 and N_2O Detection and Prevention

Detection and Prevention Challenges:

Detecting and preventing CH_4 and N_2O emissions pose distinct challenges. N_2O emissions are highly variable and influenced by operational conditions, making continuous monitoring essential (Rodriguez-Caballero et al., 2014). Advanced monitoring technologies and dynamic simulations are critical for accurate detection and control (Lu et al., 2024).

In contrast, CH_4 emissions are more predictable, primarily occurring during specific processes like anaerobic digestion. However, the challenge lies in minimizing fugitive emissions during biogas handling and utilization (Song et al., 2023).

Comparative Analysis:

N_2O detection is generally more complex due to its episodic nature and the influence of multiple operational parameters. In contrast, CH_4 detection is more straightforward but requires effective containment and utilization strategies to prevent emissions.

CH_4 Emission Sources and Proportions in Wastewater Treatment Systems

Emission Sources:

CH_4 emissions in WWTPs primarily originate from anaerobic digestion, sludge handling, and open treatment systems like stabilization ponds (Song et al., 2023). Anaerobic processes such as Upflow Anaerobic Sludge Blanket (UASB) reactors are significant contributors due to their potential for fugitive emissions (Song et al., 2023).

Proportions of Emission Sources:

Anaerobic digestion is the dominant source, accounting for a substantial portion of CH_4 emissions. Open systems like stabilization ponds contribute significantly to fugitive emissions, while emissions from aerobic treatment units are relatively low (Song et al., 2023).

Conclusion

Operational optimization presents a viable pathway to reduce GHG emissions from WWTPs. While significant progress has been made in understanding and mitigating N_2O and CH_4 emissions, challenges remain in

achieving consistent and efficient emission reductions. Future research should focus on developing integrated strategies that balance emission reduction with treatment efficiency and cost-effectiveness. Additionally, advancing monitoring technologies and understanding the impact of climatic conditions will be crucial for effective emission control. Addressing these research gaps will be essential for achieving net-zero GHG emissions in wastewater treatment systems.

WaterRAG Final review

Comprehensive Literature Review: Reducing Greenhouse Gas Emissions from Wastewater Treatment Processes through Operational Optimization

Introduction

Wastewater treatment plants (WWTPs) are significant sources of non-CO_2 greenhouse gases (GHGs), primarily nitrous oxide (N_2O) and methane (CH_4), both of which have global warming potentials (GWPs) far exceeding that of carbon dioxide (CO_2) (N_2O: ~265–300×, CH_4: ~25–28×, IPCC 2013/2022). As the sector moves toward energy optimization and net-zero carbon goals, direct emissions of N_2O and CH_4 increasingly dominate the carbon footprint of WWTPs (Valkova et al., 2021; Maktabifard et al., 2023). This review critically examines the state of knowledge and operational strategies for reducing these emissions, compares detection and mitigation challenges, and synthesizes insights into CH_4 emission sources and their relative contributions. Additionally, it addresses the mitigation potential and feasibility of various strategies, the limitations and uncertainties of current GHG detection and emission factor methodologies, and the influence of policy and regulatory frameworks. The review also explores the trade-offs and potential unintended consequences of operational optimization, providing a holistic perspective on sustainable GHG management in WWTPs. Recent advances in CH_4-specific mitigation technologies, real-world case studies, and a critical evaluation of emission factor uncertainties are integrated to provide a comprehensive and up-to-date synthesis.

Reducing N_2O Emissions through Operational Optimization and Promising New Technologies

Mechanisms and Key Emission Points

N_2O emissions in WWTPs primarily arise from biological nitrogen removal (BNR) processes—specifically, during nitrification and denitrification in activated sludge systems (Blomberg et al., 2018; Du et al., 2022). Up to 80% of a WWTP's carbon footprint can be attributed to N_2O (Khalil et al., 2023; Daelman et al., 2013). Emission factors are highly variable, ranging from 0.036% to over 2% of influent total nitrogen (TN), with some studies reporting dynamic emission factors up to 14.6% (Rodriguez-Caballero et al., 2014; Hwangbo et al., 2021).

Operational Optimization Strategies

a. Aeration Control and Dissolved Oxygen (DO) Management

Maintaining optimal DO levels in aeration tanks is critical. Both low and excessively high DO can trigger N_2O production—low DO promotes incomplete nitrification/denitrification, while high DO can inhibit denitrifiers and lead to N_2O accumulation (Shukuru et al., 2025; Shahid et al., 2023). Advanced sensor-based control of DO, coupled with feedback systems, has been shown to reduce N_2O peaks without compromising treatment efficiency (Shukuru et al., 2025; Daelman et al., 2013). Quantitatively, Solís et al. (2022) demonstrated that novel DO control strategies targeting N_2O reduction can achieve up to a 9% decrease in total GHG emissions compared to baseline operations, although the overall impact is constrained by indirect emissions and other uncontrollable sources.

b. Process Configuration and Nitrogen Loading

Fluctuations in influent nitrogen or organic carbon can destabilize microbial communities, increasing N_2O emissions (Lei et al., 2025; Ribera-Guardia et al., 2019). Spatial and temporal variations in N_2O emissions are observed along plug-flow reactors, with peaks often linked to zones where NH_4^+ first appears (Ribera-Guardia et al., 2019).

c. Advanced Biological Processes

Anammox-based processes offer lower N_2O emissions compared to conventional nitrification-denitrification, as they bypass nitrite/nitrate reduction steps where N_2O is typically produced (Du et al., 2022; Shukuru et al.,

2025; He et al., 2023). Partial nitritation/anammox and shortcut denitrification processes can further reduce N_2O by minimizing nitrite accumulation (He et al., 2023).

d. Process Stability and Operational Troubleshooting

Operational problems such as sudden changes in temperature, digester foaming, or sub-optimal BNR operation can cause N_2O emissions to spike by up to tenfold (Yoshida et al., 2014).

Promising New Technologies and Mitigation Approaches

Beyond operational optimization, several innovative N_2O mitigation technologies are emerging:

Chemical Inhibitors and Additives: The use of chemical inhibitors targeting key enzymes in the N_2O production pathway has shown promise at lab scale but faces challenges in full-scale application due to potential toxicity and cost (Yao et al., 2022).

Membrane Technologies: Membrane-aerated biofilm reactors (MABRs) and other membrane-based processes can provide more controlled oxygen delivery, reducing N_2O formation by stabilizing redox conditions (Yao et al., 2022).

Novel Catalysts: Research into catalytic materials that promote complete denitrification to N_2, bypassing N_2O, is ongoing, though practical deployment remains limited (Yao et al., 2022).

Cycle and Phase Modulation: Full-scale studies have demonstrated that shortening aerobic/anoxic cycles can reduce N_2O emissions by up to 56% (Vasilaki et al., 2019; Mampaey et al., 2016). For example, an optimum control strategy using short aerobic-anoxic cycles (20-min aerobic phase and short anoxic stage) has been shown to minimize N_2O emissions in sequencing batch reactors (Rodriguez-Caballero et al., 2015).

Machine Learning and Predictive Modeling: Machine learning models and extended activated sludge models (e.g., ASM3-N_2O) are being developed to dynamically predict N_2O emissions and inform real-time operational adjustments (Khalil et al., 2023; Hwangbo et al., 2021; Lei et al., 2025). Zhang et al. (2025) highlight that while operational optimization and intelligent prediction tools can contribute to GHG mitigation, their direct impact on N_2O reduction is modest (3.27% reduction in total GHG emissions), emphasizing the need for integrated strategies.

Plant-Wide Modeling: Integrating plant-wide models enables holistic GHG management, optimizing both process performance and emission reduction (Guo et al., 2024; Solís et al., 2022).

N_2O Recovery: Emerging technologies propose capturing N_2O for use as an oxidant in energy recovery systems, turning a liability into a resource (Maktabifard et al., 2023).

Quantitative Comparison of Mitigation Potential and Case Studies

Recent scenario analyses provide valuable quantitative insights. Nayeb et al. (2019) found that upgrading WWTPs to include modern biological nitrogen removal units can significantly reduce N_2O emissions, with reductions closely tied to the proportion of advanced facilities in the system. Multi-objective optimization studies, such as those by Sweetapple et al. (2014), have demonstrated that GHG emissions can be reduced by up to 18.5% with a corresponding 4.1% reduction in operational costs, though trade-offs exist between emissions, costs, and effluent quality. In their case studies, a 10% reduction in GHG emissions was achieved without increasing operational costs, but further reductions often required accepting higher costs or increased effluent nitrogen. These real-world examples underscore the practical feasibility and limitations of operational optimization for N_2O mitigation.

Economic and Practical Feasibility

Implementing advanced N_2O mitigation strategies in full-scale WWTPs presents both technical and economic challenges. The adoption of real-time monitoring, machine learning control, and N_2O recovery technologies often requires substantial initial investment and operational complexity (Molinos-Senante et al., 2014; Cairone et al., 2024). However, these investments can be offset by long-term operational savings, improved process efficiency, and enhanced resource recovery (Cairone et al., 2024). Shang et al. (2024) emphasize that successful mitigation depends on flexible strategies that accommodate dynamic plant conditions and are supported by regulatory frameworks incentivizing emission reductions. In practice, economic feasibility and policy support are as critical as technical performance in determining the adoption of advanced N_2O mitigation technologies.

Policy and Regulatory Frameworks

Policy interventions are increasingly recognized as essential drivers for GHG mitigation in WWTPs. For instance, in China, targeted policies have been proposed to prioritize provinces with high N_2O emission factors or high emission reduction potential, with incentive mechanisms encouraging operators to implement measures such as aeration and feed optimization (Yang et al., 2023). In Australia, carbon pricing and emission reduction targets have spurred operational changes, with some WWTPs achieving a 35% reduction in N_2O emissions and 20% reduction in operating costs through DO management (Duan et al., 2020; Yang et al., 2023). Similarly, carbon taxes in Canada (e.g., Quebec, British Columbia) and green finance initiatives in ASEAN and the Middle East are fostering investment in GHG mitigation (Molinos-Senante et al., 2014; Yao et al., 2022). Nonetheless, transaction costs, asymmetric information, and the need to balance effluent quality with emission reduction remain policy challenges (Molinos-Senante et al., 2014; Yao et al., 2022).

Trade-Offs and Unintended Consequences

Operational optimization for N_2O mitigation can introduce trade-offs with energy use, effluent quality, and even other GHG emissions. Multi-objective optimization studies have shown that reducing N_2O emissions through tighter process control may increase energy consumption or operational costs, and in some cases, may shift emissions toward CH_4 if anaerobic conditions are inadvertently promoted (Sweetapple et al., 2014; Lu et al., 2024). For example, automatic control strategies can reduce GHG emissions by up to 9.6%, but achieving simultaneous improvements in effluent quality, operational cost, and GHG emissions is rarely possible—trade-offs must be carefully balanced (Sweetapple et al., 2014; Lu et al., 2024; Kim et al., 2015). Decision-makers must therefore consider site-specific priorities and constraints, employing multi-objective optimization to identify Pareto-optimal solutions that balance environmental and economic objectives.

Critical Evaluation and Gaps

While operational optimization—especially DO control and process stability—has proven effective, the high variability of N_2O emissions and the complexity of biological pathways present ongoing challenges. Advanced processes like anammox are promising but require careful integration and monitoring. Quantitative evidence suggests that while operational strategies can yield measurable reductions, the greatest GHG mitigation is

achieved when combined with broader energy-saving and resource recovery measures (Zhang et al., 2025). There is a need for more robust, real-time emission monitoring and predictive control systems, as well as further research into N_2O recovery and utilization, particularly in the context of economic and regulatory feasibility. The scaling-up of lab-scale mitigation technologies to full-scale WWTPs remains a significant challenge, necessitating site-specific validation and adaptation (Yao et al., 2022).

Reducing CH_4 Emissions through Operational Optimization and Emerging Technologies

Mechanisms and Key Emission Points

CH_4 is produced under anaerobic conditions by methanogenic archaea, primarily during sludge digestion, in anaerobic zones of treatment reactors, and in sewer networks (Song et al., 2023; Rodriguez-Caballero et al., 2014). While aerobic units generally have low CH_4 emissions, open anaerobic systems (e.g., stabilization ponds, UASB reactors) and poorly managed digesters are major sources (Song et al., 2023; Hwang et al., 2016). Recent studies highlight that CH_4 emissions from WWTPs can be persistent and relatively consistent day-to-day, with plant-scale emission rates varying within a factor of 1.6 to 3.7 across different operational and environmental conditions (Moore et al., 2023). However, significant variability can occur, especially when sampling is not sufficiently replicative or when operational anomalies arise.

Operational Optimization Strategies

a. Biogas Capture and Utilization

Capturing and utilizing biogas from sludge digesters is the most effective way to reduce CH_4 emissions, converting a potent GHG into renewable energy (Ma et al., 2017; Song et al., 2023; Guo et al., 2024). Nayeb et al. (2019) quantified that improved WWTP performance and increased public access to treatment can reduce CH_4 emissions by 60.4% to 84.3% compared to baseline scenarios, underscoring the substantial mitigation potential of biogas capture and utilization. Ensuring gas-tight digesters and minimizing fugitive emissions from storage and handling are critical (Song et al., 2023).

b. Process Design and Management

Minimizing unintended anaerobic conditions in aerobic reactors and sewer systems can prevent CH_4 formation (Rodriguez-Caballero et al., 2014). Rapid dewatering and stabilization of sludge reduce the window for anaerobic CH_4 production (Guo et al., 2024).

c. Sewer System Management and Advanced Mitigation Technologies

Recent research has highlighted the importance of sewer networks as significant CH_4 sources, especially in warm climates and with long retention times (Song et al., 2023; Zhu et al., 2024). Several emerging mitigation strategies have been proposed and tested:

Chemical Dosing: The addition of oxygen, ferric salts, hydroxide, free nitrous acid, or nitrite can suppress methanogenesis in sewers (Song et al., 2023; Zhu et al., 2024). However, these approaches may incur higher costs and potentially disrupt downstream WWTP operations.

Pipeline Maintenance: Regular cleaning of sewer pipelines reduces sediment accumulation, which is a key site for CH_4 production (Zhu et al., 2024).

Methanotrophic Biofilters: Engineered "end-of-pipe" solutions such as methanotrophic air biofilters are being developed to biologically oxidize CH_4 at sewer gas relief points and headspace ventilation points, targeting CH_4 hotspots (Song et al., 2023).

Sewer Aeration: Intermittent aeration can suppress methanogenesis, though this must be balanced against increased energy use (Song et al., 2023).

System Design: Reducing hydraulic retention times and optimizing WWTP capacity planning can limit CH_4 formation in extensive sewer networks (Zhu et al., 2024).

d. Dissolved Methane Recovery

A significant fraction of CH_4 generated in anaerobic digesters remains dissolved in the reject wastewater stream. Technologies such as membrane separation have been proposed for dissolved methane recovery, though their economic viability at scale remains to be fully demonstrated (Ribera-Guardia et al., 2019; Song et al., 2023). Covering reactors and digestate storage tanks to minimize leaks and upgrading treatment processes for higher CH_4 recovery are also recommended (Song et al., 2023).

e. Monitoring and Hotspot Identification

Continuous, long-term monitoring campaigns are essential to characterize CH_4 emission variability and to link emissions to process parameters or performance indicators (Song et al., 2023). The development of advanced sensors, machine learning models, and tailored analytical methods is expected to improve detection and enable targeted mitigation.

Quantitative Comparison of Mitigation Potential and Case Studies

Scenario modeling by Nayeb et al. (2019) demonstrates that upgrading WWTPs and expanding access to treatment can reduce CH_4 emissions by up to 84% compared to baseline, highlighting the critical role of operational optimization and infrastructure improvement. In multi-objective optimization case studies, Sweetapple et al. (2014) found that GHG emissions (including CH_4) could be reduced by up to 17.4% while also cutting operational costs by 3.6%, though further reductions often required trade-offs with effluent quality. These findings illustrate the practical potential and limitations of operational and technological interventions for CH_4 mitigation.

Economic and Practical Feasibility

The economic feasibility of CH_4 mitigation strategies is influenced by both initial investment and long-term operational savings. While biogas capture systems and infrastructure upgrades require capital expenditure, they can yield substantial returns through renewable energy generation and reduced energy costs (Cairone et al., 2024). However, barriers such as technology bottlenecks, higher investments, and operational complexity—particularly in developing countries—can hinder widespread adoption (He et al., 2019). The transition to advanced, energy-efficient processes is further complicated by the need for skilled personnel and supportive policy frameworks. For sewer-based interventions, costs and potential operational impacts must be carefully weighed against emission reduction benefits (Zhu et al., 2024).

Policy and Regulatory Frameworks

Policy mechanisms play a pivotal role in driving CH_4 mitigation. Carbon pricing, green finance, and regulatory mandates are increasingly being adopted in various regions to incentivize biogas recovery and

infrastructure upgrades (Molinos-Senante et al., 2014; Yao et al., 2022). For example, carbon taxes in Canada and carbon neutrality goals in over 130 countries are fostering investment in CH_4 mitigation technologies (Yao et al., 2022). However, the effectiveness of these policies depends on local economic conditions, transaction costs, and the ability to balance environmental and operational objectives.

Trade-Offs and Unintended Consequences

Operational strategies to reduce CH_4 emissions can sometimes conflict with other objectives. For instance, increasing aeration to suppress methanogenesis in sewers or reactors may raise energy consumption and operational costs (Sweetapple et al., 2014; Kim et al., 2015). Multi-objective optimization studies reveal that improvements in GHG emissions may come at the expense of higher costs or, in some cases, effluent quality (Lu et al., 2024). Moreover, shifting toward anaerobic treatment for energy recovery can inadvertently increase fugitive CH_4 emissions if gas capture is incomplete (Song et al., 2023; Moore et al., 2023). Thus, a balanced, site-specific approach is essential.

Critical Evaluation and Gaps

Biogas recovery is a mature and effective mitigation strategy, but fugitive emissions from digesters and sewers remain problematic, especially in older or poorly maintained infrastructure. There is a trade-off between maximizing energy/resource recovery and minimizing CH_4 leakage, particularly as anaerobic technologies are increasingly adopted for sustainability (Song et al., 2023). Quantitative evidence underscores the high mitigation potential of operational optimization and infrastructure improvement, but practical implementation is often constrained by economic and technical barriers. At the urban scale, studies have shown that wastewater infrastructure can be the dominant source of CH_4 emissions in some cities, emphasizing the need for targeted mitigation in these systems (Fernandez et al., 2022). Emerging technologies such as dissolved methane recovery and methanotrophic biofilters offer promising avenues, but require further demonstration and economic assessment.

Comparing the Feasibility and Difficulty of CH_4 and N_2O Detection and Prevention

Detection

N_2O Detection:

Challenges: N_2O emissions are highly variable in time and space, with sharp peaks linked to process events (Ribera-Guardia et al., 2019; Rodriguez-Caballero et al., 2014). Direct measurement is technically challenging and costly, often requiring high-sensitivity analyzers and complex sampling setups (Khalil et al., 2023; Yoshida et al., 2014). The dynamic nature of WWTP operations further complicates the implementation of reliable monitoring systems (Shang et al., 2024).

Methodological Limitations: There is no standardized methodology for N_2O monitoring in full-scale systems (Vasilaki et al., 2019). Emission factors or periodic sampling are commonly used but can miss emission peaks and underrepresent true emissions (Blomberg et al., 2018; Hwangbo et al., 2021). Calibration, maintenance, and interpretation of high-frequency data require significant expertise and resources (Kosse et al., 2018).

Advances: Real-time, plant-integrated measurement systems and machine learning models are emerging to improve detection and prediction (Yoshida et al., 2014; Khalil et al., 2023; Zhang et al., 2025). Ground-based remote sensing and hyperspectral techniques offer high spatial resolution and sensitivity, but require careful calibration and validation (Galfalk et al., 2022).

CH_4 Detection:

Challenges: CH_4 emissions are more spatially distributed, with significant contributions from both treatment units and collection systems (Song et al., 2023; Chong et al., 2024). Detection is complicated by low background concentrations and the need to distinguish between biogenic and fossil sources. Methodological disparities between static chambers, remote sensing, and flux chambers can lead to significant differences in reported emissions (Galfalk et al., 2022; Reddy et al., 2022).

Current Practice: Flux chambers, off-gas collection, and plant-integrated measurements are used, but may underestimate emissions if not all sources are captured (Chong et al., 2024). Fixed emission factors are sometimes used, but can lead to underestimation of true emissions (Kosse et al., 2018). Recent large-scale

studies have shown that current IPCC-based inventories, which rely on limited field measurements and expert assessment, significantly underestimate sector-wide CH_4 emissions (Moore et al., 2023; Song et al., 2023).
Advances: High-sensitivity, real-time analyzers and plant-wide emission inventories are improving accuracy (Yoshida et al., 2014; Song et al., 2023). Satellite-based remote sensing is increasingly able to detect large CH_4 emission events, though detection limits remain relatively high (Biener et al., 2024).
Limitations and Uncertainties in GHG Detection and Emission Factor Methodologies:
Emission Factor Uncertainties: Current methods for calculating N_2O and CH_4 emissions often rely on fixed emission factors (EFs) derived from limited field data, which do not account for differences in process configuration, operational conditions, or climate (Ramírez-Melgarejo et al., 2020; Li et al., 2024). For example, CH_4 EFs are typically based on default maximum CH_4-producing capacity and country-specific correction factors, without distinguishing treatment technologies (Li et al., 2024). For N_2O, IPCC guidelines estimate emissions based on population or per-capita factors, which can diverge significantly from plant-level, technology-specific measurements (Li et al., 2024). Monte Carlo simulations and probabilistic approaches are increasingly used to quantify uncertainty, revealing that emission estimates can vary widely depending on the methodology and input data (Ramírez-Melgarejo et al., 2020; Li et al., 2024; Sweetapple et al., 2013).
Sampling Duration and Methodology: The duration and frequency of sampling campaigns significantly influence EF estimates, with short-term campaigns often missing episodic emission events (Ramírez-Melgarejo et al., 2020). Methodological differences between empirical and theoretical approaches further complicate comparative evaluation and mitigation planning.
Impact on Mitigation Planning: The use of outdated or non-representative EFs can lead to substantial underestimation of sector-wide emissions, misallocation of mitigation resources, and missed opportunities for targeted interventions (Moore et al., 2023; Song et al., 2023; Li et al., 2024). There is a pressing need for updated, measurement-informed EFs that reflect the diversity of treatment processes and regional conditions.
Prevention

N_2O Prevention: Requires tight process control (especially DO and nitrogen loading), rapid response to process upsets, and advanced process configurations (e.g., anammox). Prevention is complicated by the multiplicity of biological pathways and the sensitivity to operational fluctuations (Blomberg et al., 2018; Lei et al., 2025; Shang et al., 2024). Innovative technologies such as membrane reactors, chemical inhibitors, and cycle optimization can further reduce emissions, but require site-specific validation and careful integration (Yao et al., 2022; Vasilaki et al., 2019).

CH_4 Prevention: More straightforward in principle—capture and utilize biogas, prevent anaerobic conditions in unintended locations, and maintain infrastructure integrity. However, system-wide prevention is complicated by diffuse sources (e.g., sewers) and the need for continuous monitoring (Song et al., 2023; Zhu et al., 2024). Emerging approaches such as methanotrophic biofilters, chemical dosing, and dissolved methane recovery offer new opportunities but require further demonstration and economic assessment.

Comparative Summary

Both gases are challenging to monitor, but N_2O is more temporally variable and sensitive to process dynamics, making real-time detection more critical and complex. CH_4 is more spatially distributed, requiring comprehensive system-wide monitoring and targeted interventions. Methodological limitations, calibration, and data interpretation challenges are significant for both gases, and integrating multiple measurement approaches is recommended. Notably, recent research has highlighted that sector-wide CH_4 emissions are likely underestimated due to insufficient process-level data and reliance on outdated emission factors, especially for diverse plant types and climates (Moore et al., 2023; Noyola et al., 2018; Song et al., 2023). Prevention of N_2O is more process-sensitive and technologically demanding, while CH_4 prevention is more infrastructure- and management-dependent.

CH_4 Emission Sources in the Wastewater Treatment System and Their Proportions

Main Sources and Proportions by Plant Type and Climate

Recent large-scale studies and reviews have provided more granular insights into CH_4 emission sources, their proportions, and the influence of plant type and climate:

Anaerobic Digesters: The largest point source, especially if biogas is not fully captured or digesters are not gas-tight. Plant-scale studies show that digesters can account for 40 – 60% of total CH_4 emissions in conventional WWTPs (Song et al., 2023; Moore et al., 2023).

Sludge Handling and Storage: CH_4 can be emitted during sludge thickening, dewatering, and storage if anaerobic conditions persist, contributing 10 – 20% of total emissions (Song et al., 2023).

Sewer Networks: Significant CH_4 production can occur in gravity sewers, especially in warm climates and long retention times. Recent urban-scale analyses indicate that sewers can contribute 20 – 40% of total CH_4 emissions, and in some cities, wastewater infrastructure (including sewers) is the dominant urban CH_4 source (Fernandez et al., 2022; Zhu et al., 2024).

Treatment Reactors:

Anaerobic Reactors (e.g., UASB, stabilization ponds): Major CH_4 emitters due to intentional anaerobic conditions, with open systems potentially accounting for up to 50% of emissions in certain plant configurations (Song et al., 2023).

Aerobic Reactors: Generally low CH_4 emissions, but can emit if anaerobic microzones develop (<5% of total emissions).

Constructed Wetlands and Lagoons: Deep or poorly aerated systems can emit substantial CH_4, particularly in developing regions (Shukuru et al., 2025).

Influence of Plant Type and Climate:

CH_4 emission factors and source proportions vary significantly by treatment type and climate (Moore et al., 2023; Noyola et al., 2018; Song et al., 2023). Conventional activated sludge systems may underestimate CH_4 emissions due to unaccounted emissions from sewers and primary clarifiers, especially in warmer climates where higher temperatures increase CH_4 production (Noyola et al., 2018). Anaerobic reactors and open

stabilization ponds are associated with higher emissions, particularly from dissolved CH_4 in effluent that is not captured (Song et al., 2023; Ribera-Guardia et al., 2019).

Sector-Wide Underestimation and Data Gaps:

Current IPCC-based inventories, which rely on limited field measurements and expert assessment, significantly underestimate sector-wide CH_4 emissions (Moore et al., 2023; Song et al., 2023; Li et al., 2024). Technology-specific, plant-level emission factors derived from direct measurement are needed to improve accuracy and inform mitigation planning.

Tabulated/Graphical Data:

The literature consistently reports the following approximate source proportions for CH_4 emissions in conventional WWTPs (Song et al., 2023; Moore et al., 2023; Noyola et al., 2018):

Source	Proportion of Total CH_4 Emissions (Typical Range)
Anaerobic digesters	40 – 60%
Sludge handling/storage	10 – 20%
Sewer networks	20 – 40% (higher in warm climates)
Anaerobic reactors/ponds	Up to 50% (in plants with such units)
Aerobic reactors	<5%
Constructed wetlands/lagoons	Variable, can be dominant in some regions

Plant-Scale and Urban-Scale Variability:

Replicative sampling at U.S. WWTPs shows that day-to-day CH_4 emissions are generally consistent, within a factor of 1.6 of the plant-averaged emission rate, but can vary up to a factor of 3.7 under different environmental and operational conditions (Moore et al., 2023). Urban-scale studies reveal that wastewater infrastructure can be the primary source of anthropogenic CH_4 emissions in some cities (Fernandez et al., 2022).

Critical Evaluation

The relative contributions of CH_4 sources depend heavily on plant configuration, operational practices, climate, and infrastructure integrity. Anaerobic digesters and sewers are consistently identified as the largest sources, but open systems (ponds, lagoons) can dominate in certain contexts, especially in warm climates. Accurate quantification remains challenging due to spatial and temporal variability, and mitigation efforts must be tailored to the specific emission profile of each facility. The importance of replicative and multi-method sampling is increasingly recognized for robust emission inventories (Moore et al., 2023; Galfalk et al., 2022). There is a pressing need for updated, measurement-informed emission factors that reflect the diversity of treatment processes and regional conditions (Moore et al., 2023; Song et al., 2023; Li et al., 2024).

Synthesis and Future Directions

Key Insights

Wastewater treatment plants (WWTPs) present significant opportunities for greenhouse gas mitigation through operational optimization, advanced technologies, and strategic policy frameworks, with operational measures like dissolved oxygen control, process stability, and biogas management serving as the foundation for reducing both N_2O and CH_4 emissions by 10-18%, while more substantial infrastructure upgrades and energy-saving measures can achieve reductions up to 84% for methane and 43% for total GHGs. Advanced biological processes including anammox and shortcut nitrogen removal, membrane technologies, plant-wide modeling, and emerging methane-specific mitigation strategies such as chemical dosing in sewers, methanotrophic biofilters, and dissolved methane recovery are expanding the available mitigation toolbox beyond traditional biogas capture approaches. However, significant research gaps remain in real-time plant-wide monitoring systems, integration of predictive modeling and machine learning into operational control, systematic studies on methane emissions from sewers and sludge handling, and the development of updated, measurement-informed emission factors that address current methodological uncertainties that often lead to underestimation of actual emissions. Moving forward, successful GHG mitigation in WWTPs requires a multi-objective optimization approach that balances emission reduction with effluent quality and operational costs, supported

by robust monitoring systems, economic feasibility studies, lifecycle analyses, and policy frameworks that incentivize both emission reduction and energy recovery while ensuring financial sustainability, ultimately necessitating the integration of advanced process control, infrastructure maintenance, and adaptive management strategies with replicative, multi-method monitoring approaches to achieve accurate emission quantification and effective mitigation planning.

Conclusion

Reducing GHG emissions from wastewater treatment requires a holistic, system-wide approach that integrates operational optimization, advanced process technologies, comprehensive monitoring, and supportive policy frameworks. Quantitative evidence underscores the importance of combining operational strategies with infrastructure upgrades and energy-saving measures to achieve substantial emission reductions. While significant progress has been made in understanding and mitigating N_2O and CH_4 emissions, ongoing research, innovation, and policy support are essential to achieve net-zero goals and sustainable urban water management. The integration of real-time data, predictive control, and energy/resource recovery—balanced with economic and practical considerations—will be key to the next generation of climate-resilient WWTPs. Methodological rigor, updated emission factors, and multi-scale, replicative monitoring are critical for accurate emission inventories and effective mitigation, ensuring that WWTPs can transition from GHG sources to net reducers in the urban environment.

References

Yoshida, H et al. (2014). Plant-integrated measurement of greenhouse gas emissions from a municipal wastewater treatment plant. WATER RES.

Song, CH et al. (2023). Methane Emissions from Municipal Wastewater Collection and Treatment Systems. ENVIRON SCI TECHNOL.

Khalil, M et al. (2023). Machine learning for modeling N_2O emissions from wastewater treatment plants: Aligning model performance, complexity, and interpretability. WATER RES.
Rodriguez-Caballero, A et al. (2014). Evaluation of process conditions triggering emissions of green-house gases from a biological wastewater treatment system. SCI TOTAL ENVIRON.
Shukuru, BN et al. (2025). Mitigating CH_4 and N_2O emissions from domestic and industrial wastewater. RENEW SUST ENERG REV.
Ma, R et al. (2017). Reduction of greenhouse gases emissions during anoxic wastewater treatment by strengthening nitrite-dependent anaerobic methane oxidation process. BIORESOURCE TECHNOL.
Valkova, T et al. (2021). A method to estimate the direct nitrous oxide emissions of municipal wastewater treatment plants based on the degree of nitrogen removal. J ENVIRON MANAGE.
He, YY et al. (2023). Net-zero greenhouse gas emission from wastewater treatment: Mechanisms, opportunities and perspectives. RENEW SUST ENERG REV.
Maktabifard, M et al. (2023). Net-zero carbon condition in wastewater treatment plants: A systematic review of mitigation strategies and challenges. RENEW SUST ENERG REV.
Blomberg, K et al. (2018). Development of an Extended ASM3 Model for Predicting the Nitrous Oxide Emissions in a Full-Scale Wastewater Treatment Plant. ENVIRON SCI TECHNOL.
Guo, DT et al. (2024). A system engineering perspective for net zero carbon emission in wastewater and sludge treatment industry: A review. SUSTAIN PROD CONSUMP.
Du, R et al. (2022). A review of enhanced municipal wastewater treatment through energy savings and carbon recovery to reduce discharge and CO_2 footprint. BIORESOURCE TECHNOL.
Shahid, MK et al. (2023). Comprehensive analysis of greenhouse gas emissions and emission factors in a Korean domestic wastewater treatment plant: Insights into mechanisms and pathways for climate change mitigation. J WATER PROCESS ENG.
Lei, TY et al. (2025). Dynamically predicting nitrous oxide emissions in a full-scale industrial activated sludge reactor under multiple aeration patterns and COD/N ratios. WATER RES.

Hwang, KL et al. (2016). Characteristics of methane and nitrous oxide emissions from the wastewater treatment plant. BIORESOURCE TECHNOL.
Chong, YT et al. (2024). More applicable quantification of non-CO2 greenhouse gas emissions from wastewater treatment plants by on-site plant-integrated measurements. SCI TOTAL ENVIRON.
Ribera-Guardia, A et al. (2019). Nitrous oxide and methane emissions from a plug-flow full-scale bioreactor and assessment of its carbon footprint. J CLEAN PROD.
Hwangbo, S et al. (2021). Integrated Model for Understanding N2O Emissions from Wastewater Treatment Plants: A Deep Learning Approach. ENVIRON SCI TECHNOL.
Nayeb, H et al. (2019). Estimating greenhouse gas emissions from Iran's domestic wastewater sector and modeling the emission scenarios by 2030. J CLEAN PROD.
Solí s, B et al. (2022). A plant-wide model describing GHG emissions and nutrient recovery options for water resource recovery facilities. WATER RES.
Zhang, ZT et al. (2025). Comprehensive assessment, intelligent prediction, and precise mitigation strategies for greenhouse gas emissions in full-scale wastewater treatment plants. ENVIRON RES.
Molinos-Senante, M et al. (2014). Economic and environmental performance of wastewater treatment plants: Potential reductions in greenhouse gases emissions. RESOUR ENERGY ECON.
Cairone, S et al. (2024). Revolutionizing wastewater treatment toward circular economy and carbon neutrality goals: Pioneering sustainable and efficient solutions for automation and advanced process control with smart and cutting-edge technologies. J WATER PROCESS ENG.
Shang, ZX et al. (2024). Direct and indirect monitoring methods for nitrous oxide emissions in full-scale wastewater treatment plants: A critical review. J ENVIRON MANAGE.
He, Y et al. (2019). Assessment of energy consumption of municipal wastewater treatment plants in China. J CLEAN PROD.
Moore, DP et al. (2023). Underestimation of Sector-Wide Methane Emissions from United States Wastewater Treatment. ENVIRON SCI TECHNOL.

Fernandez, JM et al. (2022). Street-level methane emissions of Bucharest, Romania and the dominance of urban wastewater.. ATMOS ENVIRON-X.
Yao, XC et al. (2024). Optimizing water and nitrogen management to balance greenhouse gas emissions and yield in Chinese rice paddies. FIELD CROP RES.
Yao, H et al. (2022). Contribution of nitrous oxide to the carbon footprint of full-scale wastewater treatment plants and mitigation strategies- a critical review. ENVIRON POLLUT.
Vasilaki, V et al. (2019). A decade of nitrous oxide (N2O) monitoring in full-scale wastewater treatment processes: A critical review. WATER RES.
Document ID: 10.2166/wcc.2024.603
Galfalk, M et al. (2022). Ground-based remote sensing of CH4 and N2O fluxes from a wastewater treatment plant and nearby biogas production with discoveries of unexpected sources. ENVIRON RES.
Biener, KJ et al. (2024). Temporal Variation and Persistence of Methane Emissions from Shallow Water-Oil and Gas Production in the Gulf of Mexico. ENVIRON SCI TECHNOL.
Kosse, P et al. (2018). Quantifying direct carbon dioxide emissions from wastewater treatment units by nondispersive infrared sensor (NDIR) - A pilot study. SCI TOTAL ENVIRON.
Noyola, A et al. (2018). Methane correction factors for estimating emissions from aerobic wastewater treatment facilities based on field data in Mexico and on literature review. SCI TOTAL ENVIRON.
Yang, MJ et al. (2023). Greenhouse gas emissions from wastewater treatment plants in China: Historical emissions and future mitigation potentials. RESOUR CONSERV RECY.
Sweetapple, C et al. (2014). Multi-objective optimisation of wastewater treatment plant control to reduce greenhouse gas emissions. WATER RES.
Lu, H et al. (2024). Deep Learning-Based Multiobjective Optimization for Balancing Effluent Quality, Operational Cost, and Greenhouse Gas Emissions in Wastewater Treatment Plant Control. ACS EST WATER.
Kim, D et al. (2015). Optimization of wastewater treatment plant operation for greenhouse gas mitigation. J ENVIRON MANAGE.

Zhu, HT et al. (2024). Closing the gap in methane emission from urban wastewater sewer system in China. J CLEAN PROD.
Ramírez-Melgarejo, M et al. (2020). Analysis of empirical methods for the quantification of N2O emissions in wastewater treatment plants: Comparison of emission results obtained from the IPCC Tier 1 methodology and the methodologies that integrate operational data. SCI TOTAL ENVIRON.
Li, HY et al. (2024). Methane and nitrous oxide emissions from municipal wastewater treatment plants in China: A plant-level and technology-specific study. ENVIRON SCI ECOTECH.
Sweetapple, C et al. (2013). Identifying key sources of uncertainty in the modelling of greenhouse gas emissions from wastewater treatment. WATER RES.

Question: Please provide a comprehensive review on "Ammonia-oxidizing microorganisms and their N2O emission in wastewater". The review should include the following characteristics:
1. Key characteristics and advantages of different Nitrifiers
2. Mechanistic/genetic level differences leading to N_2O production

GPT4.1

1. Key Characteristics and Advantages
1.1 Ammonia-Oxidizing Bacteria (AOB)
Key Characteristics:
- **Taxonomy:** Belong mainly to the Betaproteobacteria (e.g., Nitrosomonas, Nitrosospira).
- **Metabolism:** Oxidize ammonia (NH_3) to nitrite (NO_2^-) via hydroxylamine (NH_2OH) as an intermediate.

- **Optimal Conditions:** Prefer neutral to slightly alkaline pH (7–8.5) and moderate to high ammonia concentrations.
- **Growth Rate:** Generally higher than AOA and comammox under typical wastewater conditions.
- **Oxygen Requirement:** Obligate aerobes; sensitive to low dissolved oxygen (DO).
Advantages:
- **Robustness:** Well-adapted to high-ammonia, high-organic-load environments typical of municipal and industrial wastewater.
- **Established Role:** Long history of successful application in conventional activated sludge and biofilm systems.
1.2 Ammonia-Oxidizing Archaea (AOA)
Key Characteristics:
- **Taxonomy:** Belong to the phylum Thaumarchaeota (e.g., Nitrosopumilus, Nitrososphaera).
- **Metabolism:** Similar pathway to AOB but with distinct enzymes and regulatory mechanisms.
- **Optimal Conditions:** Thrive in low-ammonia, low-organic-load, and often low-DO environments.
- **Growth Rate:** Slower than AOB; more competitive under oligotrophic (nutrient-poor) conditions.
- **Oxygen Requirement:** Can tolerate lower DO than AOB.
Advantages:
- **Niche Adaptation:** Can dominate in systems with low ammonia concentrations, such as tertiary treatment or advanced nutrient removal processes.

- **Energy Efficiency:** Potentially more energy-efficient ammonia oxidation, leading to lower oxygen demand.
1.3 Complete Ammonia Oxidizers (Comammox)
Key Characteristics:
- **Taxonomy:** Belong to the genus Nitrospira (e.g., Nitrospira inopinata).
- **Metabolism:** Capable of oxidizing ammonia directly to nitrate (NO_3^-) within a single organism, bypassing the need for separate AOB and nitrite-oxidizing bacteria (NOB).
- **Optimal Conditions:** Prefer low-ammonia, low-DO, and biofilm-associated environments.
- **Growth Rate:** Generally slower than AOB and NOB, but highly efficient at low substrate concentrations.
Advantages:
- **Process Simplification:** Potential to streamline nitrification by reducing the number of functional groups required.
- **Efficiency:** High substrate affinity allows effective ammonia removal at low concentrations, beneficial for polishing effluents.
---
2. Mechanistic and Genetic-Level Differences Leading to N_2O Production
2.1 Pathways of N_2O Production in Nitrifiers
N_2O can be produced during nitrification via several mechanisms:
a) Hydroxylamine Oxidation Pathway (AOB-dominated)
- **Mechanism:** During the oxidation of hydroxylamine (NH_2OH) to nitrite, incomplete oxidation can result in the release of N_2O.
- **Enzymes Involved:** Hydroxylamine oxidoreductase (HAO) and ammonia monooxygenase (AMO).

- **Genetic Markers:** hao, amoA genes.
b) Nitrifier Denitrification (AOB and some comammox)
- **Mechanism:** Under low-oxygen or anoxic conditions, AOB can reduce nitrite to N_2O via nitric oxide (NO) as an intermediate.
- **Enzymes Involved:** Nitrite reductase (NirK), nitric oxide reductase (Nor).
- **Genetic Markers:** nirK, norB genes.
c) Abiotic Reactions
- **Mechanism:** Chemical decomposition of intermediates (e.g., NH_2OH, NO_2^-) can also lead to N_2O formation, especially under acidic or high-nitrite conditions.
2.2 Differences Among Nitrifiers
Ammonia-Oxidizing Bacteria (AOB)
- **N_2O Production:** Major contributors to N_2O emissions in wastewater treatment.
- **Genetic Basis:** Possess both the canonical ammonia oxidation pathway and genes for partial denitrification (nirK, norB).
- **Regulation:** N_2O production is enhanced under low DO, high nitrite, and high ammonia conditions.
- **Mechanistic Nuance:** AOB can produce N_2O both via hydroxylamine oxidation (even under aerobic conditions) and via nitrifier denitrification (under oxygen-limited conditions).
Ammonia-Oxidizing Archaea (AOA)
- **N_2O Production:** Generally produce less N_2O than AOB.
- **Genetic Basis:** Lack canonical denitrification genes (e.g., nirK, norB) found in AOB; N_2O production is thought to be minimal and may occur via alternative or less efficient pathways.
- **Mechanistic Nuance:** The exact mechanisms are less well understood, but AOA are not considered major contributors to N_2O emissions in wastewater systems.

Comammox
- **N_2O Production:** Limited data, but initial studies suggest lower N_2O yields compared to AOB.
- **Genetic Basis:** Possess genes for complete ammonia oxidation but may lack or have reduced expression of denitrification genes.
- **Mechanistic Nuance:** N_2O production is likely associated with the hydroxylamine oxidation step, but the overall contribution is thought to be minor.
2.3 Environmental and Operational Factors
- **Dissolved Oxygen (DO):** Low DO increases N_2O production, especially via nitrifier denitrification in AOB.
- **Ammonia and Nitrite Concentrations:** High substrate concentrations can lead to increased N_2O yields.
- **pH and Temperature:** Acidic pH and higher temperatures can enhance N_2O production.
- **Organic Carbon:** Presence of organic carbon can stimulate heterotrophic denitrification, which may also contribute to N_2O emissions.

Conclusion
Ammonia-oxidizing microorganisms are central to nitrogen removal in wastewater treatment systems. AOB are the dominant nitrifiers in most systems, especially under high-ammonia conditions, but AOA and comammox play important roles in specific niches, particularly at low ammonia concentrations. The production of N_2O, a potent greenhouse gas, is primarily associated with AOB through both the hydroxylamine oxidation pathway and nitrifier denitrification, with genetic and mechanistic differences underpinning these processes. Understanding the ecology, physiology, and genetics of these nitrifiers is essential for

optimizing nitrogen removal and minimizing greenhouse gas emissions in wastewater treatment.

References
1. Daims, H., et al. (2015). Complete nitrification by Nitrospira bacteria. Nature, 528(7583), 504–509.
2. Stein, L. Y. (2019). Insights into the physiology of ammonia-oxidizing microorganisms. Current Opinion in Chemical Biology, 49, 9–15.
3. Kozlowski, J. A., et al. (2016). Comammox Nitrospira: A new player in the nitrogen cycle. Trends in Microbiology, 24(9), 702–712.
4. Yu, R., et al. (2010). Mechanisms and specificities of N_2O production during ammonia oxidation by Nitrosomonas europaea. Water Research, 44(7), 1953–1962.
5. Prosser, J. I., & Nicol, G. W. (2012). Archaeal and bacterial ammonia-oxidisers in soil: The quest for niche specialisation and differentiation. Trends in Microbiology, 20(11), 523–531.

GPT4.1 Search

Comprehensive Review: Ammonia-Oxidizing Microorganisms and Their N_2O Emission in Wastewater

---

1. Introduction

Nitrous oxide (N_2O) is a potent greenhouse gas with a global warming potential approximately 298 times that of CO_2 over a 100-year period (IPCC, 2021). Wastewater treatment plants

(WWTPs) are recognized as significant anthropogenic sources of N_2O, primarily due to microbial nitrogen transformations. Among the key players are ammonia-oxidizing microorganisms (AOMs), including ammonia-oxidizing bacteria (AOB), ammonia-oxidizing archaea (AOA), and, more recently, complete ammonia oxidizers (comammox). Understanding the emission characteristics, mechanisms, and mitigation strategies for N_2O in these systems is crucial for sustainable wastewater management.

---

2. N_2O Emission Characteristics and Mechanisms

2.1. Microbial Pathways of N_2O Production

N_2O production in WWTPs is mainly attributed to two biological processes: nitrification and denitrification. AOMs, especially AOB, are central to N_2O emissions during nitrification. Two primary mechanisms are recognized:

- **Hydroxylamine Oxidation Pathway:** During the oxidation of ammonia (NH_3) to nitrite (NO_2^-), hydroxylamine (NH_2OH) is an obligate intermediate. Incomplete oxidation of NH_2OH can lead to N_2O formation, especially under suboptimal oxygen conditions (Kampschreur et al., 2009; Stein, 2011).
- **Nitrifier Denitrification:** Under low dissolved oxygen (DO) or high nitrite concentrations, AOB can reduce nitrite to N_2O via nitric oxide (NO) as an intermediate (Wrage et al., 2001; Stein, 2011).

AOA and comammox have also been implicated in N_2O production, but their quantitative contributions remain less well characterized (Kits et al., 2017; Wang et al., 2022).

2.2. Emission Characteristics

N_2O emissions from WWTPs are highly variable, ranging from 0.01% to 25% of influent nitrogen load, depending on operational conditions (Ahn et al., 2010; Kampschreur et al., 2009). Key influencing factors include:

- **Dissolved Oxygen (DO):** Low DO promotes nitrifier denitrification, while high DO can suppress N_2O production but may increase NH_2OH oxidation-derived N_2O (Law et al., 2012).
- **Nitrite Accumulation:** Elevated NO_2^- concentrations enhance N_2O production via both AOB and heterotrophic denitrifiers (Kampschreur et al., 2009).
- **pH and Temperature:** Lower pH and higher temperatures generally increase N_2O yields (Desloover et al., 2012).

2.3. Quantitative Data

Ahn et al. (2010) reported N_2O emission factors of 0.01–1.8% of influent nitrogen in full-scale WWTPs, with peaks during transient conditions such as start-up or process disturbances. Kampschreur et al. (2009) highlighted that up to 90% of N_2O can be produced during the nitrification phase, particularly under low DO.

---

3. Optimal Process Configurations

3.1. Conventional Nitrification-Denitrification

Traditional activated sludge processes with separate aerobic and anoxic zones are widely used. However, these systems can exhibit significant N_2O emissions, especially during periods of nitrite accumulation or oxygen limitation (Ahn et al., 2010).

3.2. Shortcut Nitrogen Removal (e.g., Partial Nitritation-Anammox)

Shortcut processes, such as partial nitritation-anammox (PN/A), offer energy and carbon savings by bypassing nitrate formation. While anammox bacteria do not produce N_2O, partial nitritation by AOB can still generate N_2O, particularly under low DO or high NO_2^- (Desloover et al., 2012; Lackner et al., 2014).

3.3. Emerging Configurations

- **Granular Sludge Systems:** Aerobic granular sludge can enhance process stability and reduce N_2O emissions by maintaining higher DO gradients and better substrate diffusion (Pronk et al., 2015).
- **Integrated Fixed-Film Activated Sludge (IFAS):** Biofilm-based systems can support more stable microbial communities, potentially reducing transient N_2O peaks (Wang et al., 2022).

---

4. Feasibility of Emission Reduction Strategies and Technologies

4.1. Process Control Strategies

- **DO Setpoint Optimization:** Maintaining DO at optimal levels (typically 1–2 mg/L) can minimize both NH_2OH oxidation and nitrifier denitrification pathways (Law et al., 2012).
- **Nitrite Management:** Avoiding nitrite accumulation through process control or step-feed strategies reduces N_2O production (Kampschreur et al., 2009).

4.2. Advanced Monitoring and Modeling

Real-time N_2O monitoring and dynamic process modeling enable early detection of emission peaks and facilitate responsive control (Foley et al., 2010). However, implementation at full scale remains limited by sensor reliability and cost.

4.3. Microbial Community Engineering

Selective enrichment of low N_2O-producing AOB or promotion of comammox, which may have lower N_2O yields, is a promising but nascent approach (Kits et al., 2017; Wang et al., 2022).

4.4. Practical Implications and Limitations

While many strategies demonstrate efficacy at lab and pilot scale, full-scale implementation faces challenges:

- **Operational Complexity:** Tight process control requires advanced automation and skilled operators.
- **Cost:** Upgrading sensors, control systems, or retrofitting reactors can be expensive.
- **Trade-offs:** Some strategies (e.g., higher DO) may increase energy consumption, offsetting climate benefits (Foley et al., 2010).

---

5. Research Gaps and Future Directions

Despite advances, several gaps remain:

- **Quantifying AOA and Comammox Contributions:** Most studies focus on AOB, with limited data on the roles of AOA and comammox in N_2O dynamics (Kits et al., 2017).
- **Mechanistic Understanding at Full Scale:** Laboratory findings need validation in complex, dynamic full-scale systems.
- **Integrated Assessment:** Life cycle and techno-economic analyses are needed to balance emission reductions with energy and cost implications (Foley et al., 2010).
- **Microbial Community Manipulation:** More research is needed on engineering microbial consortia for lower N_2O emissions.

---

6. Synthesis and Conclusions

Ammonia-oxidizing microorganisms are central to N_2O emissions in wastewater treatment, with emission characteristics strongly influenced by operational and environmental factors. While process optimization and advanced monitoring offer promising mitigation avenues, practical implementation is constrained by technical and economic barriers. Future research should prioritize full-scale validation, integrated assessment, and microbial community engineering to achieve sustainable N_2O management in WWTPs.

References

- Ahn, J. H., Kim, S., Park, H., Rahm, B., Pagilla, K., & Chandran, K. (2010). N2O emissions from activated sludge processes, 2008–2009: Results of a national monitoring survey in the United States. *Environmental Science & Technology*, 44(12), 4505–4511. https://pubs.acs.org/doi/10.1021/es903845y
- Desloover, J., Vlaeminck, S. E., Clauwaert, P., Verstraete, W., & Boon, N. (2012). Strategies to mitigate N2O emissions from biological nitrogen removal systems. *Current Opinion in Biotechnology*, 23(3), 474–482. https://doi.org/10.1016/j.copbio.2011.11.008
- Foley, J., de Haas, D., Yuan, Z., & Lant, P. (2010). Nitrous oxide generation in full-scale biological nutrient removal wastewater treatment plants. *Water Research*, 44(3), 831–844. https://doi.org/10.1016/j.watres.2009.10.013

- IPCC (2021). Climate Change 2021: The Physical Science Basis. Contribution of Working Group I to the Sixth Assessment Report of the Intergovernmental Panel on Climate Change. https://www.ipcc.ch/report/ar6/wg1/
- Kampschreur, M. J., Temmink, H., Kleerebezem, R., Jetten, M. S. M., & van Loosdrecht, M. C. M. (2009). Nitrous oxide emission during wastewater treatment. *Water Research*, 43(17), 4093–4103. https://doi.org/10.1016/j.watres.2009.03.001
- Kits, K. D., Sedlacek, C. J., Lebedeva, E. V., Han, P., Bulaev, A., Pjevac, P., ... & Daims, H. (2017). Kinetic analysis of a complete nitrifier reveals an oligotrophic lifestyle. *Nature*, 549(7671), 269–272. https://www.nature.com/articles/nature23679
- Lackner, S., Gilbert, E. M., Vlaeminck, S. E., Joss, A., Horn, H., & van Loosdrecht, M. C. M. (2014). Full-scale partial nitritation/anammox experiences – An application survey. *Water Research*, 55, 292–303. https://doi.org/10.1016/j.watres.2014.02.032
- Law, Y., Ye, L., Pan, Y., & Yuan, Z. (2012). Nitrous oxide emissions from wastewater treatment processes. *Philosophical Transactions of the Royal Society B: Biological Sciences*, 367(1593), 1265–1277. https://doi.org/10.1098/rstb.2011.0317
- Pronk, M., de Kreuk, M. K., de Bruin, L. M., Kamminga, P., Kleerebezem, R., van Loosdrecht, M. C. M. (2015). Full scale performance of the aerobic granular sludge process for sewage treatment. *Water Research*, 84, 207–217. https://doi.org/10.1016/j.watres.2015.07.011

- Stein, L. Y. (2011). Surveying N2O-producing pathways in bacteria. *Methods in Enzymology*, 486, 131–152. https://doi.org/10.1016/B978-0-12-381294-0.00006-9<br>- Wang, Y., Ma, L., Mao, Y., Jiang, X., Xia, Y., Yu, K., & Zhang, T. (2022). Comammox in drinking water systems: Occurrence, abundance, and activity. *Water Research*, 215, 118247. https://doi.org/10.1016/j.watres.2022.118247<br>- Wrage, N., Velthof, G. L., van Beusichem, M. L., & Oenema, O. (2001). Role of nitrifier denitrification in the production of nitrous oxide. *Soil Biology and Biochemistry*, 33(12–13), 1723–1732. https://doi.org/10.1016/S0038-0717(01)00096-7
**NaiveRAG**
Comprehensive Review: Ammonia-Oxidizing Microorganisms/Organisms in Wastewater Treatment Systems<br>#### 1. Key Characteristics and Advantages of Different Nitrifiers<br>**A. Ammonia-Oxidizing Bacteria (AOB)**<br>- **Role:** AOB are the primary microorganisms responsible for the oxidation of ammonia (NH_4^+) to nitrite (NO_2^-) in conventional nitrification processes in wastewater treatment plants (WWTPs) [12, 39, 53].<br>- **Metabolism:** They are autotrophic, using CO_2 as a carbon source and ammonia as an energy source [4, 12].<br>- **Environmental Preferences:** AOB thrive under aerobic conditions, but can also contribute to N_2O production under low dissolved oxygen (DO) or high nitrite conditions [37, 53].<br>- **Advantages:** Well-established in conventional activated sludge systems; robust under a range of operational conditions [12, 39].

- **Limitations:** Significant contributors to N_2O emissions, especially under suboptimal oxygen or nitrite-accumulating conditions [53].
B. Ammonia-Oxidizing Archaea (AOA)
- **Role:** AOA can also oxidize ammonia to nitrite, particularly in systems with low DO and long solids retention times [4, 12].
- **Metabolism:** Similar to AOB, but often more competitive under low ammonia and low DO conditions [4].
- **Advantages:** Higher affinity for ammonia and oxygen, potentially leading to lower N_2O emissions compared to AOB [40].
- **Limitations:** Generally less abundant and less studied in WWTPs; their contribution to N_2O emissions is considered minor [22, 40].
C. Nitrite-Oxidizing Bacteria (NOB)
- **Role:** NOB oxidize nitrite (NO_2^-) to nitrate (NO_3^-), completing the nitrification process [12, 39].
- **Metabolism:** Autotrophic, using nitrite as an energy source [4].
- **Advantages:** Essential for full nitrification; their suppression is desirable in shortcut nitrogen removal processes (e.g., partial nitritation/anammox) [8].
- **Limitations:** Compete with AOB for oxygen; their activity can be detrimental in processes aiming for shortcut nitrogen removal [8].
D. Anammox Bacteria
- **Role:** Anaerobic ammonium oxidation (anammox) bacteria convert ammonia and nitrite directly to dinitrogen gas (N_2) under anoxic conditions [8, 17].
- **Metabolism:** Obligate anaerobes; autotrophic [8].

- **Advantages:** Lower oxygen and organic carbon requirements, reduced sludge production, and lower N_2O emissions compared to conventional nitrification-denitrification [8, 17, 40].
- **Limitations:** Slow growth rates and sensitivity to oxygen [8].
E. Comammox Bacteria
- **Role:** Recently discovered, these bacteria can oxidize ammonia directly to nitrate in a single organism [26].
- **Advantages:** Potential for more efficient and stable nitrification, possibly with lower N_2O emissions [40].
- **Limitations:** Still under investigation; operational advantages and N_2O emission profiles require further study [40].
F. Heterotrophic Ammonia Oxidizers
- **Role:** Can oxidize ammonia under high organic loading and low DO, but at much lower rates than autotrophic nitrifiers [4].
- **Advantages:** May become significant under specific conditions (high COD/N, low DO) [4].
- **Limitations:** Not major contributors in conventional systems [4].
2. Mechanistic/Genetic Level Differences Leading to N_2O Production
A. Main Biological Pathways for N_2O Production
- **Hydroxylamine (NH_2OH) Oxidation:** AOB and AOA oxidize ammonia to hydroxylamine, which can be further oxidized to nitrite. N_2O can be produced as a byproduct, especially if NH_2OH accumulates [22, 25, 33, 53].
- **Nitrifier Denitrification:** Under low DO or high nitrite, AOB can reduce nitrite to N_2O using ammonia or other electron donors. This pathway is catalyzed by AOB-encoded nitrite reductase (NirK) and nitric oxide reductase (Nor) [39, 41, 55].

- **Heterotrophic Denitrification:** Facultative heterotrophic bacteria reduce nitrate/nitrite to N_2 via N_2O as an intermediate. N_2O can accumulate if the final step (N_2O to N_2) is inhibited, often due to low carbon or oxygen presence [55].
B. Genetic and Enzymatic Factors
- **nosZ Gene:** Encodes nitrous oxide reductase, the only enzyme capable of reducing N_2O to N_2, thus acting as a sink for N_2O. The abundance and activity of nosZ-containing bacteria are critical for minimizing net N_2O emissions [1, 7].
- **nirK and nor Genes:** Encode nitrite and nitric oxide reductases, respectively, in AOB and denitrifiers. Their expression and activity determine the rate and extent of N_2O production via nitrifier denitrification and heterotrophic denitrification [39, 41].
- **Environmental Regulation:** Expression of these genes and the activity of their associated enzymes are strongly influenced by DO, nitrite concentration, pH, and carbon availability [30, 45, 55].
C. Mechanistic Differences Among Nitrifiers
- **AOB:** Major contributors to N_2O via both hydroxylamine oxidation and nitrifier denitrification, especially under low DO and high nitrite [22, 39, 53].
- **AOA:** Less significant in N_2O production; their metabolic pathways are less prone to N_2O accumulation [22, 40].
- **Anammox:** Generally produce much less N_2O, making them advantageous for low-emission nitrogen removal [17, 40].
- **Comammox:** Early evidence suggests lower N_2O emissions, but more research is needed [40].
D. Impact of Microbial Community Structure

- The relative abundance of AOB, AOA, anammox, and nosZ-containing bacteria determines the net N_2O emission potential of a system. Enriching N_2O-reducing bacteria (via bioaugmentation or operational strategies) can significantly reduce emissions [7, 1, 15].

References

1. Zhao, YW et al. (2024). CURR RES BIOTECHNOL. [1, 3, 20, 26, 30]
2. Yang, R et al. (2023). J WATER PROCESS ENG. [2]
3. Kampschreur, MJ et al. (2009). WATER RES. [4]
4. Xu, PP et al. (2021). J ENVIRON MANAGE. [8, 12, 23]
5. An, ZM et al. (2024). BIORESOURCE TECHNOL. [7, 15, 17]
6. Castellano-Hinojosa, A et al. (2018). CHEMOSPHERE. [18, 37]
7. Rodriguez-Caballero, A et al. (2013). BIORESOURCE TECHNOL. [39, 41]
8. Vasilaki, V et al. (2019). WATER RES. [22]
9. Wunderlin, P et al. (2012). WATER RES. [10, 11, 43, 55]
10. Quan, XC et al. (2012). WATER RES. [45]
11. Wang, DB et al. (2016). ENVIRON SCI TECHNOL. [49]
12. Duan, HR et al. (2021). ENVIRON SCI TECHNOL. [40]
13. Li, JG et al. (2023). CHEM ENG J. [52]
14. Massara, TM et al. (2017). SCI TOTAL ENVIRON. [46]
15. Aboobakar, A et al. (2013). WATER RES. [53, 57]
16. Ni, BJ et al. (2015). WATER RES. [33]
17. Lee, YJ et al. (2022). BIORESOURCE TECHNOL. [28]
18. Wang, YY et al. (2016). CHEM ENG J. [6, 13, 31]
19. Toor, UA et al. (2015). INT BIODETER BIODEGR. [35]
20. Pan, K et al. (2024). J CLEAN PROD. [50]

WaterRAG Initial review

Ammonia-Oxidizing Microorganisms in Wastewater Treatment Systems: Key Characteristics, Advantages, and Mechanistic Insights into N_2O Production
---
1. Introduction
Ammonia oxidation is a cornerstone of biological nitrogen removal (BNR) in wastewater treatment plants (WWTPs), historically attributed to two distinct microbial guilds: ammonia-oxidizing bacteria (AOB) and nitrite-oxidizing bacteria (NOB). Recent discoveries have expanded this paradigm to include ammonia-oxidizing archaea (AOA) and complete ammonia-oxidizing (comammox) bacteria, fundamentally altering our understanding of nitrification ecology, process optimization, and greenhouse gas (GHG) emissions, particularly nitrous oxide (N_2O). This review synthesizes current knowledge on the key characteristics and advantages of different nitrifiers, and critically examines the mechanistic and genetic bases underlying their N_2O production in wastewater systems.
---
2. Key Characteristics and Advantages of Different Nitrifiers
2.1. Canonical Ammonia-Oxidizing Bacteria (AOB)
Ecology and Physiology:
AOB, mainly from the Betaproteobacteriales genera *Nitrosomonas* and *Nitrosospira*, have long been considered the dominant ammonia oxidizers in municipal WWTPs (Cotto et al., 2020; Han et al., 2019). They initiate nitrification by oxidizing ammonia (NH_3) to nitrite (NO_2^-) via ammonia monooxygenase (AMO) and hydroxylamine dehydrogenase (HAO).
Advantages:
- **High Growth Rates:** AOB generally exhibit faster growth than comammox and AOA, especially under high ammonia and oxygen conditions (Mehrani et al., 2020).

- **Process Robustness:** Their resilience to operational fluctuations makes them reliable in conventional, well-mixed activated sludge systems.
Limitations:
- **Lower Substrate Affinity:** AOB have relatively low affinity for ammonia and oxygen, making them less competitive under low substrate conditions (Xu et al., 2022; He et al., 2023).
- **High N_2O Emissions:** AOB are significant contributors to N_2O emissions via both hydroxylamine oxidation and nitrifier denitrification pathways (Liu et al., 2021; Lin et al., 2022).
2.2. Ammonia-Oxidizing Archaea (AOA)
Ecology and Physiology:
AOA, first discovered in marine and soil environments, are now recognized in engineered systems, including WWTPs and drinking water plants (Al-Ajeel et al., 2022). They possess distinct AMO enzymes and often dominate in low-ammonia, low-oxygen, or extreme environments.
Advantages:
- **High Substrate Affinity:** AOA have higher affinities for ammonia and oxygen than AOB, enabling them to thrive in oligotrophic and low-DO environments (Xu et al., 2022; He et al., 2023).
- **Low N_2O Emissions:** AOA generally emit less N_2O compared to AOB, attributed to their lack of canonical nitric oxide reductase (NOR) genes and alternative metabolic pathways (Liu et al., 2021; Liu et al., 2024).
Limitations:
- **Uncertain Contribution:** Despite their abundance, the actual contribution of AOA to ammonia oxidation in WWTPs is variable and sometimes limited, as their activity does not always correlate with gene abundance (Al-Ajeel et al., 2022).

2.3. Complete Ammonia-Oxidizing (Comammox) Bacteria
Discovery and Ecology:
Comammox bacteria, primarily within the *Nitrospira* genus (lineage II), can oxidize ammonia directly to nitrate (NO_3^-) in a single organism (Daims et al., 2015; van Kessel et al., 2015). Their discovery overturned the two-step nitrification paradigm and revealed new complexity in nitrogen cycling (Cotto et al., 2020; Roots et al., 2019).
Key Characteristics:
- **Metabolic Versatility:** Comammox possess genes for urea and cyanate degradation, formate and hydrogen oxidation, indicating broad metabolic capabilities (Xu et al., 2022; Palomo et al., 2018).
- **High Substrate Affinity:** They exhibit the highest known affinities for ammonia and oxygen among nitrifiers, enabling dominance in low-ammonia, low-DO, and long solids retention time (SRT) systems (Cotto et al., 2020; Roots et al., 2019; Zheng et al., 2023).
- **Slow Growth, High Yield:** Comammox grow more slowly than AOB but have higher biomass yields, favoring biofilm or attached-growth systems with low substrate flux (Mehrani et al., 2020).
- **Mixotrophy:** Some comammox can utilize organic substrates, supporting synergistic interactions with heterotrophs and denitrifiers (Xu et al., 2022).
Advantages in Wastewater Treatment:
- **Effluent Quality:** Systems dominated by comammox can achieve lower effluent ammonia concentrations and higher overall nitrification activity (Xu et al., 2022; Zheng et al., 2023).
- **GHG Mitigation:** Comammox-dominated systems exhibit lower direct (N_2O) and indirect (via reduced aeration) GHG emissions (Xu et al., 2022; He et al., 2023; Liu et al., 2024).

- **Process Stability:** Their high substrate affinity and metabolic versatility confer resilience to fluctuating operational conditions, particularly in advanced and low-DO BNR systems (Cotto et al., 2020; Roots et al., 2019).

Limitations:

- **Competition in High-Substrate Systems:** In high-ammonia, well-mixed systems, faster-growing AOB may outcompete comammox (Mehrani et al., 2020).
- **Potential Process Detriment:** In processes requiring nitrite accumulation (e.g., partial nitritation-anammox), comammox can be detrimental by converting nitrite to nitrate, undermining process goals (Roots et al., 2019).

2.4. Ammonia-Oxidizing Microbial Community Interactions

Niche Differentiation:

The prevalence and activity of AOB, AOA, and comammox are shaped by environmental parameters such as pH, DO, SRT, temperature, and influent characteristics (Xu et al., 2022; Cotto et al., 2020; He et al., 2022). For example, comammox dominate in low-DO, low-ammonia, and attached-growth systems, while AOB prevail in high-load, well-mixed reactors.

Functional Redundancy and Complementarity:

Recent studies highlight that numerical abundance does not always equate to functional dominance; gene expression and enzymatic activity must be considered (Zheng et al., 2023). Stable isotope probing and meta-omics approaches are essential for disentangling the actual contributions of each group (Al-Ajeel et al., 2022; Zheng et al., 2023).

---

3. Mechanistic and Genetic Differences Leading to N_2O Production

3.1. Pathways of N_2O Production in Nitrifiers

N_2O, a potent greenhouse gas, is produced during nitrification via two main pathways:

- **Hydroxylamine Oxidation Pathway:**

During ammonia oxidation, hydroxylamine (NH_2OH) is an obligate intermediate. Its incomplete oxidation can abiotically or enzymatically yield N_2O (Liu et al., 2021; Lin et al., 2022).
- **Nitrifier Denitrification Pathway:**
Under low-oxygen or high-nitrite conditions, some nitrifiers reduce nitrite or nitric oxide (NO) to N_2O using denitrification enzymes (Liu et al., 2021; Liu et al., 2024).
3.2. Genetic and Mechanistic Differences among Nitrifiers
AOB:
- **Genetic Basis:**
AOB possess canonical nitric oxide reductase (NOR) genes, enabling them to reduce NO to N_2O via nitrifier denitrification (Liu et al., 2021; Liu et al., 2024).
- **High N_2O Emission:**
Both hydroxylamine oxidation and denitrification contribute to high N_2O yields, especially under low DO or high nitrite (Lin et al., 2022; Liu et al., 2021).
- **Quantitative Data:**
N_2O emission factors for AOB are typically higher than those for AOA or comammox, with emissions increasing under suboptimal aeration or nitrite accumulation (Liu et al., 2021; He et al., 2023).
AOA:
- **Genetic Basis:**
AOA generally lack canonical NOR genes (Liu et al., 2021; Liu et al., 2024). However, N_2O can still be produced abiotically from NH_2OH or NO, or via alternative, less efficient enzymatic routes (Liu et al., 2021).
- **Low N_2O Emission:**

Pure culture and environmental studies consistently show lower N_2O emission factors for AOA compared to AOB (Liu et al., 2021; Liu et al., 2024).
- **Mechanistic Uncertainties:**
Some AOA (e.g., *Nitrosocosmicus oleophilus*) may produce N_2O via nitrifier denitrification under excess nitrite or low pH, but this is not widespread (Liu et al., 2021).
Comammox:
- **Genetic Basis:**
Comammox Nitrospira lack the genes for canonical NOR and other denitrification enzymes, precluding the nitrifier denitrification pathway (Liu et al., 2024; Lin et al., 2022; He et al., 2023).
- **Very Low N_2O Emission:**
N_2O production is limited to minor abiotic routes from hydroxylamine or NO intermediates, resulting in much lower N_2O emissions than AOB (Xu et al., 2022; Lin et al., 2022; Liu et al., 2024).
- **Process Implications:**
Systems dominated by comammox can achieve significant GHG mitigation, making them attractive for sustainable wastewater management (He et al., 2023; Liu et al., 2024).
3.3. Practical Implications and Quantitative Comparisons
- **Operational Strategies:**
Lowering DO can reduce AOB-driven N_2O emissions but may shift community composition toward AOA or comammox, further reducing GHG output (Liu et al., 2021; Xu et al., 2022).
- **Process Design:**
Integrating comammox or AOA as primary ammonia oxidizers, particularly in anammox-driven or low-DO systems, can enhance nitrogen removal efficiency and minimize N_2O emissions (He et al., 2023; Lin et al., 2022; Liu et al., 2024).

- **Quantitative Data:**
Studies report that comammox- and AOA-dominated systems have N_2O emission factors several-fold lower than AOB-dominated systems (Liu et al., 2021; He et al., 2023).
---
4. Critical Evaluation and Research Gaps
4.1. Current State of Knowledge
- **Community Dynamics:**
There is growing evidence that comammox Nitrospira can numerically and functionally dominate ammonia oxidation in many full-scale WWTPs, especially under low-DO, long SRT, and attached-growth conditions (Zheng et al., 2023; Cotto et al., 2020; Xu et al., 2022).
- **Functional Redundancy:**
The actual contribution of each nitrifier group is context-dependent and cannot be inferred from abundance alone; activity-based assays and meta-omics are essential (Zheng et al., 2023; Al-Ajeel et al., 2022).
- **GHG Emissions:**
Mechanistic understanding of N_2O production is robust for AOB but remains limited for AOA and comammox, though the latter are consistently associated with lower emissions (Liu et al., 2021; He et al., 2023).
4.2. Research Gaps and Future Directions
- **Mechanistic Studies on Comammox and AOA:**
While genetic analyses suggest low N_2O emission potential, direct quantification and mechanistic studies under realistic WWTP conditions are needed (He et al., 2023; Lehtovirta-Morley et al., 2018).
- **Ecophysiological Diversity:**

The diversity of comammox and AOA in different wastewater environments, and their responses to operational perturbations, require further exploration (He et al., 2022; Zheng et al., 2023).

- **Process Integration:**

Strategies for selectively enriching comammox or AOA in full-scale systems, and understanding their interactions with anammox and denitrifiers, are underdeveloped (Xu et al., 2022; He et al., 2023).

- **Advanced Methodologies:**

Application of stable isotope probing, meta-transcriptomics, and functional gene assays will be crucial for resolving the activity and ecological roles of different nitrifiers (Annavajhala et al., 2018; Zheng et al., 2023).

---

5. Synthesis and Conclusions

The discovery and characterization of comammox bacteria and AOA have revolutionized our understanding of nitrification in wastewater treatment. Comammox Nitrospira, with their high substrate affinity, metabolic versatility, and low N_2O emission potential, are emerging as key players in advanced BNR systems, particularly under low-DO and low-ammonia conditions. AOA, while less well understood in engineered systems, also offer advantages in terms of substrate affinity and GHG mitigation.

Mechanistically, the absence of canonical denitrification genes in comammox and most AOA underpins their low N_2O emissions, in stark contrast to AOB. This genetic distinction has profound implications for process design and sustainability, as the selection or enrichment of low-N_2O-emitting nitrifiers can contribute to net-zero GHG goals in wastewater treatment.

However, significant research gaps remain, particularly regarding the in situ activity, regulation, and ecological interactions of comammox and AOA. Future research should

prioritize functional assays, advanced -omics, and process-scale studies to harness the full potential of these microorganisms for sustainable nitrogen removal and climate mitigation in wastewater treatment.

WaterRAG Final review

1. Introduction
Ammonia oxidation is a cornerstone of biological nitrogen removal (BNR) in wastewater treatment plants (WWTPs), historically attributed to two distinct microbial guilds: ammonia-oxidizing bacteria (AOB) and nitrite-oxidizing bacteria (NOB). Recent discoveries have expanded this paradigm to include ammonia-oxidizing archaea (AOA) and complete ammonia-oxidizing (comammox) bacteria, fundamentally altering our understanding of nitrification ecology, process optimization, and greenhouse gas (GHG) emissions, particularly nitrous oxide (N_2O). This review synthesizes current knowledge on the key characteristics and advantages of different nitrifiers, critically examines the mechanistic and genetic bases underlying their N_2O production in wastewater systems, and integrates recent advances in quantitative emission data, environmental and operational controls, detection methodologies, the impact of emerging process configurations, and regulatory and monitoring strategies. Additionally, it addresses the influence of co-occurring microbial processes, the impact of environmental inhibitors, economic and practical feasibility of community manipulation, and highlights emerging molecular tools for in situ functional analysis. Recent literature is integrated to provide explicit quantitative comparisons, mechanistic insights, and a nuanced understanding of diversity, detection, and process configuration impacts.

---

2. Key Characteristics and Advantages of Different Nitrifiers
2.1. Canonical Ammonia-Oxidizing Bacteria (AOB)
Ecology and Physiology:

AOB, mainly from the Betaproteobacteriales genera *Nitrosomonas* and *Nitrosospira*, have long been considered the dominant ammonia oxidizers in municipal WWTPs (Cotto et al., 2020; Han et al., 2019). They initiate nitrification by oxidizing ammonia (NH_3) to nitrite (NO_2^-) via ammonia monooxygenase (AMO) and hydroxylamine dehydrogenase (HAO).

Kinetic and Physiological Parameters:

AOB typically exhibit higher maximum specific growth rates (μ_max) than AOA and comammox, especially under high ammonia and oxygen conditions (Mehrani et al., 2020; Law et al., 2019). Their substrate affinities are lower: for instance, *Nitrosomonas europaea* displays ammonium half-saturation constants (K_m) in the range of 0.077–0.55 mg N/L and oxygen K_m values of 0.3–0.8 mg O_2/L (He et al., 2023). These relatively high K_m values indicate lower substrate affinity compared to AOA and comammox (Lehtovirta-Morley et al., 2018; Jung et al., 2022).

Intra-group Diversity and Functional Redundancy:

AOB communities in full-scale WWTPs exhibit significant diversity and functional redundancy, which is thought to confer process stability (Siripong & Rittmann, 2007). Despite a relatively restricted number of known AOB species, community structures across different plants often show significant similarity, suggesting that operational stability is supported by both diversity and redundancy.

Advantages:

- **High Growth Rates:** AOB generally exhibit faster growth than comammox and AOA, especially under high ammonia and oxygen conditions (Mehrani et al., 2020).
- **Process Robustness:** Their resilience to operational fluctuations makes them reliable in conventional, well-mixed activated sludge systems.

Limitations:

- **Lower Substrate Affinity:** AOB have relatively low affinity for ammonia and oxygen, making them less competitive under low substrate conditions (Xu et al., 2022; He et al., 2023; Lehtovirta-Morley et al., 2018).
- **High N_2O Emissions:** AOB are significant contributors to N_2O emissions via both hydroxylamine oxidation and nitrifier denitrification pathways (Liu et al., 2021; Lin et al., 2022). Quantitatively, N_2O emission yields from pure cultures of AOB vary from 0.05% to 3.3% of oxidized ammonia (Kim et al., 2010; Colliver & Stephenson, 2000; Yoshida, 1988), with recent studies reporting yields around 0.51% under standardized conditions (An et al., 2024). In full-scale WWTPs, emission factors can range from 0.018% to over 5% of influent nitrogen load, depending on process configuration and operational parameters (Yao et al., 2022; Valkova et al., 2021; Massara et al., 2017). Notably, batch and continuous process modeling reveal high variability in key parameters such as the half-saturation constant for nitrite and the reduction factor for N_2O production, which can influence emission estimates by more than 100% across studies (Ni et al., 2015).
2.2. Ammonia-Oxidizing Archaea (AOA)
Ecology and Physiology:
AOA, first discovered in marine and soil environments, are now recognized in engineered systems, including WWTPs and drinking water plants (Al-Ajeel et al., 2022). They possess distinct AMO enzymes and often dominate in low-ammonia, low-oxygen, or extreme environments.
Kinetic and Physiological Parameters:
AOA are characterized by exceptionally high substrate affinities. For example, *Nitrosopumilus maritimus* exhibits ammonium K_m values as low as 0.0016–0.002 mg N/L and oxygen K_m values of 0.064–0.12 mg O_2/L (He et al., 2023). However, recent work has demonstrated a broader range of substrate affinities among AOA, with K_m(NH_3) values

spanning from 3.6 nM to 4.4 μM, reflecting significant variability within the group (Jung et al., 2022; Lehtovirta-Morley et al., 2018). This variability is influenced by phylogeny, cell morphology, and environmental factors such as pH and temperature, which can modulate kinetic properties and niche differentiation (Jung et al., 2022; Al-Ajeel et al., 2022).
Intra-group Diversity and Ecological Differentiation:
AOA display considerable diversity, with different lineages adapted to specific niches within engineered systems (Al-Ajeel et al., 2022). Their abundance and activity can vary widely between plants and operational regimes, and their contribution to ammonia oxidation is not always proportional to their gene abundance (Castellano-Hinojosa et al., 2018).
Advantages:
- **High Substrate Affinity:** AOA have higher affinities for ammonia and oxygen than AOB, enabling them to thrive in oligotrophic and low-DO environments (Xu et al., 2022; He et al., 2023; Lehtovirta-Morley et al., 2018).
- **Low N_2O Emissions:** AOA generally emit less N_2O compared to AOB, attributed to their lack of canonical nitric oxide reductase (NOR) genes and alternative metabolic pathways (Liu et al., 2021; Liu et al., 2024). Quantitative studies report N_2O yields in the range of 0.068–0.158% (An et al., 2024; Han et al., 2021), substantially lower than AOB.
Limitations:
- **Uncertain Contribution:** Despite their abundance, the actual contribution of AOA to ammonia oxidation in WWTPs is variable and sometimes limited, as their activity does not always correlate with gene abundance (Al-Ajeel et al., 2022). Methodological challenges in distinguishing AOA activity from that of AOB and comammox further complicate ecological assessments (Cotto et al., 2020).
2.3. Complete Ammonia-Oxidizing (Comammox) Bacteria
Discovery and Ecology:

Comammox bacteria, primarily within the *Nitrospira* genus (lineage II), can oxidize ammonia directly to nitrate (NO_3^-) in a single organism (Daims et al., 2015; van Kessel et al., 2015). Their discovery overturned the two-step nitrification paradigm and revealed new complexity in nitrogen cycling (Cotto et al., 2020; Roots et al., 2019).
Kinetic and Physiological Parameters:
Comammox Nitrospira, such as *N. inopinata*, exhibit the highest known substrate affinities among nitrifiers, with K_m(app) for NH_3 as low as 49 nM—lower than all characterized AOB and comparable to or even exceeding the affinity of some AOA (Lehtovirta-Morley et al., 2018; Jung et al., 2022). Their oxygen K_m values are also low, supporting their dominance in low-ammonia, low-DO, and long solids retention time (SRT) systems (He et al., 2023). Not all comammox or AOA possess extremely high substrate affinities, and environmental factors such as pH and temperature can modulate these properties (Jung et al., 2022).
Intra-group Diversity and Ecological Differentiation:
Comammox Nitrospira exhibit substantial diversity, with clade A and clade B lineages showing different distributions and ecological preferences (Al-Ajeel et al., 2022; Cotto et al., 2020). Their prevalence is strongly influenced by process configuration, SRT, and the presence of attached growth phases (Cotto et al., 2020; Zheng et al., 2023).
Key Characteristics:
- **Metabolic Versatility:** Comammox possess genes for urea and cyanate degradation, formate and hydrogen oxidation, indicating broad metabolic capabilities (Xu et al., 2022; Palomo et al., 2018).
- **High Substrate Affinity:** Their low K_m values for both ammonia and oxygen enable efficient substrate scavenging under oligotrophic conditions (Lehtovirta-Morley et al., 2018; Jung et al., 2022).

- **Slow Growth, High Yield:** Comammox grow more slowly than AOB but have higher biomass yields, favoring biofilm or attached-growth systems with low substrate flux (Mehrani et al., 2020).
- **Mixotrophy:** Some comammox can utilize organic substrates, supporting synergistic interactions with heterotrophs and denitrifiers (Xu et al., 2022).
Advantages in Wastewater Treatment:
- **Effluent Quality:** Systems dominated by comammox can achieve lower effluent ammonia concentrations and higher overall nitrification activity (Xu et al., 2022; Zheng et al., 2023).
- **GHG Mitigation:** Comammox-dominated systems exhibit lower direct (N_2O) and indirect (via reduced aeration) GHG emissions (Xu et al., 2022; He et al., 2023; Liu et al., 2024). Quantitative studies indicate N_2O yields of 0.07–0.2% (Han et al., 2021; An et al., 2024), and pilot-scale enrichment of comammox on biocarriers has been shown to decrease system-wide N_2O emissions by up to 36.8% (Tian et al., 2021). Their contribution to total NOy emissions is also relatively minor compared to AOB (Han et al., 2021).
- **Process Stability:** Their high substrate affinity and metabolic versatility confer resilience to fluctuating operational conditions, particularly in advanced and low-DO BNR systems (Cotto et al., 2020; Roots et al., 2019).
Limitations:
- **Competition in High-Substrate Systems:** In high-ammonia, well-mixed systems, faster-growing AOB may outcompete comammox (Mehrani et al., 2020).
- **Potential Process Detriment:** In processes requiring nitrite accumulation (e.g., partial nitritation-anammox), comammox can be detrimental by converting nitrite to nitrate, undermining process goals (Roots et al., 2019).
2.4. Quantitative Comparison of Kinetic and Physiological Parameters

A comprehensive, side-by-side comparison of kinetic parameters and N_2O emission yields for AOB, AOA, and comammox is summarized in Table 1. These data, derived from both pure cultures and mixed-culture studies under standardized conditions, highlight the niche differentiation among ammonia oxidizers and inform process optimization:

Group	Representative Organism	K_m (NH_3)	K_m (O_2)	μ_max (d^{-1})	N_2O Yield (% of NH_4^+ oxidized)	Reference
AOB	*Nitrosomonas europaea*	0.077–0.55 mg N/L	0.3–0.8 mg O_2/L	~0.8–1.0	0.05–3.3 (typ. ~0.5–2.9)	He et al., 2023; Kim et al., 2010; An et al., 2024
AOA	*Nitrosopumilus maritimus*	0.0016–0.002 mg N/L	0.064–0.12 mg O_2/L	~0.1–0.2	0.068–0.158	He et al., 2023; Han et al., 2021; An et al., 2024
Comammox	*Nitrospira inopinata*	49 nM (0.0007 mg N/L)	~0.1 mg O_2/L	~0.1–0.2	0.07–0.2	Lehtovirta-Morley et al., 2018; Han et al., 2021; An et al., 2024

Note: K_m values and μ_max are subject to environmental modulation (e.g., pH, temperature) and may vary among strains and operational conditions.

---

3. Impact of Operational Strategies and Process Configurations on Nitrifier Selection, Activity, and N_2O Emissions

Operational parameters such as dissolved oxygen (DO), solids retention time (SRT), aeration regime, and influent characteristics play pivotal roles in shaping nitrifier community structure, activity, and N_2O emissions. Recent studies have also highlighted the influence of process

configuration—mainstream vs. sidestream, shortcut nitrogen removal, and hybrid systems—on the selection and dominance of specific nitrifier groups.

Aeration Strategies:

Intermittent aeration has been shown to significantly reduce N_2O emissions compared to continuous aeration. For example, full-scale implementation of intermittent aeration resulted in approximately 90% lower N_2O emissions during test periods (Duan et al., 2021). In low C/N wastewater treatment, intermittent aeration not only increased total nitrogen removal efficiency (to ~93.5%) but also reduced the N_2O emission factor from 0.42% (continuous aeration) to 0.19% (Xie et al., 2023). Notably, intermittent aeration promoted the enrichment of comammox (gene abundance up to 24.7%) while reducing AOB abundance, aligning with the goal of minimizing N_2O emissions due to comammox's lack of NO reductase (Xie et al., 2023; Liu et al., 2017).

DO and SRT Manipulation:

Reducing DO from 1 to 0.5 mg/L can lower AOB growth rates by 3–26%, with a less pronounced effect on NOB (Ma et al., 2009). However, low DO may require longer SRTs (e.g., 15 days) to maintain stable ammonia oxidation, especially under variable loading (Ma et al., 2009). Importantly, low DO can also reduce N_2O emissions by limiting the activity of the nitrifier denitrification (ND) pathway, provided ammonia oxidation is not compromised (Duan et al., 2021). Conversely, excessively low DO may increase N_2O production if nitrite accumulates.

Process Configuration and Nitrifier Selection:

Comprehensive surveys of full-scale systems reveal that process configuration strongly influences nitrifier community structure (Cotto et al., 2020). Mainstream nitrification-denitrification (ND) and simultaneous nitrification-denitrification (SND) systems with long SRTs and attached growth phases favor comammox dominance, while sidestream partial

nitritation-anammox (PNA) systems select for AOB and suppress comammox due to high substrate concentrations and the need for nitrite accumulation. This ecological differentiation is reflected in N_2O emission profiles, with comammox-dominated systems exhibiting lower emissions (An et al., 2024; Tian et al., 2021).
Case Studies and Quantitative Outcomes:
Pilot and full-scale studies consistently demonstrate that operational strategies and process configurations favoring comammox or AOA (e.g., low DO, intermittent aeration, long SRT, attached growth) lead to lower N_2O emissions and improved nitrogen removal efficiency (Xie et al., 2023; Duan et al., 2021; Cotto et al., 2020; Tian et al., 2021).
---
4. Influence of Environmental Inhibitors and Ecological Differentiation
Environmental inhibitors such as free ammonia (FA), free nitrous acid (FNA), heavy metals, and micropollutants can profoundly affect nitrifier activity, community structure, and N_2O emissions.
- **Free Ammonia (FA) and Free Nitrous Acid (FNA):**
  FA and FNA are known to inhibit both AOB and NOB, but AOB generally exhibit higher tolerance to FA, while NOB are more sensitive (Al-Ajeel et al., 2022). FNA can inhibit both AOB and comammox, potentially leading to nitrite accumulation and increased N_2O emissions. The sensitivity of AOA and comammox to these inhibitors is less well characterized but may be higher due to their lower K_m values and oligotrophic adaptations (Jung et al., 2022).
- **Heavy Metals and Micropollutants:**
  The presence of heavy metals and certain organic micropollutants can selectively inhibit nitrifier groups, alter community structure, and disrupt ammonia oxidation, potentially

increasing N_2O emissions due to incomplete nitrification or stress-induced denitrification (Al-Ajeel et al., 2022).

- **Ecological Differentiation and Diversity:**

The diversity within each nitrifier group, including functional redundancy among AOB and the presence of multiple comammox and AOA lineages, provides resilience to environmental stressors and operational perturbations (Siripong & Rittmann, 2007; Al-Ajeel et al., 2022). However, shifts in community structure (e.g., dominance of comammox or AOA) may occur under chronic exposure, influencing both process performance and N_2O emission profiles (Castellano-Hinojosa et al., 2018).

---

5. Quantitative Comparison of N_2O Emission Factors Across Nitrifiers

Recent advances have enabled rigorous, side-by-side comparisons of N_2O emission factors across AOB, AOA, and comammox under controlled conditions. For instance, Han et al. (2021) demonstrated that the N_2O yield for AOB (*N. europaea*) is the highest (typically 0.5–3.3%), while AOA (*N. gargensis*, *Ca. N. uzonensis*) and comammox (*N. inopinata*) exhibit much lower yields (0.068–0.2%). These findings are corroborated by An et al. (2024), who report N_2O yields of 0.51% for AOB, 0.068–0.158% for AOA, and 0.07–0.2% for comammox. In pilot-scale systems, enrichment of comammox on biocarriers reduced N_2O emissions by 36.8% (Tian et al., 2021). These explicit, standardized comparisons reinforce the importance of community composition in determining N_2O emission potential and provide a quantitative basis for process optimization.

---

6. Mechanistic and Genetic Differences Leading to N_2O Production

6.1. Pathways of N_2O Production in Nitrifiers

N_2O, a potent greenhouse gas, is produced during nitrification via two main pathways:

- **Hydroxylamine Oxidation Pathway:**

During ammonia oxidation, hydroxylamine (NH_2OH) is an obligate intermediate. Its incomplete oxidation can abiotically or enzymatically yield N_2O (Liu et al., 2021; Lin et al., 2022). Recent studies have shown that AOB can directly oxidize hydroxylamine to N_2O at elevated rates of ammonia oxidation, highlighting the importance of intermediates such as NH_2OH, NOH, and NO in dictating N_2O yields (Zhao et al., 2024).

- **Nitrifier Denitrification Pathway:**

Under low-oxygen or high-nitrite conditions, some nitrifiers reduce nitrite or nitric oxide (NO) to N_2O using denitrification enzymes (Liu et al., 2021; Liu et al., 2024). The dominance of this pathway in aerobic zones is supported by correlations between N_2O emissions, NO_2^- concentrations, and AOB abundance (Vasilaki et al., 2019).

6.2. Genetic and Enzymatic Mechanisms and Regulation

AOB:

- **Key Genes and Enzymes:**

AOB possess canonical genes encoding ammonia monooxygenase (amoA/B/C), hydroxylamine dehydrogenase (hao), and, in some strains, nitric oxide reductase (norB/norC), enabling both ammonia oxidation and nitrifier denitrification (Zhou et al., 2020; Zhao et al., 2024; Wang et al., 2024). The presence or absence of norB/norC is a key determinant of N_2O production potential (Han et al., 2021).

- **Gene Expression Dynamics:**

Recent transcriptomic and proteomic studies (Yu et al., 2018) reveal that AMO and NIR concentrations increase under transient and repeated anoxia, while HAO remains relatively stable. During oxic-to-anoxic transitions, upregulation of nirK is observed, facilitating NO production via nitrifier denitrification. Upon re-oxygenation, hao is upregulated, promoting N_2O production via the hydroxylamine oxidation pathway. Notably, amoA and hao exhibit

similar expression patterns, but only amoA shows significant long-term upregulation with repeated anoxic-oxic cycling, suggesting a key role in adaptation and stress response. The regulation of cytochromes (e.g., c-554, c-P460) further modulates electron flow and detoxification capacities, impacting N_2O yields.

- **Pathway Integration:**

  The interconnectedness of pathways via shared enzymes (e.g., HAO) complicates attribution of N_2O production to discrete routes, underscoring the value of multi-omics approaches for pathway resolution (Yu et al., 2018).

AOA:

- **Key Genes and Enzymes:**

  AOA possess distinct AMO and HAO homologs but generally lack canonical NOR genes (Liu et al., 2021; Liu et al., 2024). N_2O production is thus limited to abiotic or alternative enzymatic routes, with lower overall yields.

- **Emission Pathways:**

  Pure culture and environmental studies consistently show lower N_2O emission factors for AOA compared to AOB (Liu et al., 2021; Liu et al., 2024; Han et al., 2021). Some AOA may produce N_2O via alternative denitrification under specific conditions (e.g., excess nitrite, low pH), but this is not widespread.

Comammox:

- **Key Genes and Enzymes:**

  Comammox Nitrospira encode amoCAB, hao, and nxrAB for complete ammonia oxidation but lack canonical denitrification genes (e.g., norB, nosZ) (Liu et al., 2024; Lin et al., 2022; He et al., 2023; Zhang et al., 2023). Their N_2O production is limited to minor abiotic routes from hydroxylamine or NO intermediates, resulting in much lower N_2O emissions than AOB (Xu et al., 2022; Lin et al., 2022; Liu et al., 2024).

Summary Table of Mechanistic/Genetic Differences:

Group	Key Genes/Enzymes	N_2O Production Pathways	Typical Emission Factor	Reference
AOB	amoA/B/C, hao, norB/C	Hydroxylamine oxidation, ND	High (0.05–3.3% NH_4^+)	Kim et al., 2010; An et al., 2024; Han et al., 2021
AOA	amoA, hao (distinct forms)	Limited, mainly abiotic	Low (0.068–0.158%)	Han et al., 2021; An et al., 2024
Comammox	amoCAB, hao, nxrAB	Hydroxylamine oxidation	Very low (0.07–0.2%)	Han et al., 2021; An et al., 2024

ND: Nitrifier denitrification pathway.

---

7. Impact of Co-occurring Microbial Processes on N_2O Emissions

The interpretation of nitrifier-specific N_2O contributions in complex communities requires consideration of co-occurring processes such as heterotrophic denitrification, anammox, and dissimilatory nitrate reduction to ammonium (DNRA).

- **Anammox Systems:**

In anammox-based processes, AOB and denitrifiers are the main contributors to N_2O production, while anammox bacteria (AnAOB) can indirectly mitigate N_2O emissions by consuming NO and NO_2^- produced by denitrifiers (An et al., 2024; Lin et al., 2022; Lotti et al., 2014; Wan et al., 2021). AOB also protect AnAOB from DO toxicity, and denitrifiers facilitate nitrate reduction and protect AnAOB from organic carbon toxicity (Yan et al., 2010; Langone et al., 2014).

- **Heterotrophic Denitrification and DNRA:**

The presence and activity of denitrifiers lacking nosZ (e.g., Comamonadaceae) can lead to increased N_2O emissions, while denitrifiers with nosZ (e.g., Flavobacterium) are associated with lower emissions (Mao et al., 2022). The balance between these functional groups, as well as environmental variables such as C/N ratio and ammonium concentration, shapes the net N_2O flux.

- **Community Structure and Niche Partitioning:**

Network analyses reveal that specific nitrifiers (e.g., Nitrosomonas) are positively correlated with N_2O emissions, while certain denitrifiers can act as N_2O sinks or sources depending on their genetic repertoire (Mao et al., 2022). The diversity and redundancy within nitrifier and denitrifier communities contribute to process stability and resilience (Siripong & Rittmann, 2007).

---

8. Detection, Quantification, and Emerging Molecular Tools

Accurate detection and quantification of AOB, AOA, and comammox in situ are essential for understanding their ecological roles and contributions to N_2O emissions. Traditional methods include quantitative PCR (qPCR), fluorescence in situ hybridization (FISH), and metagenomics, each with specific limitations regarding primer specificity, probe coverage, and resolution (Cotto et al., 2020; Roots et al., 2019).

Advances and Limitations in Detection:

- **qPCR and Primer Specificity:**

qPCR assays targeting amoA, nxrB, and other functional genes are widely used, but primer specificity and coverage remain challenges, particularly for comammox, where primer sets may yield unspecific products or fail to capture the full diversity detected by metagenomics (Cotto et al., 2020; Roots et al., 2019).

- **FISH and Resolution:**

FISH provides spatial resolution but is limited by probe specificity and detection thresholds, especially for low-abundance groups.

- **Metagenomics and -Omics Approaches:**

Metagenomics and metatranscriptomics offer comprehensive profiling but are not ideally suited for high-throughput analysis of low-abundance nitrifiers due to cost and sequencing depth constraints (Cotto et al., 2020). However, these approaches enable the detection of functional gene expression and activity, as demonstrated by stable isotope probing and transcriptomic analyses (Zheng et al., 2023; Mehrani et al., 2020).

- **False Positives/Negatives and Activity Assessment:**

A persistent challenge is distinguishing presence from activity; gene abundance does not always correlate with functional contribution (Al-Ajeel et al., 2022; Castellano-Hinojosa et al., 2018). Multi-omics and stable isotope approaches are increasingly used to link metabolic activity to specific taxa, but standardization and integration with process data are needed for routine application.

---

9. Regulatory, Economic, and Practical Feasibility of N_2O Mitigation Strategies

Monitoring Approaches:

Extensive research has led to increasingly mature N_2O monitoring methods in full-scale WWTPs, including both direct (e.g., in situ sensors, gas-phase measurements) and indirect (e.g., emission factor calculations, modeling) approaches (Shang et al., 2024). The choice of method depends on the monitoring objective, with a trade-off between data resolution and practicality. The combined use of N_2O sensors and mass transfer models enhances understanding of N_2O generation and release dynamics.

Economic and Practical Feasibility:

Implementing N_2O mitigation strategies in full-scale WWTPs is complex, requiring flexibility to accommodate dynamic operational conditions and influent variability (Shang et al., 2024). Economic feasibility is critical; modifications must be justified by emission reductions and potential operational savings. Barriers include uncertainties in the effectiveness of lab-scale strategies at full scale, the applicability of mathematical models, potential impacts on nutrient removal, and additional operational costs (Duan et al., 2020). Regulatory and policy frameworks play a significant role in incentivizing and facilitating mitigation efforts, underscoring the need for supportive policies and standardized monitoring (Shang et al., 2024).
Best Practices and Policy Implications:
- **Site-Specific Monitoring and Pathway Identification:**
  Accurate quantification and identification of N_2O generation pathways are prerequisites for effective mitigation (Yao et al., 2022; Duan et al., 2020).
- **Operational Parameter Regulation:**
  Key strategies include optimizing aeration intensity and DO concentration, increasing COD/N and SRT, and regulating temperature and pH (Yao et al., 2022; Duan et al., 2020). Intermittent aeration and low DO control, initially validated in laboratory studies, have been shown to be effective at full scale (Duan et al., 2020).
- **Process Innovation:**
  Adoption of innovative BNR processes such as anammox-based systems and comammox-enriched configurations can achieve both nitrogen removal and N_2O mitigation (Yao et al., 2022; An et al., 2024).
- **Bioaugmentation and Community Engineering:**
  Enriching N_2O-reducing bacteria (e.g., nosZ-containing denitrifiers) and strategic adaptation of the microbial community can further reduce emissions (An et al., 2024).
- **Policy and Standardization:**

The establishment of supportive mitigation policies, standardization of sampling and monitoring, and integration of machine learning models for emission prediction are critical for scaling up mitigation efforts (Shang et al., 2024; Yao et al., 2022).
Implementation Framework:
A systematic approach for N_2O mitigation includes baseline monitoring, mechanistic interpretation, strategy evaluation (including modeling), and full-scale implementation, as demonstrated in recent long-term studies (Duan et al., 2020).
---
10. Contradictions, Uncertainties, and Research Gaps
Despite significant advances, several contradictions and uncertainties remain:
- **Ecological Roles and Emission Potentials:**
The distribution, abundance, and activity of AOA and comammox in engineered systems are influenced by complex and dynamic factors, leading to variable and sometimes conflicting findings (Al-Ajeel et al., 2022). While comammox and AOA are consistently associated with lower N_2O emissions, their absolute contributions remain uncertain due to methodological limitations and environmental variability (Han et al., 2021; Roots et al., 2019).
- **Detection and Quantification Challenges:**
The lack of highly specific molecular tools for distinguishing comammox from NOB, and the limitations of current qPCR and FISH probes, hinder accurate assessment of nitrifier community structure and function (Cotto et al., 2020; Roots et al., 2019). Advanced -omics approaches are promising but require further standardization and integration with process data.
- **Process-Scale Uncertainties:**
Translating laboratory findings to full-scale WWTPs is complicated by fluctuating influent characteristics, operational instability, and the inherent complexity of real-world systems (Zhao et al., 2024; Al-Ajeel et al., 2022).

- **Community Interactions:**

The impact of co-occurring microbial processes, such as heterotrophic denitrification and anammox, on observed N_2O emissions necessitates a systems-level perspective and integrated community management (An et al., 2024; Lin et al., 2022).

- **Environmental Inhibitors:**

The effects of FA, FNA, heavy metals, and micropollutants on the activity and N_2O production of each nitrifier group require further elucidation, particularly for AOA and comammox (Al-Ajeel et al., 2022).

- **Intra-group Diversity and Functional Redundancy:**

The ecological differentiation and redundancy within AOB, AOA, and comammox communities contribute to process stability but also complicate predictions of N_2O emission potential under changing operational regimes (Siripong & Rittmann, 2007; Al-Ajeel et al., 2022).

- **Economic and Practical Barriers:**

The economic and operational feasibility of manipulating nitrifier communities for N_2O mitigation at full scale remains a key challenge, with uncertainties in cost-benefit outcomes and regulatory support (Shang et al., 2024; Duan et al., 2020).

---

11. Synthesis and Conclusions

The discovery and characterization of comammox bacteria and AOA have revolutionized our understanding of nitrification in wastewater treatment. Comammox Nitrospira, with their high substrate affinity, metabolic versatility, and low N_2O emission potential, are emerging as key players in advanced BNR systems, particularly under low-DO and low-ammonia conditions. AOA, while less well understood in engineered systems, also offer advantages in terms of substrate affinity and GHG mitigation.

Mechanistically, the absence of canonical denitrification genes in comammox and most AOA underpins their low N_2O emissions, in stark contrast to AOB. This genetic distinction has profound implications for process design and sustainability, as the selection or enrichment of low-N_2O-emitting nitrifiers can contribute to net-zero GHG goals in wastewater treatment. Explicit, standardized comparisons now provide quantitative benchmarks for N_2O yields across nitrifier groups, supporting evidence-based process optimization.

However, significant research gaps remain, particularly regarding the in situ activity, regulation, and ecological interactions of comammox and AOA. Methodological challenges in detection and quantification, as well as uncertainties in translating laboratory findings to full-scale systems, underscore the need for improved molecular tools and integrated multi-omics approaches. The influence of environmental inhibitors, intra-group diversity, and the economic feasibility of community manipulation for N_2O mitigation require further investigation. Future research should prioritize functional assays, advanced -omics, and process-scale studies to harness the full potential of these microorganisms for sustainable nitrogen removal and climate mitigation in wastewater treatment.

References

1. Xu, SY et al. (2022). Survival strategy of comammox bacteria in a wastewater nutrient removal system with sludge fermentation liquid as additional carbon source. SCI TOTAL ENVIRON.

2. Hu, PF et al. (2025). The core anammox redox reaction system of 12 anammox bacterial genera and their evolution and application implications. WATER RES.

3. Cotto, I et al. (2020). Long solids retention times and attached growth phase favor prevalence of comammox bacteria in nitrogen removal systems. nan.

4. Huang, T et al. (2022). Comammox Nitrospira Bacteria Are Dominant Ammonia Oxidizers in Mainstream Nitrification Bioreactors Emended with Sponge Carriers. nan.
5. Liu, GQ et al. (2021). Long-Term Low Dissolved Oxygen Operation Decreases N2O Emissions in the Activated Sludge Process. ENVIRON SCI TECHNOL.
6. Lehtovirta-Morley, LE et al. (2018). Ammonia oxidation: Ecology, physiology, biochemistry and why they must all come together. nan.
7. He, SS et al. (2022). Comammox bacteria predominate among ammonia-oxidizing microorganisms in municipal but not in refinery wastewater treatment plants. nan.
8. Annavajhala, MK et al. (2018). Comammox Functionality Identified in Diverse Engineered Biological Wastewater Treatment Systems. nan.
9. Zheng, MS et al. (2023). Ubiquitous occurrence and functional dominance of comammox Nitrospira in full-scale wastewater treatment plants. nan.
10. Han, P et al. (2019). Specific Micropollutant Biotransformation Pattern by the Comammox Bacterium Nitrospira inopinata. ENVIRON SCI TECHNOL.
11. Liu, T et al. (2024). Sustainable wastewater management through nitrogen-cycling microorganisms. NAT WATER.
12. Roots, P et al. (2019). Comammox Nitrospira are the dominant ammonia oxidizers in a mainstream low dissolved oxygen nitrification reactor. nan.
13. Mehrani, MJ et al. (2020). The occurrence and role of Nitrospira in nitrogen removal systems. nan.
14. Lin, ZM et al. (2022). Nitrous Oxide Emission from Full-Scale Anammox-Driven Wastewater Treatment Systems. LIFE-BASEL.
15. Al-Ajeel, S et al. (2022). Ammonia-oxidizing archaea and complete ammonia-oxidizing Nitrospira in water treatment systems. nan.

16. He, YY et al. (2023). Net-zero greenhouse gas emission from wastewater treatment: Mechanisms, opportunities and perspectives. RENEW SUST ENERG REV.
17. Wen, JF et al. (2020). Nitrous oxide emission and microbial community of full-scale anoxic/ aerobic membrane bioreactors at low dissolved oxygen setpoints. J WATER PROCESS ENG.
18. Yao, H et al. (2022). Contribution of nitrous oxide to the carbon footprint of full-scale wastewater treatment plants and mitigation strategies- a critical review. ENVIRON POLLUT.
19. Ni, BJ et al. (2013). Mathematical Modeling of Nitrous Oxide (N2O) Emissions from Full-Scale Wastewater Treatment Plants. ENVIRON SCI TECHNOL.
20. Valkova, T et al. (2021). A method to estimate the direct nitrous oxide emissions of municipal wastewater treatment plants based on the degree of nitrogen removal. J ENVIRON MANAGE.
21. Massara, TM et al. (2017). A review on nitrous oxide (N2O) emissions during biological nutrient removal from municipal wastewater and sludge reject water. SCI TOTAL ENVIRON.
22. Thwaites, BJ et al. (2021). Analysis of nitrous oxide emissions from aerobic granular sludge treating high saline municipal wastewater. SCI TOTAL ENVIRON.
23. Massara, TM et al. (2018). Development of an ASM2d-N2O model to describe nitrous oxide emissions in municipal WWTPs under dynamic conditions. CHEM ENG J.
24. Lotito, AM et al. (2012). Nitrous oxide emissions from the oxidation tank of a pilot activated sludge plant. WATER RES.
25. Zhao, YW et al. (2024). Impact of influent characteristics and operational parameters on nitrous oxide emissions in wastewater treatment: Strategies for mitigation and microbial insights. CURR RES BIOTECHNOL.
26. An, ZM et al. (2024). Nitrous oxide emissions in novel wastewater treatment processes: A comprehensive review. BIORESOURCE TECHNOL.

27. Han, P et al. (2021). N2O and NOy production by the comammox bacterium Nitrospira inopinata in comparison with canonical ammonia oxidizers. WATER RES.
28. Ni, BJ et al. (2015). Recent advances in mathematical modeling of nitrous oxides emissions from wastewater treatment processes. WATER RES.
29. Sun, HS et al. (2022). Impact of influent strengths on nitrous oxide emission and its molecular mechanism in constructed wetlands treating swine wastewater. ENVIRON RES.
30. Boiocchi, R et al. (2025). Trends of N2O production during decentralized wastewater treatment: A critical review. J ENVIRON CHEM ENG.
31. Vasilaki, V et al. (2019). A decade of nitrous oxide (N2O) monitoring in full-scale wastewater treatment processes: A critical review. WATER RES.
32. Zhou, X et al. (2020). Unravelling nitrogen removal and nitrous oxide emission from mainstream integrated nitrification-partial denitrification-anammox for low carbon/nitrogen domestic wastewater. J ENVIRON MANAGE.
33. Mao, WL et al. (2022). Identification of key water parameters and microbiological compositions triggering intensive N2O emissions during landfill leachate treatment process. SCI TOTAL ENVIRON.
34. Shang, ZX et al. (2024). Direct and indirect monitoring methods for nitrous oxide emissions in full-scale wastewater treatment plants: A critical review. J ENVIRON MANAGE.
35. Duan, HR et al. (2020). Mitigating nitrous oxide emissions at a full-scale wastewater treatment plant. WATER RES.
36. Johnston, J et al. (2024). Metatranscriptomic Analysis Reveals Synergistic Activities of Comammox and Anammox Bacteria in Full-Scale Attached Growth Nitrogen Removal System. nan.
37. Zhang, Y et al. (2023). Hot spring distribution and survival mechanisms of thermophilic comammox Nitrospira. ISME J.

38. Su, ZC et al. (2023). Nitrite Oxidation in Wastewater Treatment: Microbial Adaptation and Suppression Challenges. ENVIRON SCI TECHNOL.
39. Law, YY et al. (2019). High Dissolved Oxygen Selection against Nitrospira Sublineage I in Full-Scale Activated Sludge. nan.
40. Jung, MY et al. (2022). Ammonia-oxidizing archaea possess a wide range of cellular ammonia affinities. ISME J.
41. Ma, Y et al. (2009). Achieving nitrogen removal via nitrite in a pilot-scale continuous pre-denitrification plant. nan.
42. Duan, HR et al. (2021). Insights into Nitrous Oxide Mitigation Strategies in Wastewater Treatment and Challenges for Wider Implementation. ENVIRON SCI TECHNOL.
43. Xie, YW et al. (2023). N2O emission reduction in the biological nitrogen removal process for wastewater with low C/N ratios: mechanisms and strategies. FRONT BIOENG BIOTECH.
44. Wang, DX et al. (2024). Ammonia oxidizing bacteria (AOB) denitrification and bacterial denitrification as the main culprit of high N2O emission in SBR with low C/ N ratio wastewater. J ENVIRON MANAGE.
45. Kim, SW et al. (2010). Nitrous oxide emission from nitrifying activated sludge dependent on denitrification by ammonia-oxidizing bacteria. BIORESOURCE TECHNOL.
46. Yu, R et al. (2018). Nitrosomonas europaea adaptation to anoxic-oxic cycling: Insights from transcription analysis, proteomics and metabolic network modeling. nan.
47. Castellano-Hinojosa, A et al. (2018). Linking nitrous oxide emissions to population dynamics of nitrifying and denitrifying prokaryotes in four full-scale wastewater treatment plants. CHEMOSPHERE.
48. Siripong, S et al. (2007). Diversity study of nitrifying bacteria in full-scale municipal wastewater treatment plants. nan.

Table S19. Fact-checking of key arguments in the review task "Ammonia-oxidizing microorganisms and their N2O emission in wastewater"

Arguments	Original statement	Supporting sentences from retrieved documents
AOB have relatively low substrate affinity, exemplified by higher ammonium and oxygen half-saturation constants for Nitrosomonas europaea.	"Their substrate affinities are lower: for instance, Nitrosomonas europaea displays ammonium half-saturation constants (K_m) in the range of 0.077–0.55 mg N/L and oxygen K_m values of 0.3–0.8 mg O_2/L (He et al., 2023)."	"citation_info": "He, YY et al. (2023). Net-zero greenhouse gas emission from wastewater treatment: Mechanisms, opportunities and perspectives. RENEW SUST ENERG REV.", "original_sentence": "On the contrary, the K m values of Nitrosomonas europaea (i.e. AOB) were in the ranges of 0.077-0.55 mg N/L for ammonium and 0.3-0.8 mg O 2 /L for DO, respectively [11] ."
Model-parameter uncertainty in batch/continuous N_2O models can shift emission estimates by >100% across studies.	"Notably, batch and continuous process modeling reveal high variability in key parameters such as the half-saturation constant for nitrite and the reduction factor for N_2O production, which can influence emission estimates by more than 100% across studies (Ni et al., 2015)."	"citation_info": "Ni, BJ et al. (2015). Recent advances in mathematical modeling of nitrous oxides emissions from wastewater treatment processes. WATER RES.", "original_sentence": "For the six single-pathway models of AOB (Models AeD in Table S2), the model parameters were obtained after significant calibration efforts, and thus some of the parameters showed high variation (more than 100%) among case studies during model evaluations (Ni et al., 2011 (Ni et al., , 2013a;; Sp erandio et al., 2014) . Among them, the half saturation constant for nitrite or FNA (K NO2,AOB or K HNO2,AOB for Models A, A1, B, B1) and the reduction factor for N 2 O production (h AOB , for all the six single-pathway models) were most highly variable (see Table S5 in SI) and very influential on N 2 O emissions (Sp erandio et al., 2014) ." "citation_info": "Ni, BJ et al. (2015). Recent advances in mathematical

		modeling of nitrous oxides emissions from wastewater treatment processes. WATER RES.",<br>"original_sentence": "For the six single-pathway models of AOB (Models AeD in Table S2), the model parameters were obtained after significant calibration efforts, and thus some of the parameters showed high variation (more than 100%) among case studies during model evaluations (Ni et al., 2011 (Ni et al., , 2013a;; Sp erandio et al., 2014) . Among them, the half saturation constant for nitrite or FNA (K NO2,AOB or K HNO2,AOB for Models A, A1, B, B1) and the reduction factor for N 2 O production (h AOB , for all the six single-pathway models) were most highly variable (see Table S5 in SI) and very influential on N 2 O emissions (Sp erandio et al., 2014) ."<br>"citation_info": "Ni, BJ et al. (2015). Recent advances in mathematical modeling of nitrous oxides emissions from wastewater treatment processes. WATER RES.",<br>"original_sentence": "Among them, the half saturation constant for nitrite or FNA (K NO2,AOB or K HNO2,AOB for Models A, A1, B, B1) and the reduction factor for N 2 O production (h AOB , for all the six single-pathway models) were most highly variable (see Table S5 in SI) and very influential on N 2 O emissions (Sp erandio et al., 2014) ."

AOA can exhibit very high substrate affinity (very low K_m for ammonium and oxygen), exemplified by Nitrosopumilus maritimus.	"For example, Nitrosopumilus maritimus exhibits ammonium K_m values as low as 0.0016–0.002 mg N/L and oxygen K_m values of 0.064–0.12 mg O_2/L (He et al., 2023)."	"citation_info": "He, YY et al. (2023). Net-zero greenhouse gas emission from wastewater treatment: Mechanisms, opportunities and perspectives. RENEW SUST ENERG REV.",<br>"original_sentence": "It was reported that Nitrosopumilus maritimus (i.e. AOA) had low K m values for ammonium (K m = 0.0016-0.002 mg N/L) and DO (K m = 0.064-0.12 mg O 2 /L)."
Comammox Nitrospira can have extremely high ammonia affinity (very low K_m(app) for NH_3), supporting dominance in oligotrophic/low-substrate niches.	"Kinetic and Physiological Parameters: Comammox Nitrospira, such as N. inopinata, exhibit the highest known substrate affinities among nitrifiers, with K_m(app) for NH_3 as low as 49 nM—lower than all characterized AOB and comparable to or even exceeding the affinity of some AOA (Lehtovirta-Morley et al., 2018; Jung et al., 2022)."	"citation_info": "Lehtovirta-Morley, LE et al. (2018). Ammonia oxidation: Ecology, physiology, biochemistry and why they must all come together. nan.",<br>"original_sentence": "AOB have higher half-saturation constants (K m) and lower substrate affinities than AOA and comammox Nitrospira, with K m (NH 3) ranging between 6 and 11 µM for AOB, 3.6 nM and 4.4 µM for AOA and 49 nM for the comammox bacterium N. inopinata (Martens-Habbena et al. 2009; Prosser and Nicol 2012; Kits et al. 2017)."<br>"citation_info": "Al-Ajeel, S et al. (2022). Ammonia-oxidizing archaea and complete ammonia-oxidizing Nitrospira in water treatment systems. nan.",<br>"original_sentence": "Recent studies examined substrate affinity and kinetics of N. inopinata (pure culture) and Ca. N. kreftii (a highly enriched culture), revealing that both species have high apparent ammonia affinities (reported K m(app) of 49-83 nM and 36 nM NH 3 , respectively), especially compared to AOB (Kits et al., 2017; Sakoula et al., 2021a) ."<br>"citation_info": "Al-Ajeel, S et al. (2022). Ammonia-oxidizing archaea and complete ammonia-oxidizing Nitrospira in water treatment systems. nan.",

		"original_sentence": "Only the marine AOA representative N. maritimus has a higher reported ammonia affinity than N. inopinata (Kits et al., 2017) ."<br>"citation_info": "Jung, MY et al. (2022). Ammonia-oxidizing archaea possess a wide range of cellular ammonia affinities. ISME J.",<br>"original_sentence": "However, recently it was shown that (i) the only comammox isolate Nitrospira inopinata has a K m(app) for NH 3 lower than that of all characterized AOB and (ii) that the K m(app) for NH 3 in a few nonmarine AOA strains is not always orders of magnitude lower than that of AOB [5] ."<br>"citation_info": "Jung, MY et al. (2022). Ammonia-oxidizing archaea possess a wide range of cellular ammonia affinities. ISME J.",<br>"original_sentence": "However, recently it was shown that (i) the only comammox isolate Nitrospira inopinata has a K m(app) for NH 3 lower than that of all characterized AOB and (ii) that the K m(app) for NH 3 in a few nonmarine AOA strains is not always orders of magnitude lower than that of AOB [5] ."
In low C/N wastewater, intermittent aeration can raise total nitrogen removal (≈93.5%) while lowering the N_2O emission factor (0.42% → 0.19%).	"In low C/N wastewater treatment, intermittent aeration not only increased total nitrogen removal efficiency (to ~93.5%) but also reduced the N_2O emission factor from 0.42% (continuous aeration) to 0.19% (Xie et al., 2023)."	"citation_info": "Xie, YW et al. (2023). N2O emission reduction in the biological nitrogen removal process for wastewater with low C/N ratios: mechanisms and strategies. FRONT BIOENG BIOTECH.",<br>"original_sentence": "In the treatment of low C/N (=6) wastewater, compared to continuous aeration, intermittent aeration had a higher TN removal efficiency (93.5% on average), and the N 2 O emission factor decreased from 0.42% of continuous aeration to 0.19%, which promoted PN-D."<br>"citation_info": "Xie, YW et al. (2023). N2O emission reduction in the

		biological nitrogen removal process for wastewater with low C/N ratios: mechanisms and strategies. FRONT BIOENG BIOTECH.",<br>"original_sentence": "In the treatment of low C/N (=6) wastewater, compared to continuous aeration, intermittent aeration had a higher TN removal efficiency (93.5% on average), and the N 2 O emission factor decreased from 0.42% of continuous aeration to 0.19%, which promoted PN-D."
Lowering DO from 1 to 0.5 mg/L can reduce AOB growth rates by 3–26% (with smaller effects on NOB).	"DO and SRT Manipulation: Reducing DO from 1 to 0.5 mg/L can lower AOB growth rates by 3–26%, with a less pronounced effect on NOB (Ma et al., 2009)."	"citation_info": "Ma, Y et al. (2009). Achieving nitrogen removal via nitrite in a pilot-scale continuous pre-denitrification plant. nan.",<br>"original_sentence": "With the Ko values summarized in Table 4 , the AOB growth rate would be lowered by 3-26% when DO is reduced from 1 to 0.5 mg/L. This implies that a longer SRT may be required in order to achieve stable ammonia oxidation under low DO conditions, thus requiring a larger reactor volume. On the other hand, however, there has been strong evidence suggesting that nitrifiers decay at lower rates under low DO conditions (Yuan and Blackall, 2002) , hence enhancing the AOB population. The exact impact of low DO conditions on the AOB population development should be investigated further."<br>"citation_info": "Ma, Y et al. (2009). Achieving nitrogen removal via nitrite in a pilot-scale continuous pre-denitrification plant. nan.",<br>"original_sentence": "The use of low DO concentrations in the aerobic zones is expected to lower the AOB growth rate, albeit to a less extent in comparison to that of NOB."

Operating at low DO may require longer solids retention times (e.g., ~15 days) to maintain stable ammonia oxidation.	“However, low DO may require longer SRTs (e.g., 15 days) to maintain stable ammonia oxidation, especially under variable loading (Ma et al., 2009).”	"citation_info": "Ma, Y et al. (2009). Achieving nitrogen removal via nitrite in a pilot-scale continuous pre-denitrification plant. nan.", "original_sentence": "This study demonstrated that an SRT of 15 days is adequate for achieving stable ammonia oxidation in a typical A/O system under DO conditions of 0.5-0.7 mg/L at 21 C." "citation_info": "Ma, Y et al. (2009). Achieving nitrogen removal via nitrite in a pilot-scale continuous pre-denitrification plant. nan.", "original_sentence": "A higher SRT may be required to achieve high-level ammonia removal at all times including peak load conditions."
Recent standardized comparisons report distinct N_2O yields across nitrifier groups (AOB 0.51%; AOA 0.068–0.158%; comammox 0.07–0.2%).	“These findings are corroborated by An et al. (2024), who report N_2O yields of 0.51% for AOB, 0.068–0.158% for AOA, and 0.07–0.2% for comammox.”	"citation_info": "An, ZM et al. (2024). Nitrous oxide emissions in novel wastewater treatment processes: A comprehensive review. BIORESOURCE TECHNOL.", "original_sentence": "Previous studies reported N 2 O yields of 0.51% for AOB, a range of 0.068-0.158% for AOA, and 0.07-0.2% for Comammox (Han et al., 2021; Kits et al., 2019) ." "citation_info": "Han, P et al. (2021). N2O and NOy production by the comammox bacterium Nitrospira inopinata in comparison with canonical ammonia oxidizers. WATER RES.", "original_sentence": "These results are generally consistent with previous findings, where yields of N 2 O during aerobic ammonia oxidation for AOB were reaching levels of up to 0.51%, and those for AOA range from 0.068 % to 0.158% (Hink et al., 2017 ; Stieglmeier et al., 2014) . AOB generate more N 2 O

		than AOA in various ecosystems (Hink et al., 2018 ; Hink et al., 2017 ; Meinhardt et al., 2018) . Comammox N. inopinata in this study exhibited different N 2 O/NH 3 yields (~0.2%) compared to previous work (~0.07%) (Kits et al., 2019) ."<br>"citation_info": "Han, P et al. (2021). N2O and NOy production by the comammox bacterium Nitrospira inopinata in comparison with canonical ammonia oxidizers. WATER RES.",<br>"original_sentence": "These results are generally consistent with previous findings, where yields of N 2 O during aerobic ammonia oxidation for AOB were reaching levels of up to 0.51%, and those for AOA range from 0.068 % to 0.158% (Hink et al., 2017 ; Stieglmeier et al., 2014) ."

Table S20. Case study of AI-powered wastewater engineering consultancy

**Case study:**<br>Wastewater Treatment Plant Near-Zero Carbon Emission Engineering Problem<br>Background<br>A municipal government plans to upgrade an existing wastewater treatment plant with the goal of achieving near-zero carbon emissions by 2030. The plant currently operates a five-reactor Modified Ludzack-Ettinger (MLE) nitrogen removal process with the following configuration:<br>Current Process Configuration:<br>Primary Treatment: Sedimentation tank<br>Secondary Treatment: Five-reactor system (R1-R2 anoxic → R3-R5 aerobic)<br>Internal recycle: 60,000 m^3 /d (R5→R1)

DO control: 2.0 mg/L setpoint in aerobic reactors
Sludge Treatment: Gravity thickening → Anaerobic digestion (35° C) → Dewatering
No energy recovery: CH_4 generation (1,059 kg/d) currently not utilized
Current Status Data
Influent Water Quality Characteristics:
Total Chemical Oxygen Demand (TCOD): 592.5 mg COD/L
Ammonium nitrogen (NH_4^+): 23.9 mg N/L
Total Kjeldahl Nitrogen (TKN): 45.6 mg N/L
Total Suspended Solids (TSS): 380.3 mg/L
Flow rate: 20,648 m^3 /d
Effluent Quality:
Primary clarifier effluent tCOD: 349 mg COD/L
WWTP effluent total nitrogen: 9.91 mg N/L
Current Energy Consumption:
Mixing energy: 768 kWh/d
Aeration energy: 4,000 kWh/d
Pump energy: 441.5 kWh/d
Heating energy: 4,177 kWh/d
Methane generation: 1,059 kg CH_4/d
Sludge Production Data:
Sludge treated with anaerobic digestion: 7,992 kg/d (dry weight)
Sludge produced for disposal: 2,683 kg/d (dry weight)
Sludge disposal volume: 9.58 m^3 /d
Sludge disposal concentration: 280,000 mg SS/L
Nitrogen in digested sludge: 31.46 kgN/d

Phosphorus in digested sludge: 11.54 kgP/d (N/P ratio = 2.72)<br>Energy and Emission Parameters:<br>Electricity carbon intensity: 0.334 kgCO_2-eq/kWh<br>N_2O emission factor for wastewater treatment: 0.011 kg N_2O-N/kgN<br>Electricity generation efficiency from biogas: 35%<br>Current Carbon Emission Inventory:<br>Scope 1 Fugitive N_2O emissions: 136.9 gCO_2-eq/m^3<br>Scope 1 Fugitive CH_4 emissions: 139.9 gCO_2-eq/m^3<br>Scope 1 Fuel emissions: 96.9 gCO_2-eq/m^3<br>Scope 2 Energy consumption emissions: 78.1 gCO_2-eq/m^3<br>Total emissions: 439.6 gCO_2-eq/m^3<br>Engineering Question<br>Question: Near-Zero Carbon Emission Technology Solutions<br>To achieve the 2030 near-zero carbon emission goal, please propose a comprehensive technical engineering report
**GPT 4.1**
Achieving near-zero carbon emissions in wastewater treatment plants (WWTPs) by 2030 necessitates a multifaceted approach that integrates energy efficiency, renewable energy generation, greenhouse gas (GHG) mitigation, and resource recovery. Below is a comprehensive technical engineering report outlining strategies to transform the existing WWTP into a near-zero carbon emission facility.<br>Energy Efficiency Enhancements<br>1.1. Aeration System Optimization<br>Current Status:<br>Aeration energy consumption: 4,000 kWh/day<br>Proposed Actions:<br>Implement Advanced Aeration Controls: Deploy real-time monitoring and control systems to adjust aeration rates based on real-time oxygen demand, reducing unnecessary energy use.

Upgrade to High-Efficiency Aerators: Replace existing aerators with high-efficiency models that offer better oxygen transfer rates and lower energy consumption.
Expected Outcomes:
Potential energy savings of 20-50% in aeration processes, translating to reductions of 800 to 2,000 kWh/day.
1.2. Pumping System Improvements
Current Status:
Pump energy consumption: 441.5 kWh/day
Proposed Actions:
Install Variable Frequency Drives (VFDs): Equip pumps with VFDs to adjust motor speed based on real-time flow requirements, enhancing energy efficiency.
Regular Maintenance and Upgrades: Conduct routine maintenance and consider replacing outdated pumps with energy-efficient models.
Expected Outcomes:
Energy savings of approximately 15-25%, equating to reductions of 66 to 110 kWh/day.
Renewable Energy Integration
2.1. Biogas Utilization
Current Status:
Methane generation: 1,059 kg CH_4/day
Methane currently not utilized
Proposed Actions:
Install Combined Heat and Power (CHP) Units: Utilize biogas produced from anaerobic digestion to generate electricity and heat.
Biogas Cleaning and Upgrading: Implement systems to remove impurities from biogas, enhancing its quality for energy production.
Calculations:
Energy content of methane: 55.5 MJ/kg
Total energy potential: 1,059 kg/day $\times$ 55.5 MJ/kg = 58,774.5 MJ/day
Assuming 35% conversion efficiency:

Electrical energy generated: 58,774.5 MJ/day × 0.35 = 20,570.58 MJ/day
Converted to kWh: 20,570.58 MJ/day ÷ 3.6 MJ/kWh = 5,714.05 kWh/day
Expected Outcomes:
Generation of approximately 5,714 kWh/day, potentially offsetting the plant's total energy consumption of 9,386.5 kWh/day.
Reduction in Scope 2 emissions by decreasing reliance on grid electricity.
2.2. Solar Photovoltaic (PV) Systems
Proposed Actions:
Install Solar Panels: Deploy solar PV systems on available plant infrastructure and adjacent land to generate renewable electricity.
Expected Outcomes:
Depending on system size and local solar irradiance, solar PV can further offset grid electricity usage, contributing to emission reductions.
Greenhouse Gas Mitigation
3.1. Nitrous Oxide (N_2O) Emission Reduction
Current Status:
Fugitive N_2O emissions: 136.9 g CO_2-eq/m^3
Proposed Actions:
Implement N_2O Monitoring and Control: Deploy real-time monitoring systems to detect and control N_2O emissions.
Optimize Nitrification/Denitrification Processes: Adjust operational parameters to minimize conditions that favor N_2O production.
Expected Outcomes:
Reduction in N_2O emissions, which have a global warming potential 298 times that of CO_2.
3.2. Methane (CH_4) Emission Reduction
Current Status:
Fugitive CH_4 emissions: 139.9 g CO_2-eq/m^3
Proposed Actions:
Capture and Utilize Biogas: As outlined in Section 2.1, capturing and utilizing biogas will reduce methane emissions.
Leak Detection and Repair: Regularly inspect and repair leaks in anaerobic digesters and gas handling systems.

Expected Outcomes:
Significant reduction in CH_4 emissions, contributing to overall GHG mitigation.
Resource Recovery and Circular Economy
4.1. Biosolids Management
Current Status:
Sludge produced for disposal: 2,683 kg/day (dry weight)
Proposed Actions:
Class A Biosolids Production: Implement advanced treatment processes to produce Class A biosolids suitable for land application.
Biosolids Reuse: Utilize treated biosolids as soil amendments in agriculture or for land reclamation.
Expected Outcomes:
Reduction in disposal costs and associated emissions.
Creation of a valuable product from waste streams.
4.2. Water Reuse
Proposed Actions:
Implement Tertiary Treatment: Upgrade treatment processes to produce high-quality effluent suitable for non-potable reuse applications.
Develop Reuse Programs: Establish partnerships with local industries and municipalities to utilize reclaimed water for irrigation, industrial processes, or groundwater recharge.
Expected Outcomes:
Conservation of freshwater resources.
Reduction in energy and emissions associated with potable water production and distribution.
Digitalization and Smart Technologies
Proposed Actions:
Deploy Digital Twin Technology: Create a virtual model of the WWTP to simulate and optimize operations, energy use, and emissions.
Implement Advanced Process Control (APC): Utilize APC systems to enhance process stability and efficiency.
Expected Outcomes:

Improved operational efficiency.<br>Enhanced decision-making capabilities.<br>Further reductions in energy consumption and emissions.<br>Financial and Environmental Impact<br>Financial Considerations:<br>Capital Investment: Initial costs for technology upgrades and installations.<br>Operational Savings: Reduced energy costs from efficiency measures and renewable energy generation.<br>Revenue Streams: Potential income from biogas utilization, biosolids sales, and water reuse programs.<br>Environmental Impact:<br>GHG Emission Reductions: Significant decreases in CO_2, CH_4, and N_2O emissions.<br>Resource Conservation: Enhanced water and nutrient recycling.<br>Sustainability: Alignment with global climate goals and local environmental regulations.<br>Case Studies and References<br>Severn Trent's Net Zero Hub: A UK-based WWTP retrofitted to achieve carbon neutrality through integrated technologies, including biogas utilization and digital twin implementation. (severntrent.com)<br>Windsor's NetZero Wastewater Treatment: A California WWTP achieving net-zero status by using 100% clean energy and reusing all biosolids and recycled water. (townofwindsor.ca.gov)<br>Mannheim Wastewater Treatment Plant: A German facility converting biogas into climate-neutral methanol for use as marine fuel, demonstrating innovative resource recovery. (cleanenergywire.org)<br>By implementing the outlined strategies, the WWTP can transition towards near-zero carbon emissions by 2030, setting a benchmark for sustainable wastewater management.
**NaiveRAG**
Specific Applications or Operational Conditions Demonstrating Superior Performance<br>To achieve near-zero carbon emissions at the municipal wastewater treatment plant (WWTP) by 2030, a comprehensive, multi-pronged engineering strategy is required. This must address all major sources of greenhouse gas (GHG) emissions—particularly N_2O and CH_4—while

maximizing energy recovery and integrating renewable energy sources. Below is a systematic, evidence-based technical report synthesizing the most relevant and actionable solutions from the provided literature.

System Integration for Near-Zero Emissions

Model Framework:
A "water and carbon" near-zero emission WWTP model should be adopted, integrating the main WWTP system with subsystems for renewable energy (e.g., photovoltaic, PV) and wastewater energy recovery. These subsystems interact to achieve energy self-sufficiency and minimize GHG emissions (Zhang, BQ et al., 2024 [1]).

Key Principle:
Coupling energy recovery and renewable energy: The plant should utilize both the potential energy in wastewater (biogas/CH_4) and renewable sources (PV) to offset fossil fuel use and reduce Scope 2 emissions.

Mitigation of Direct (Scope 1) GHG Emissions

a. N_2O Emissions from Biological Nitrogen Removal (BNR)
N_2O is a dominant contributor: In BNR processes (like MLE, A/O, A_2O), N_2O can exceed 50% of total carbon footprint (CF) (Maktabifard, M et al., 2023 [2]).
Mitigation Strategies:
Aeration and Feed Control: Optimizing dissolved oxygen (DO) setpoints and feed regimes can significantly reduce N_2O emissions (He, YY et al., 2023 [3]).
Process Optimization: Rational configuration and real-time control tools can help predict and minimize N_2O and CH_4 emissions (Lancioni, N et al., 2024 [7]).

Alternative Nitrogen Removal: Mainstream Anammox processes, which bypass the need for organic carbon in denitrification, can reduce both N_2O emissions and energy demand (Wu, ZQ et al., 2024 [6]; Zhang, XY et al., 2024 [13]).

Recommendation:
Implement advanced aeration control and consider transitioning to Anammox-based nitrogen removal for low C/N influent, as conventional BNR is both carbon- and energy-intensive under these conditions (Zhang, Y et al., 2024 [12]; Zhang, XY et al., 2024 [13]).

b. CH_4 Emissions from Sludge Treatment
Current Status: 1,059 kg CH_4/d generated but not utilized.
Mitigation and Recovery:
Biogas Capture and Utilization: All biogas from anaerobic digestion should be captured and used for combined heat and power (CHP) generation, achieving up to 35% electricity conversion efficiency (Plant Data; Scherson, YD et al., 2014 [8]).
Fugitive Emissions Control: Ensure digester covers, gas-tight handling, and flare excess gas to minimize leaks (Maktabifard, M et al., 2023 [2]).

Quantitative Impact:
Potential Energy Recovery: 1,059 kg CH_4/d $\approx$ 53 GJ/d (using 50 MJ/kg CH_4), which at 35% efficiency yields $\approx$ 5,140 kWh/d—enough to offset a significant portion of current electricity use (Plant Data).

Reduction of Indirect (Scope 2) Emissions

a. Energy Optimization
Aeration is the largest energy consumer: 4,000 kWh/d (Plant Data).
Strategies:
Fine-tune DO setpoints: Lowering DO from 2.0 mg/L to the minimum required for process stability can reduce aeration energy (Kim, D et al., 2015 [9]).

Upgrade to high-efficiency blowers and diffusers.
Real-time process control: Use hybrid control tools to optimize energy use while maintaining effluent quality (Lancioni, N et al., 2024 [7]).

b. Renewable Energy Integration
PV and Other Renewables: Install on-site PV to supply part or all of the plant's electricity demand, further reducing Scope 2 emissions (Zhang, BQ et al., 2024 [1]).

Process Reconfiguration for Resource Recovery

a. A/B Process with Carbon Capture
High-rate Activated Sludge (A-stage): Captures more organics for anaerobic digestion, increasing biogas yield (Wu, ZQ et al., 2024 [6]).
B-stage (Anammox or shortcut N removal): Handles high-nitrogen, low-carbon effluent from A-stage, reducing need for external carbon and minimizing N_2O (Wu, ZQ et al., 2024 [6]).

b. Microalgae-based Nutrient Recovery
Carbon-negative potential: Microalgae can recover N and P while sequestering CO_2, but economic feasibility is currently limited by high substrate demand (Mubashar, M et al., 2024 [4]).

Sludge Management and Further GHG Reduction
Dewatering and Disposal: Optimize for minimal water content to reduce transport and disposal emissions (Guo, DT et al., 2024 [14]).
Biosolids Gasification: Consider gasification of biosolids for additional energy recovery and volume reduction (Scherson, YD et al., 2014 [8]).

System Engineering and Life Cycle Perspective
Holistic Optimization: Apply system engineering to integrate all process steps, minimize redundancy, and optimize logistics for lowest overall GHG emissions (Guo, DT et al., 2024 [14]).

Life Cycle Assessment (LCA): Use LCA to evaluate and prioritize interventions with the highest GHG abatement per unit cost or effort (Guo, DT et al., 2024 [14]; Maktabifard, M et al., 2023 [15]).

Quantitative and Practical Recommendations

a. Immediate Actions
Capture and utilize all biogas for CHP: This alone can offset most of the plant's electricity demand and reduce fugitive CH_4 emissions.
Optimize aeration and mixing: Implement advanced control to reduce energy use and N_2O emissions.
Upgrade process monitoring: Install real-time sensors for DO, N_2O, and CH_4 to enable dynamic process control.

b. Medium-term Upgrades
Transition to A/B process with Anammox: Reduces both energy and carbon requirements for nitrogen removal.
Install PV panels: Target 100% renewable electricity supply, if feasible.

c. Long-term Innovations
Explore microalgae-based nutrient recovery: As technology matures, this could provide carbon-negative treatment and valuable byproducts.
Consider biosolids gasification: For further energy recovery and minimization of disposal volume.

Expected Outcomes
Energy self-sufficiency: Achievable through biogas CHP and PV integration (Zhang, BQ et al., 2024 [1]; He, YY et al., 2023 [3]).
GHG emission reduction: Up to 76.4% demonstrated in large WWTPs with energy recovery and process optimization (He, YY et al., 2023 [3]).
Near-zero emissions: Defined as emissions as close to zero as possible, recognizing that some residual emissions may remain but can be offset by renewable energy and resource recovery (Zhang, BQ et al., 2024 [1]; Maktabifard, M et al., 2023 [2]).

WaterRAG Final report

Engineering Report: Achieving Near-Zero Carbon Emissions in Municipal Wastewater Treatment by 2030

Updated with Integrated Plant-Specific Emission Uncertainty Quantification, Event-Driven Variability Analysis, and Long-Term Monitoring Strategies

---

Summary

This report addresses the engineering challenge of upgrading a municipal wastewater treatment plant (WWTP) to achieve near-zero carbon emissions by 2030. The plant currently operates a five-reactor Modified Ludzack-Ettinger (MLE) process with conventional aerobic/anaerobic treatment and no energy recovery from biogas. The main challenges identified are: (1) high and under-quantified direct greenhouse gas (GHG) emissions—especially nitrous oxide (N_2O) and methane (CH_4)—with significant uncertainty and event-driven variability; (2) substantial indirect emissions from energy consumption; (3) underutilization of energy recovery opportunities; and (4) critical gaps in site-specific operational data, emission quantification, and long-term monitoring. Recent literature and plant-specific data confirm that direct N_2O and CH_4 emissions often exceed indirect emissions from electricity use, but their quantification is complicated by high site-specific, temporal, and spatial variability, as well as limitations in current monitoring and inventory methods (Chong et al., 2024; Song et al., 2023; Samuelsson et al., 2018; Bühler et al., 2022).

Importantly, new evidence highlights that the uncertainty in GHG emission estimates is substantial, with 95% confidence intervals for plant-level CH_4 and N_2O emissions often spanning ±30–100% of mean values (Li et al., 2024; Mannina et al., 2015; Negi et al., 2022; Song et al., 2023). Event-driven operational factors—such as wet weather, diurnal load changes, and equipment failures—can cause emission spikes several times higher than baseline levels (Marinelli et al., 2021; Ribera-Guardia et al., 2019). These findings underscore the need for robust, long-term, and high-frequency monitoring, as well as adaptive management strategies with clear key performance indicators (KPIs) and verification protocols (Duan et al., 2020; Yao et al., 2022; Vasilaki et al., 2019).

Evidence-based solutions include process reconfiguration (e.g., carbon capture, Anammox, DAMO), advanced energy and methane recovery, digitalization, and system-wide optimization. However, their implementation faces technical, operational, and economic barriers, particularly regarding operational reliability, long-term performance, and integration with legacy infrastructure. Critically, the integration of plant-wide, site-specific GHG quantification, uncertainty analysis, event-driven risk management, and continuous monitoring is essential for effective and sustained mitigation. Implementing these strategies, with careful attention to plant-specific data, operational realities, and solution risks, is

expected to reduce total GHG emissions by over 80%, enable energy self-sufficiency, and set a replicable model for sustainable urban water management.

Current Problems
1. **High and Under-Quantified Direct GHG Emissions (Scope 1) with Significant Uncertainty and Event-Driven Variability**
- **Plant-Specific Emission Breakdown and Quantification Gaps:** The current plant emits 136.9 gCO_2-eq/m^3 as N_2O and 139.9 gCO_2-eq/m^3 as CH_4, with Scope 1 fuel emissions at 96.9 gCO_2-eq/m^3 and Scope 2 (energy) at 78.1 gCO_2-eq/m^3. Direct N_2O and CH_4 emissions together account for over 60% of the total GHG footprint (439.6 gCO_2-eq/m^3), exceeding indirect emissions from electricity use—a trend confirmed in recent LCA studies (Buonocore et al., 2018; Parravicini et al., 2022; Ross et al., 2020).
- **Substantial Uncertainty in Emission Estimates:** **Recent studies demonstrate that the uncertainty in plant-level GHG emission estimates is high, with 95% confidence intervals for CH_4 and N_2O emissions often spanning ±30–100% of the mean (Li et al., 2024; Mannina et al., 2015; Negi et al., 2022; Song et al., 2023).** For example, Mannina et al. (2015) found that the 5th–95th percentile range for N_2O emissions in model predictions varied by a factor of 2.5, and Li et al. (2024) reported that emission factors for CH_4 and N_2O can deviate by up to 100% due to variability in activity data and emission factors. This uncertainty is further compounded by the left-skewed distribution of possible emission outcomes, indicating a non-negligible risk of emissions far exceeding mean estimates (Kelly et al., 2019; Hall et al., 2011).
- **Event-Driven Emission Spikes:** **Operational events such as wet weather, diurnal load changes, and equipment failures can cause GHG emissions to spike by factors of 3–6 compared to baseline levels (Marinelli et al., 2021; Ribera-Guardia et al., 2019).** For instance, Marinelli et al. (2021) observed that CH_4 and N_2O emissions during rain events were 3–6 times higher than during dry periods, due to increased dissolved gases and influent variability. Equipment malfunctions have also been linked to elevated N_2O emissions (Kim et al., 2015; Ribera-Guardia et al., 2019).
- **Temporal and Spatial Variability:** Emissions from individual sources (e.g., digesters, reactors, sludge handling) can vary dramatically over time and space, with short-term or localized measurements failing to capture peak events, leaks, or operational upsets (Song et al., 2023; Bühler et al., 2022; Fredenslund et al., 2018).
- **Measurement and Inventory Limitations:** Current inventories often use generic emission factors and aggregate stepwise estimates, which do not reflect plant-specific conditions or capture unknown emission sources (Moore et al., 2023; Chong et al., 2024). This leads to large

uncertainty bands and potentially misleading inventories, especially when emission factors are based on limited or non-representative datasets (Hall et al., 2011; Negi et al., 2022).

2. **High Indirect GHG Emissions (Scope 2) from Energy Consumption**

- **Electricity Use:** The plant consumes 5,209 kWh/d (mixing, aeration, pumping, heating), resulting in 78.1 gCO_2-eq/m^3 from grid electricity (carbon intensity: 0.334 kgCO_2-eq/kWh). Aeration alone accounts for 4,000 kWh/d, a major energy and indirect emission source (Fang et al., 2024; Geglio et al., 2022).
- **Uncertainty in Indirect Emissions:** While indirect GHG emissions from electricity and chemicals are generally less variable than direct emissions, uncertainty in infrastructure-related emissions can be significant, with 95% confidence intervals ranging from -15% to +34% of mean values (Negi et al., 2022).

3. **Inefficient Sludge and Biogas Management**

- **No Biogas Utilization and Fugitive Emissions:** All biogas is flared or vented, missing the opportunity for energy recovery and further reducing net emissions (Maktabifard et al., 2023). Methane emissions from digester malfunction or venting can increase the net carbon footprint by orders of magnitude (Delre et al., 2019). **Leaks at pressure relief valves and unmonitored sources can contribute to significant, yet often unquantified, CH_4 emissions (Fredenslund et al., 2018).**
- **Sludge Disposal:** 2,683 kg/d of digested sludge is disposed of without resource recovery, contributing to additional emissions and lost nutrient value (Fang et al., 2024).

4. **Process Limitations in Carbon and Nitrogen Removal**

- **Carbon Loss:** Conventional MLE processes oxidize much of the influent organic carbon, limiting its availability for energy recovery (Wu et al., 2024).
- **Denitrification Carbon Demand:** The process relies on organic carbon for denitrification, which is suboptimal for low C/N influent and increases N_2O emissions (Xie et al., 2023).

5. **Critical Gaps in Site-Specific Operational Data, Emission Inventory, and Long-Term Monitoring**

- **Lack of Comprehensive, High-Resolution, and Long-Term Monitoring:** Most emission estimates are based on unit-level or short-term measurements, which may overlook unknown sources and fail to represent whole-plant emissions, especially under fluctuating flow and turbulence (Chong et al., 2024; Samuelsson et al., 2018; Bühler et al., 2022). **Studies show that on-site measurements often account for only

two-thirds of the total plant-integrated emissions, with the remainder arising from unmonitored or inaccessible sources (Samuelsson et al., 2018).** Short-term campaigns are insufficient to capture the full range of emissions induced by operational and seasonal variability (Bühler et al., 2022; Vasilaki et al., 2019).
- **Uncertainty Propagation:** Missing or unreliable operational data (e.g., influent characteristics, process performance) can significantly impair model predictions and emission inventories, leading to high epistemic uncertainty (De Mulder et al., 2018; Janssens-Maenhout et al., 2019; Sun et al., 2024).
- **Lack of Plant-Resolved Emission Factors:** Generalized emission factors do not account for spatial, temporal, and process-specific variability, resulting in large uncertainty bands and potentially misleading inventories (Shukuru et al., 2025; Parravicini et al., 2022; Song et al., 2023).
- **Absence of Long-Term Verification and Adaptive Management:** There is a lack of standardized, long-term monitoring and verification plans with clear KPIs to ensure sustained emission reductions and adaptive management (Duan et al., 2020; Yao et al., 2022; Vasilaki et al., 2019).
6. **Implementation Barriers: Regulatory, Workforce, and Infrastructure Challenges**
- **Regulatory and Policy Barriers:** Strict and sometimes duplicative regulatory requirements, uncertainty in future environmental regulations, and a conservative approach to permitting hinder the adoption of innovative, low-emission technologies (Mihelcic et al., 2017; Goh et al., 2024).
- **Workforce and Capacity Gaps:** There is a shortage of skilled personnel for operation, maintenance, and monitoring of advanced systems, compounded by limited training and career development opportunities (Diaz-Elsayed et al., 2019; Yadav et al., 2021).
- **Integration with Legacy Infrastructure:** High upfront costs, compatibility issues, and the need for phased integration with existing systems present significant challenges to implementing new technologies (Goh et al., 2024; Diaz-Elsayed et al., 2019).

Problem Causes
1. **Biological Nitrogen Removal Process Design, Operational Variability, and Event-Driven Emission Mechanisms**
- **N_2O Formation Mechanisms and Variability:** Conventional nitrification-denitrification pathways in MLE systems are prone to N_2O production, especially under suboptimal DO, fluctuating loads, low C/N ratios, and during transitions between anoxic and aerobic conditions (Du et al., 2022; Valkova et al., 2021; Rodriguez-Garcia et al., 2012). **Scenario analyses and plant-wide models show that changes in DO

setpoints, sludge retention time, and external carbon dosing can significantly alter GHG emissions (Flores-Alsina et al., 2011; Mannina et al., 2019).** For example, increasing DO from 1 to 3 mg/L or altering sludge age can change N_2O emissions by up to 30% (Flores-Alsina et al., 2011).
- **Event-Driven Emission Spikes:** **Wet weather events, diurnal load changes, and equipment failures can cause emission spikes several times higher than baseline (Marinelli et al., 2021; Ribera-Guardia et al., 2019).** During rain events, CH_4 and N_2O emissions were observed to be 3–6 times higher due to increased dissolved gases and influent variability (Marinelli et al., 2021). Malfunctioning plants and equipment failures are consistently linked to higher N_2O emissions (Kim et al., 2015; Ribera-Guardia et al., 2019; Johnson et al., 2022).
- **Measurement and Modelling Limitations:** The complexity of GHG formation mechanisms, combined with site-specific operational variability, leads to significant uncertainty in emission quantification (Mannina et al., 2015; Sweetapple et al., 2013). **Uncertainty analysis using Monte Carlo methods reveals that 95% confidence intervals for N_2O and CH_4 emissions can span ±30–100% of mean values, with N_2O showing particularly high variability (Li et al., 2024; Mannina et al., 2015; Song et al., 2023).** This is further exacerbated by the left-skewed distribution of possible emission outcomes (Kelly et al., 2019; Hall et al., 2011).
- **Operational Data Gaps:** Reliable quantification of plant-wide N_2O and CH_4 emissions requires comprehensive, high-frequency, and long-term monitoring across all reactors and emission points, as emissions can vary significantly between parallel units and over time (Chen et al., 2019; Bühler et al., 2022; Samuelsson et al., 2018).
2. **Aeration and Energy Use**
- **High Oxygen Demand and Control Challenges:** The MLE process requires high aeration to maintain DO at 2.0 mg/L, leading to excessive energy use (Fang et al., 2024). **Improper DO control or sensor failures can result in over-aeration, increased nitrate production, and higher N_2O emissions (Lackner et al., 2014; Duan et al., 2021).** The risk of process upsets is heightened if advanced control systems are not robustly implemented.
- **Energy Mix Variability:** The GHG impact of electricity use is highly sensitive to the regional energy mix, with renewable integration offering significant emission reduction potential (Geglio et al., 2022).
3. **Sludge and Biogas Management**
- **Unrecovered Methane and Emission Variability:** Anaerobic digestion produces CH_4, but lack of gas capture/utilization leads to fugitive emissions. **Leaks at pressure relief valves, unmonitored digesters, and unaccounted sources can cause significant, variable CH_4 emissions,

especially during operational upsets or equipment failures (Fredenslund et al., 2018; Bühler et al., 2022).** Dissolved methane in effluent is a persistent challenge, as it can be emitted downstream if not recovered (Liu et al., 2023).
- **Measurement Uncertainty:** Quantification of fugitive biogas emissions is hampered by the lack of direct measurements, short monitoring periods, and the inaccuracy of flow meters, making mass or flow balance approaches unreliable (Firouzian et al., 2022; Bühler et al., 2022).
- **Sludge Disposal Practices:** Disposal of digested sludge without energy or nutrient recovery increases overall carbon footprint (Fang et al., 2024).
4. **Lack of Carbon Capture and Resource-Oriented Design**
- **Carbon Oxidation:** The current process oxidizes most influent organic carbon, reducing the substrate available for biogas production (Wu et al., 2024).
- **No Carbon Redirection:** Absence of high-rate primary treatment or A-stage processes limits carbon capture for energy recovery (Wu et al., 2024; Ji et al., 2023).
5. **System Fragmentation, Data, and Decision Support Limitations**
- **Siloed Operations and Data Collection Challenges:** Lack of plant-wide, system-engineered optimization prevents synergistic improvements in energy, emissions, and resource recovery (Guo et al., 2024). **Short-term or low-frequency data impairs model predictions and emission inventories, leading to high epistemic uncertainty and underestimation of emissions (De Mulder et al., 2018; Sun et al., 2024; Chong et al., 2024).**
- **Lack of Plant-Resolved Emission Factors:** Generalized emission factors do not account for spatial, temporal, and process-specific variability, resulting in large uncertainty bands and potentially misleading inventories (Shukuru et al., 2025; Parravicini et al., 2022; Song et al., 2023).
- **Absence of Long-Term Verification and Adaptive Management:** There is a lack of standardized, long-term monitoring and verification plans with clear KPIs to ensure sustained emission reductions and adaptive management (Duan et al., 2020; Yao et al., 2022; Vasilaki et al., 2019).
6. **Implementation Barriers: Regulatory, Workforce, and Infrastructure Challenges**
- **Regulatory and Policy Barriers:** Conservative permitting, lack of test data on operational performance, and uncertain future regulations slow technology adoption (Mihelcic et al., 2017; Goh et al., 2024).

- **Workforce and Capacity Gaps:** Shortage of skilled personnel, limited training, and lack of coordination between agencies hinder effective operation and monitoring (Diaz-Elsayed et al., 2019; Yadav et al., 2021).
- **Integration with Legacy Infrastructure:** High upfront costs, compatibility issues, and the need for phased integration with existing systems present significant challenges (Goh et al., 2024; Diaz-Elsayed et al., 2019).

Solution Approaches
1. **Process Reconfiguration for Carbon Capture and Nitrogen Removal**
a. **Adopt A/B Process with High-Rate Carbon Capture**
- **Technical Details:** Implement a high-rate activated sludge (HRAS) or chemically enhanced primary treatment (CEPT) as A-stage to maximize organic carbon capture, followed by anaerobic digestion for energy recovery (Wu et al., 2024; Ji et al., 2023).
- **Expected Impact:** Increases biogas yield by up to 40%, reduces aeration demand, and provides a carbon-lean stream for advanced nitrogen removal (Ji et al., 2023).
- **Implementation Roadmap:** Begin with pilot-scale trials to optimize A-stage operation and assess impacts on downstream processes. **Combine plant-integrated and unit-based GHG measurements to accurately quantify emission reductions and pinpoint major emission sources (Chong et al., 2024).** Address data gaps by implementing continuous, high-frequency, and long-term monitoring to capture operational and seasonal variability, and explicitly quantify uncertainty using Monte Carlo or similar statistical methods (Li et al., 2024; Mannina et al., 2015; Song et al., 2023).
- **Feasibility and Cost-Benefit:** Capital investment is required for new infrastructure, but increased energy recovery and reduced emissions can offset costs. Comprehensive LCA and cost analysis tools should be used to support decision-making, incorporating uncertainty ranges in emission reduction estimates (Kaljunen et al., 2022; Negi et al., 2022).
b. **Implement Mainstream Anammox and/or DAMO-Anammox for Nitrogen Removal**
- **Technical Details:** Replace conventional denitrification with partial nitritation-Anammox (PN/A) or denitrifying anaerobic methane oxidation (DAMO)-Anammox processes in B-stage (He et al., 2023; Du et al., 2022).

- **Design Parameters:** Maintain low DO (<0.5 mg/L), sidestream nitrite supply, and temperature control for Anammox activity (He et al., 2023). **DAMO-Anammox processes can provide greater tolerance to variable feed composition and help polish effluent to meet strict nitrogen limits, as demonstrated in full-scale trials (Lim et al., 2021).**
- **Expected Impact:** Reduces N_2O emissions by up to 80%, cuts aeration energy by 50–60%, and enables energy-neutral or positive operation (Du et al., 2022; He et al., 2023).
- **Implementation Roadmap:** Conduct pilot-scale demonstrations to validate process stability and effluent quality. **Adopt a stepwise, risk-managed implementation strategy: first test in a single reactor, assess impacts on pollutant removal and operational costs, then scale up to all reactors (Duan et al., 2021).** Use mathematical modeling to predict GHG mitigation under different operational scenarios, ensuring models are calibrated with high-resolution, plant-specific data and uncertainty analysis (Seshan et al., 2024; Zhang et al., 2025; Mannina et al., 2015).
- **Feasibility and Cost-Benefit:** Full-scale integration may require significant adaptation of existing systems. **Operational risks include process instability under fluctuating influent conditions and the need for stringent nitrite/ammonium ratios (Lim et al., 2021).** Economic analyses should include capital, O&M, and potential incentives/penalties for NOx emissions, with explicit consideration of uncertainty in emission reduction outcomes (Beckinghausen et al., 2020; Kelly et al., 2019).
c. **Optimize Aeration and DO Control**
- **Technical Details:** Implement advanced DO control (e.g., real-time sensors, variable frequency drives) and switch to high-efficiency diffused aeration (Guo et al., 2024).
- **Expected Impact:** Reduces both energy use and N_2O emissions, with potential 20–30% energy savings (Fang et al., 2024; Sweetapple et al., 2014).
- **Implementation Roadmap:** Upgrade aeration systems in phases, starting with the highest energy-consuming reactors. **Ensure robust risk management: improper DO control can lead to ammonium/nitrite accumulation or process failure (Duan et al., 2021).** Validate performance improvements with plant-integrated GHG and energy monitoring, ensuring sufficient duration and frequency to capture operational variability and event-driven spikes (Bühler et al., 2022; Marinelli et al., 2021).
- **Feasibility and Cost-Benefit:** Sensor reliability and integration with plant-wide control systems are critical; failure in DO control can lead to severe process upsets (Lackner et al., 2014).
2. **Enhanced Energy Recovery and GHG Management**

a. **Biogas Capture and Combined Heat & Power (CHP)**
- **Technical Details:** Install biogas capture, cleaning, and CHP units to utilize all methane from anaerobic digestion for electricity and heat (Maktabifard et al., 2023).
- **Expected Impact:** Offsets >70% of grid electricity, reduces fugitive CH_4 emissions, and enables energy self-sufficiency (Maktabifard et al., 2023; Parravicini et al., 2022).
- **Implementation Roadmap:** Begin with feasibility and pilot-scale studies to optimize gas capture and CHP sizing. **Address operational risks by ensuring robust monitoring and maintenance of digesters and gas handling systems, as leaks and equipment failures can cause emission spikes (Fredenslund et al., 2018; Bühler et al., 2022; Marinelli et al., 2021).** Integrate with plant-wide energy management systems.
- **Feasibility and Cost-Benefit:** High capital and O&M costs, but substantial energy savings and emission reductions. Economic analyses should include incentives for renewable energy and carbon credits, with explicit uncertainty ranges (Beckinghausen et al., 2020; Negi et al., 2022).
b. **Dissolved Methane Recovery from Effluent**
- **Technical Details:** Install membrane contactors or degassing units to recover dissolved methane from digester effluent (He et al., 2023; Liu et al., 2023).
- **Expected Impact:** Further reduces fugitive CH_4 emissions by 10–20% (He et al., 2023).
- **Implementation Roadmap:** Pilot-scale trials to assess operational reliability and integration with existing effluent lines. **Recognize that energy demand for membrane-based recovery increases as targeted dissolved methane concentrations decrease, and process efficiency is governed by concentration gradients (Liu et al., 2023).** Continuous monitoring is essential to capture temporal variability in emissions (Bühler et al., 2022; Song et al., 2023).
- **Feasibility and Cost-Benefit:** Technology is emerging; cost-effectiveness and operational reliability need further demonstration. **Operational risks include membrane fouling, energy costs, and incomplete methane recovery (Shahid et al., 2020; Liu et al., 2023).**
c. **GHG Valorization**
- **Technical Details:** Explore N_2O and CH_4 recovery for use as energy sources or chemical feedstocks (He et al., 2023; Du et al., 2022).
- **Limitations:** N_2O recovery is technically challenging at full scale, but CH_4 management is feasible and impactful (He et al., 2023).
3. **Sludge and Resource Management**

a. **Optimize Sludge Treatment for Energy and Nutrient Recovery**
- **Technical Details:** Enhance anaerobic digestion (e.g., co-digestion, thermal hydrolysis pre-treatment) to maximize biogas yield and reduce residual sludge (Guo et al., 2024).
- **Expected Impact:** Reduces sludge disposal volume, offsets fertilizer use, and further cuts GHG emissions.
- **Implementation Roadmap:** Pilot-scale studies to optimize pre-treatment and co-digestion recipes. Use LCA and cost analysis tools to evaluate environmental and economic benefits, including uncertainty analysis (Kaljunen et al., 2022; Negi et al., 2022).
b. **Consider Hydrothermal Processes for Sludge with High Water Content**
- **Technical Details:** For wet sludge, hydrothermal oxidation can achieve volume reduction and energy recovery with lower net emissions than incineration (Guo et al., 2024).
- **Expected Impact:** Achieves collaborative management of wastewater and sludge for net-zero emissions.
4. **Energy and Carbon Source Optimization**
a. **Transition to Renewable Electricity**
- **Technical Details:** Procure green electricity from the grid or install on-site solar PV to further reduce Scope 2 emissions (Valkova et al., 2021; Geglio et al., 2022).
- **Expected Impact:** Reduces indirect emissions, especially as direct emissions become dominant.
b. **Optimize Chemical Dosing and Transportation**
- **Technical Details:** Use real-time process monitoring to minimize chemical use and optimize logistics (Guo et al., 2024).
- **Expected Impact:** Marginal but relevant reduction in Scope 3 emissions.
5. **System Engineering, Plant-Wide Optimization, and Long-Term Monitoring & Verification**
a. **Adopt Plant-Wide Modeling, Monitoring, and Control with Uncertainty Quantification**
- **Technical Details:** Implement digital twins and plant-wide models to optimize energy, emissions, and resource recovery in real time (Guo et al., 2024; Mannina et al., 2016). **Integrate both plant-integrated and unit-based GHG measurement approaches to improve emission source identification and total accounting accuracy (Chong et al., 2024).** Ensure models are calibrated with high-resolution, plant-specific data to capture operational and seasonal variability, and explicitly quantify uncertainty using Monte Carlo or similar methods (Li et al., 2024; Mannina et al., 2015; Song et al., 2023).

- **Expected Impact:** Maximizes synergies, ensures compliance with effluent standards, and enables adaptive management.
- **Implementation Roadmap:** Develop and calibrate plant-wide models using integrated, on-site GHG measurement data. Use these models to evaluate mitigation scenarios before full-scale implementation. Address data gaps with advanced data reconciliation and gap-filling techniques (De Mulder et al., 2018). **Incorporate uncertainty analysis and scenario planning to inform robust decision-making (Kelly et al., 2019; Hall et al., 2011).**
- **Feasibility and Cost-Benefit:** Requires reliable, comprehensive data collection and integration across all plant sections (Nguyen et al., 2020; Mannina et al., 2016). Investment in decision-support tools (e.g., LCA, cost analysis) is essential for informed technology selection (Kaljunen et al., 2022).
b. **Long-Term Monitoring, Verification, and Adaptive Management**
- **Technical Details:** Establish standardized, long-term monitoring and verification plans with clear KPIs for GHG emissions, energy use, effluent quality, and operational costs (Duan et al., 2020; Yao et al., 2022; Sweetapple et al., 2014). **KPIs should include: (1) total and specific GHG emissions (with 95% confidence intervals), (2) frequency and magnitude of emission spikes, (3) energy self-sufficiency ratio, (4) effluent compliance rates, and (5) operational cost per unit treated.**
- **Implementation Roadmap:**
  1. **Baseline Assessment:** Conduct plant-integrated GHG measurements and develop a robust emission inventory, using facility-level, plant-resolved emission factors and explicit uncertainty ranges (Chong et al., 2024; Shukuru et al., 2025; Li et al., 2024).
  2. **Pilot-Scale Trials:** Implement pilot-scale demonstrations of key technologies (e.g., A/B process, Anammox, advanced digestion, biogas recovery) to validate performance and emissions reduction, ensuring sufficient duration and frequency to capture operational and seasonal variability and event-driven spikes (Bühler et al., 2022; Samuelsson et al., 2018; Marinelli et al., 2021).
  3. **Modeling and Decision Support:** Use mathematical models and LCA/cost tools to evaluate scenarios and guide investment, ensuring models are calibrated with high-resolution, plant-specific data and uncertainty analysis (Seshan et al., 2024; Zhang et al., 2025; Mannina et al., 2015).
  4. **Phased Full-Scale Implementation:** Roll out proven solutions in phases, prioritizing high-impact upgrades and ensuring compatibility with legacy infrastructure (Goh et al., 2024).

5. **Continuous Monitoring and Adaptive Management:** Maintain plant-wide GHG and energy monitoring, regularly update emission inventories with uncertainty ranges, and adapt operations based on KPI trends and event-driven risk assessments (Duan et al., 2020; Yao et al., 2022; Vasilaki et al., 2019).
- **Justification and Case Studies:** Long-term monitoring and adaptive management have been shown to validate and sustain emission reductions in full-scale plants (Duan et al., 2020). Standardized, site-specific monitoring is critical for accurate emission factors and effective mitigation (Yao et al., 2022; Vasilaki et al., 2019).
c. **Addressing Implementation Barriers: Regulatory, Workforce, and Infrastructure Integration**
- **Regulatory Alignment:** Engage with policymakers to develop and update regulations that support innovation and align with technological advancements, while streamlining permitting processes (Mihelcic et al., 2017; Goh et al., 2024).
- **Workforce Development:** Invest in education and training programs to build the necessary skills for operation, maintenance, and monitoring of advanced systems. Collaborative programs with institutes and industries can build a talent pipeline (Goh et al., 2024; Diaz-Elsayed et al., 2019; Yadav et al., 2021).
- **Infrastructure Integration:** Plan for phased integration with legacy systems, leveraging public-private partnerships and international grants to offset high upfront costs. Customize solutions to local contexts and conduct thorough feasibility studies to ensure adaptability and scalability (Goh et al., 2024; Diaz-Elsayed et al., 2019).

Expected Outcomes and Benefits
- **GHG Emission Reduction:** Integrated solutions can reduce total GHG emissions by 80–100% (Guo et al., 2024; Fang et al., 2024; Parravicini et al., 2022), with explicit uncertainty ranges provided for all estimates.
- **Energy Self-Sufficiency:** Biogas utilization and process optimization can offset >70% of energy demand, with potential for net energy-positive operation (Maktabifard et al., 2023).
- **Resource Recovery:** Enhanced sludge and nutrient management supports circular economy goals and reduces environmental impact (Fang et al., 2024).
- **Regulatory Compliance:** Advanced nitrogen removal (Anammox/DAMO) ensures effluent quality while minimizing emissions (He et al., 2023; Lim et al., 2021).

- **Decision Support:** Use of plant-wide modeling, LCA, cost analysis, and uncertainty quantification enables informed, adaptive management and investment planning (Kaljunen et al., 2022; Kelly et al., 2019).
- **Replicability:** The proposed system engineering approach provides a scalable model for other municipal WWTPs.

References

Chen, Y., Wu, J.P., Zhao, J.W., Yang, H.Z., Dovom, H.A., Sivakumar, M., Jiang, G.M. 2025. A critical review of sulfide and methane control in urban sewer systems using nitrogen compounds. *Water Research*, **277**.

Liu, T., Duan, H.R., Luecker, S., Zheng, M., Daims, H., Yuan, Z.G., Guo, J.H. 2024. Sustainable wastewater management through nitrogen-cycling microorganisms. *Nature Water*, **2**(10), 936-952.

Ran, X.C., Wang, T., Zhou, M.D., Li, Z.B., Wang, H., Tsybekmitova, G.T., Guo, J.H., Wang, Y.Y. 2025. A Novel Perspective on the Instability of Mainstream Partial Nitrification: The Niche Differentiation of Nitrifying Guilds. *Environmental Science & Technology*, **59**(18), 8922-8938.

Shah, A.A., Walia, S., Kazemian, H. 2024. Advancements in combined electrocoagulation processes for sustainable wastewater treatment: A comprehensive review of mechanisms, performance, and emerging applications. *Water Research*, **252**.

Song, C.H., Zhu, J.J., Yuan, Z.G., van Loosdrecht, M.C.M., Ren, Z.J. 2024. Defining and achieving net-zero emissions in the wastewater sector. *Nature Water*, **2**(10), 927-935.
